# Prevalence of locoregional and distant lymph node metastases in children and adolescents/young adults with soft tissue sarcomas: a Bayesian meta-analysis of proportions

**DOI:** 10.1016/j.eclinm.2025.103390

**Published:** 2025-08-07

**Authors:** Illya Martynov, Luca Tobi, Annika Strömer, Andreas Mayr, Amadeus T. Heinz, Martin Ebinger, Monika Sparber-Sauer, Joerg Fuchs, Reza M. Vahdad, Guido Seitz

**Affiliations:** a Department of Pediatric Surgery and Urology, Centre for Pediatric Surgery, Philipps-University, University Hospital Giessen-Marburg, Baldingerstraße, Marburg 35043, Germany; b Department of Pediatric Surgery, Centre for Pediatric Surgery, University Hospital Giessen-Marburg, Rudolf-Buchheim-Strasse 7, Giessen 35392, Germany; c Department for Medical Biometry and Statistics, Philipps-University Marburg, Marburg 35043, Germany; d Pädiatrie 5 (Pädiatrische Onkologie, Hämatologie, Immunologie), Klinikum der Landeshauptstadt Stuttgart gKAöR, Olgahospital, Stuttgart Cancer Center, Zentrum für Kinder-, Jugend- und Frauenmedizin, Stuttgart, Germany; e Department of Pediatric Hematology and Oncology, University Children's Hospital, Tuebingen, Germany; f Department of Pediatric Surgery and Pediatric Urology, University Children’s Hospital, Tuebingen, Germany

**Keywords:** Lymph node metastases, Soft tissue sarcomas, Rhabdomyosarcoma, ‘Non-rhabdomyosarcoma’ soft tissue sarcoma, Bayesian meta-analysis

## Abstract

**Background:**

The presence of both regional and distant lymph node metastases (LNM) in paediatric and adolescent/young adult (AYA) patients with soft tissue sarcomas (STS) significantly impacts clinical outcomes. However, reported rates of LNM vary widely across the literature and are often accompanied by substantial uncertainty. We aimed to quantitatively synthesise global proportions of LNM across different histological subtypes and tumour sites in this population.

**Methods:**

In this meta-analysis, we systematically searched MEDLINE, Scopus, and Web of Science from inception until May 1, 2024 (updated on June 1, 2025) for studies published in English that reported LNM rates in patients with STS aged 0–21 years. Eligible study designs included cohort studies, case-control studies, case series, and randomised controlled trials. Patient-level data were not requested from study authors. LNM had to be confirmed clinically, by imaging, or histologically. We excluded reviews, editorials, and case reports with fewer than three patients. We conducted a random-effects Bayesian meta-analysis using a logit transformation to synthesise LNM proportions. The posterior distributions of LNM prevalence were summarised by the posterior mean and 95% credible intervals (CrIs). Study quality was assessed across seven domains: confounding, participant selection, exposure classification, deviations from intended exposures, missing data, outcome measurement, and selective reporting.

**Findings:**

Of 3969 records screened, 263 articles were included in the data synthesis. These comprised 147 studies on rhabdomyosarcoma (RMS), 106 on non-rhabdomyosarcoma soft tissue sarcoma (NRSTS), and 10 on mixed RMS/NRSTS cohorts, representing 53,093 patients with STS. The pooled posterior mean proportion of LNM in patients with RMS (n = 41,547) was 0.228 (95% CrI: 0.202–0.255), with the highest rates observed in patients with alveolar RMS (posterior mean proportion: 0.370; 95% CrI: 0.276–0.473). Subgroup analysis by RMS primary site revealed the highest LNM rates in the perianal/perineal region (0.466; 95% CrI: 0.397–0.537), extremity (0.281; 0.210–0.363), and non-parameningeal head/neck region (0.259; 0.167–0.376). Among patients with NRSTS (n = 11,546), the pooled posterior mean proportion of LNM was 0.111 (95% CrI: 0.092–0.133), with desmoplastic small round cell tumour (0.440; 0.335–0.552), clear cell sarcoma (0.212; 0.163–0.275), and malignant rhabdoid tumour (0.199; 0.141–0.273) showing the highest rates. Most analyses had moderate-to-high heterogeneity (95% CrI for tau: 0.7443–1.1139).

**Interpretation:**

Our Bayesian meta-analysis synthesises global evidence on the prevalence of LNM in paediatric and AYA patients with STS, highlighting the significant heterogeneity in LNM rates by histological subtype, particularly in NRSTS, and by tumour location, especially in RMS. Future studies should aim to standardise lymph node staging protocols and reporting practices to improve classification accuracy and enhance comparability across studies.

**Funding:**

None.

**Research in context**

**Evidence before this study**

While lymph node status is an established prognostic factor in paediatric soft tissue sarcomas (STS), reported rates of lymph node metastases (LNM) vary widely. To determine the extent of existing evidence and the need for quantitative synthesis, we searched MEDLINE, Scopus, and Web of Science from database inception to May 1, 2024, limited to English-language publications. This yielded 3969 articles, indicating substantial heterogeneity and a lack of pooled prevalence estimates. No previous meta-analysis has provided a comprehensive synthesis of LNM prevalence in paediatric and adolescent/young adult (AYA) populations with STS.

**Added value of this study**

This is the first large-scale systematic review and Bayesian meta-analysis to quantify the global prevalence of LNM across both rhabdomyosarcoma (RMS) and non-rhabdomyosarcoma soft tissue sarcomas (NRSTS) in children and AYA. By including over 53,000 patients from 263 studies, this analysis provides the most comprehensive estimates to date, stratified by histological subtype and tumour location. The Bayesian framework enables probability-based interpretation and accounts for heterogeneity across studies, which is common in rare paediatric cancers.

**Implications of all the available evidence**

Our findings highlight the need for harmonised nodal staging protocols and standardised reporting practices to enable accurate classification and improve comparability across studies. By providing robust prevalence estimates for LNM, they offer a valuable evidence base to guide clinical decision-making and support evidence-based guideline development. Future studies should aim to standardise lymph node staging protocols and reporting practices to improve classification accuracy and comparability across different tumour entities and anatomical sites.

## Introduction

Soft tissue sarcomas (STS) comprise a heterogeneous group of malignant tumours originating from connective tissues and account for approximately 7% of all childhood cancers.^1^ Within this group, rhabdomyosarcoma (RMS) is the most common subtype, comprising around one half of paediatric STS cases.^2^^,^^3^ The remaining entities can be classified as ‘non-rhabdomyosarcoma’ soft tissue sarcomas (NRSTS), a highly diverse group of mesenchymal tumours characterised by a wide range of histological subtypes and biological behaviours, each exhibiting distinct clinical trajectories.^4^ RMS can originate in various anatomical locations throughout the body, not limited to skeletal muscle, and is historically classified into different histological subtypes, with the most common being alveolar (ARMS) and embryonal (ERMS).^5^ Other less common subtypes include pleomorphic rhabdomyosarcoma (plRMS), almost exclusively seen in adults, and spindle cell/sclerosing rhabdomyosarcoma (scRMS), which can occur in both children and adults.^6^ In addition to the histopathologic categorisation of RMS, molecular investigations have shown that ARMS is frequently associated with chromosomal translocations *t(2;13) or t(1;13)*, which lead to the fusion of *PAX3-FOXO1* or *PAX7-FOXO1*, respectively.^7^ Overall, tumours that are fusion-positive (FP-RMS) tend to exhibit more aggressive behaviour, with a greater likelihood of spreading to lymph nodes (LN) and recurring than fusion-negative RMS (FN-RMS).^8^ Beyond these fusion status–related behavioural differences, recent genomic studies have highlighted distinct mutational patterns within FP-RMS and FN-RMS subtypes. In FP-RMS, alterations are frequently observed in genes such as CDK4 (13%), MYCN (10%), BCOR (6%), NF1 (4%), and TP53 (4%). In contrast, FN-RMS more commonly involves mutations within the RAS signaling pathway, seen in up to 32% of cases—particularly affecting NRAS (17%), KRAS (9%), and HRAS (8%). Overexpression of MYCN and disruptions in TP53 in FP-RMS are linked to a more aggressive clinical course. Similarly, in FN-RMS, alterations in MYOD1 and TP53 are considered markers of unfavorable prognosis.^9^^,^^10^ Along with molecular characterisation, risk classification for treatment allocation incorporates factors such as age at diagnosis (≤10 years *vs.* >10 years), tumour size (≤5 cm *vs.* >5 cm), clinical group (based on the extent of residual tumour after initial surgery, according to the Intergroup Rhabdomyosarcoma Study (IRS)), primary tumour site (favorable *vs.* unfavorable), and LN involvement (N1 *vs.* N0; locoregional *vs.* distant LN disease).^11^, ^12^, ^13^ Locoregional LN involvement refers to nodes located within the expected first-echelon drainage of the primary tumour, whereas any nodal spread beyond these regions is categorised as distant or metastatic.^14^ In metastatic RMS, prognosis is primarily guided by the Oberlin risk factors, which include age ≥10 years, unfavorable primary tumour site, bone marrow involvement, and ≥3 metastatic sites. Each factor is associated with worse survival, and outcomes decline with the number of risk factors present.^15^^,^^16^ Interestingly, patients with exclusively LN involvement beyond the regional lymph node stations—formally classified as metastatic RMS—have better survival compared with those with other metastatic patterns.^17^

LN staging is performed using clinico-radiological methods, typically ultrasound as the initial imaging modality, followed by magnetic resonance imaging (MRI), with or without additional functional techniques such as fluorodeoxyglucose positron emission tomography (FDG PET).^14^^,^^18^ Pathological evaluation of LN, if pursued (for example, in all patients with extremity RMS^19^ or in patients with paratesticular RMS older than 10 years,^20^ regardless of imaging findings), may be performed either through random sampling or through a sentinel lymph node procedure (SLNP).^21^ However, in real-world clinical practice, systematic surgical nodal staging according to established protocols is often performed inconsistently.^22^ Furthermore, the propensity for LN metastasis differs between RMS and NRSTS, as well as between different histological subtypes and tumour locations. In RMS, LN involvement can range from 5% in “low-risk” patients^23^ to 64% in perineal/perianal RMS,^24^ and is more commonly seen in fusion-positive ARMS.^25^ Similarly, the primary tumour site influences the risk of lymphatic involvement, with tumours located in the extremities^26^^,^^27^ or perineal/perianal region^28^^,^^29^ being more likely to involve LN compared with tumours in other favorable locations, such as the orbit or nonparameningeal head and neck.^13^^,^^30^ NRSTS also exhibit highly variable patterns of lymphatic spread depending on the specific histological subtype and cytogenetic features, with overall reported rates ranging from 1.75% to 7.5%,^31^ which are generally lower than those observed in RMS. However, certain NRSTS subtypes, such as desmoplastic small round cell tumour (DSRCT), characterised by the *t(11;22) (p13;q12)* translocation resulting in *EWSR1-WT1* oncoprotein,^32^ and synovial sarcoma, which harbors the *t(X;18) (p11.2;q11.2)* translocation and the abnormal *SYT::SSX* fusion transcript,^33^ are more prone to lymphatic dissemination, with LN involvement reported in up to 60% and 29% of cases, respectively.^34^, ^35^, ^36^, ^37^, ^38^, ^39^, ^40^, ^41^

Given the uncertainties and substantial variability in the reported prevalence of LNM across different STS subtypes in the paediatric and AYA age groups, we aimed to comprehensively synthesise the available lymph node data using a Bayesian meta-analysis framework.

## Methods

### Study design and ethics

This meta-analysis was conducted in accordance with the PRISMA guidelines^42^ and the STROBE checklist^43^ for meta-analyses. This study is a meta-analysis of previously published data and, as such, does not involve any direct interaction with patients or the collection of primary data. As a result, ethical approval and consent to participate were not required and were not sought for this study.

### Search strategy

A comprehensive literature search was performed to identify studies reporting on LN status among paediatric/AYA (≤21 years) with STS. The databases searched included MEDLINE (PubMed), Scopus, and Web of Science, from inception until May 1, 2024. This search was updated on June 1, 2025. Search terms and search strategy are shown in Supplementary Table S1. We only included articles available in English.

### Eligibility criteria

Studies were included if they met the following criteria: (1) the examined population consisted entirely or partially of paediatric/AYA patients (0–21 years) with STS; (2) at least three cases were reported; (3) LN assessment was based on clinical findings, imaging (e.g., ultrasound, MRI, CT, FDG-PET/CT, FDG-PET/MRI), and/or histological examination; (4) the cohort included patients with either locoregional LN involvement (localised RMS/NRSTS), LN involvement beyond the regional basin (metastatic disease), or both; (5) the study design was a cohort study, case-control study, case series, or randomised controlled trial; and (6) the full text was available with sufficient data for extraction. Exclusion criteria were: (1) studies focusing exclusively on adult populations (>21 years); (2) reviews, editorials, letters to the editor, book chapters, or case reports with fewer than three patients; and (3) studies not reporting LN status. In accordance with the SAGER guidelines,^44^ we acknowledge that sex and gender were not considered as variables in the design, analysis, or interpretation of this meta-analysis. The vast majority of included studies did not report disaggregated data by sex or gender, and we were therefore unable to perform subgroup analyses based on these factors.

### Data extraction

Two reviewers (IM, LT) independently screened titles and abstracts for eligibility and further examined the main tables and results sections, as LN data were often not explicitly reported in the title or abstract. Full texts of studies deemed potentially eligible were subsequently reviewed to confirm inclusion. A summary of all variables extracted from each study is provided in Supplementary Table S2.

### Outcome measures

The primary outcome was the prevalence of LN involvement among patients with RMS/NRSTS, further stratified by histological subtypes and tumour locations.

### Risk-of-bias and quality assessment

A seven-domain quality assessment (confounding, selection of participants, classification of exposures, deviations from intended exposures, missing data, measurement of outcomes, and selection of the reported result) and a four-tier risk of bias rating (low, moderate, serious, unclear) were applied to each study. Any disagreements were resolved through discussion.

### Statistical analysis

We conducted a Bayesian random-effects meta-analysis on logit-transformed proportions to estimate the prevalence of LNM across studies. The logit transformation was used to stabilise variances and handle proportions near 0% or 100%; a continuity correction of 0.5 was applied to studies reporting 0% or 100% event rates to ensure stable variance estimation across all studies.^45^ For the heterogeneity parameter “tau” (i.e., the between-study standard deviation), we specified a half-normal prior with a scale parameter of 1. The overall effect “mu” (i.e., the average logit-transformed proportion across studies) was modeled using a weakly informative normal prior with mean 0 and standard deviation 4, as implemented in the R add-on package ‘bayesmeta'. Posterior distributions of LN positivity proportions were summarised using the posterior mean and 95% credible intervals (CrIs), representing the range within which the true prevalence is expected to lie with 95% probability. Sensitivity analyses were performed to assess the robustness of findings under different exclusion scenarios: (1) the main analysis included all studies; (2) the overlap exclusion removed studies with overlapping cohorts; (3) the bias exclusion removed studies at serious risk of bias; and (4) the combined exclusion removed both overlapping and high-risk studies. We also plotted posterior samples of key parameters (e.g., mu, tau) to visualise distributional shape and subgroup variation, using trace plots to illustrate sampling variability and heterogeneity patterns across studies. Since bayesmeta derives posterior distributions analytically rather than via Markov Chain Monte Carlo (MCMC) sampling, these trace plots were used solely for visualising parameter variability across studies and do not serve as diagnostic tools for MCMC convergence. All analyses were conducted in R (v4.0.2)^46^ using the following packages: ‘bayesmeta'^47^ for Bayesian random-effects meta-analysis and posterior inference; ‘ggplot2`^48^ and ‘ggridges'^49^ for data visualisation, including density and ridgeline plots; ‘dplyr'^50^ and ‘tidyr'^51^ for data preparation and wrangling; ‘readxl'^52^ for importing Excel-based datasets; and ‘mgcv'^53^ for supplementary generalised additive modelling.

### Role of the funding source

There was no funding source for this study.

## Results

### Sample characteristics

**Fig. 1:**
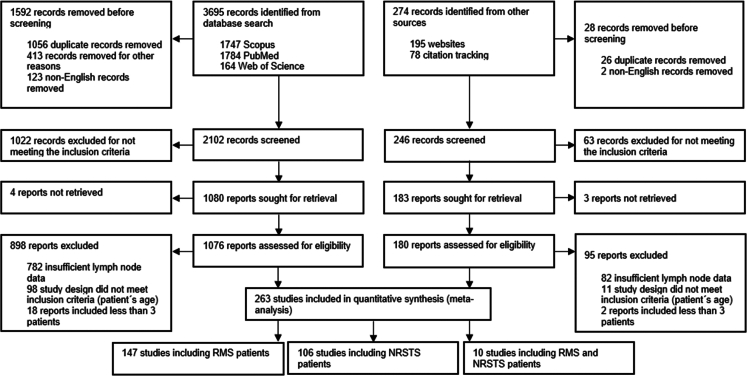
**PRISMA flow diagram of study selection.** This diagram illustrates the study selection process according to the PRISMA guidelines. RMS = rhabdomyosarcoma; NRSTS = non-rhabdomyosarcoma soft tissue sarcoma.

**Fig. 2:**
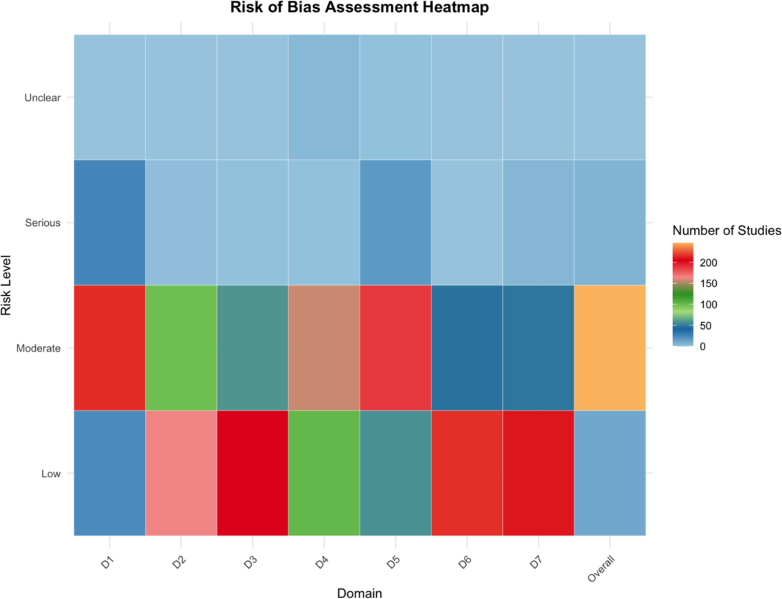
**Risk of bias assessments.** The figure displays the distribution of risk of bias ratings (low, moderate, serious, unclear) across individual domains (D1–D7) and overall for all included studies. Colour intensity corresponds to the number of studies, as indicated by the legend. D1 = confounding, D2 = selection of participants, D3 = classification of exposures, D4 = deviations from intended exposures, D5 = missing data, D6 = measurement of outcomes, D7 = selection of the reported result.

**Supplementary Fig. S1:**
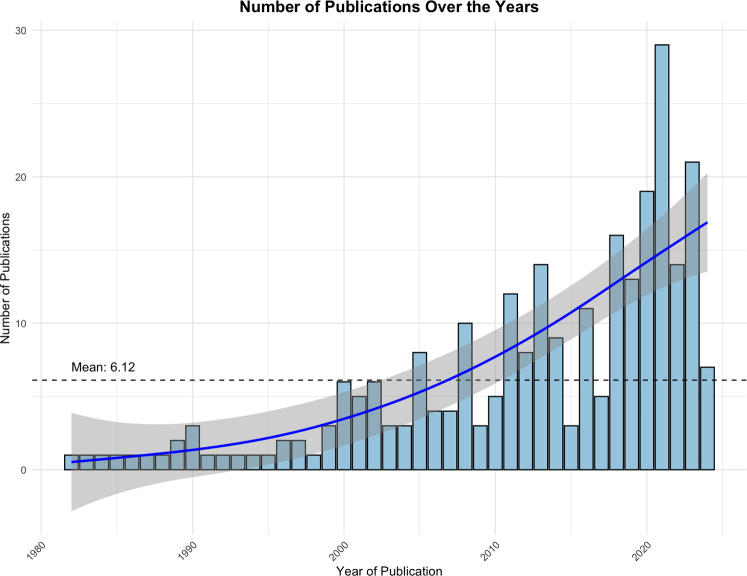


**Supplementary Fig. S2:**
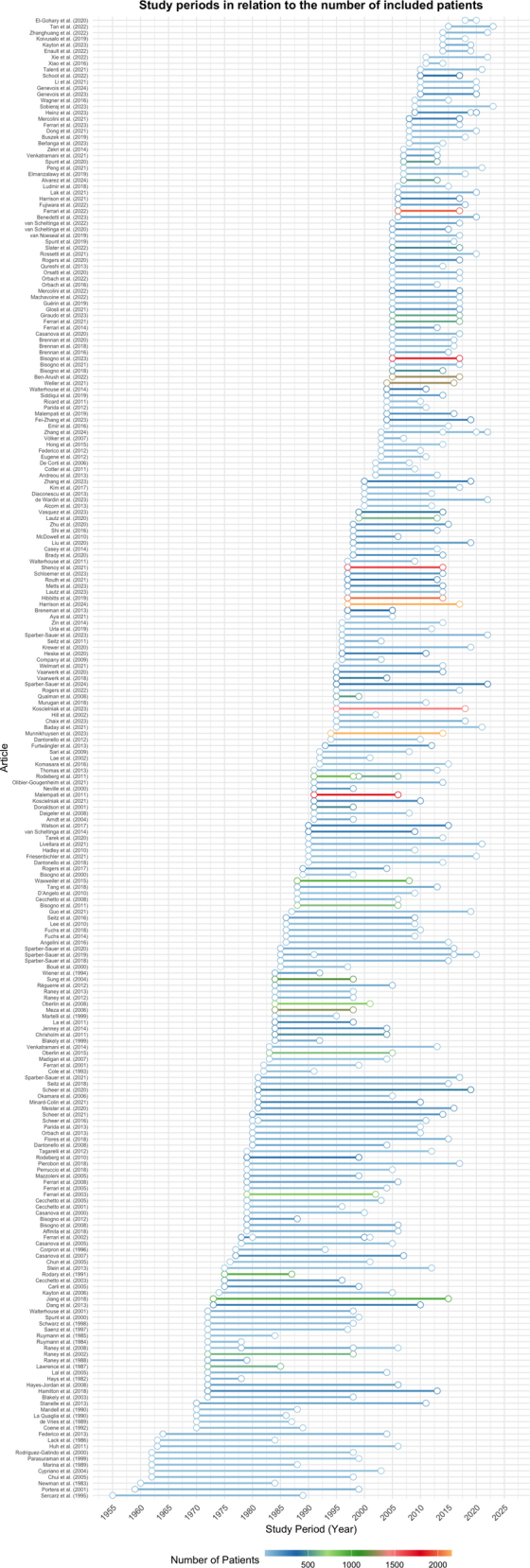


From a total of 3969 records initially screened, 263 articles (6.63%) were included in the final data synthesis (Fig. 1). An updated search was performed on June 1, 2025, which identified 17 additional eligible studies; however, these were not included in the current analysis owing to limited resources, time constraints, and the fact that the newly identified studies were small, heterogeneous, and unlikely to meaningfully impact the overall estimates. The included studies comprised 147 on RMS, 106 on NRSTS, and 10 on mixed RMS/NRSTS cohorts (Table 1), collectively encompassing 53,093 paediatric and AYA patients with STS. LN data were available for 52,883 individuals, including 9477 (17.92%) classified as N1, 39,871 (75.39%) as N0, and 3535 (6.69%) as Nx. Overall, 41,547 patients (78.25%) had RMS, (92.77%), while 11,546 patients (21.75%) had NRSTS. Within the included RMS studies, data on IRS postsurgical stage were available for 80.35% (n = 33,382) of the patients, of whom 73.31% (n = 30,458) were categorised as having IRS I–III, while 7.04% (n = 2924) were classified as IRS IV (metastatic disease). For the included patients with NRSTS, IRS data were available for 43.54% (n = 5025), of whom 39.19% (n = 4525) were classified as IRS I–III, and 4.33% (n = 500) as IRS IV. Among Patients with RMS, 16,929 (40.75%) had tumours <5 cm, and 18,151 (43.69%) had tumours >5 cm (data available for 84.43% of participants). Similarly, in the NRSTS population, 3505 (30.36%) had tumours <5 cm, and 4346 (37.64%) had tumours >5 cm (data available for 68% of participants). The assessment of LN involvement was reported to have been conducted clinically/radiologically in 89 articles (RMS: n = 67; NRSTS: n = 22) and/or surgically in 91 articles (RMS: n = 61; NRSTS: n = 30). A significant proportion of the included studies (n = 207, 78.71%) utilised (potentially) overlapping datasets, such as patient cohorts from the same CWS trials (CWS-86, CWS-91, CWS-2002P) or the SoTiSaR registry (Supplementary Table S3). As a result, there is a potential overlap in the data involving a total of 49,860 patients across these studies. The included studies were published between 1982 and 2024 with the highest concentration of publications occurring between 2000 and 2005 (Supplementary Fig. S1). The earliest study started in 1955 and the last study ended in 2022 (Supplementary Fig. S2). The mean duration of the individual study period was 14.36 ± 9.62 years, ranging from 1 year to 42 years. The overall mean sample size per study was 201 ± 377 (range 3–2157 patients). The detailed characteristics of the included studies are summarised in Table 1. The quality assessment of included studies showed that 11 (4.18%) studies had low risk of bias, 246 (93.54%) studies having moderate and 6 (2.28%) studies having serious risk of bias (Fig. 2 and Supplementary Table S4).

**Table 1:** Baseline characteristics of included studies.

	Overall		RMS		NRSTS		Mixed RMS/NRSTS cohort	
	n = 263	%	n = 147	%	n = 106	%	n = 10	%
**Region**								
Europa	126	47.91	70	47.62	50	47.17	6	60.00
America	109	41.44	67	45.58	38	35.85	4	40.00
Asia	26	9.89	10	6.80	16	15.09	0	0.00
Africa	2	0.76	0	0.00	2	1.89	0	0.00
**Data source**								
Register (International, National or regional database)^a^	168	63.88	113	76.87	54	50.94	1	10
Hospital electronic medical records	95	36.12	34	23.13	52	49.06	9	90
**Sample size**								
Mean, SD	201 ± 377		281 ± 458		105 ± 203		44 ± 58	
Range	3–2157		3–2157		4–1303		8–204	
3–10	25	9.51	13	8.84	11	10.38	1	10.00
11–50	96	36.50	41	27.89	48	45.28	7	70.00
51–100	45	17.11	20	13.61	24	22.64	1	10.00
>100	97	36.88	73	49.66	23	21.70	1	10.00
**Year of publication**								
Range	1982–2024		1982–2024		1986–2024		2006–2019	
<1990	9	3.42	6	4.08	3	2.83	0	0.00
1990–2010	73	27.76	40	27.21	29	27.36	4	40.00
2011–2020	110	41.83	62	42.18	42	39.62	6	60.00
>2020	71	27.00	39	26.53	32	30.19	0	0.00
**Tumour location**^b^								
Abdomen, Pelvis	48	6.09	10	2.16	38	12.14	0	0.00
Biliary tract, Liver	17	2.16	9	1.94	8	2.56	0	0.00
Body wall, Trunk	68	8.63	22	4.75	44	14.06	2	15.38
Extremity	139	17.64	66	14.25	67	21.41	6	46.15
Gastrointestinal tract	2	0.25	1	0.22	1	0.32	0	0.00
GU - Bladder-Prostate	59	7.49	57	12.31	2	0.64	0	0.00
GU - Non-BP	56	7.11	51	11.02	5	1.60	0	0.00
Head and neck, non-parameningeal	75	9.52	56	12.10	19	6.07	0	0.00
Head and neck, parameningeal	69	8.76	59	12.74	10	3.19	0	0.00
Head and neck, non-specified	50	6.35	11	2.38	38	12.14	1	7.69
Orbit	54	6.85	46	9.94	8	2.56	0	0.00
Paratesticular	19	2.41	17	3.67	2	0.64	0	0.00
Perineal, perianal	25	3.17	21	4.54	4	1.28	0	0.00
Retroperitoneal	30	3.81	16	3.46	14	4.47	0	0.00
Thorax	34	4.31	12	2.59	22	7.03	0	0.00
Visceral	28	3.55	1	0.22	27	8.63	1	7.69
Not specified	15	1.90	8	1.73	4	1.28	3	23.08
**RMS histology**^b^								
Alveolar	116	33.43	113	33.14	–		3	23.08
Botryoid	36	10.37	36	10.56	–		0	0.00
Embryonal	123	35.45	120	35.19	–		3	23.08
Leiomyomatous/spindle cell	29	8.36	29	8.50	–		0	0.00
Mixed-type	10	2.88	10	2.93	–		0	0.00
Pleomorphic	9	2.59	9	2.64	–		0	0.00
Undifferentiated sarcoma	11	3.17	11	3.23	–		0	0.00
Not specified	13	3.75	13	3.81	–		7	53.85
**NRSTS histology**^b^								
Adult-type fibrosarcoma	10	3.86	–		7	3.40	3	5.66
Alveolar soft part sarcoma	21	8.11	–		18	8.74	3	5.66
Angiomatoid fibrous histiocytoma	3	1.16	–		2	0.97	1	1.89
Angiosarcoma	9	3.47	–		8	3.88	1	1.89
Chondrosarcoma	3	1.16	–		2	0.97	1	1.89
Clear cell sarcoma	21	8.11	–		17	8.25	4	7.55
Dermatofibrosarcoma protuberans	5	1.93	–		4	1.94	1	1.89
Desmoplastic small round cell tumour	9	3.47	–		8	3.88	1	1.89
Embryonal sarcoma of the liver	3	1.16	–		3	1.46	0	0.00
Epithelioid hemangiothelioma	2	0.77	–		2	0.97	0	0.00
Epithelioid sarcoma	20	7.72	–		13	6.31	7	13.21
Ewing sarcoma	18	6.95	–		11	5.34	7	13.21
Fibromyxoid sarcoma	3	1.16	–		2	0.97	1	1.89
Fibrosarcoma	3	1.16	–		2	0.97	1	1.89
Hemangiopericytoma	5	1.93	–		4	1.94	1	1.89
High-grade sarcoma	1	0.39	–		0	0.00	1	1.89
Infantile fibrosarcoma	5	1.93	–		4	1.94	1	1.89
Inflammatory myofibroblastic tumour	3	1.16	–		3	1.46	0	0.00
Leiomyosarcoma	13	5.02	–		12	5.83	1	1.89
Liposarcoma	11	4.25	–		9	4.37	2	3.77
Malignant fibrous histiocytoma	8	3.09	–		7	3.40	1	1.89
Malignant glomus tumour	1	0.39	–		0	0.00	1	1.89
Malignant rhabdoid tumour	8	3.09	–		8	3.88	0	0.00
Malignant sarcomatoid tumour	1	0.39	–		1	0.49	0	0.00
MPNST	18	6.95	–		15	7.28	3	5.66
Myofibroblastic sarcoma	1	0.39	–		1	0.49	0	0.00
Primitive myxoid mesenchymal tumour of infancy	1	0.39	–		1	0.49	0	0.00
Primitive peripheral neuroectodermic tumour	8	3.09	–		7	3.40	1	1.89
Synovial sarcoma	27	10.42	–		20	9.71	7	13.21
Undifferentiated sarcoma	18	6.95	–		15	7.28	3	5.66
**IRS group**								
I–III	68	25.86	49	33.33	19	17.92	0	0.00
IV	2	0.76	2	1.36	0	0.00	0	0.00
I–IV	94	35.74	55	37.41	38	35.85	1	10.00
Not specified	99	37.64	41	27.89	49	46.23	9	90.00
**M Stadium**								
M0	15	5.70	10	6.80	4	3.77	1	10.00
M1	13	4.94	10	6.80	3	2.83	0	0.00
M0 and M1 (mixed)	124	47.15	45	30.61	76	71.70	3	30.00
Not specified	111	42.21	82	55.78	23	21.70	6	60.00
**Age at the time of the diagnosis**								
Infant	2	0.76	2	1.36	0	0.00	0	0.00
Pediatric (up until)	97	36.88	54	36.73	40	37.74	3	30.00
Young adult (up until)	57	21.67	27	18.37	26	24.53	4	40.00
Mixed cohort	46	17.49	17	11.56	27	25.47	2	20.00
Not specified	61	23.19	47	31.97	13	12.26	1	10.00
**Lymph node location**^b^								
Sentinel	12	3.38	4	1.93	2	1.59	6	27.27
Regional	98	27.61	66	31.88	24	19.05	8	36.36
Distant	80	22.54	56	27.05	18	14.29	6	27.27
Not specified	165	46.48	81	39.13	82	65.08	2	9.09
**Lymph node sampling**								
Random sampling	86	32.70	62	42.18	22	20.75	2	20.00
Both (SLNB and random)	12	4.56	4	2.72	2	1.89	6	60.00
Not specified	165	62.74	81	55.10	82	77.36	2	20.00
**Potentially overlapping cohorts**								
Yes	207	78.71	128	87.07	76	71.70	3	30.00
No	56	21.29	19	12.93	30	28.30	7	70.00

The table summarises the main characteristics of the 263 studies included in the meta-analysis, stratified by study population: rhabdomyosarcoma (RMS), non-rhabdomyosarcoma soft tissue sarcoma (NRSTS), and mixed cohorts. Percentages refer to the proportion within each subgroup unless otherwise indicated. Multiple entries per study were possible in some categories (e.g. tumour location, histology).

RMS = rhabdomyosarcoma; NRSTS = non-rhabdomyosarcoma soft tissue sarcoma; GU = genitourinary; BP = bladder-prostate; MPNST = malignant peripheral nerve sheath tumour; DSRCT = desmoplastic small round cell tumour; SLNB = sentinel lymph node biopsy; IRS = Intergroup Rhabdomyosarcoma Study.

a Detailed data on register studies is provided in Supplementary Table S3.

b Multiple entries per study possible, percentages refer to total mentions.

### LNM in patients with RMS

The overall prevalence of LNM across 41,547 patients with RMS, irrespective of tumour location or histological subtype, was estimated at a posterior mean proportion of 0.228 (95% credible interval: 0.202–0.255), indicating that 22.8% of patients with RMS are likely to have LNM, with a 95% probability that the true prevalence lies between 20.2% and 25.5%. The posterior mean of the between-study standard deviation (tau) was 0.860 (95% credible interval: 0.744–0.981), indicating moderate-to-high heterogeneity between studies in their true effect sizes. Sensitivity analyses, adjusted for studies with serious risk of bias (posterior mean proportion: 0.225; 95% CrI: 0.199–0.252) and possible overlapping cohorts (posterior mean proportion: 0.208; 95% CrI: 0.174–0.247), confirmed the robustness of the LNM prevalence estimates (Supplementary Table S5).

### LN involvement in RMS by tumour location

**Fig. 3:**
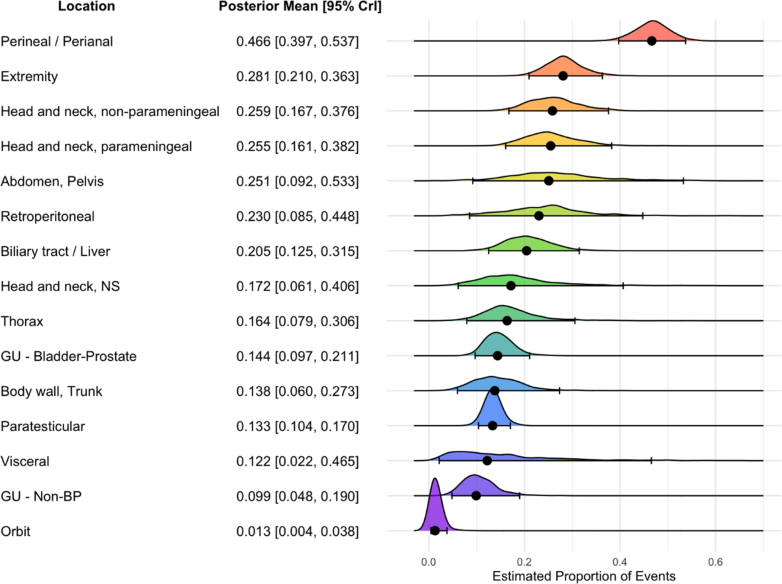
**Pooled LNM prevalences in patients with RMS, by tumour location.** Each row shows the posterior mean proportion of lymph node metastases (LNM) with 95% credible intervals (CrI), derived from Bayesian meta-analysis using logit-transformed proportions. Black dots represent the posterior mean; horizontal lines indicate the 95% CrI. The density curves illustrate the posterior distributions. RMS = rhabdomyosarcoma; LNM = lymph node metastases; CrI = credible interval; GU = genitourinary; NS = not specified; BP = bladder-prostate.

A subgroup meta-analysis for patients with RMS, stratified by tumour location, revealed substantial variability in reported LNM rates both within individual study data for specific tumour locations and in the pooled estimates across different tumour sites. The individual study level data on each specific RMS location is presented in Supplementary Fig. S3. The pooled prevalence of LNM was highest in patients with perineal/perianal RMS (n = 12 studies; n = 298 patients; posterior mean proportion: 0.466; 95% CrI: 0.397–0.537), followed by extremity RMS (n = 23 studies; n = 1984 patients; posterior mean proportion 0.281, 95% CrI: 0.210–0.363), non-parameningeal head and neck RMS (n = 10 studies; n = 858 patients; posterior mean proportion: 0.259; 95% CrI: 0.167–0.376), and parameningeal head and neck RMS (n = 11 studies; n = 1920 patients; posterior mean proportion: 0.255; 95% CrI: 0.161–0.382). Tumours located in the genitourinary tract excluding bladder and prostate (n = 10 studies; n = 816 patients; posterior mean proportion: 0.099; 95% CrI: 0.048–0.190) or the orbit (n = 7 studies; n = 552 patients; posterior mean proportion: 0.013; 95% CrI: 0.004–0.038) exhibited the lowest pooled prevalence of LNM (Fig. 3). Sensitivity analyses did not alter the pooled prevalence estimates for each RMS tumour site (Supplementary Fig. S4).

### LN involvement in RMS by histological subtype

Subgroup analysis by histological RMS subtype showed that patients with alveolar RMS (n = 26 studies; n = 1565 patients; posterior mean proportion: 0.370; 95% CrI: 0.276–0.473) had the highest pooled LNM prevalence, followed by the mixed-type (n = 3 studies; n = 13 patients; posterior mean proportion: 0.299; 95% CrI: 0.075–0.688), and undifferentiated RMS (n = 4 studies; n = 52 patients; posterior mean proportion: 0.282; 95% CrI: 0.103–0.560). Patients with embryonal RMS (n = 34 studies; n = 4418 patients; posterior mean proportion: 0.164; 95% CrI: 0.124–0.215) and Spindle cell (Leiomyomatous) histology (n = 4 studies; n = 32 patients; posterior mean proportion: 0.128; 95% CrI: 0.034–0.378) had the lowest LNM prevalences (Supplementary Fig. S5). These estimates remained stable after the sensitivity analysis (Supplementary Fig. S6).

### LNM in patients with NRSTS

Among patients with NRSTS (n = 11,546), the Bayesian pooled prevalence of LNM was estimated with a posterior mean of 0.111 (95% CrI: 0.092–0.133). The between-study standard deviation (tau) was 0.936 (95% CrI: 0.767–1.113), indicating substantial heterogeneity in LNM rates across NRSTS studies. After adjusting for studies with serious risk of bias (posterior mean: 0.112; 95% CrI: 0.093–0.135) and for studies with potentially overlapping cohorts (posterior mean: 0.109; 95% CrI: 0.077–0.152), we confirmed the stability of our findings (Supplementary Table S5).

### LN involvement in NRSTS by tumour location

**Supplementary Fig. S7:**
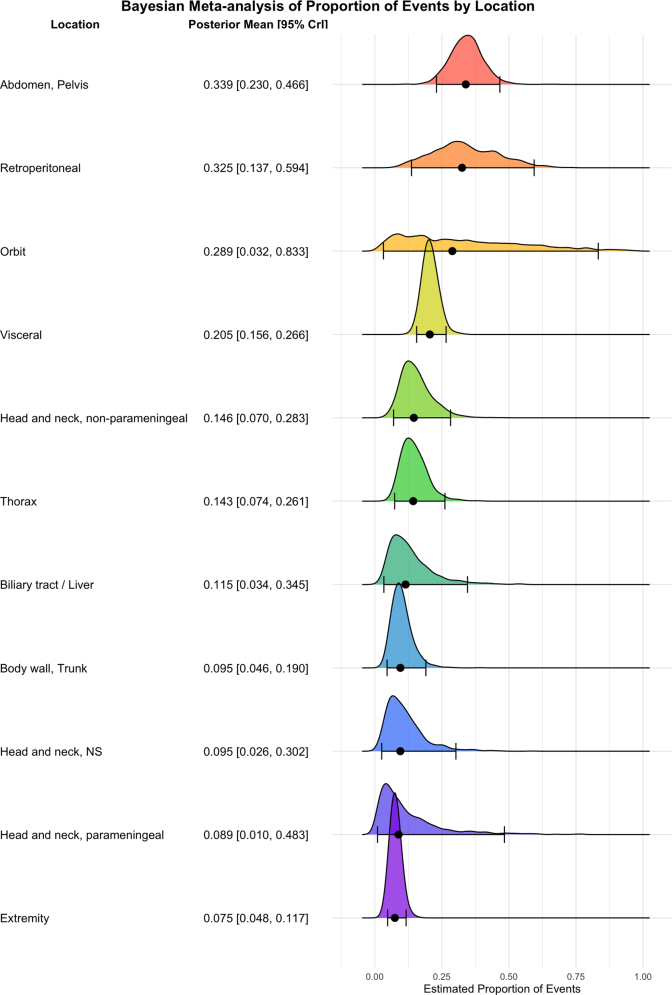


A sub-meta-analysis of patients with NRSTS stratified by anatomical region showed that tumours located in the abdomen/pelvis (n = 18 studies; n = 158 patients; posterior mean proportion: 0.339; 95% CrI 0.230–0.466) and retroperitoneum (n = 10 studies; n = 18 patients; posterior mean proportion: 0.325; 95% CrI: 0.137–0.594) had the highest pooled prevalence of LNM. In contrast, NRSTS located in the extremities (n = 30 studies; n = 977 patients; posterior mean proportion: 0.075; 95% CrI: 0.048–0.117) exhibited the lowest prevalence of LNM (Supplementary Fig. S7). The individual study-level data regarding anatomical region of involvement in NRSTS is provided in Supplementary Fig. S8. Sensitivity analyses did not alter the pooled prevalence estimates for each NRSTS tumour (Supplementary Fig. S9).

### LN involvement in NRSTS by histological subtype

**Fig. 4:**
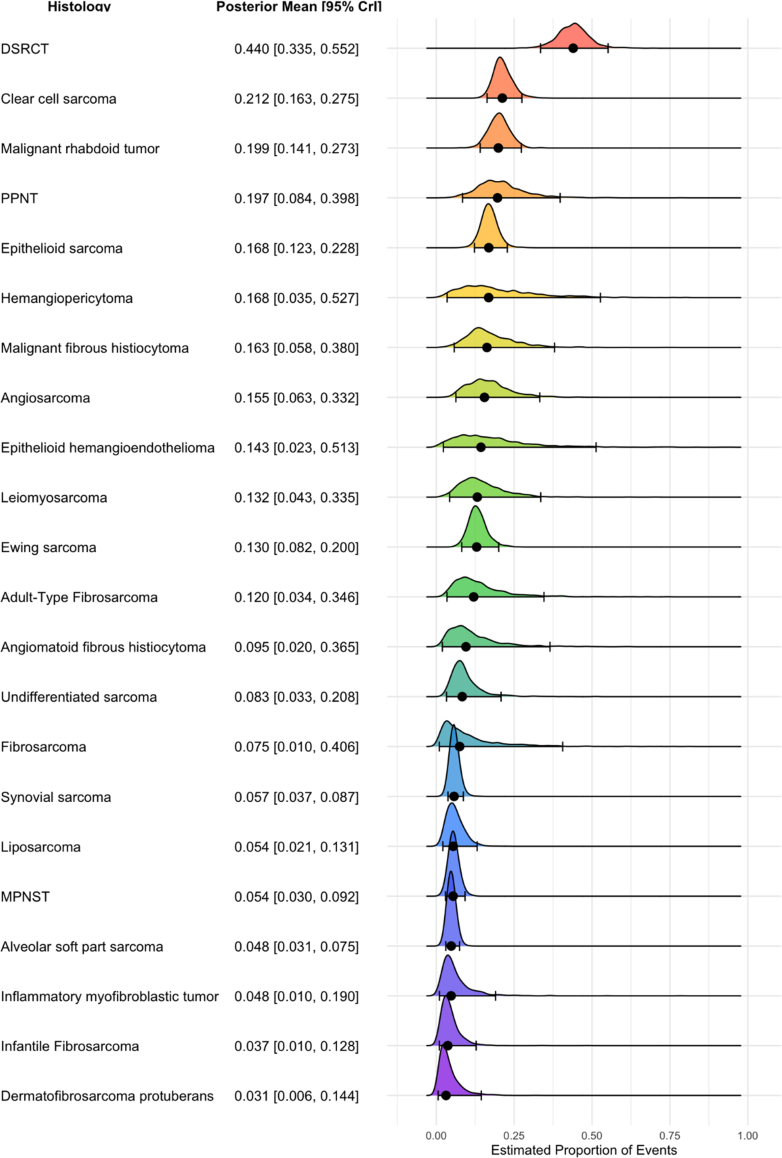
**Pooled LNM prevalences in patients with NRSTS, by tumour histology.** Each row shows the posterior mean proportion of lymph node metastases (LNM) with 95% credible intervals (CrI), derived from Bayesian meta-analysis using logit-transformed proportions. Black dots represent the posterior mean; horizontal lines indicate the 95% CrI. The density curves illustrate the posterior distributions. NRSTS = non-rhabdomyosarcoma soft tissue sarcoma; DSRCT = desmoplastic small round cell tumour; PPNT = peripheral primitive neuroectodermal tumour; MPNST = malignant peripheral nerve sheath tumour; CrI = credible interval.

If analysing pooled LNM proportions in patients with NRSTS stratified by tumour histology (individual study-level data for each histosubtype are provided in Supplementary Fig. S10), patients with DSRCT (n = 9 studies; n = 285 patients; posterior mean proportion: 0.440; 95% CrI: 0.335–0.552) showed the highest rate of LNM, followed by clear cell sarcoma (n = 16 studies; n = 638 patients; posterior mean proportion: 0.212; 95% CrI: 0.163–0.275), and malignant rhabdoid tumour (n = 8 studies; n = 253 patients; posterior mean proportion: 0.199; 95% CrI: 0.141–0.273), whereas NRSTS like infantile fibrosarcoma (n = 4 studies; n = 138 patients; posterior mean proportion: 0.037; 95% CrI: 0.010–0.128) and dermatofibrosarcoma protuberans (n = 3 studies; n = 92 patients; posterior mean proportion: 0.031; 95% CrI: 0.006–0.144), showed the lowest prevalences of LN involvement (Fig. 4). Sensitivity analyses did not significantly alter the pooled prevalence estimates for each NRSTS tumour histology (Supplementary Fig. S11). Density plots and trace plots for both RMS and NRSTS are presented in Supplementary Fig. S12.

## Discussion

In this Bayesian proportional meta-analysis of 263 studies and 53,093 patients, we synthesised the prevalence of LNM across different STS types occurring in children/AYA worldwide. Patients with RMS had an overall higher pooled posterior mean proportion of LNM (22.8%) compared with patients with NRSTS (11.1%). This is not surprising, as RMS and NRSTS have different patterns of metastatic spread. RMS is described for having a higher propensity for lymphatic spread compared with NRSTS. In NRSTS, localised disease is more common, and in metastatic disease, hematogenous dissemination seems to occur more frequently.^31^^,^^54^

In our analysis, LNM prevalence estimates varied severely by anatomical tumour locations and histological subtypes within RMS and patients with NRSTS. While the highest proportions of LNM were observed in perineal/perianal RMS (46.6%), extremity RMS (28%), and non-parameningeal head and neck RMS (25.8%), the same anatomical locations in patients with NRSTS had notably lower proportions of LNM, including 7.5% in extremity and 14.5% in non-parameningeal head and neck sites. By contrast, NRSTS located in the abdomen/pelvis (33.9%) or retroperitoneum (32.5%) had the highest LNM prevalence rates. In general, the primary site, *per se*, is a known prognostic factor in RMS.^11^^,^^12^ Favorable sites (e.g., orbit, paratesticular) are associated with better oncological outcomes as they are typically more accessible for surgery or radiation and often present earlier than unfavorable sites (e.g., parameningeal head and neck, extremities, trunk), which frequently pose greater challenges for local control and tend to be detected later. In contrast to RMS, there is no universally accepted site-based categorisation for NRSTS, where prognosis and treatment strategies are guided by factors such as histologic subtype, tumour size (<5 cm *vs.* >5 cm), grade (low *vs.* high), completeness of resection (R0 *vs.* R1 *vs.* R2), and the presence of distant metastases.^55^ Therefore, the results of our study regarding LNM prevalences stratified by anatomical region should be interpreted in the context of established clinicopathologic variables such as the tumour’s histology (ARMS *vs.* ERMS),^56^ molecular biology (*PAX3/PAX7–FOXO1* fusion status),^5^^,^^8^^,^^57^ patient age (<10 years *vs.* >10 years),^58^^,^^59^ and tumour size (<5 cm *vs.* >5 cm)^59^ for RMS patients and histologic subtype^31^ or tumour invasiveness^60^ for patients with NRSTS. Moreover, studies reporting higher prevalences of LNM among both RMS and patients with NRSTS tended to include more advanced disease populations, characterised in the RMS group by alveolar histology,^24^ *PAX3/PAX7–FOXO1* fusion positivity,^61^, ^62^, ^63^ older patients,^64^, ^65^, ^66^ and larger tumours,^15^^,^^28^^,^^29^^,^^67^, ^68^, ^69^ and in the NRSTS group by specific histologic subtypes (e.g., DSRCT) and more invasive tumours.^31^ We further demonstrated a substantial variation in LNM prevalence rates among different RMS and NRSTS histotypes. The highest LNM proportions were observed among patients with RMS and alveolar (36.9%), mixed-type (29.9%), and undifferentiated (28.2%) histologies. Notably, the high prevalence of lymph node metastases (LNM) in patients with ARMS is frequently associated with concurrent unfavorable clinical factors, including age over 10 years, primary tumours at unfavorable sites, advanced stage and group disease, and large tumours.^70^ In contrast, Michael A. et al. observed a 0% LNM rate in children with ARMS who exhibited low-risk clinical features,^71^ the observed LNM rates should be interpreted within this context.

In the NRSTS group, the highest rates of LNM were observed in patients with DSRCT (43.9%), clear cell sarcoma (20.3%), and malignant rhabdoid tumour (19.9%). A possible explanation for the high prevalence of LNM in DSRCT (reported rates of LN involvement in individual studies range from 40% to 60%^34^, ^35^, ^36^, ^37^, ^38^, ^39^, ^40^), a highly aggressive soft tissue neoplasm, is that patients tend to present at advanced stages, with 50% of cases showing metastatic disease at the time of diagnosis.^72^ The patient cohorts with DSRCT in our dataset demonstrated a high prevalence of regionally disseminated or metastatic disease, ranging from 21% to 90%.^35^, ^36^, ^37^, ^38^^,^^40^^,^^73^, ^74^, ^75^ Therefore, the observed rates of LNM in DSRCT should be interpreted within this metastatic context. In contrast to DSRCT, most patients with clear cell sarcoma had either non-metastatic localised disease^76^^,^^77^ or predominantly localised disease, with metastatic rates ranging from 7% to 15%,^78^, ^79^, ^80^, ^81^ although one study reported a higher rate of 42% in stage IV cases.^82^ Thus, the high rates of LNM are possibly attributed to the tumour's biology, which exhibits a high propensity for lymphatic spread.^83^^,^^84^ Malignant rhabdoid tumours are highly aggressive neoplasms originating in the kidney, non-renal soft tissues, and the central nervous system. The high prevalence of LNM in MRTs (range from data from individual studies 6–28%^85^, ^86^, ^87^, ^88^, ^89^, ^90^, ^91^, ^92^) may be attributed to the tumours' intrinsic biology, characterised by high invasiveness with a propensity for lymphatic dissemination, as well as patient characteristics within the analysed studies, where 43–85% of patients were reported to be in stage IV.^86^^,^^89^, ^90^, ^91^ Additionally, Brennan et al. demonstrated that the proportion of N1 patients was significantly lower in those with localised disease (11.6%) compared with patients with metastatic disease (47.8%).^87^

Although not yet widely used in clinical research, we chose a Bayesian statistical framework (conception of probability as a degree of belief^93^) over traditional frequentist paradigm (P[data|hypothesis]; expected frequency of observed data under many hypothetical repetitions)^94^ for our study design. This choice was driven by anticipated high data heterogeneity and limited sample sizes across paediatric STS studies, guided by prior knowledge (*a priori* beliefs) from pre-existing literature and expert opinions. The interpretation of our prevalence data is straightforward, providing full probability distributions for LNM along with credible intervals that offer a clear view of the likelihood of true prevalence. This probabilistic interpretation aligns well with clinical realities, where uncertainty is inherent and decision-making often requires weighing different probabilities.

We are aware of several limitations in our study. First, because we relied on primary study-level data, we were unable to account for individual patient and tumour characteristics (e.g., fusion status in RMS) or detailed clinical outcomes. Although we were able to extract data on LN involvement, attributing it to specific patient subgroups was frequently limited, particularly with respect to distinguishing between LN involvement in locoregional (N1, M0) and metastatic (N0, M1 to distant LN or N1, M1) context. Thus, our ability to perform robust subgroup analyses was inherently restricted. Second, despite employing a robust search strategy that included bibliographic database searches and supplementary citation tracking using Scopus and Google Scholar, there remains the potential for missing relevant studies. Information regarding LNM is not consistently reported in titles or abstracts, increasing the risk of excluding studies that may contain pertinent data but were not retrieved during the search process. Therefore, we additionally applied text mining tools in PubMed to search information regarding LNM at the sentence level. Third, the timeframe of the included studies was extensive, ranging from early study periods starting in 1955^95^ (published1995), 1964^96^ (published 2015), or 1970^97^ (published 1992) to more contemporary series ending recruitment in 2018.^98^ Over this period, advances in diagnostic methods, such as the introduction of CT into clinical practice in the 1970s,^99^ MRI in the 1980s,^100^ and the more recent adoption of PET/CT and PET/MRI in the 2000s,^101^^,^^102^ may have influenced diagnostic accuracy and detection rates of LNM.

Finally, the diagnostic basis for LN involvement varied across the included studies and was often insufficiently reported. Most studies reported LN staging based on histologic and/or clinical/radiologic assessments,^25^^,^^29^^,^^56^ while others additionally incorporated FDG-PET/CT.^103^ However, in the majority of cases, the specific criteria used to define nodal involvement remained unclear. This variability in staging methodology may have contributed to the observed heterogeneity in LNM prevalence and limits the comparability of findings across studies. Our observations are in line with the recent consensus work by van Scheltinga et al.,^14^ which underscores the lack of standardisation in LN staging in paediatric STS. Such methodological heterogeneity remains a major challenge for meta-analyses and highlights the urgent need for harmonised diagnostic criteria in future research.

In conclusion, this is the first study to synthesise global estimates of both locoregional and distant LNM prevalence in paediatric and AYA patients with STS. Our pooled data indicate that LNM prevalence in both NRSTS and RMS varies substantially by tumour location and histological subtype. Finally, there is an urgent need to harmonise protocols and reporting standards for LN assessment in paediatric STS.

## Contributors

Authorship Confirmation/Contribution Statement: IM: conceptualisation, methodology, investigation, data curation, writing; LT: methodology, investigation, writing–review and editing; AS: methodology, writing–review and editing; AM: methodology, writing–review and editing; ATH: writing–review and editing; MSS: writing–review and editing; ME: writing–review and editing; JF: writing–review and editing; RMV: writing–review and editing; GS: supervision, writing–review and editing. IM and LT accessed and verified the underlying data.

## Data sharing statement

All datasets generated and analysed during the current study, along with the complete R code used for data processing and analysis, are available in the Supplementary materials.

## Declaration of interests

We declare no competing interests.

## Appendix A Supplementary data

LN Codes

LN Final Table

Supplementary Table S5

Trends RMS NRSTS

NRSTS Histology Trends

NRSTS Histology

NRSTS Location Trend

NRSTS Location

NRSTS all

RMS Histology

RMS Sensitivity

RMS Trend Location

RMS all

RoB Heatmap

Study period

Supplementary Fig. S3

RMS Location

Trend RMS Histology
