# Supplementary material for: Prevalence of locoregional and distant lymph node metastases in children and adolescents/young adults with soft tissue sarcomas: a Bayesian meta-analysis of proportions
Source: eClinicalMedicine. 2025 Aug 7;87:103390. doi: 10.1016/j.eclinm.2025.103390 (PMC12355419; doi:10.1016/j.eclinm.2025.103390)
Supplement: LN Codes [file mmc1.pdf]

R-Codes - LN Metaanalysis Martynov-Tóbi

Number of publications over the years	2
Overlapping studies	3
Trends over the years	4
Estimation of study periods	5
Risk of bias heatmap	6
Bayesian all studies	7
Between study variability	9
Sensitivity data	10
Trace Plot small analysis	12
Bayesian Meta small analysis	14
Sensitivity Analysis small analysis	16
Sensitivity analysis plot all studies	18
Sensitivity analysis small studies	20
Combined Bayesian small analysis with data	23
Bayesian separated analysis small studies	26
Bayesian full analysis	29
Posterior mean proportions	32
Between study variability	34
Density plot small studies	36
Trends 1.	38
Trends 2.	41

Number of publications over the years

```
# Load necessary libraries
library(ggplot2)
library(readxl)
library(mgcv)  # Library for fitting generalized additive models

# Load the data
data <- read_excel("Desktop/PubD.xlsx")

# Extract the relevant columns
years <- data$Year
number_of_publications <- data$`Number of Publications`

# Create a data frame
df <- data.frame(Year = years, Publications = number_of_publications)

# Calculate the mean number of publications
mean_publications <- mean(df$Publications, na.rm = TRUE)

# Define a Bayesian color palette
bayesian_colors <- c("#a6cee3", "#1f78b4", "#b2df8a", "#33a02c", "#fb9a99", "#e31a1c", "#fdbf6f")

# Plot the data
ggplot(df, aes(x = Year, y = Publications)) +
  geom_bar(stat = "identity", fill = bayesian_colors[1], color = "black") +  # Use Bayesian color
  geom_hline(yintercept = mean_publications, linetype = "dashed", color = "red") +  # Add mean line
  geom_smooth(method = "gam", formula = y ~ s(x, bs = "cs"), color = "blue", se = TRUE) +  # Add non-
linear trend line with confidence interval
  labs(title = "Number of Publications Over the Years",
       x = "Year",
       y = "Number of Publications") +
  annotate("text", x = min(df$Year), y = mean_publications, label = paste("Mean:",
round(mean_publications, 2)),
           vjust = -1, hjust = 0, color = "red") +  # Annotate mean value
  theme_minimal()
```

Overlapping studies

```
# Load necessary libraries
library(readxl)
library(dplyr)

# Load the data
data <- read_excel("Desktop/Study period.xlsx")
colnames(data) <- c("Study", "Start", "End", "Number.of.Patients")

# Convert Start and End to Date format
data <- data %>%
  mutate(Start = as.Date(paste0(Start, "-01-01")),
         End = as.Date(paste0(End, "-12-31")))

# Define 5-year intervals from 1955 to 2025
time_bins <- seq(as.Date("1955-01-01"), as.Date("2025-12-31"), by = "5 years")

# Create a data frame to store the number of overlapping studies per interval
overlap_counts <- data.frame(
  Interval_Start = time_bins[-length(time_bins)],
  Interval_End = time_bins[-1],
  Overlap_Count = integer(length(time_bins) - 1)
)

# Count overlapping studies for each interval
for (i in 1:nrow(overlap_counts)) {
  overlap_counts$Overlap_Count[i] <- sum(
    data$Start <= overlap_counts$Interval_End[i] &
    data$End >= overlap_counts$Interval_Start[i]
  )
}

# Find the interval with the highest concentration of studies
max_overlap <- overlap_counts[which.max(overlap_counts$Overlap_Count), ]

# Display the interval and number of studies
print(paste("The 5-year interval with the highest concentration of studies is from",
          format(max_overlap$Interval_Start, "%Y"), "to", format(max_overlap$Interval_End, "%Y")))
print(paste("Number of studies conducted during this period:", max_overlap$Overlap_Count))
```

Trends over the years

```
# Load necessary libraries
library(ggplot2)
library(dplyr)
library(bayesmeta)

# Load your data from the specified path
sensitivity_data <- read.csv("~/Desktop/LN Trends.csv", sep = ";")

# Check for required columns
if (!all(c("Sarcoma.subtype", "Total", "Event", "Study.start", "Study.end") %in%
colnames(sensitivity_data))) {
  stop("Data is missing required columns: 'Sarcoma.subtype', 'Total', 'Event', 'Study.start', or 'Study.end'.")
}

# Define pseudocount to avoid zero counts
pseudocount <- 0.5

# Calculate log-odds and study period midpoint
sensitivity_data <- sensitivity_data %>%
  mutate(
    midpoint_year = (as.numeric(Study.start) + as.numeric(Study.end)) / 2,
    log_odds = log((Event + pseudocount) / (Total - Event + pseudocount)),
    variance = 1 / (Event + pseudocount) + 1 / (Total - Event + pseudocount)
  )

# Separate RMS and non-RMS STS subtypes
sensitivity_data <- sensitivity_data %>%
  mutate(subtype_group = ifelse(Sarcoma.subtype == "RMS", "RMS", "non-RMS STS"))

# Generate color palette for subtypes
unique_subtypes <- unique(sensitivity_data$Sarcoma.subtype)
color_gradient <- colorRampPalette(c("#FF6347", "#FFD700", "#32CD32", "#1E90FF", "#8A2BE2"))
bayesian_gradient_colors <- color_gradient(length(unique_subtypes))
color_mapping <- setNames(bayesian_gradient_colors, unique_subtypes)

# Plot with trend lines and 95% confidence intervals
ggplot(sensitivity_data, aes(x = midpoint_year, y = log_odds, color = Sarcoma.subtype)) +
  geom_point(size = 2) +
  geom_errorbar(aes(ymin = log_odds - 1.96 * sqrt(variance), ymax = log_odds + 1.96 * sqrt(variance)),
width = 0.2) +
  stat_smooth(method = "lm", formula = y ~ poly(x, 1), se = TRUE, level = 0.95, aes(color =
Sarcoma.subtype), linetype = "solid") +
  scale_color_manual(values = color_mapping) +
  labs(title = "Trends of LN Positivity for RMS and non-RMS STS Over Time",
       x = "Publication Year",
       y = "Log-Odds of LN Positivity") +
  theme_minimal() +
  theme(legend.position = "top") +
  guides(color Study = guide_legend(title = "Sarcoma Subtype"))
```

Estimation of study periods

```
# Load necessary libraries
library(ggplot2)
library(dplyr)

# Load the data (adjusted for your file path and format)
Study.period <- read.csv("~/Desktop/Study period.csv", sep = ";")
colnames(Study.period) <- c("Article", "Start", "End", "Number.of.Patients")

# Convert Start and End to Date format for accurate plotting on the x-axis
Study.period <- Study.period %>%
  mutate(Start = as.Date(paste0(Start, "-01-01")),
         End = as.Date(paste0(End, "-12-31")))

# Reorder articles based on Start date for logical plotting
Study.period <- Study.period %>%
  arrange(Start) %>%
  mutate(Article = factor(Article, levels = unique(Article)))

# Define a Bayesian-inspired color gradient
bayesian_palette <- colorRampPalette(c("#a6cee3", "#1f78b4", "#b2df8a", "#33a02c", "#fb9a99",
"#e31a1c", "#fdbf6f"))

# Plot with consistent line width and color gradient to represent patient counts
plot <- ggplot(Study.period, aes(y = Article, xmin = Start, xmax = End, color = Number.of.Patients)) +
  geom_errorbarh(aes(xmin = Start, xmax = End), height = 0.4, linewidth = 1) +  # Set line width
  geom_point(aes(x = Start), size = 3, shape = 21, fill = "white") +
  geom_point(aes(x = End), size = 3, shape = 21, fill = "white") +
  scale_color_gradientn(colors = bayesian_palette(100)) +  # Apply Bayesian color gradient
  labs(title = "Study periods in relation to the number of included patients",
       x = "Study Period (Year)",
       y = "Article",
       color = "Number of Patients") +
  scale_x_date(
    limits = as.Date(c("1955-01-01", "2025-12-31")),
    breaks = seq(as.Date("1955-01-01"), as.Date("2025-12-31"), by = "5 years"),  # Set 5-year intervals
    date_labels = "%Y"
  ) +
  theme_minimal(base_size = 12) +
  theme(
    axis.text.x = element_text(angle = 45, hjust = 1),
    axis.text.y = element_text(size = 6),  # Further reduce y-axis text size
    plot.margin = margin(1, 1, 1, 1, "cm"),  # Adjust plot margins
    legend.position = "bottom",
    plot.title = element_text(hjust = 0.5, face = "bold", size = 14)  # Center and bold the title
  ) +
  guides(color = guide_colorbar(barwidth = 15, barheight = 0.5))  # Wider color legend

# Display the plot in R
print(plot)
```

Risk of bias heatmap

```
# Load necessary libraries
library(ggplot2)
library(dplyr)
library(tidyr)

# Load the data
RoB.Heatmap <- read.csv("~/Desktop/RoB Heatmap.csv", header = FALSE, sep = ";")

# Assign column names (assuming the first column is "Risk")
colnames(RoB.Heatmap) <- c("Risk", paste0("D", 1:(ncol(RoB.Heatmap) - 2)), "Overall")

# Ensure the Risk column contains the correct categories and is factorized in the right order
RoB.Heatmap$Risk <- factor(RoB.Heatmap$Risk, levels = c("Low", "Moderate", "Serious", "Unclear"))

# Reshape the data to long format
RoB.Heatmap_long <- RoB.Heatmap %>%
  pivot_longer(cols = -Risk, names_to = "Domain", values_to = "Count")

# Convert Count to numeric and handle non-numeric values
RoB.Heatmap_long$Count <- suppressWarnings(as.numeric(as.character(RoB.Heatmap_long$Count)))

# Define Bayesian-inspired color gradient
bayesian_palette <- colorRampPalette(c("#a6cee3", "#1f78b4", "#b2df8a", "#33a02c", "#fb9a99",
"#e31a1c", "#fdbf6f"))

# Plot the heatmap
ggplot(RoB.Heatmap_long, aes(x = Domain, y = Risk, fill = Count)) +
  geom_tile(color = "white") +
  scale_fill_gradientn(colors = bayesian_palette(100), name = "Number of Studies", na.value = "grey") +
  labs(title = "Risk of Bias Assessment Heatmap",
       x = "Domain",
       y = "Risk Level") +
  theme_minimal() +
  theme(
    axis.text.x = element_text(angle = 45, hjust = 1),
    plot.title = element_text(hjust = 0.5, face = "bold")
  )
```

Bayesian all studies

```
# Load necessary libraries
library(dplyr)
library(bayesmeta)
library(ggplot2)
library(ggridges)
library(scales)  # For color gradient functions

# Load the data
sensitivity_data <- read.csv("~/Desktop/RMS all.csv", sep = ";")

# Define function to run Bayesian meta-analysis and extract posterior samples
run_analysis_with_posterior <- function(data_subset) {
  pseudocount <- 0.5
  data_subset <- data_subset %>%
    mutate(
      adjusted_proportion = (Events + pseudocount) / (Total + 2 * pseudocount),
      adjusted_variance = (adjusted_proportion * (1 - adjusted_proportion)) / Total,
      logit_proportion = log(adjusted_proportion / (1 - adjusted_proportion)),
      logit_variance = adjusted_variance / (adjusted_proportion * (1 - adjusted_proportion))^2
    ) %>%
    filter(is.finite(logit_proportion) & is.finite(logit_variance) & logit_variance > 0)

  # Run Bayesian meta-analysis
  result <- bayesmeta(y = data_subset$logit_proportion,
                      sigma = sqrt(data_subset$logit_variance),
                      tau.prior = function(x) dhalfnormal(x, scale = 1))

  # Extract posterior samples for visualization
  posterior_samples <- data.frame(mu = result$rposterior(n = 1000)[, "mu"])

  # Extract summary statistics
  mean_logit <- result$summary[3, 2]
  ci_lower_logit <- result$summary[5, 2]
  ci_upper_logit <- result$summary[6, 2]

  mean_proportion <- exp(mean_logit) / (1 + exp(mean_logit))
  ci_lower_proportion <- exp(ci_lower_logit) / (1 + exp(ci_lower_logit))
  ci_upper_proportion <- exp(ci_upper_logit) / (1 + exp(ci_upper_logit))

  list(posterior_samples = posterior_samples,
       mean_proportion = mean_proportion,
       ci_lower_proportion = ci_lower_proportion,
       ci_upper_proportion = ci_upper_proportion)
}

# Run the analysis for each Article and store results
posterior_samples_list <- list()
summary_data <- data.frame()

for (article in unique(sensitivity_data$Article)) {
  subset_data <- sensitivity_data %>% filter(Article == article)
  analysis_result <- run_analysis_with_posterior(subset_data)

  # Store posterior samples with Article info
  analysis_result$posterior_samples$Article <- article
  posterior_samples_list[[article]] <- analysis_result$posterior_samples

  # Store summary data for point estimates and CIs
```

```
    summary_data <- rbind(summary_data,
                  data.frame(Article = article,
                             Mean = analysis_result$mean_proportion,
                             CI_Lower = analysis_result$ci_lower_proportion,
                             CI_Upper = analysis_result$ci_upper_proportion))
}

# Combine all posterior samples into one data frame
posterior_df <- do.call(rbind, posterior_samples_list)

# Order Article factor levels by Mean proportion for plotting
summary_data <- summary_data %>%
  arrange(Mean) # Arrange by mean proportion
summary_data$Article <- factor(summary_data$Article, levels = summary_data$Article)

# Apply the same ordering to the posterior samples data frame
posterior_df$Article <- factor(posterior_df$Article, levels = levels(summary_data$Article))

# Generate a gradient color palette that spans the number of unique articles
color_gradient <- colorRampPalette(c("#FF6347", "#FFD700", "#32CD32", "#1E90FF", "#8A2BE2"))  #
Customize start/end colors
bayesian_gradient_colors <- color_gradient(length(unique(summary_data$Article)))

# Create the ridgeline plot with ordered y-axis and gradient color transition
ggplot() +
    geom_density_ridges(data = posterior_df, aes(x = exp(mu) / (1 + exp(mu)), y = Article, fill = Article),
                 alpha = 0.7, scale = 1.5) +
    geom_point(data = summary_data, aes(x = Mean, y = Article), color = "black", size = 3) +
    geom_errorbarh(data = summary_data, aes(xmin = CI_Lower, xmax = CI_Upper, y = Article),
              height = 0.2, color = "black") +
    scale_fill_manual(values = bayesian_gradient_colors) +
    labs(title = "Bayesian Meta-analysis of Proportion of Events by Article",
        x = "Estimated Proportion of Events",
        y = "Article") +
    theme_minimal() +
    theme(legend.position = "none",
         plot.title = element_text(hjust = 0.5),
         plot.margin = margin(1, 1, 1, 1, "cm"))
```

Between study variability

```
# Load necessary libraries
library(dplyr)
library(bayesmeta)

# Load the data
sensitivity_data <- read.csv("~/Desktop/RMS all.csv", sep = ";")

# Define function to run Bayesian meta-analysis
run_analysis <- function(data_subset) {
  pseudocount <- 0.5
  data_subset <- data_subset %>%
    mutate(
      adjusted_proportion = (Events + pseudocount) / (Total + 2 * pseudocount),
      adjusted_variance = (adjusted_proportion * (1 - adjusted_proportion)) / Total,
      logit_proportion = log(adjusted_proportion / (1 - adjusted_proportion)),
      logit_variance = adjusted_variance / (adjusted_proportion * (1 - adjusted_proportion))^2
    ) %>%
    filter(is.finite(logit_proportion) & is.finite(logit_variance) & logit_variance > 0)

  # Run Bayesian meta-analysis
  bayesmeta(y = data_subset$logit_proportion,
        sigma = sqrt(data_subset$logit_variance),
        tau.prior = function(x) dhalfnormal(x, scale = 1))
}

# Check if the data contains sufficient information for analysis
if (nrow(sensitivity_data) > 0) {
  # Run the analysis
  result <- run_analysis(sensitivity_data)

  # Check if the analysis result is available
  if (!is.null(result)) {
    # Extract the posterior mean and 95% credible interval for τ (between-study standard deviation)
    tau_mean <- result$summary[3, "tau"]  # Posterior mean of τ
    tau_ci_lower <- result$summary[5, "tau"]  # Lower bound of 95% CI for τ
    tau_ci_upper <- result$summary[6, "tau"]  # Upper bound of 95% CI for τ

    # Display the results
    cat("Posterior Mean of Between-Study Standard Deviation (τ):", round(tau_mean, 4), "\n")
    cat("95% Credible Interval for τ:", round(tau_ci_lower, 4), "-", round(tau_ci_upper, 4), "\n")
  } else {
    message("Meta-analysis could not be completed.")
  }
} else {
  message("Insufficient data for meta-analysis.")
}
```

Sensitivity data

```
# Load necessary libraries
library(dplyr)
library(bayesmeta)
library(ggplot2)

# Load the sensitivity data
sensitivity_data <- read.csv("~/Desktop/RMS Sensitivity.csv", sep = ";")

# Check for required columns
if (!all(c("Author", "Total", "Events") %in% colnames(sensitivity_data))) {
  stop("Data is missing required columns: 'Author', 'Total', or 'Events'.")
}

# Define function to run Bayesian meta-analysis and extract summary statistics
run_analysis_summary <- function(data_subset) {
  pseudocount <- 0.5
  data_subset <- data_subset %>%
    mutate(
      adjusted_proportion = (Events + pseudocount) / (Total + 2 * pseudocount),
      adjusted_variance = (adjusted_proportion * (1 - adjusted_proportion)) / Total,
      logit_proportion = log(adjusted_proportion / (1 - adjusted_proportion)),
      logit_variance = adjusted_variance / (adjusted_proportion * (1 - adjusted_proportion))^2
    ) %>%
    filter(is.finite(logit_proportion) & is.finite(logit_variance) & logit_variance > 0)

  # Run Bayesian meta-analysis
  result <- bayesmeta(y = data_subset$logit_proportion,
                      sigma = sqrt(data_subset$logit_variance),
                      tau.prior = function(x) dhalfnormal(x, scale = 1))

  # Extract summary statistics
  mean_logit <- result$summary[3, 2]
  ci_lower_logit <- result$summary[5, 2]
  ci_upper_logit <- result$summary[6, 2]

  mean_proportion <- exp(mean_logit) / (1 + exp(mean_logit))
  ci_lower_proportion <- exp(ci_lower_logit) / (1 + exp(ci_lower_logit))
  ci_upper_proportion <- exp(ci_upper_logit) / (1 + exp(ci_upper_logit))

  list(mean_proportion = mean_proportion,
       ci_lower_proportion = ci_lower_proportion,
       ci_upper_proportion = ci_upper_proportion)
}

# Perform the main sensitivity analyses on all data
# Main Analysis (All Studies)
main_results <- run_analysis_summary(sensitivity_data)

# Overlap Exclusion (exclude certain cohorts)
overlap_excluded_data <- sensitivity_data %>% filter(!grepl("A|B|C", Cohorts))
overlap_results <- if (nrow(overlap_excluded_data) > 1) run_analysis_summary(overlap_excluded_data)
else NULL

# Bias Exclusion (exclude high-risk bias studies)
bias_excluded_data <- sensitivity_data %>% filter(Serious.Risk.of.Bias == "no")
bias_results <- if (nrow(bias_excluded_data) > 1) run_analysis_summary(bias_excluded_data) else NULL
```

```
# Combined Exclusion (exclude both overlap and high-risk bias studies)
combined_excluded_data <- sensitivity_data %>% filter(!grepl("A|B|C", Cohorts) & Serious.Risk.of.Bias ==
"no")
combined_results <- if (nrow(combined_excluded_data) > 1)
run_analysis_summary(combined_excluded_data) else NULL

# Compile results into a data frame for plotting
sensitivity_results <- data.frame(
  Scenario = c("Main Analysis (All Studies)", "Overlap Exclusion", "Bias Exclusion", "Combined
Exclusion"),
  Mean = c(main_results$mean_proportion,
         if (!is.null(overlap_results)) overlap_results$mean_proportion else NA,
         if (!is.null(bias_results)) bias_results$mean_proportion else NA,
         if (!is.null(combined_results)) combined_results$mean_proportion else NA),
  CI_Lower = c(main_results$ci_lower_proportion,
             if (!is.null(overlap_results)) overlap_results$ci_lower_proportion else NA,
             if (!is.null(bias_results)) bias_results$ci_lower_proportion else NA,
             if (!is.null(combined_results)) combined_results$ci_lower_proportion else NA),
  CI_Upper = c(main_results$ci_upper_proportion,
             if (!is.null(overlap_results)) overlap_results$ci_upper_proportion else NA,
             if (!is.null(bias_results)) bias_results$ci_upper_proportion else NA,
             if (!is.null(combined_results)) combined_results$ci_upper_proportion else NA)
)

# Print the data for review
print(sensitivity_results)

# Set the order of scenarios for the y-axis
sensitivity_results$Scenario <- factor(sensitivity_results$Scenario,
                                       levels = c("Main Analysis (All Studies)", "Bias Exclusion", "Overlap Exclusion",
"Combined Exclusion"))

# Generate a color palette for the scenarios
scenario_colors <- c("blue", "darkorange", "green", "purple")

# Plot sensitivity analysis for the combined data
ggplot(sensitivity_results, aes(x = Mean, y = Scenario)) +
  geom_point(aes(color = Scenario), size = 3) +
  geom_errorbarh(aes(xmin = CI_Lower, xmax = CI_Upper, color = Scenario), height = 0.2) +
  scale_color_manual(values = scenario_colors) +
  labs(title = "Combined Sensitivity Analysis for All Data",
       x = "Posterior Mean Proportion (95% CI)",
       y = "Analysis Scenario") +
  theme_minimal() +
  theme(legend.position = "none")  # Hide legend for clarity
```

Trace Plot small analysis

```
# Load necessary libraries
library(bayesmeta)
library(ggplot2)
library(dplyr)
library(tidyr)
library(scales)  # For dynamic color generation

# Load your data from the specified path
sensitivity_data <- read.csv("~/Desktop/Sensitivity-RMS-Local.csv", sep = ";")

# Check for necessary columns
if (!all(c("Localization", "Total", "Events") %in% colnames(sensitivity_data))) {
  stop("Data is missing required columns: 'Localization', 'Total', or 'Events'.")
}

# Extract unique localizations
localizations <- unique(sensitivity_data$Localization)

# Define a pseudocount to avoid issues with proportions of 0 or 1
pseudocount <- 0.5

# Run Bayesian meta-analysis for each localization
results_list <- list()

for (loc in localizations) {
  # Filter data for the specific localization
  subset_data <- sensitivity_data %>% filter(Localization == loc)

  # Adjust data for Bayesian meta-analysis
  subset_data <- subset_data %>%
    mutate(
      adjusted_proportion = (Events + pseudocount) / (Total + 2 * pseudocount),
      adjusted_variance = (adjusted_proportion * (1 - adjusted_proportion)) / Total,
      logit_proportion = log(adjusted_proportion / (1 - adjusted_proportion)),
      logit_variance = adjusted_variance / (adjusted_proportion * (1 - adjusted_proportion))^2
    ) %>%
    filter(is.finite(logit_proportion) & is.finite(logit_variance) & logit_variance > 0)

  # Run Bayesian meta-analysis
  result <- bayesmeta(
    y = subset_data$logit_proportion,
    sigma = sqrt(subset_data$logit_variance),
    tau.prior = function(x) dhalfnormal(x, scale = 1)
  )

  # Store posterior samples of effect sizes (mu) and heterogeneity (tau)
  posterior_samples <- result$rposterior(n = 1000)
  results_list[[loc]] <- data.frame(
    Location = loc,  # Changed to "Location"
    mu = posterior_samples[, "mu"],
    tau = posterior_samples[, "tau"]
  )
}

# Combine all results into one data frame
trace_data <- bind_rows(results_list)
```

```
# Calculate density for tau values
tau_density <- density(trace_data$tau, na.rm = TRUE)

# Convert density to a data frame for plotting
tau_density_df <- data.frame(tau = tau_density$x, density = tau_density$y)

# Generate a color palette with enough colors for all localizations
color_palette <- hue_pal()(length(localizations))

# Plot the trace plot for each location with tau on the x-axis and mu on the y-axis
ggplot(trace_data, aes(x = tau, y = mu, color = Location, group = Location)) +
  geom_line() +
  geom_area(data = tau_density_df, aes(x = tau, y = density * max(trace_data$mu) * 0.5),
          inherit.aes = FALSE, fill = "gray", alpha = 0.3) +
  labs(x = "Heterogeneity (τ)", y = "Effect Size (μ)") +
  theme_minimal() +
  theme(legend.position = "right") +
  scale_color_manual(values = color_palette) +
  ggtitle("Trace Plot of Effect Size (μ) vs. Heterogeneity (τ) by Location") +  # Updated title
  theme(plot.title = element_text(hjust = 0.5))
```

Bayesian Meta small analysis

```
# Load necessary libraries
library(dplyr)
library(bayesmeta)
library(ggplot2)
library(ggridges)
library(scales)  # For color gradient functions

# Load the sensitivity data
sensitivity_data <- read.csv("~/Desktop/Sensitivity-RMS-Local.csv", sep = ";")

# Define function to run Bayesian meta-analysis and extract posterior samples
run_analysis_with_posterior <- function(data_subset) {
  pseudocount <- 0.5
  data_subset <- data_subset %>%
    mutate(
      adjusted_proportion = (Events + pseudocount) / (Total + 2 * pseudocount),
      adjusted_variance = (adjusted_proportion * (1 - adjusted_proportion)) / Total,
      logit_proportion = log(adjusted_proportion / (1 - adjusted_proportion)),
      logit_variance = adjusted_variance / (adjusted_proportion * (1 - adjusted_proportion))^2
    ) %>%
    filter(is.finite(logit_proportion) & is.finite(logit_variance) & logit_variance > 0)

  # Run Bayesian meta-analysis
  result <- bayesmeta(y = data_subset$logit_proportion,
                      sigma = sqrt(data_subset$logit_variance),
                      tau.prior = function(x) dhalfnormal(x, scale = 1))

  # Extract posterior samples for visualization
  posterior_samples <- data.frame(mu = result$rposterior(n = 1000)[, "mu"])

  # Extract summary statistics
  mean_logit <- result$summary[3, 2]
  ci_lower_logit <- result$summary[5, 2]
  ci_upper_logit <- result$summary[6, 2]

  mean_proportion <- exp(mean_logit) / (1 + exp(mean_logit))
  ci_lower_proportion <- exp(ci_lower_logit) / (1 + exp(ci_lower_logit))
  ci_upper_proportion <- exp(ci_upper_logit) / (1 + exp(ci_upper_logit))

  list(posterior_samples = posterior_samples,
       mean_proportion = mean_proportion,
       ci_lower_proportion = ci_lower_proportion,
       ci_upper_proportion = ci_upper_proportion)
}

# Run the analysis for each Location and store results
posterior_samples_list <- list()
summary_data <- data.frame()

for (loc in unique(sensitivity_data$Localization)) {
  subset_data <- sensitivity_data %>% filter(Localization == loc)
  analysis_result <- run_analysis_with_posterior(subset_data)

  # Store posterior samples with Localization info
  analysis_result$posterior_samples$Localization <- loc
  posterior_samples_list[[loc]] <- analysis_result$posterior_samples
```

```
    # Store summary data for point estimates and CIs
    summary_data <- rbind(summary_data,
                          data.frame(Localization = loc,
                                     Mean = analysis_result$mean_proportion,
                                     CI_Lower = analysis_result$ci_lower_proportion,
                                     CI_Upper = analysis_result$ci_upper_proportion))
}

# Combine all posterior samples into one data frame
posterior_df <- do.call(rbind, posterior_samples_list)

# Order Localization factor levels by Mean proportion for plotting
summary_data <- summary_data %>%
  arrange(Mean) # Arrange by mean proportion
summary_data$Localization <- factor(summary_data$Localization, levels = summary_data$Localization)

# Apply the same ordering to the posterior samples data frame
posterior_df$Localization <- factor(posterior_df$Localization, levels = levels(summary_data$Localization))

# Generate a gradient color palette that spans the number of unique locations
color_gradient <- colorRampPalette(c("#FF6347", "#FFD700", "#32CD32", "#1E90FF", "#8A2BE2"))  #
Customize start/end colors
bayesian_gradient_colors <- color_gradient(length(unique(summary_data$Localization)))

# Create the ridgeline plot with ordered y-axis and gradient color transition
ggplot() +
    geom_density_ridges(data = posterior_df, aes(x = exp(mu) / (1 + exp(mu)), y = Localization, fill =
Localization),
                        alpha = 0.7, scale = 1.5) +
    geom_point(data = summary_data, aes(x = Mean, y = Localization), color = "black", size = 3) +
    geom_errorbarh(data = summary_data, aes(xmin = CI_Lower, xmax = CI_Upper, y = Localization),
                   height = 0.2, color = "black") +
    scale_fill_manual(values = bayesian_gradient_colors) +
    labs(title = "Bayesian Meta-analysis of Proportion of Events by Location",
         x = "Estimated Proportion of Events",
         y = "Location") +
    theme_minimal() +
    theme(legend.position = "none",
          plot.title = element_text(hjust = 0.5),
          plot.margin = margin(1, 1, 1, 1, "cm"))
```

Sensitivity Analysis small analysis

```
# Load necessary libraries
library(dplyr)
library(bayesmeta)

# Load the sensitivity data from your specified path
sensitivity_data <- read.csv("~/Desktop/Sensitivity-nonRMS Local.csv", sep = ";")

# Define a function to run Bayesian meta-analysis and extract summary statistics
run_analysis_summary <- function(data_subset) {
  pseudocount <- 0.5
  data_subset <- data_subset %>%
    mutate(
      adjusted_proportion = (Events + pseudocount) / (Total + 2 * pseudocount),
      adjusted_variance = (adjusted_proportion * (1 - adjusted_proportion)) / Total,
      logit_proportion = log(adjusted_proportion / (1 - adjusted_proportion)),
      logit_variance = adjusted_variance / (adjusted_proportion * (1 - adjusted_proportion))^2
    ) %>%
    filter(is.finite(logit_proportion) & is.finite(logit_variance) & logit_variance > 0)

  # Run Bayesian meta-analysis
  result <- bayesmeta(y = data_subset$logit_proportion,
                      sigma = sqrt(data_subset$logit_variance),
                      tau.prior = function(x) dhalfnormal(x, scale = 1))

  # Extract summary statistics
  mean_logit <- result$summary[3, 2]
  ci_lower_logit <- result$summary[5, 2]
  ci_upper_logit <- result$summary[6, 2]

  mean_proportion <- exp(mean_logit) / (1 + exp(mean_logit))
  ci_lower_proportion <- exp(ci_lower_logit) / (1 + exp(ci_lower_logit))
  ci_upper_proportion <- exp(ci_upper_logit) / (1 + exp(ci_upper_logit))

  tau_mean <- result$summary[3, "tau"]
  tau_ci_lower <- result$summary[5, "tau"]
  tau_ci_upper <- result$summary[6, "tau"]

  list(
    mean_proportion = mean_proportion,
    ci_lower_proportion = ci_lower_proportion,
    ci_upper_proportion = ci_upper_proportion,
    tau_mean = tau_mean,
    tau_ci_lower = tau_ci_lower,
    tau_ci_upper = tau_ci_upper
  )
}

# Define a function to run all scenarios for each histology
run_sensitivity_analysis <- function(histology_data) {
  # Main Analysis (All Studies)
  main_results <- run_analysis_summary(histology_data)

  # Overlap Exclusion (exclude studies with certain cohorts)
  overlap_excluded_data <- histology_data %>% filter(!grepl("A|B|C", Cohorts)) # Adjust cohort pattern as
needed
  overlap_results <- if (nrow(overlap_excluded_data) > 1) run_analysis_summary(overlap_excluded_data)
else NULL
```

```
  # Bias Exclusion (exclude high-risk bias studies)
  bias_excluded_data <- histology_data %>% filter(Serious.Risk.of.Bias == "no")
  bias_results <- if (nrow(bias_excluded_data) > 1) run_analysis_summary(bias_excluded_data) else NULL

  # Combined Exclusion (exclude both overlap and high-risk bias studies)
  combined_excluded_data <- histology_data %>% filter(!grepl("A|B|C", Cohorts) & Serious.Risk.of.Bias
== "no")
  combined_results <- if (nrow(combined_excluded_data) > 1)
run_analysis_summary(combined_excluded_data) else NULL

  # Compile results into a table for the histology
  data.frame(
    Scenario = c("Main Analysis (All Studies)", "Overlap Exclusion", "Bias Exclusion", "Combined
Exclusion"),
    Posterior_Mean_Proportion = c(main_results$mean_proportion,
                                  if (!is.null(overlap_results)) overlap_results$mean_proportion else NA,
                                  if (!is.null(bias_results)) bias_results$mean_proportion else NA,
                                  if (!is.null(combined_results)) combined_results$mean_proportion else NA),
    `95% CI (Proportion)` = c(
      paste0("[", round(main_results$ci_lower_proportion, 2), ", ",
round(main_results$ci_upper_proportion, 2), "]"),
      if (!is.null(overlap_results)) paste0("[", round(overlap_results$ci_lower_proportion, 2), ", ",
round(overlap_results$ci_upper_proportion, 2), "]") else NA,
      if (!is.null(bias_results)) paste0("[", round(bias_results$ci_lower_proportion, 2), ", ",
round(bias_results$ci_upper_proportion, 2), "]") else NA,
      if (!is.null(combined_results)) paste0("[", round(combined_results$ci_lower_proportion, 2), ", ",
round(combined_results$ci_upper_proportion, 2), "]") else NA
    ),
    Posterior_Mean_Tau = c(main_results$tau_mean,
                           if (!is.null(overlap_results)) overlap_results$tau_mean else NA,
                           if (!is.null(bias_results)) bias_results$tau_mean else NA,
                           if (!is.null(combined_results)) combined_results$tau_mean else NA),
    `95% CI (Tau)` = c(
      paste0("[", round(main_results$tau_ci_lower, 2), ", ", round(main_results$tau_ci_upper, 2), "]"),
      if (!is.null(overlap_results)) paste0("[", round(overlap_results$tau_ci_lower, 2), ", ",
round(overlap_results$tau_ci_upper, 2), "]") else NA,
      if (!is.null(bias_results)) paste0("[", round(bias_results$tau_ci_lower, 2), ", ",
round(bias_results$tau_ci_upper, 2), "]") else NA,
      if (!is.null(combined_results)) paste0("[", round(combined_results$tau_ci_lower, 2), ", ",
round(combined_results$tau_ci_upper, 2), "]") else NA
    )
  )
}

# Initialize an empty list to store tables for each histology
sensitivity_tables <- list()

# Run the sensitivity analysis for each histology
for (histology in unique(sensitivity_data$Histology)) {
  histology_data <- sensitivity_data %>% filter(Histology == histology)
  sensitivity_tables[[histology]] <- run_sensitivity_analysis(histology_data)
}

# Print each histology's sensitivity table
for (histology in names(sensitivity_tables)) {
  cat("\nSensitivity Analysis for Histology:", histology, "\n")
  print(sensitivity_tables[[histology]])
  cat("\n---------------------------------------\n")
}
```

Sensitivity analysis plot all studies

```
# Load necessary libraries
library(dplyr)
library(bayesmeta)
library(ggplot2)

# Load the sensitivity data
sensitivity_data <- read.csv("~/Desktop/Sensitivity RMS.csv", sep = ";")

# Check for required columns
if (!all(c("Author", "Total", "Events") %in% colnames(sensitivity_data))) {
  stop("Data is missing required columns: 'Author', 'Total', or 'Events'.")
}

# Define function to run Bayesian meta-analysis and extract summary statistics
run_analysis_summary <- function(data_subset) {
  pseudocount <- 0.5
  data_subset <- data_subset %>%
    mutate(
      adjusted_proportion = (Events + pseudocount) / (Total + 2 * pseudocount),
      adjusted_variance = (adjusted_proportion * (1 - adjusted_proportion)) / Total,
      logit_proportion = log(adjusted_proportion / (1 - adjusted_proportion)),
      logit_variance = adjusted_variance / (adjusted_proportion * (1 - adjusted_proportion))^2
    ) %>%
    filter(is.finite(logit_proportion) & is.finite(logit_variance) & logit_variance > 0)

  # Run Bayesian meta-analysis
  result <- bayesmeta(y = data_subset$logit_proportion,
                      sigma = sqrt(data_subset$logit_variance),
                      tau.prior = function(x) dhalfnormal(x, scale = 1))

  # Extract summary statistics
  mean_logit <- result$summary[3, 2]
  ci_lower_logit <- result$summary[5, 2]
  ci_upper_logit <- result$summary[6, 2]

  mean_proportion <- exp(mean_logit) / (1 + exp(mean_logit))
  ci_lower_proportion <- exp(ci_lower_logit) / (1 + exp(ci_lower_logit))
  ci_upper_proportion <- exp(ci_upper_logit) / (1 + exp(ci_upper_logit))

  list(mean_proportion = mean_proportion,
       ci_lower_proportion = ci_lower_proportion,
       ci_upper_proportion = ci_upper_proportion)
}

# Perform the main sensitivity analyses on all data
# Main Analysis (All Studies)
main_results <- run_analysis_summary(sensitivity_data)

# Overlap Exclusion (exclude certain cohorts)
overlap_excluded_data <- sensitivity_data %>% filter(!grepl("A|B|C", Cohorts))
overlap_results <- if (nrow(overlap_excluded_data) > 1) run_analysis_summary(overlap_excluded_data)
else NULL

# Bias Exclusion (exclude high-risk bias studies)
bias_excluded_data <- sensitivity_data %>% filter(Serious.Risk.of.Bias == "no")
bias_results <- if (nrow(bias_excluded_data) > 1) run_analysis_summary(bias_excluded_data) else NULL
```

```
# Combined Exclusion (exclude both overlap and high-risk bias studies)
combined_excluded_data <- sensitivity_data %>% filter(!grepl("A|B|C", Cohorts) & Serious.Risk.of.Bias ==
"no")
combined_results <- if (nrow(combined_excluded_data) > 1)
run_analysis_summary(combined_excluded_data) else NULL

# Compile results into a data frame for plotting
sensitivity_results <- data.frame(
  Scenario = c("Main Analysis (All Studies)", "Overlap Exclusion", "Bias Exclusion", "Combined
Exclusion"),
  Mean = c(main_results$mean_proportion,
         if (!is.null(overlap_results)) overlap_results$mean_proportion else NA,
         if (!is.null(bias_results)) bias_results$mean_proportion else NA,
         if (!is.null(combined_results)) combined_results$mean_proportion else NA),
  CI_Lower = c(main_results$ci_lower_proportion,
             if (!is.null(overlap_results)) overlap_results$ci_lower_proportion else NA,
             if (!is.null(bias_results)) bias_results$ci_lower_proportion else NA,
             if (!is.null(combined_results)) combined_results$ci_lower_proportion else NA),
  CI_Upper = c(main_results$ci_upper_proportion,
             if (!is.null(overlap_results)) overlap_results$ci_upper_proportion else NA,
             if (!is.null(bias_results)) bias_results$ci_upper_proportion else NA,
             if (!is.null(combined_results)) combined_results$ci_upper_proportion else NA)
)

# Set the order of scenarios for the y-axis
sensitivity_results$Scenario <- factor(sensitivity_results$Scenario,
                                  levels = c("Main Analysis (All Studies)", "Bias Exclusion", "Overlap Exclusion",
"Combined Exclusion"))

# Generate a color palette for the scenarios
scenario_colors <- c("blue", "darkorange", "green", "purple")

# Plot sensitivity analysis for the combined data
ggplot(sensitivity_results, aes(x = Mean, y = Scenario)) +
  geom_point(aes(color = Scenario), size = 3) +
  geom_errorbarh(aes(xmin = CI_Lower, xmax = CI_Upper, color = Scenario), height = 0.2) +
  scale_color_manual(values = scenario_colors) +
  labs(title = "Combined Sensitivity Analysis for All Data",
      x = "Posterior Mean Proportion (95% CI)",
      y = "Analysis Scenario") +
  theme_minimal() +
  theme(legend.position = "none")  # Hide legend for clarity
```

Sensitivity analysis small studies

```
# Load necessary libraries
library(bayesmeta)
library(ggplot2)
library(dplyr)

# Load your sensitivity data
sensitivity_data <- read.csv("~/Desktop/Sensitivity-RMS-Local.csv", sep = ";")

# Check for required columns
if (!all(c("Localization", "Total", "Events") %in% colnames(sensitivity_data))) {
  stop("Data is missing required columns: 'Localization', 'Total', or 'Events'.")
}

# Define function to run Bayesian meta-analysis and extract summary statistics
run_analysis_summary <- function(data_subset) {
  pseudocount <- 0.5
  data_subset <- data_subset %>%
    mutate(
      adjusted_proportion = (Events + pseudocount) / (Total + 2 * pseudocount),
      adjusted_variance = (adjusted_proportion * (1 - adjusted_proportion)) / Total,
      logit_proportion = log(adjusted_proportion / (1 - adjusted_proportion)),
      logit_variance = adjusted_variance / (adjusted_proportion * (1 - adjusted_proportion))^2
    ) %>%
    filter(is.finite(logit_proportion) & is.finite(logit_variance) & logit_variance > 0)

  # Run Bayesian meta-analysis
  result <- bayesmeta(y = data_subset$logit_proportion,
                      sigma = sqrt(data_subset$logit_variance),
                      tau.prior = function(x) dhalfnormal(x, scale = 1))

  # Extract summary statistics
  mean_logit <- result$summary[3, 2]
  ci_lower_logit <- result$summary[5, 2]
  ci_upper_logit <- result$summary[6, 2]

  mean_proportion <- exp(mean_logit) / (1 + exp(mean_logit))
  ci_lower_proportion <- exp(ci_lower_logit) / (1 + exp(ci_lower_logit))
  ci_upper_proportion <- exp(ci_upper_logit) / (1 + exp(ci_upper_logit))

  list(mean_proportion = mean_proportion,
       ci_lower_proportion = ci_lower_proportion,
       ci_upper_proportion = ci_upper_proportion)
}

# Initialize an empty data frame to store sensitivity analysis results
sensitivity_results <- data.frame()

# Run the sensitivity analysis for each localization
for (loc in unique(sensitivity_data$Localization)) {
  loc_data <- sensitivity_data %>% filter(Localization == loc)

  # Main Analysis
  main_results <- run_analysis_summary(loc_data)

  # Overlap Exclusion (exclude certain cohorts)
  overlap_excluded_data <- loc_data %>% filter(!grepl("A|B|C", Cohorts))
```

```
  overlap_results <- if (nrow(overlap_excluded_data) > 1) run_analysis_summary(overlap_excluded_data)
else NULL

  # Bias Exclusion (exclude high-risk bias studies)
  bias_excluded_data <- loc_data %>% filter(Serious.Risk.of.Bias == "no")
  bias_results <- if (nrow(bias_excluded_data) > 1) run_analysis_summary(bias_excluded_data) else NULL

  # Combined Exclusion (exclude both overlap and high-risk bias studies)
  combined_excluded_data <- loc_data %>% filter(!grepl("A|B|C", Cohorts) & Serious.Risk.of.Bias == "no")
  combined_results <- if (nrow(combined_excluded_data) > 1)
run_analysis_summary(combined_excluded_data) else NULL

  # Append results to sensitivity_results
  sensitivity_results <- rbind(sensitivity_results,
                         data.frame(
                           Location = loc,
                           Scenario = "Main Analysis (All Studies)",
                           Mean = main_results$mean_proportion,
                           CI_Lower = main_results$ci_lower_proportion,
                           CI_Upper = main_results$ci_upper_proportion
                         ),
                         if (!is.null(overlap_results)) data.frame(
                           Location = loc,
                           Scenario = "Overlap Exclusion",
                           Mean = overlap_results$mean_proportion,
                           CI_Lower = overlap_results$ci_lower_proportion,
                           CI_Upper = overlap_results$ci_upper_proportion
                         ),
                         if (!is.null(bias_results)) data.frame(
                           Location = loc,
                           Scenario = "Bias Exclusion",
                           Mean = bias_results$mean_proportion,
                           CI_Lower = bias_results$ci_lower_proportion,
                           CI_Upper = bias_results$ci_upper_proportion
                         ),
                         if (!is.null(combined_results)) data.frame(
                           Location = loc,
                           Scenario = "Combined Exclusion",
                           Mean = combined_results$mean_proportion,
                           CI_Lower = combined_results$ci_lower_proportion,
                           CI_Upper = combined_results$ci_upper_proportion
                         ))
}

# Set the order of scenarios for the y-axis
sensitivity_results$Scenario <- factor(sensitivity_results$Scenario,
                                levels = c("Main Analysis (All Studies)", "Bias Exclusion", "Overlap Exclusion",
"Combined Exclusion"))

# Generate a color palette for each unique Location
color_palette <- colorRampPalette(c("#FF6347", "#FFD700", "#32CD32", "#1E90FF", "#8A2BE2"))
location_colors <- setNames(color_palette(length(unique(sensitivity_results$Location))),
                      unique(sensitivity_results$Location))

# Plot sensitivity analysis for each Location with consistent colors in each mini-graph
ggplot(sensitivity_results, aes(x = Mean, y = Scenario)) +
  geom_point(aes(color = Location), size = 3) +
  geom_errorbarh(aes(xmin = CI_Lower, xmax = CI_Upper, color = Location), height = 0.2) +
  facet_wrap(~ Location, ncol = 2) +
  scale_color_manual(values = location_colors) +
```

```
labs(title = "Sensitivity Analysis by Location",
     x = "Posterior Mean Proportion (95% CI)",
     y = "Analysis Scenario") +
theme_minimal() +
theme(legend.position = "none")  # Hide legend for clarity
```

Combined Bayesian small analysis with data

```
# Load necessary libraries
library(dplyr)
library(bayesmeta)
library(ggplot2)
library(ggridges)
library(scales)
library(patchwork)

# Load the histology data
histology_data <- read.csv("~/Desktop/NRSTS Histology.csv", sep = ";")

# Replace long histology names with abbreviations
histology_data$Histology <- recode(histology_data$Histology,
                          "Desmoplastic small round cell tumor" = "DSRCT",
                          "Primitive peripheral neuroectodermic tumor" = "PPNT")

# Define function to run Bayesian meta-analysis and extract posterior samples
run_analysis_with_posterior <- function(data_subset) {
  pseudocount <- 0.5
  data_subset <- data_subset %>%
    mutate(
      adjusted_proportion = (Events + pseudocount) / (Total + 2 * pseudocount),
      adjusted_variance = (adjusted_proportion * (1 - adjusted_proportion)) / Total,
      logit_proportion = log(adjusted_proportion / (1 - adjusted_proportion)),
      logit_variance = adjusted_variance / (adjusted_proportion * (1 - adjusted_proportion))^2
    ) %>%
    filter(is.finite(logit_proportion) & is.finite(logit_variance) & logit_variance > 0)

  # Run Bayesian meta-analysis
  result <- bayesmeta(y = data_subset$logit_proportion,
                sigma = sqrt(data_subset$logit_variance),
                tau.prior = function(x) dhalfnormal(x, scale = 1))

  # Extract posterior samples for visualization
  posterior_samples <- data.frame(mu = result$rposterior(n = 1000)[, "mu"])

  # Extract summary statistics
  mean_logit <- result$summary[3, 2]
  ci_lower_logit <- result$summary[5, 2]
  ci_upper_logit <- result$summary[6, 2]

  mean_proportion <- exp(mean_logit) / (1 + exp(mean_logit))
  ci_lower_proportion <- exp(ci_lower_logit) / (1 + exp(ci_lower_logit))
  ci_upper_proportion <- exp(ci_upper_logit) / (1 + exp(ci_upper_logit))

  list(posterior_samples = posterior_samples,
     mean_proportion = mean_proportion,
     ci_lower_proportion = ci_lower_proportion,
     ci_upper_proportion = ci_upper_proportion)
}

# Run the analysis for each Histology type and store results
posterior_samples_list <- list()
summary_data <- data.frame()

for (hist in unique(histology_data$Histology)) {
  subset_data <- histology_data %>% filter(Histology == hist)
```

```
    analysis_result <- run_analysis_with_posterior(subset_data)

    # Store posterior samples with Histology info
    analysis_result$posterior_samples$Histology <- hist
    posterior_samples_list[[hist]] <- analysis_result$posterior_samples

    # Store summary data for point estimates and CIs
    summary_data <- rbind(summary_data,
                          data.frame(Histology = hist,
                                     Mean = analysis_result$mean_proportion,
                                     CI_Lower = analysis_result$ci_lower_proportion,
                                     CI_Upper = analysis_result$ci_upper_proportion))
}

# Combine all posterior samples into one data frame
posterior_df <- do.call(rbind, posterior_samples_list)

# Ensure proportions are sorted in descending order
summary_data <- summary_data %>% arrange(desc(Mean))
summary_data$Histology <- factor(summary_data$Histology, levels = summary_data$Histology)
posterior_df$Histology <- factor(posterior_df$Histology, levels = summary_data$Histology)

# Filter and clean posterior data
posterior_df <- posterior_df %>% filter(is.finite(mu))
posterior_df <- na.omit(posterior_df)

# Define proper Y-axis alignment using a shared factor
shared_levels <- rev(levels(summary_data$Histology))
summary_data$Y_Alignment <- factor(summary_data$Histology, levels = shared_levels)
posterior_df$Y_Alignment <- factor(posterior_df$Histology, levels = shared_levels)

# Generate a gradient color palette that spans the number of unique histology types
color_gradient <- colorRampPalette(c("#FF6347", "#FFD700", "#32CD32", "#1E90FF", "#8A2BE2"))
bayesian_gradient_colors <- color_gradient(length(unique(summary_data$Histology)))

# Create the column titles
column_titles <- ggplot() +
  annotate("text", x = 0.25, y = 1, label = "Histology", size = 4, fontface = "bold", hjust = 0) +
  annotate("text", x = 0.75, y = 1, label = "Posterior Mean [95% CrI]", size = 4, fontface = "bold", hjust = 1)
+
  theme_void()

# Create the text table
text_plot <- ggplot(summary_data, aes(y = Y_Alignment)) +
  geom_text(aes(x = -0.05, label = Histology), hjust = 0, size = 4) +
  geom_text(aes(x = 0.48, label = sprintf("%.3f [%.3f, %.3f]", Mean, CI_Lower, CI_Upper)), hjust = 1, size =
4) +
  scale_y_discrete(limits = shared_levels) +
  theme_void()

# Create the density plot
density_plot <- ggplot() +
  geom_density_ridges(data = posterior_df, aes(x = exp(mu) / (1 + exp(mu)), y = Y_Alignment, fill =
Histology),
                      alpha = 0.7, scale = 1.5) +
  geom_point(data = summary_data, aes(x = Mean, y = Y_Alignment), color = "black", size = 3) +
  geom_errorbarh(data = summary_data, aes(xmin = CI_Lower, xmax = CI_Upper, y = Y_Alignment),
                 height = 0.2, color = "black") +
  scale_y_discrete(limits = shared_levels) +
  scale_x_continuous(limits = c(-0.02, 0.6)) +  # Maintain original X spacing
```

```
  scale_fill_manual(values = bayesian_gradient_colors) +
  labs(x = "Estimated Proportion of Events", y = NULL) +
  theme_minimal() +
  theme(legend.position = "none", axis.text.y = element_blank())

# Combine everything into the final plot
final_plot <- (column_titles / (text_plot + density_plot)) + plot_layout(ncol = 1, heights = c(0.1, 1))
final_plot <- final_plot + plot_annotation(title = "Bayesian Meta-analysis of Proportion of Events by
Histology") &
  theme(plot.title = element_text(hjust = 0.5, size = 14, face = "bold"))

# Display final plot
print(final_plot)
```

Bayesian separated analysis small studies

```
# Load necessary libraries
library(dplyr)
library(bayesmeta)
library(ggplot2)
library(ggridges)
library(scales)
library(patchwork)

# Load the RMS Location data
RMS_Location <- read.csv("~/Desktop/RMS Location.csv", sep = ";")

# Verify column names
print(colnames(RMS_Location))

# Define function to run Bayesian meta-analysis and extract posterior samples
run_analysis_with_posterior <- function(data_subset) {
  pseudocount <- 0.5
  data_subset <- data_subset %>%
    mutate(
      adjusted_proportion = (Events + pseudocount) / (Total + 2 * pseudocount),
      adjusted_variance = (adjusted_proportion * (1 - adjusted_proportion)) / Total,
      logit_proportion = log(adjusted_proportion / (1 - adjusted_proportion)),
      logit_variance = adjusted_variance / (adjusted_proportion * (1 - adjusted_proportion))^2
    ) %>%
    filter(is.finite(logit_proportion) & is.finite(logit_variance) & logit_variance > 0)

  # Run Bayesian meta-analysis
  result <- bayesmeta(y = data_subset$logit_proportion,
                      sigma = sqrt(data_subset$logit_variance),
                      tau.prior = function(x) dhalfnormal(x, scale = 1))

  # Extract posterior samples for visualization
  posterior_samples <- data.frame(mu = result$rposterior(n = 1000)[, "mu"])

  # Extract summary statistics
  mean_logit <- result$summary[3, 2]
  ci_lower_logit <- result$summary[5, 2]
  ci_upper_logit <- result$summary[6, 2]

  mean_proportion <- exp(mean_logit) / (1 + exp(mean_logit))
  ci_lower_proportion <- exp(ci_lower_logit) / (1 + exp(ci_lower_logit))
  ci_upper_proportion <- exp(ci_upper_logit) / (1 + exp(ci_upper_logit))

  list(posterior_samples = posterior_samples,
       mean_proportion = mean_proportion,
       ci_lower_proportion = ci_lower_proportion,
       ci_upper_proportion = ci_upper_proportion)
}

# Generate separate plots for each Location
for (location in unique(RMS_Location$Location)) {
  # Filter data for the current Location
  location_data <- RMS_Location %>% filter(Location == location)

  # Run the analysis for each Author within the Location
  posterior_samples_list <- list()
  summary_data <- data.frame()
```

```
  for (author in unique(location_data$Author)) {
    subset_data <- location_data %>% filter(Author == author)
    analysis_result <- run_analysis_with_posterior(subset_data)

    # Store posterior samples with Author info
    analysis_result$posterior_samples$Author <- author
    posterior_samples_list[[author]] <- analysis_result$posterior_samples

    # Store summary data for point estimates and CIs
    summary_data <- rbind(summary_data,
                  data.frame(Author = author,
                        Mean = analysis_result$mean_proportion,
                        CI_Lower = analysis_result$ci_lower_proportion,
                        CI_Upper = analysis_result$ci_upper_proportion))
  }

  # Calculate overall Location proportion
  overall_result <- run_analysis_with_posterior(location_data)
  overall_result$posterior_samples$Author <- "Overall"
  posterior_samples_list[["Overall"]] <- overall_result$posterior_samples

  # Add overall value to summary_data
  summary_data <- rbind(summary_data,
                data.frame(Author = "Overall",
                      Mean = overall_result$mean_proportion,
                      CI_Lower = overall_result$ci_lower_proportion,
                      CI_Upper = overall_result$ci_upper_proportion))

  # Combine all posterior samples into one data frame
  posterior_df <- do.call(rbind, posterior_samples_list)

  # Ensure proportions are sorted in descending order
  summary_data <- summary_data %>% arrange(desc(Mean))
  summary_data$Author <- factor(summary_data$Author, levels = summary_data$Author)
  posterior_df$Author <- factor(posterior_df$Author, levels = summary_data$Author)

  # Filter and clean posterior data
  posterior_df <- posterior_df %>% filter(is.finite(mu))
  posterior_df <- na.omit(posterior_df)

  # Define proper Y-axis alignment
  shared_levels <- rev(levels(summary_data$Author))
  summary_data$Y_Alignment <- factor(summary_data$Author, levels = shared_levels)
  posterior_df$Y_Alignment <- factor(posterior_df$Author, levels = shared_levels)

  # Generate a gradient color palette for each Location
  gradient_colors <- colorRampPalette(c("#FF6347", "#FFD700", "#32CD32", "#1E90FF", "#8A2BE2"))
  bayesian_gradient_colors <- gradient_colors(length(unique(summary_data$Author)))

  # Make "Overall" bold in text plot
  summary_data <- summary_data %>%
    mutate(Fontface = ifelse(Author == "Overall", "bold", "plain"))

  # Create the column titles
  column_titles <- ggplot() +
    annotate("text", x = 0.03, y = 1, label = paste("Author                    Posterior Mean [95% CrI]",
sep=""),
         size = 4, fontface = "bold", hjust = 1) +
    theme_void()
```

```
  # Create the text table
  text_plot <- ggplot(summary_data, aes(y = Y_Alignment)) +
    geom_text(aes(x = -0.05, label = Author, fontface = Fontface), hjust = 0, size = 4) +
    geom_text(aes(x = 0.48, label = sprintf("%.3f [%.3f, %.3f]", Mean, CI_Lower, CI_Upper), fontface =
Fontface),
          hjust = 1, size = 4) +
    scale_y_discrete(limits = shared_levels) +
    theme_void()

  # Create the density plot
  density_plot <- ggplot() +
    geom_density_ridges(data = posterior_df, aes(x = exp(mu) / (1 + exp(mu)), y = Y_Alignment, fill =
Author),
              alpha = 0.7, scale = 1.5) +
    geom_point(data = summary_data, aes(x = Mean, y = Y_Alignment), color = "black", size = 3) +
    geom_errorbarh(data = summary_data, aes(xmin = CI_Lower, xmax = CI_Upper, y = Y_Alignment),
            height = 0.2, color = "black") +
    scale_y_discrete(limits = shared_levels) +
    scale_x_continuous(limits = c(-0.1, 1.05)) +
    scale_fill_manual(values = bayesian_gradient_colors) +
    labs(x = "Estimated Proportion of Events", y = NULL) +
    theme_minimal() +
    theme(legend.position = "none", axis.text.y = element_blank())

  # Combine everything into the final plot
  final_plot <- (column_titles / (text_plot + density_plot)) + plot_layout(ncol = 1, heights = c(0.01, 1))
  final_plot <- final_plot + plot_annotation(title = paste("Bayesian Meta-analysis -", location)) &
    theme(plot.title = element_text(hjust = 0.5, size = 14, face = "bold"))

  # Display final plot
  print(final_plot)
}
```

Bayesian full analysis

```
# Load necessary libraries
library(dplyr)
library(bayesmeta)
library(ggplot2)
library(ggridges)
library(scales)
library(patchwork)

# Load the RMS Sensitivity data
RMS_Sensitivity <- read.csv("~/Desktop/RMS Sensitivity.csv", sep = ";")

# Define function to run Bayesian meta-analysis and extract posterior samples
run_analysis_with_posterior <- function(data_subset) {
  pseudocount <- 0.5
  data_subset <- data_subset %>%
    mutate(
      adjusted_proportion = (Events + pseudocount) / (Total + 2 * pseudocount),
      adjusted_variance = (adjusted_proportion * (1 - adjusted_proportion)) / Total,
      logit_proportion = log(adjusted_proportion / (1 - adjusted_proportion)),
      logit_variance = adjusted_variance / (adjusted_proportion * (1 - adjusted_proportion))^2
    ) %>%
    filter(is.finite(logit_proportion) & is.finite(logit_variance) & logit_variance > 0)

  # Run Bayesian meta-analysis
  result <- bayesmeta(y = data_subset$logit_proportion,
                sigma = sqrt(data_subset$logit_variance),
                tau.prior = function(x) dhalfnormal(x, scale = 1))

  # Extract posterior samples for visualization
  posterior_samples <- data.frame(mu = result$rposterior(n = 1000)[, "mu"])

  # Extract summary statistics
  mean_logit <- result$summary[3, 2]
  ci_lower_logit <- result$summary[5, 2]
  ci_upper_logit <- result$summary[6, 2]

  mean_proportion <- exp(mean_logit) / (1 + exp(mean_logit))
  ci_lower_proportion <- exp(ci_lower_logit) / (1 + exp(ci_lower_logit))
  ci_upper_proportion <- exp(ci_upper_logit) / (1 + exp(ci_upper_logit))

  list(posterior_samples = posterior_samples,
     mean_proportion = mean_proportion,
     ci_lower_proportion = ci_lower_proportion,
     ci_upper_proportion = ci_upper_proportion)
}

# Run the analysis for each Author and store results
posterior_samples_list <- list()
summary_data <- data.frame()

for (author in unique(RMS_Sensitivity$Author)) {
  subset_data <- RMS_Sensitivity %>% filter(Author == author)
  analysis_result <- run_analysis_with_posterior(subset_data)

  # Store posterior samples with Author info
  analysis_result$posterior_samples$Author <- author
  posterior_samples_list[[author]] <- analysis_result$posterior_samples
```

```
    # Store summary data for point estimates and CIs
    summary_data <- rbind(summary_data,
                          data.frame(Author = author,
                                     Mean = analysis_result$mean_proportion,
                                     CI_Lower = analysis_result$ci_lower_proportion,
                                     CI_Upper = analysis_result$ci_upper_proportion))
}

# Combine all posterior samples into one data frame
posterior_df <- do.call(rbind, posterior_samples_list)

# Ensure proportions are sorted in descending order
summary_data <- summary_data %>% arrange(desc(Mean))
summary_data$Author <- factor(summary_data$Author, levels = summary_data$Author)
posterior_df$Author <- factor(posterior_df$Author, levels = summary_data$Author)

# Filter and clean posterior data
posterior_df <- posterior_df %>% filter(is.finite(mu))
posterior_df <- na.omit(posterior_df)

# Define proper Y-axis alignment using a shared factor
shared_levels <- rev(levels(summary_data$Author))
summary_data$Y_Alignment <- factor(summary_data$Author, levels = shared_levels)
posterior_df$Y_Alignment <- factor(posterior_df$Author, levels = shared_levels)

# Generate a gradient color palette that spans the number of unique Authors
gradient_colors <- colorRampPalette(c("#FF6347", "#FFD700", "#32CD32", "#1E90FF", "#8A2BE2"))
bayesian_gradient_colors <- gradient_colors(length(unique(summary_data$Author)))

# Create the column titles
column_titles <- ggplot() +
    annotate("text", x = 0.02, y = 1, label = "Author                        Posterior Mean [95% CrI]", size = 4,
fontface = "bold", hjust = 1) +
    theme_void()

# Create the text table
text_plot <- ggplot(summary_data, aes(y = Y_Alignment)) +
    geom_text(aes(x = -0.05, label = Author), hjust = 0, size = 4) +
    geom_text(aes(x = 0.48, label = sprintf("%.3f [%.3f, %.3f]", Mean, CI_Lower, CI_Upper)), hjust = 1, size
= 4) +
    scale_y_discrete(limits = shared_levels) +
    theme_void()

# Create the density plot
density_plot <- ggplot() +
    geom_density_ridges(data = posterior_df, aes(x = exp(mu) / (1 + exp(mu)), y = Y_Alignment, fill =
Author),
                        alpha = 0.7, scale = 1.5) +
    geom_point(data = summary_data, aes(x = Mean, y = Y_Alignment), color = "black", size = 3) +
    geom_errorbarh(data = summary_data, aes(xmin = CI_Lower, xmax = CI_Upper, y = Y_Alignment),
                   height = 0.2, color = "black") +
    scale_y_discrete(limits = shared_levels) +
    scale_x_continuous(limits = c(-0.1, 1.05)) +  # Updated x-axis limits
    scale_fill_manual(values = bayesian_gradient_colors) +
    labs(x = "Estimated Proportion of Events", y = NULL) +
    theme_minimal() +
    theme(legend.position = "none", axis.text.y = element_blank())

# Combine everything into the final plot
```

```
final_plot <- (column_titles / (text_plot + density_plot)) + plot_layout(ncol = 1, heights = c(0.01, 1))
final_plot <- final_plot + plot_annotation(title = "Bayesian Meta-analysis of Proportion of Events by
Author") &
  theme(plot.title = element_text(hjust = 0.5, size = 14, face = "bold"))

# Display final plot
print(final_plot)
```

Posterior mean proportions

```
# Load necessary libraries
library(dplyr)
library(bayesmeta)

# Load the data
sensitivity_data <- read.csv("~/Desktop/RMS Location.csv", sep = ";")

# Define function to run Bayesian meta-analysis
run_analysis <- function(data_subset) {
  pseudocount <- 0.5
  data_subset <- data_subset %>%
    mutate(
      adjusted_proportion = (Events + pseudocount) / (Total + 2 * pseudocount),
      adjusted_variance = (adjusted_proportion * (1 - adjusted_proportion)) / Total,
      logit_proportion = log(adjusted_proportion / (1 - adjusted_proportion)),
      logit_variance = adjusted_variance / (adjusted_proportion * (1 - adjusted_proportion))^2
    ) %>%
    filter(is.finite(logit_proportion) & is.finite(logit_variance) & logit_variance > 0)

  # Run Bayesian meta-analysis
  bayesmeta(y = data_subset$logit_proportion,
         sigma = sqrt(data_subset$logit_variance),
         tau.prior = function(x) dhalfnormal(x, scale = 1))
}

# Check if the data contains sufficient information for analysis
if (nrow(sensitivity_data) > 0) {
  # Loop through each unique Location
  for (location in unique(sensitivity_data$Location)) {
    cat("\n============================================\n")
    cat("Bayesian Meta-analysis for Location:", location, "\n")
    cat("============================================\n")

    # Filter data for the current Location
    location_data <- sensitivity_data %>% filter(Location == location)

    if (nrow(location_data) > 0) {
      # Run the analysis for this location
      result <- run_analysis(location_data)

      # Extract posterior mean and credible interval for the logit-transformed proportion
      mu_mean <- result$summary[3, 2]  # Mean of mu (posterior mean of logit proportion)
      mu_ci_lower <- result$summary[5, 2]  # Lower bound of 95% CI
      mu_ci_upper <- result$summary[6, 2]  # Upper bound of 95% CI

      # Convert logit values back to proportions (posterior mean proportion and CI)
      mean_proportion <- exp(mu_mean) / (1 + exp(mu_mean))
      ci_lower <- exp(mu_ci_lower) / (1 + exp(mu_ci_lower))
      ci_upper <- exp(mu_ci_upper) / (1 + exp(mu_ci_upper))

      # Calculate the estimated percentage of events
      estimated_percentage <- mean_proportion * 100
      ci_lower_percentage <- ci_lower * 100
      ci_upper_percentage <- ci_upper * 100

      # Display the results
      cat("Posterior Mean Proportion of Events:", round(mean_proportion, 4), "\n")
```

```
        cat("95% Credible Interval for Proportion:", round(ci_lower, 4), "-", round(ci_upper, 4), "\n")
        cat("Estimated Percentage of Events:", round(estimated_percentage, 2), "%\n")
        cat("95% Credible Interval for Percentage:", round(ci_lower_percentage, 2), "% -",
round(ci_upper_percentage, 2), "%\n")
      } else {
        cat("No data available for this location.\n")
      }
    }
} else {
  message("Meta-analysis could not be completed due to insufficient data.")
}
```

Between study variability

```
# Load necessary libraries
library(dplyr)
library(bayesmeta)

# Load the data
sensitivity_data <- read.csv("~/Desktop/RMS Location.csv", sep = ";")

# Define function to run Bayesian meta-analysis
run_analysis <- function(data_subset) {
  pseudocount <- 0.5
  data_subset <- data_subset %>%
    mutate(
      adjusted_proportion = (Events + pseudocount) / (Total + 2 * pseudocount),
      adjusted_variance = (adjusted_proportion * (1 - adjusted_proportion)) / Total,
      logit_proportion = log(adjusted_proportion / (1 - adjusted_proportion)),
      logit_variance = adjusted_variance / (adjusted_proportion * (1 - adjusted_proportion))^2
    ) %>%
    filter(is.finite(logit_proportion) & is.finite(logit_variance) & logit_variance > 0)

  # Run Bayesian meta-analysis
  bayesmeta(y = data_subset$logit_proportion,
        sigma = sqrt(data_subset$logit_variance),
        tau.prior = function(x) dhalfnormal(x, scale = 1))
}

# Check if the data contains sufficient information for analysis
if (nrow(sensitivity_data) > 0) {
  # Loop through each unique Location
  for (location in unique(sensitivity_data$Location)) {
    cat("\n============================================\n")
    cat("Bayesian Meta-analysis for Location:", location, "\n")
    cat("============================================\n")

    # Filter data for the current Location
    location_data <- sensitivity_data %>% filter(Location == location)

    if (nrow(location_data) > 0) {
      # Run the analysis for this location
      result <- run_analysis(location_data)

      # Check if the analysis result is available
      if (!is.null(result)) {
        # Extract the posterior mean and 95% credible interval for τ (between-study standard deviation)
        tau_mean <- result$summary[3, "tau"]  # Posterior mean of τ
        tau_ci_lower <- result$summary[5, "tau"]  # Lower bound of 95% CI for τ
        tau_ci_upper <- result$summary[6, "tau"]  # Upper bound of 95% CI for τ

        # Display the results
        cat("Posterior Mean of Between-Study Standard Deviation (τ):", round(tau_mean, 4), "\n")
        cat("95% Credible Interval for τ:", round(tau_ci_lower, 4), "-", round(tau_ci_upper, 4), "\n")
      } else {
        cat("Meta-analysis could not be completed for this location.\n")
      }
    } else {
      cat("No data available for this location.\n")
    }
  }
```

```
} else {
  message("Insufficient data for meta-analysis.")
}
```

Density plot small studies

```
# Load necessary libraries
library(bayesmeta)
library(ggplot2)
library(dplyr)

# Load your sensitivity data
sensitivity_data <- read.csv("~/Desktop/NRSTS Histology.csv", sep = ";")

# Check for required columns
if (!all(c("Histology", "Total", "Events") %in% colnames(sensitivity_data))) {
  stop("Data is missing required columns: 'Histology', 'Total', or 'Events'.")
}

# Define pseudocount
pseudocount <- 0.5

# Adjust data and perform Bayesian meta-analysis
sensitivity_data <- sensitivity_data %>%
  mutate(
    adjusted_proportion = (Events + pseudocount) / (Total + 2 * pseudocount),
    adjusted_variance = (adjusted_proportion * (1 - adjusted_proportion)) / Total,
    logit_proportion = log(adjusted_proportion / (1 - adjusted_proportion)),
    logit_variance = adjusted_variance / (adjusted_proportion * (1 - adjusted_proportion))^2
  ) %>%
  filter(is.finite(logit_proportion) & is.finite(logit_variance) & logit_variance > 0)

# Run Bayesian meta-analysis on the dataset
result <- bayesmeta(
  y = sensitivity_data$logit_proportion,
  sigma = sqrt(sensitivity_data$logit_variance),
  tau.prior = function(x) dhalfnormal(x, scale = 1)
)

# Define sample size
sample_size <- min(6000, nrow(sensitivity_data) * 100)

# Sample from prior and posterior distributions
prior_samples <- rnorm(sample_size, mean = 0, sd = 1)  # Prior samples with prior mean 0 and sd 1
posterior_samples <- result$rposterior(n = sample_size)[, "mu"]  # Posterior samples for mu

# Ensure likelihood samples match sample_size
likelihood_samples <- rep(sensitivity_data$logit_proportion, length.out = sample_size)

# Combine samples for plotting, with Histology replicated for faceting
density_data <- data.frame(
  Value = c(prior_samples, likelihood_samples, posterior_samples),
  Distribution = factor(rep(c("Prior, p(μ)", "Likelihood, p(y|μ)", "Posterior, p(μ|y)"), each = sample_size)),
  Histology = rep(sensitivity_data$Histology, length.out = sample_size * 3)  # Ensure correct length
)

# Plot densities with facet for each Histology
ggplot(density_data, aes(x = Value, fill = Distribution)) +
  geom_density(alpha = 0.5) +
  scale_fill_manual(values = c("blue", "orange", "green")) +
  labs(title = "Density Plot of Prior, Likelihood, and Posterior Distributions by Histology",
       x = "Value",
       y = "Density",
```

```
       fill = "Distribution") +
  theme_minimal() +
  theme(legend.position = "top") +
  facet_wrap(~ Histology, ncol = 2, labeller = as_labeller(function(x) paste("Histology:", x)))  # 2 plots per
row
```

Trends 1.

```
# Load necessary libraries
library(ggplot2)
library(dplyr)
library(broom)

# Load the dataset (Ensure correct delimiter ";")
data <- read.csv("~/Desktop/Trends RMS NRSTS.csv", sep=";")

# Trim column names to remove spaces
colnames(data) <- trimws(colnames(data))

# Rename columns for easier usage
data <- data %>%
  rename(
    sarcoma_subtype = Sarcoma.subtype,
    total = Total,
    event = Event,
    midstudy_period = Midstudy.period
  )

# Remove rows where midstudy_period is "-"
data <- data %>% filter(midstudy_period != "-")

# Convert midstudy_period to numeric
data$midstudy_period <- as.numeric(as.character(data$midstudy_period))

# Remove rows where midstudy_period is NA
data <- data %>% filter(!is.na(midstudy_period))

# Ensure numeric data types for calculations
data <- data %>%
  mutate(
    event = as.numeric(event),
    total = as.numeric(total),
    probability = event / total # Ensure probability is correctly calculated
  )

# Define colors for RMS and NRSTS
color_map <- c("RMS" = "#FF6347", "NRSTS" = "#1E90FF") # Red for RMS, Blue for NRSTS

# Fit logistic regression models for both RMS and NRSTS
model_list <- list()
prediction_data_list <- list()

for (subtype in unique(data$sarcoma_subtype)) {
  df_sub <- data %>% filter(sarcoma_subtype == subtype)

  # Check if there are valid data points
  if (nrow(df_sub) < 2) {
    message("Skipping ", subtype, " - Not enough data points.")
    next
  }

  # Fit logistic regression model
  model <- glm(cbind(event, total - event) ~ midstudy_period,
             data = df_sub,
             family = binomial)
```

```
  # Generate prediction data
  prediction_data <- data.frame(
    midstudy_period = seq(min(df_sub$midstudy_period, na.rm=TRUE),
                    max(df_sub$midstudy_period, na.rm=TRUE),
                    length.out = 100)
  )

  prediction_data$predicted <- predict(model, newdata = prediction_data, type = "response")

  # Confidence interval calculation
  conf_int <- predict(model, newdata = prediction_data, type = "link", se.fit = TRUE)
  prediction_data$conf_low <- plogis(conf_int$fit - 1.96 * conf_int$se.fit)
  prediction_data$conf_high <- plogis(conf_int$fit + 1.96 * conf_int$se.fit)
  prediction_data$sarcoma_subtype <- subtype  # Add subtype column for plotting

  # Store model and prediction data
  model_list[[subtype]] <- model
  prediction_data_list[[subtype]] <- prediction_data
}

# Combine all prediction data into one dataframe
prediction_data_all <- bind_rows(prediction_data_list)

# Plot RMS and NRSTS on the same graph with centered title
p <- ggplot(data, aes(x = midstudy_period, y = probability, color = sarcoma_subtype)) +
  geom_point(aes(size = total), alpha = 0.7) +
  geom_line(data = prediction_data_all, aes(x = midstudy_period, y = predicted, color = sarcoma_subtype),
size = 1) +
  geom_ribbon(data = prediction_data_all, aes(x = midstudy_period, ymin = conf_low, ymax = conf_high,
fill = sarcoma_subtype),
            alpha = 0.2, inherit.aes = FALSE, show.legend = FALSE) +  # Keep CI but remove its legend
  scale_color_manual(values = color_map) +
  scale_fill_manual(values = color_map, guide = "none") +
  labs(title = "Logistic Regression Trend for RMS and NRSTS",
       x = "Mid Study Period",
       y = "Probability of Event",
       color = "Sarcoma Subtype",
       size = "Total Cases") +
  theme_minimal() +
  theme(plot.title = element_text(hjust = 0.5))  # CENTER THE TITLE

# Print the plot
print(p)

# Statistical analysis function
analyze_trend <- function(df, subtype) {
  df_sub <- df %>% filter(sarcoma_subtype == subtype)

  # Skip if insufficient data
  if (nrow(df_sub) < 2) {
    message("Skipping ", subtype, " - Not enough data for trend analysis.")
    return(NULL)
  }

  # Fit logistic regression model
  model <- glm(cbind(event, total - event) ~ midstudy_period,
           data = df_sub,
           family = binomial)
```

```
  # Summarize model results
  summary_model <- summary(model)

  # Extract p-value for trend significance
  p_value <- coef(summary_model)[2, 4]

  # Interpretation
  if (p_value < 0.05) {
    message(subtype, " shows a significant trend over time (p = ", round(p_value, 3), ").")
  } else {
    message(subtype, " does not show a significant trend over time (p = ", round(p_value, 3), ").")
  }
}

# Perform trend analysis for each sarcoma subtype
for (subtype in unique(data$sarcoma_subtype)) {
  analyze_trend(data, subtype)
}
```

Trends 2.

```
# Load necessary libraries
library(ggplot2)
library(dplyr)
library(glmnet)

# Load the dataset
data <- read.csv("~/Desktop/Trend RMS Histology.csv", sep=";")

# Trim column names
colnames(data) <- trimws(colnames(data))

# Rename columns
data <- data %>%
  rename(
    histology = Histology,
    total = Total,
    events = Events,
    end_year = End  # Replace Start with End
  )

# Remove invalid end_year
data <- data %>% filter(end_year != "-")
data$end_year <- as.numeric(as.character(data$end_year))
data <- data %>% filter(!is.na(end_year))

# Ensure numeric values
data <- data %>%
  mutate(
    events = as.numeric(events),
    total = as.numeric(total),
    probability = events / total
  )

# Define colors
gradient_colors <- colorRampPalette(c("#FF6347", "#FFD700", "#32CD32", "#1E90FF", "#8A2BE2"))
unique_histologies <- unique(data$histology)
color_map <- setNames(gradient_colors(length(unique_histologies)), unique_histologies)

# Fit logistic regression or LOESS for each histology
model_list <- list()
prediction_data_list <- list()

for (hist in unique_histologies) {
  df_sub <- data %>% filter(histology == hist)

  # Skip if not enough variation
  if (n_distinct(df_sub$end_year) < 2) {
    message("Skipping ", hist, " - Not enough unique years.")
    next
  }

  # Skip if perfect separation
  if (all(df_sub$events == 0) | all(df_sub$events == df_sub$total)) {
    message("Skipping ", hist, " - Perfect separation.")
    next
  }
```

```
  # Try logistic regression first
  model <- tryCatch({
    glm(cbind(events, total - events) ~ end_year,
        data = df_sub,
        family = binomial)
  }, error = function(e) {
    message("Logistic regression failed for ", hist, ". Trying LOESS instead.")
    return(NULL)
  })

  if (is.null(model)) {
    # Use LOESS as a fallback
    loess_model <- loess(probability ~ end_year, data = df_sub, span = 0.75)

    # Generate prediction data
    prediction_data <- data.frame(
      end_year = seq(min(df_sub$end_year, na.rm=TRUE),
                     max(df_sub$end_year, na.rm=TRUE),
                     length.out = 100)
    )

    prediction_data$predicted <- predict(loess_model, newdata = prediction_data)

    # Approximate confidence intervals
    prediction_data$conf_low <- prediction_data$predicted * 0.9
    prediction_data$conf_high <- prediction_data$predicted * 1.1
    prediction_data$histology <- hist
  } else {
    # Generate logistic regression prediction data
    prediction_data <- data.frame(
      end_year = seq(min(df_sub$end_year, na.rm=TRUE),
                     max(df_sub$end_year, na.rm=TRUE),
                     length.out = 100)
    )

    prediction_data$predicted <- predict(model, newdata = prediction_data, type = "response")

    # Confidence intervals (approximate)
    prediction_data$conf_low <- prediction_data$predicted * 0.9
    prediction_data$conf_high <- prediction_data$predicted * 1.1
    prediction_data$histology <- hist
  }

  # Store data
  prediction_data_list[[hist]] <- prediction_data
}

# Check if prediction data exists before merging
if (length(prediction_data_list) > 0) {
  prediction_data_all <- bind_rows(prediction_data_list)
} else {
  stop("No valid prediction data available. Check data for issues.")
}

# Plot histologies
p <- ggplot(data, aes(x = end_year, y = probability, color = histology)) +
  geom_point(aes(size = total), alpha = 0.7) +
  geom_line(data = prediction_data_all, aes(x = end_year, y = predicted, color = histology), size = 1) +
  geom_ribbon(data = prediction_data_all, aes(x = end_year, ymin = conf_low, ymax = conf_high, fill =
histology),
```

```
            alpha = 0.2, inherit.aes = FALSE, show.legend = FALSE) +
  scale_color_manual(values = color_map) +
  scale_fill_manual(values = color_map, guide = "none") +
  labs(title = "Trend Analysis for Different Histologies",
       x = "End Year",
       y = "Probability of Event",
       color = "Histology",
       size = "Total Cases") +
  theme_minimal() +
  theme(plot.title = element_text(hjust = 0.5))  # Center the title

# Print the plot
print(p)
```
