# Supplementary material for: Prevalence of locoregional and distant lymph node metastases in children and adolescents/young adults with soft tissue sarcomas: a Bayesian meta-analysis of proportions
Source: eClinicalMedicine. 2025 Aug 7;87:103390. doi: 10.1016/j.eclinm.2025.103390 (PMC12355419; doi:10.1016/j.eclinm.2025.103390)
Supplement: Supplementary Table S1 [file mmc3.docx]

**Appendix 1. Summary search strategy**

The following search terms were used: (pediatric* OR paediatric* OR child* OR kid* OR adolescent* OR young adult*) AND (rhabdomyosarcoma OR RMS OR soft-tissue sarcoma OR STS OR desmoplastic round cell tumor OR DSRCT OR clear cell sarcoma OR angiosarcoma OR epithelioid sarcoma OR leiomyosarcoma OR hemangioendothelioma OR liposarcoma OR fibrosarcoma OR soft-tissue Ewing sarcoma OR malignant peripheral nerve sheath tumor OR MPNST OR synovial sarcoma OR alveolar soft part sarcoma OR dermatofibrosarcoma protuberans OR peripheral primitive neuroectodermal tumor OR PNET) AND (lymph node* OR nodal OR nodes OR lymph node metastases OR lymphatic metastases OR lymphatic OR lymphatic vessels OR lymphatic system OR sentinel).
