# Supplementary material for: Prevalence of locoregional and distant lymph node metastases in children and adolescents/young adults with soft tissue sarcomas: a Bayesian meta-analysis of proportions
Source: eClinicalMedicine. 2025 Aug 7;87:103390. doi: 10.1016/j.eclinm.2025.103390 (PMC12355419; doi:10.1016/j.eclinm.2025.103390)
Supplement: Supplementary Table S2 [file mmc4.docx]

**Extracted variables**

First Author (year of publication)

PMID

Year of publication

Region of research Institution

Data source

Study period

All patients (number of patients)

All patients with LN data

Age at time of diagnosis (year) with median and range

IRS-Group

TNM

Tumor size

Fusion status

LN positivity detection (Clinical, Surgical, Radiological)

LN sampling (Sentinel, Local, Distant)

Sarcoma subtype

Localization

Histology
