# Supplementary material for: Prevalence of locoregional and distant lymph node metastases in children and adolescents/young adults with soft tissue sarcomas: a Bayesian meta-analysis of proportions
Source: eClinicalMedicine. 2025 Aug 7;87:103390. doi: 10.1016/j.eclinm.2025.103390 (PMC12355419; doi:10.1016/j.eclinm.2025.103390)
Supplement: Supplementary Table S3 [file mmc5.docx]

| **List of Register Studies** | | | | | | | | |
| --- | --- | --- | --- | --- | --- | --- | --- | --- |
|  | Overall | | RMS | | NRSTS | | Mixed RMS/NRSTS cohort | |
|  | n = 263 | % | n = 147 | % | n = 106 | % | n = 10 | % |
|  |  |  |  |  |  |  |  |  |
| *AHWTS* | 1 | 0.38 | 0 | 0 | 1 | 0.94 | 0 | 0 |
| *AIEOP Centers* | 6 | 2.28 | 1 | 0.68 | 5 | 4.72 | 0 | 0 |
| *AREN0321* | 1 | 0.38 | 0 | 0 | 1 | 0.94 | 0 | 0 |
| *AREN03B2* | 1 | 0.38 | 0 | 0 | 1 | 0.94 | 0 | 0 |
| *ARST0531* | 1 | 0.38 | 1 | 0.68 | 0 | 0 | 0 | 0 |
| *ARST08P1* | 1 | 0.38 | 1 | 0.68 | 0 | 0 | 0 | 0 |
| *BERNIE* | 3 | 1.14 | 1 | 0.68 | 2 | 1.89 | 0 | 0 |
| *BSTTR* | 1 | 0.38 | 0 | 0 | 1 | 0.94 | 0 | 0 |
| *COG - ARST0331* | 11 | 4.18 | 11 | 7.48 | 0 | 0 | 0 | 0 |
| *COG - ARST0332* | 4 | 1.52 | 0 | 0 | 4 | 3.77 | 0 | 0 |
| *COG - ARST0431* | 8 | 3.04 | 8 | 5.44 | 0 | 0 | 0 | 0 |
| *COG - ARST0531* | 9 | 3.42 | 9 | 6.12 | 0 | 0 | 0 | 0 |
| *COG - ARST08P1* | 7 | 2.66 | 7 | 4.76 | 0 | 0 | 0 | 0 |
| *COG - ARST1321* | 1 | 0.38 | 0 | 0 | 1 | 0.94 | 0 | 0 |
| *COG - D9602* | 13 | 4.94 | 13 | 8.84 | 0 | 0 | 0 | 0 |
| *COG - D9802* | 10 | 3.8 | 10 | 6.8 | 0 | 0 | 0 | 0 |
| *COG - D9803* | 13 | 4.94 | 13 | 8.84 | 0 | 0 | 0 | 0 |
| *CWS-2002* | 6 | 2.28 | 1 | 0.68 | 5 | 4.72 | 0 | 0 |
| *CWS-2002-P* | 23 | 8.75 | 10 | 6.8 | 13 | 12.26 | 0 | 0 |
| *CWS-2006* | 1 | 0.38 | 1 | 0.68 | 0 | 0 | 0 | 0 |
| *CWS-81* | 16 | 6.08 | 5 | 3.4 | 11 | 10.38 | 0 | 0 |
| *CWS-86* | 18 | 6.84 | 7 | 4.76 | 11 | 10.38 | 0 | 0 |
| *CWS-91* | 22 | 8.37 | 9 | 6.12 | 13 | 12.26 | 0 | 0 |
| *CWS-96* | 29 | 11.03 | 14 | 9.52 | 15 | 14.15 | 0 | 0 |
| *CWS-SoTiSaR* | 20 | 7.61 | 10 | 6.8 | 10 | 9.43 | 0 | 0 |
| *EpSSG MTS 2008* | 7 | 2.66 | 4 | 2.72 | 3 | 2.83 | 0 | 0 |
| *EpSSG NRSTS 2005* | 13 | 4.94 | 1 | 0.68 | 12 | 11.32 | 0 | 0 |
| *EpSSG RMS-2005* | 22 | 8.37 | 22 | 14.97 | 0 | 0 | 0 | 0 |
| *ESFT Database* | 1 | 0.38 | 0 | 0 | 1 | 0.94 | 0 | 0 |
| *French national LFS Database* | 1 | 0.38 | 1 | 0.68 | 0 | 0 | 0 | 0 |
| *ICG* | 2 | 0.76 | 0 | 0 | 2 | 1.89 | 0 | 0 |
| *INT Protocols* | 4 | 1.52 | 2 | 1.36 | 2 | 1.89 | 0 | 0 |
| *ISPOMMT* | 1 | 0.38 | 0 | 0 | 1 | 0.94 | 0 | 0 |
| *IRG RMS-4.99* | 3 | 1.14 | 3 | 2.04 | 0 | 0 | 0 | 0 |
| *IRG RMS-79* | 16 | 6.08 | 12 | 8.16 | 4 | 3.77 | 0 | 0 |
| *IRG RMS-88* | 17 | 6.46 | 13 | 8.84 | 4 | 3.77 | 0 | 0 |
| *IRG RMS-96* | 18 | 6.84 | 15 | 10.2 | 3 | 2.83 | 0 | 0 |
| *IRS-I* | 10 | 3.8 | 10 | 6.8 | 0 | 0 | 0 | 0 |
| *IRS-II* | 9 | 3.42 | 9 | 6.12 | 0 | 0 | 0 | 0 |
| *IRS-III* | 18 | 6.84 | 18 | 12.25 | 0 | 0 | 0 | 0 |
| *IRS-IV* | 21 | 7.99 | 21 | 14.29 | 0 | 0 | 0 | 0 |
| *IRS-IV pilot* | 12 | 4.56 | 12 | 8.16 | 0 | 0 | 0 | 0 |
| *IRS-V* | 1 | 0.38 | 1 | 0.68 | 0 | 0 | 0 | 0 |
| *IRS-D9501* | 1 | 0.38 | 1 | 0.68 | 0 | 0 | 0 | 0 |
| *IRSG* | 2 | 0.76 | 2 | 1.36 | 0 | 0 | 0 | 0 |
| *Instituto Nazionale Tumori of Milan* | 3 | 1.14 | 0 | 0 | 3 | 2.83 | 0 | 0 |
| *MMTIV89-91* | 2 | 0.76 | 2 | 1.36 | 0 | 0 | 0 | 0 |
| *MTS 2008* | 1 | 0.38 | 1 | 0.68 | 0 | 0 | 0 | 0 |
| *NCDB* | 4 | 1.52 | 2 | 1.36 | 2 | 1.89 | 0 | 0 |
| *NETSARC+* | 2 | 0.76 | 0 | 0 | 2 | 1.89 | 0 | 0 |
| *PPSTG* | 1 | 0.38 | 0 | 0 | 1 | 0.94 | 0 | 0 |
| *RMS4.99* | 1 | 0.38 | 1 | 0.68 | 0 | 0 | 0 | 0 |
| *SEER* | 7 | 2.66 | 6 | 4.08 | 1 | 0.94 | 0 | 0 |
| *SFCE Centers* | 1 | 0.38 | 0 | 0 | 1 | 0.94 | 0 | 0 |
| *SIOP 2001* | 2 | 0.76 | 0 | 0 | 2 | 1.89 | 0 | 0 |
| *SIOP 93-01* | 2 | 0.76 | 0 | 0 | 2 | 1.89 | 0 | 0 |
| *SIOP MMT-4* | 2 | 0.76 | 2 | 1.36 | 0 | 0 | 0 | 0 |
| *SIOP MMT-84* | 8 | 3.04 | 8 | 5.44 | 0 | 0 | 0 | 0 |
| *SIOP MMT-89* | 8 | 3.04 | 8 | 5.44 | 0 | 0 | 0 | 0 |
| *SIOP MMT-95* | 11 | 4.18 | 11 | 7.48 | 0 | 0 | 0 | 0 |
| *SIOP MMT-98* | 3 | 1.14 | 3 | 2.04 | 0 | 0 | 0 | 0 |
| *SIOP RMS-75* | 1 | 0.38 | 1 | 0.68 | 0 | 0 | 0 | 0 |
| *STSC Protocols* | 1 | 0.38 | 0 | 0 | 0 | 0 | 1 | 10 |
