# Supplementary material for: Prevalence of locoregional and distant lymph node metastases in children and adolescents/young adults with soft tissue sarcomas: a Bayesian meta-analysis of proportions
Source: eClinicalMedicine. 2025 Aug 7;87:103390. doi: 10.1016/j.eclinm.2025.103390 (PMC12355419; doi:10.1016/j.eclinm.2025.103390)
Supplement: Supplementary Table S4 [file mmc6.docx]

|  | **Low** | **23** | **163** | **203** | **102** | **57** | **216** | **210** | **11** |
| --- | --- | --- | --- | --- | --- | --- | --- | --- | --- |
|  | **Moderate** | **215** | **98** | **59** | **156** | **188** | **47** | **48** | **246** |
|  | **Seriuos** | **25** | **2** | **1** | **1** | **17** | **0** | **5** | **6** |
|  | **Unclear** | **0** | **0** | **0** | **4** | **1** | **0** | **0** | **0** |
| **No.** | **Article** | **D1** | **D2** | **D3** | **D4** | **D5** | **D6** | **D7** | **Overall** |
| **1** | **Affinita et al. (2018)** | Moderate | Moderate | Low | Low | Serious | Moderate | Low | Moderate |
| **2** | **Alcorn et al. (2013)** | Serious | Serious | Low | Low | Moderate | Moderate | Low | Serious |
| **3** | **Alvarez et al. (2024)** | Serious | Moderate | Low | Low | Moderate | Moderate | Low | Moderate |
| **4** | **Andreou et al. (2013)** | Serious | Moderate | Moderate | Low | Moderate | Moderate | Low | Moderate |
| **5** | **Angelini et al. (2016)** | Serious | Moderate | Moderate | Moderate | Moderate | Moderate | Moderate | Moderate |
| **6** | **Arndt et al. (2004)** | Moderate | Low | Low | Low | Moderate | Moderate | Moderate | Moderate |
| **7** | **Aya et al. (2021)** | Moderate | Low | Low | Moderate | Low | Low | Moderate | Moderate |
| **8** | **Baday at el. (2021)** | Moderate | Low | Low | Moderate | Moderate | Moderate | Moderate | Moderate |
| **9** | **Banerjee et al. (1997)** | Moderate | Moderate | Low | Moderate | Moderate | Low | Low | Moderate |
| **10** | **Ben-Arush et al. (2022)** | Moderate | Moderate | Moderate | Moderate | Serious | Moderate | Low | Moderate |
| **11** | **Benedetti et al. (2023)** | Moderate | Moderate | Low | Low | Moderate | Low | Low | Moderate |
| **12** | **Berlanga et al. (2023)** | Moderate | Low | Low | Moderate | Moderate | Moderate | Moderate | Moderate |
| **13** | **Bisogno et al. (2000)** | Moderate | Low | Low | Moderate | Moderate | Low | Low | Moderate |
| **14** | **Bisogno et al. (2008)** | Moderate | Moderate | Low | Moderate | Moderate | Moderate | Moderate | Moderate |
| **15** | **Bisogno et al. (2011)** | Serious | Moderate | Moderate | Moderate | Serious | Moderate | Low | Moderate |
| **16** | **Bisogno et al. (2012)** | Low | Low | Low | Low | Low | Low | Low | Low |
| **17** | **Bisogno et al. (2018)** | Moderate | Low | Moderate | Moderate | Moderate | Moderate | Low | Moderate |
| **18** | **Bisogno et al. (2021)** | Moderate | Low | Low | Low | Low | Low | Low | Moderate |
| **19** | **Bisogno et al. (2021)** | Moderate | Low | Moderate | Moderate | Low | Low | Low | Moderate |
| **20** | **Bisogno et al. (2023)** | Low | Low | Low | Low | Moderate | Moderate | Low | Low |
| **21** | **Blakely et al. (1999)** | Moderate | Moderate | Low | Moderate | Moderate | Low | Low | Moderate |
| **22** | **Blakely et al. (2003)** | Moderate | Low | Low | Moderate | Low | Low | Low | Moderate |
| **23** | **Boué et al. (2000)** | Low | Moderate | Low | Low | Moderate | Low | Low | Moderate |
| **24** | **Brady et al. (2020)** | Moderate | Low | Low | Low | Moderate | Low | Low | Moderate |
| **25** | **Breneman et al. (2013)** | Moderate | Low | Low | Low | Moderate | Low | Low | Moderate |
| **26** | **Brennan et al. (2016)** | Moderate | Low | Low | Moderate | Moderate | Low | Low | Moderate |
| **27** | **Brennan et al. (2018)** | Moderate | Low | Low | Moderate | Moderate | Low | Low | Moderate |
| **28** | **Brennan et al. (2020)** | Moderate | Low | Low | Moderate | Moderate | Low | Low | Moderate |
| **29** | **Buszek et al. (2019)** | Moderate | Low | Low | Moderate | Moderate | Low | Low | Moderate |
| **30** | **Byers et al. (2021)** | Moderate | Moderate | Low | Low | Low | Low | Low | Moderate |
| **31** | **Carli et al. (2005)** | Moderate | Low | Low | Moderate | Moderate | Low | Low | Moderate |
| **32** | **Casanova et al. (2000)** | Moderate | Low | Low | Moderate | Low | Low | Low | Moderate |
| **33** | **Casanova et al. (2005)** | Moderate | Low | Low | Moderate | Low | Low | Low | Moderate |
| **34** | **Casanova et al. (2007)** | Serious | Moderate | Moderate | Moderate | Moderate | Low | Low | Moderate |
| **35** | **Casanova et al. (2020)** | Moderate | Low | Low | Moderate | Low | Low | Low | Moderate |
| **36** | **Casey et al. (2014)** | Moderate | Low | Low | Moderate | Low | Low | Low | Moderate |
| **37** | **Cecchetto et al. (2001)** | Moderate | Low | Moderate | Moderate | Moderate | Low | Moderate | Moderate |
| **38** | **Cecchetto et al. (2003)** | Moderate | Low | Low | Moderate | Low | Low | Low | Moderate |
| **39** | **Cecchetto et al. (2005)** | Moderate | Low | Low | Moderate | Low | Low | Low | Moderate |
| **40** | **Cecchetto et al. (2008)** | Moderate | Low | Low | Moderate | Low | Low | Low | Moderate |
| **41** | **Chaix et al. (2023)** | Moderate | Low | Low | Moderate | Moderate | Low | Moderate | Moderate |
| **42** | **Chrisholm et al. (2011)** | Moderate | Low | Low | Moderate | Moderate | Low | Low | Moderate |
| **43** | **Chui et al. (2005)** | Moderate | Low | Moderate | Moderate | Low | Low | Moderate | Moderate |
| **44** | **Chun et al. (2005)** | Moderate | Moderate | Low | Low | Moderate | Moderate | Low | Moderate |
| **45** | **Coene et al. (1992)** | Moderate | Moderate | Low | Low | Moderate | Moderate | Moderate | Moderate |
| **46** | **Cole et al. (1993)** | Low | Low | Low | Low | Moderate | Low | Moderate | Low |
| **47** | **Company et al. (2009)** | Moderate | Moderate | Low | Low | Moderate | Moderate | Moderate | Moderate |
| **48** | **Corpron et al. (1996)** | Serious | Moderate | Moderate | Serious | Moderate | Low | Moderate | Moderate |
| **49** | **Cotter et al. (2011)** | Moderate | Low | Low | Low | Moderate | Low | Moderate | Moderate |
| **50** | **Cypriano et al. (2004)** | Moderate | Low | Low | Moderate | Moderate | Low | Moderate | Moderate |
| **51** | **Daigeler et al. (2008)** | Moderate | Low | Low | Moderate | Low | Low | Low | Moderate |
| **52** | **D’Angelo et al. (2010)** | Moderate | Moderate | Low | Low | Moderate | Low | Moderate | Moderate |
| **53** | **Dang et al. (2013)** | Moderate | Low | Moderate | Low | Moderate | Low | Moderate | Moderate |
| **54** | **Dantonello et al. (2008)** | Moderate | Low | Moderate | Moderate | Moderate | Low | Low | Moderate |
| **55** | **Dantonello et al. (2012)** | Moderate | Low | Moderate | Moderate | Moderate | Low | Low | Moderate |
| **56** | **Dantonello et al. (2018)** | Moderate | Low | Low | Moderate | Low | Low | Low | Moderate |
| **57** | **De Corti et al. (2006)** | Moderate | Low | Low | Moderate | Low | Low | Low | Moderate |
| **58** | **de Vries et al. (1989)** | Serious | Moderate | Low | Moderate | Serious | Moderate | Moderate | Moderate |
| **59** | **de Wardin et al. (2023)** | Moderate | Low | Low | Moderate | Low | Low | Low | Moderate |
| **60** | **Deyrup et al. (2009)** | Moderate | Moderate | Low | Low | Moderate | Low | Low | Moderate |
| **61** | **Deyrup et al. (2011)** | Moderate | Moderate | Low | Low | Moderate | Low | Low | Moderate |
| **62** | **Diaconescu et al. (2013)** | Moderate | Low | Low | Moderate | Moderate | Low | Low | Moderate |
| **63** | **Donaldson et al. (2001)** | Low | Low | Low | Moderate | Low | Low | Low | Low |
| **64** | **Dong et al. (2017)** | Moderate | Low | Low | Low | Moderate | Low | Low | Moderate |
| **65** | **Dong et al. (2021)** | Moderate | Low | Low | Low | Moderate | Low | Low | Moderate |
| **66** | **El-Gohary et al. (2020)** | Moderate | Low | Low | Low | Moderate | Low | Low | Moderate |
| **67** | **Elmanzalawy et al. (2019)** | Moderate | Low | Low | Low | Moderate | Low | Low | Moderate |
| **68** | **Emir et al. (2016)** | Moderate | Low | Low | Moderate | Serious | Low | Low | Moderate |
| **69** | **Enault et al. (2022)** | Moderate | Low | Low | Moderate | Serious | Low | Low | Moderate |
| **70** | **Eugene et al. (2012)** | Moderate | Low | Low | Moderate | Serious | Low | Low | Moderate |
| **71** | **Federico et al. (2012)** | Moderate | Low | Low | Low | Moderate | Moderate | Low | Moderate |
| **72** | **Federico et al. (2013)** | Serious | Low | Moderate | Moderate | Moderate | Moderate | Low | Moderate |
| **73** | **Fei-Zhang et al. (2023)** | Moderate | Low | Serious | Low | Moderate | Low | Low | Moderate |
| **74** | **Ferrari et al. (2001)** | Serious | Low | Moderate | Moderate | Moderate | Low | Low | Moderate |
| **75** | **Ferrari et al. (2002)** | Serious | Low | Moderate | Moderate | Moderate | Low | Low | Moderate |
| **76** | **Ferrari et al. (2002)** | Serious | Moderate | Moderate | Moderate | Low | Low | Low | Moderate |
| **77** | **Ferrari et al. (2003)** | Serious | Moderate | Moderate | Moderate | Low | Low | Low | Moderate |
| **78** | **Ferrari et al. (2005)** | Serious | Moderate | Moderate | Moderate | Low | Low | Low | Moderate |
| **79** | **Ferrari et al. (2008)** | Serious | Moderate | Moderate | Moderate | Low | Low | Low | Moderate |
| **80** | **Ferrari et al. (2014)** | Moderate | Low | Low | Moderate | Moderate | Low | Low | Moderate |
| **81** | **Ferrari et al. (2021)** | Serious | Moderate | Moderate | Moderate | Low | Low | Low | Moderate |
| **82** | **Ferrari et al. (2022)** | Serious | Moderate | Low | Moderate | Moderate | Low | Low | Moderate |
| **83** | **Ferrari et al. (2023)** | Serious | Moderate | Low | Moderate | Moderate | Low | Low | Moderate |
| **84** | **Flores et al. (2018)** | Moderate | Moderate | Low | Moderate | Moderate | Low | Low | Moderate |
| **85** | **Friesenbichler et al. (2021)** | Moderate | Low | Low | Low | Moderate | Moderate | Low | Moderate |
| **86** | **Fuchs et al. (2014)** | Moderate | Moderate | Low | Moderate | Moderate | Low | Low | Moderate |
| **87** | **Fuchs et al. (2018)** | Moderate | Low | Low | Moderate | Moderate | Low | Low | Moderate |
| **88** | **Fujiwara et al. (2022)** | Moderate | Moderate | Low | Moderate | Moderate | Low | Low | Moderate |
| **89** | **Furtwängler et al. (2013)** | Moderate | Low | Low | Moderate | Moderate | Low | Low | Moderate |
| **90** | **Genevois et al. (2023)** | Low | Low | Low | Moderate | Moderate | Moderate | Low | Moderate |
| **91** | **Genevois et al. (2024)** | Moderate | Low | Low | Moderate | Moderate | Low | Low | Moderate |
| **92** | **Giraudo et al. (2023)** | Moderate | Low | Moderate | Low | Low | Moderate | Low | Moderate |
| **93** | **Glosli et al. (2021)** | Moderate | Low | Low | Moderate | Moderate | Low | Low | Moderate |
| **94** | **Guérin et al. (2019)** | Moderate | Low | Low | Moderate | Moderate | Low | Low | Moderate |
| **95** | **Guo et al. (2021)** | Moderate | Moderate | Low | Moderate | Moderate | Low | Low | Moderate |
| **96** | **Hadley et al. (2010)** | Moderate | Moderate | Moderate | Moderate | Moderate | Moderate | Moderate | Moderate |
| **97** | **Hamilton et al. (2018)** | Moderate | Low | Low | Moderate | Moderate | Low | Low | Moderate |
| **98** | **Harrison et al. (2021)** | Low | Moderate | Low | Moderate | Moderate | Low | Low | Low |
| **99** | **Harrison et al. (2024)** | Moderate | Low | Moderate | Low | Low | Moderate | Low | Moderate |
| **100** | **Hayes-Jordan et al. (2008)** | Moderate | Low | Low | Moderate | Moderate | Low | Low | Moderate |
| **101** | **Hays et al. (1982)** | Moderate | Low | Low | Moderate | Low | Low | Low | Moderate |
| **102** | **Heinz et al. (2023)** | Moderate | Moderate | Low | Moderate | Moderate | Low | Low | Moderate |
| **103** | **Heinz et al. (2023)** | Moderate | Low | Low | Moderate | Moderate | Low | Low | Moderate |
| **104** | **Heske et al. (2020)** | Moderate | Moderate | Low | Low | Moderate | Low | Low | Moderate |
| **105** | **Hibbitts et al. (2019)** | Low | Low | Low | Low | Moderate | Low | Low | Low |
| **106** | **Hicks et al. (2002)** | Moderate | Moderate | Low | Low | Moderate | Low | Moderate | Moderate |
| **107** | **Hill et al. (2002)** | Moderate | Moderate | Low | Moderate | Serious | Low | Moderate | Moderate |
| **108** | **Hong et al. (2015)** | Moderate | Low | Low | Low | Moderate | Low | Low | Moderate |
| **109** | **Huh et al. (2011)** | Moderate | Low | Moderate | Moderate | Moderate | Low | Moderate | Moderate |
| **110** | **Jenney et al. (2014)** | Moderate | Low | Low | Moderate | Low | Moderate | Low | Moderate |
| **111** | **Jiang et al. (2018)** | Serious | Moderate | Low | Low | Serious | Low | Moderate | Serious |
| **112** | **Kayton et al. (2006)** | Moderate | Low | Low | Moderate | Moderate | Low | Low | Moderate |
| **113** | **Kayton et al. (2007)** | Moderate | Moderate | Low | Low | Moderate | Low | Low | Moderate |
| **114** | **Kayton et al. (2023)** | Serious | Low | Low | Low | Moderate | Low | Moderate | Serious |
| **115** | **Kim et al. (2017)** | Serious | Moderate | Moderate | Low | Moderate | Moderate | Serious | Serious |
| **116** | **Koivusalo et al. (2019)** | Moderate | Serious | Low | Moderate | Moderate | Moderate | Serious | Serious |
| **117** | **Komasara et al. (2016)** | Moderate | Low | Moderate | Moderate | Moderate | Low | Moderate | Moderate |
| **118** | **Koscielniak et al. (2021)** | Moderate | Low | Low | Low | Moderate | Low | Low | Moderate |
| **119** | **Koscielniak et al. (2023)** | Moderate | Low | Low | Moderate | Moderate | Low | Low | Moderate |
| **120** | **Krewer et al. (2020)** | Moderate | Low | Low | Moderate | Moderate | Low | Low | Moderate |
| **121** | **Kushner et al. (1996)** | Moderate | Moderate | Low | Low | Moderate | Low | Low | Moderate |
| **122** | **La et al. (2011)** | Moderate | Low | Low | Moderate | Moderate | Moderate | Low | Moderate |
| **123** | **Lack et al. (1986)** | Moderate | Moderate | Low | Low | Moderate | Low | Moderate | Moderate |
| **124** | **Lae et al. (2002)** | Moderate | Moderate | Low | Low | Moderate | Low | Low | Moderate |
| **125** | **Lak et al. (2021)** | Moderate | Low | Low | Moderate | Moderate | Low | Moderate | Moderate |
| **126** | **Lal et al. (2005)** | Moderate | Moderate | Low | Moderate | Moderate | Low | Low | Moderate |
| **127** | **Lautz et al. (2020)** | Low | Moderate | Low | Moderate | Low | Moderate | Low | Moderate |
| **128** | **Lautz et al. (2023)** | Low | Moderate | Low | Moderate | Low | Moderate | Low | Moderate |
| **129** | **La Quaglia et al. (1990)** | Moderate | Moderate | Low | Low | Moderate | Low | Moderate | Moderate |
| **130** | **La Quaglia et al. (1990)** | Moderate | Low | Low | Low | Moderate | Low | Moderate | Moderate |
| **131** | **Lawrence et al. (1987)** | Moderate | Moderate | Low | Low | Moderate | Low | Moderate | Moderate |
| **132** | **Lee et al. (2010)** | Moderate | Low | Low | Low | Moderate | Low | Low | Moderate |
| **133** | **Li et al. (2021)** | Moderate | Moderate | Low | Moderate | Serious | Low | Low | Moderate |
| **134** | **Liu et al. (2020)** | Moderate | Moderate | Low | Moderate | Serious | Low | Moderate | Moderate |
| **135** | **Livellara et al. (2021)** | Moderate | Low | Low | Low | Moderate | Low | Low | Moderate |
| **136** | **Ludmir et al. (2018)** | Low | Moderate | Low | Low | Moderate | Moderate | Low | Moderate |
| **137** | **Machavoine et al. (2022)** | Moderate | Low | Low | Moderate | Moderate | Low | Low | Moderate |
| **138** | **Madigan et al. (2007)** | Moderate | Moderate | Low | Low | Moderate | Low | Low | Moderate |
| **139** | **Malempati et al. (2011)** | Moderate | Low | Low | Moderate | Moderate | Low | Low | Moderate |
| **140** | **Malempati et al. (2019)** | Moderate | Low | Low | Moderate | Low | Low | Low | Moderate |
| **141** | **Mandell et al. (1990)** | Moderate | Moderate | Low | Low | Moderate | Low | Moderate | Moderate |
| **142** | **Marina et al. (1989)** | Moderate | Moderate | Low | Moderate | Serious | Low | Moderate | Moderate |
| **143** | **Martelli et al. (1999)** | Moderate | Low | Low | Moderate | Low | Low | Low | Moderate |
| **144** | **Martynov et al. (2024)** | Low | Low | Low | Low | Low | Moderate | Low | Low |
| **145** | **Mazzoleni et al. (2005)** | Moderate | Low | Moderate | Moderate | Low | Low | Low | Moderate |
| **146** | **McDowell et al. (2010)** | Moderate | Low | Moderate | Moderate | Low | Low | Low | Moderate |
| **147** | **Meister et al. (2020)** | Moderate | Low | Moderate | Moderate | Low | Low | Low | Moderate |
| **148** | **Mercolini et al. (2021)** | Moderate | Low | Moderate | Low | Moderate | Low | Low | Moderate |
| **149** | **Mercolini et al. (2022)** | Moderate | Low | Low | Moderate | Moderate | Low | Low | Moderate |
| **150** | **Metts et al. (2023)** | Moderate | Low | Moderate | Low | Moderate | Low | Low | Moderate |
| **151** | **Meza et al. (2006)** | Moderate | Low | Low | Low | Moderate | Low | Moderate | Moderate |
| **152** | **Minard-Colin et al. (2021)** | Serious | Moderate | Moderate | Low | Moderate | Low | Low | Moderate |
| **153** | **Munnikhuysen et al. (2023)** | Moderate | Low | Low | Low | Moderate | Low | Low | Moderate |
| **154** | **Murawski et al. (2019)** | Moderate | Low | Moderate | Moderate | Low | Low | Moderate | Moderate |
| **155** | **Murugan et al. (2018)** | Moderate | Moderate | Low | Low | Moderate | Low | Low | Moderate |
| **156** | **Neville et al. (2000)** | Moderate | Low | Low | Low | Moderate | Low | Low | Moderate |
| **157** | **Newman et al. (1983)** | Moderate | Moderate | Moderate | Moderate | Serious | Moderate | Serious | Moderate |
| **158** | **Oberlin et al. (2008)** | Moderate | Low | Moderate | Low | Moderate | Low | Low | Moderate |
| **159** | **Oberlin et al. (2015)** | Moderate | Moderate | Low | Moderate | Moderate | Low | Moderate | Moderate |
| **160** | **Okamara et al. (2006)** | Serious | Moderate | Moderate | Low | Moderate | Low | Moderate | Serious |
| **161** | **Olibier-Gougenheim et al. (2021)** | Serious | Moderate | Low | Low | Moderate | Low | Moderate | Moderate |
| **162** | **Orbach et al. (2013)** | Moderate | Low | Low | Moderate | Moderate | Low | Low | Moderate |
| **163** | **Orbach et al. (2016)** | Moderate | Low | Low | Low | Moderate | Moderate | Low | Low |
| **164** | **Orbach et al. (2022)** | Moderate | Low | Low | Moderate | Moderate | Low | Low | Moderate |
| **165** | **Orsatti et al. (2020)** | Moderate | Low | Low | Moderate | Moderate | Moderate | Low | Moderate |
| **166** | **Parasuraman et al. (1999)** | Moderate | Low | Low | Moderate | Moderate | Low | Low | Moderate |
| **167** | **Parida et al. (2012)** | Moderate | Moderate | Low | Moderate | Moderate | Moderate | Moderate | Moderate |
| **168** | **Parida et al. (2013)** | Moderate | Low | Moderate | Moderate | Moderate | Low | Moderate | Moderate |
| **169** | **Peng et al. (2021)** | Moderate | Low | Moderate | Moderate | Moderate | Low | Moderate | Moderate |
| **170** | **Perruccio et al. (2018)** | Low | Moderate | Low | Low | Moderate | Low | Low | Moderate |
| **171** | **Pierobon et al. (2018)** | Moderate | Low | Low | Moderate | Moderate | Low | Low | Moderate |
| **172** | **Pondrom et al. (2020)** | Low | Moderate | Low | Low | Moderate | Low | Low | Moderate |
| **173** | **Portera et al. (2001)** | Moderate | Moderate | Low | Moderate | Moderate | Low | Low | Moderate |
| **174** | **Qualman et al. (2008)** | Moderate | Low | Low | Low | Moderate | Low | Low | Moderate |
| **175** | **Qureshi et al. (2013)** | Moderate | Moderate | Low | Low | Low | Low | Low | Moderate |
| **176** | **Raney et al. (1988)** | Moderate | Moderate | Moderate | Moderate | Moderate | Moderate | Serious | Moderate |
| **177** | **Raney et al. (2002)** | Moderate | Low | Low | Low | Moderate | Low | Low | Moderate |
| **178** | **Raney et al. (2008)** | Moderate | Low | Low | Moderate | Low | Low | Low | Moderate |
| **179** | **Raney et al. (2008)** | Moderate | Moderate | Low | Moderate | Low | Low | Low | Moderate |
| **180** | **Raney et al. (2012)** | Moderate | Moderate | Low | Low | Low | Low | Low | Moderate |
| **181** | **Raney et al. (2013)** | Moderate | Low | Low | Low | Low | Moderate | Low | Moderate |
| **182** | **Réguerre et al. (2012)** | Moderate | Low | Low | Low | Moderate | Low | Low | Moderate |
| **183** | **Ricard et al. (2011)** | Moderate | Moderate | Low | Low | Moderate | Low | Low | Moderate |
| **184** | **Rodary et el. (1991)** | Low | Moderate | Low | Low | Low | Moderate | Low | Moderate |
| **185** | **Rodeberg et al. (2010)** | Moderate | Low | Low | Low | Moderate | Low | Low | Moderate |
| **186** | **Rodeberg et al. (2011)** | Moderate | Low | Low | Low | Moderate | Low | Low | Moderate |
| **187** | **Rodeberg et al. (2011)** | Moderate | Low | Low | Low | Moderate | Low | Low | Moderate |
| **188** | **Rodriguez-Galindo et al. (2000)** | Moderate | Low | Low | Moderate | Low | Low | Low | Moderate |
| **189** | **Rogers et al. (2017)** | Moderate | Low | Low | Low | Moderate | Low | Low | Moderate |
| **190** | **Rogers et al. (2020)** | Moderate | Low | Low | Low | Moderate | Low | Low | Moderate |
| **191** | **Rogers et al. (2022)** | Moderate | Low | Low | Moderate | Moderate | Low | Low | Moderate |
| **192** | **Rossetti et al. (2021)** | Moderate | Low | Low | Low | Low | Low | Low | Low |
| **193** | **Routh et al. (2021)** | Moderate | Low | Low | Moderate | Moderate | Low | Low | Moderate |
| **194** | **Ruymann et al. (1984)** | Moderate | Moderate | Moderate | Moderate | Moderate | Moderate | Serious | Moderate |
| **195** | **Ruymann et al. (1985)** | Serious | Moderate | Low | Moderate | Serious | Moderate | Moderate | Moderate |
| **196** | **Saenz et al. (1997)** | Moderate | Low | Low | Moderate | Moderate | Low | Low | Moderate |
| **197** | **Saito et al. (2017)** | Moderate | Moderate | Low | Moderate | Low | Low | Low | Moderate |
| **198** | **Sari et al. (2009)** | Moderate | Moderate | Low | Low | Moderate | Low | Low | Moderate |
| **199** | **Scheer et al. (2016)** | Moderate | Low | Low | Low | Moderate | Moderate | Low | Moderate |
| **200** | **Scheer et al. (2016)** | Moderate | Low | Low | Moderate | Moderate | Low | Low | Moderate |
| **201** | **Scheer et al. (2020)** | Moderate | Low | Low | Moderate | Moderate | Low | Low | Moderate |
| **202** | **Scheer et al. (2021)** | Moderate | Low | Low | Moderate | Moderate | Low | Low | Moderate |
| **203** | **Schloemer et al. (2023)** | Moderate | Low | Low | Moderate | Moderate | Low | Low | Moderate |
| **204** | **Schoot et al. (2022)** | Moderate | Low | Moderate | Moderate | Moderate | Low | Low | Moderate |
| **205** | **Schwarz et al. (1998)** | Moderate | Moderate | Low | Moderate | Moderate | Low | Low | Moderate |
| **206** | **Seitz et al. (2011)** | Moderate | Low | Moderate | Moderate | Moderate | Low | Low | Moderate |
| **207** | **Seitz et al. (2016)** | Moderate | Low | Moderate | Moderate | Moderate | Low | Low | Moderate |
| **208** | **Seitz et al. (2018)** | Moderate | Low | Moderate | Moderate | Moderate | Low | Low | Moderate |
| **209** | **Sercarz et al. (1995)** | Moderate | Low | Moderate | Moderate | Moderate | Low | Low | Moderate |
| **210** | **Shenoy et al. (2021)** | Moderate | Low | Moderate | Low | Moderate | Low | Low | Moderate |
| **211** | **Shi et al. (2016)** | Moderate | Low | Moderate | Moderate | Low | Low | Moderate | Moderate |
| **212** | **Siddiqui et al. (2019)** | Moderate | Low | Moderate | Moderate | Moderate | Low | Low | Moderate |
| **213** | **Slater et al. (2022)** | Low | Moderate | Moderate | Moderate | Moderate | Low | Low | Moderate |
| **214** | **Sobieraj et al. (2024)** | Moderate | Low | Low | Moderate | Low | Low | Low | Moderate |
| **215** | **Sparber-Sauer et al. (2018)** | Moderate | Low | Moderate | Moderate | Moderate | Low | Low | Moderate |
| **216** | **Sparber-Sauer et al. (2019)** | Moderate | Low | Low | Moderate | Low | Low | Low | Moderate |
| **217** | **Sparber-Sauer et al. (2019)** | Moderate | Low | Low | Moderate | Low | Low | Low | Moderate |
| **218** | **Sparber-Sauer et al. (2020)** | Moderate | Low | Moderate | Moderate | Low | Low | Low | Moderate |
| **219** | **Sparber-Sauer et al. (2021)** | Moderate | Low | Low | Moderate | Low | Low | Low | Moderate |
| **220** | **Sparber-Sauer et al. (2021)** | Moderate | Low | Moderate | Moderate | Low | Low | Low | Moderate |
| **221** | **Sparber-Sauer et al. (2023)** | Moderate | Moderate | Moderate | Moderate | Moderate | Low | Low | Moderate |
| **222** | **Sparber-Sauer et al. (2024)** | Low | Low | Moderate | Moderate | Moderate | Low | Low | Moderate |
| **223** | **Spunt et al. (2019)** | Moderate | Low | Moderate | Moderate | Moderate | Moderate | Moderate | Moderate |
| **224** | **Spunt et al. (2000)** | Moderate | Moderate | Moderate | Moderate | Moderate | Moderate | Moderate | Moderate |
| **225** | **Spunt et al. (2020)** | Low | Moderate | Low | Moderate | Moderate | Low | Low | Moderate |
| **226** | **Stanelle et al. (2013)** | Low | Moderate | Low | Moderate | Moderate | Low | Low | Moderate |
| **227** | **Stein et al. (2013)** | Moderate | Moderate | Low | Moderate | Moderate | Low | Low | Moderate |
| **228** | **Sung et al. (2004)** | Moderate | Moderate | Low | Moderate | Low | Low | Low | Moderate |
| **229** | **Tagarelli et al. (2012)** | Moderate | Moderate | Low | Low | Moderate | Low | Low | Moderate |
| **230** | **Talenti et al. (2021)** | Moderate | Moderate | Low | Low | Moderate | Low | Low | Moderate |
| **231** | **Tan et al. (2022)** | Moderate | Low | Low | Low | Moderate | Low | Low | Moderate |
| **232** | **Tang et al. (2018)** | Moderate | Moderate | Low | Moderate | Serious | Low | Moderate | Moderate |
| **233** | **Tarek et al. (2020)** | Moderate | Moderate | Low | Low | Moderate | Moderate | Low | Moderate |
| **234** | **Thomas et al. (2013)** | Moderate | Moderate | Low | Low | Serious | Moderate | Low | Moderate |
| **235** | **Urla et al. (2019)** | Moderate | Low | Low | Moderate | Moderate | Low | Low | Moderate |
| **236** | **Vaarwerk et al. (2018)** | Moderate | Low | Low | Moderate | Moderate | Low | Low | Moderate |
| **237** | **Vaarwerk et al. (2020)** | Moderate | Low | Moderate | Moderate | Moderate | Low | Low | Moderate |
| **238** | **van Noeseal et al. (2019)** | Moderate | Low | Low | Low | Moderate | Low | Low | Moderate |
| **239** | **van Scheltinga et al. (2014)** | Moderate | Moderate | Low | Moderate | Moderate | Low | Low | Moderate |
| **240** | **van Scheltinga et al. (2020)** | Moderate | Low | Low | Moderate | Moderate | Low | Low | Moderate |
| **241** | **van Scheltinga et al. (2022)** | Moderate | Low | Low | Moderate | Moderate | Low | Low | Moderate |
| **242** | **Vasquez et al. (2023)** | Moderate | Low | Low | Low | Moderate | Low | Low | Moderate |
| **243** | **Venkatramani et al. (2014)** | Moderate | Moderate | Low | Low | Moderate | Low | Low | Moderate |
| **244** | **Venkatramani et al. (2021)** | Moderate | Low | Low | Low | Moderate | Low | Low | Moderate |
| **245** | **Völker et al. (2007)** | Moderate | Low | Low | Low | Low | Low | Low | Moderate |
| **246** | **Wagner et al. (2016)** | Moderate | Low | Low | Low | Moderate | Low | Low | Moderate |
| **247** | **Walterhouse et al. (2001)** | Moderate | Low | Low | Low | Moderate | Low | Low | Moderate |
| **248** | **Walterhouse et al. (2011)** | Low | Low | Low | Low | Low | Low | Low | Low |
| **249** | **Walterhouse et al. (2014)** | Low | Moderate | Low | Low | Moderate | Low | Low | Moderate |
| **250** | **Watson et al. (2017)** | Moderate | Moderate | Low | Moderate | Moderate | Low | Low | Moderate |
| **251** | **Waxweiler et al. (2015)** | Moderate | Moderate | Low | Moderate | Moderate | Low | Moderate | Moderate |
| **252** | **Weller et al. (2021)** | Moderate | Moderate | Low | Low | Moderate | Low | Low | Moderate |
| **253** | **Welmart et al. (2021)** | Moderate | Moderate | Low | Low | Moderate | Low | Low | Moderate |
| **254** | **Wiener et al. (1994)** | Moderate | Moderate | Low | Low | Moderate | Low | Low | Moderate |
| **255** | **Xiao et al. (2016)** | Moderate | Moderate | Low | Low | Moderate | Low | Moderate | Moderate |
| **256** | **Xie et al. (2022)** | Moderate | Moderate | Low | Moderate | Serious | Moderate | Low | Moderate |
| **257** | **Zekri et al. (2014)** | Low | Low | Low | Unclear | Unclear | Low | Low | Low |
| **258** | **Zhang et al. (2023)** | Moderate | Low | Low | Unclear | Moderate | Low | Low | Moderate |
| **259** | **Zhang et al. (2024)** | Moderate | Low | Low | Low | Moderate | Low | Low | Moderate |
| **260** | **Zhang et al. (2024)** | Moderate | Low | Low | Unclear | Moderate | Low | Low | Moderate |
| **261** | **Zhanghuang et al. (2022)** | Moderate | Low | Low | Moderate | Moderate | Low | Low | Moderate |
| **262** | **Zhu et al. (2020)** | Moderate | Low | Low | Unclear | Moderate | Low | Low | Moderate |
| **263** | **Zin et al. (2014)** | Low | Low | Moderate | Low | Low | Low | Low | Moderate |
