# Supplementary material for: Prevalence of locoregional and distant lymph node metastases in children and adolescents/young adults with soft tissue sarcomas: a Bayesian meta-analysis of proportions
Source: eClinicalMedicine. 2025 Aug 7;87:103390. doi: 10.1016/j.eclinm.2025.103390 (PMC12355419; doi:10.1016/j.eclinm.2025.103390)
Supplement: Supplementary Table S5 [file mmc7.docx]

**RMS**

- all patients: 41547
- **LN all patients**
  - Posterior Mean Proportion of Events: 0.228
    - 95% Credible Interval for Proportion: 0.2027 - 0.2554
  - Estimated Percentage of Events: 22.8 %
    - 95% Credible Interval for Percentage: 20.27 % - 25.54 %
  - Posterior Mean of Between-Study Standard Deviation (τ): 0.8604
    - 95% Credible Interval for τ: 0.7443 - 0.9814
  - Sensitivity Analysis

*Mean* *CI_Lower* *CI_Upper*

Main Analysis (All Studies) 0.2280021 0.2026722 0.2554461

Overlap Exclusion 0.2083875 0.1740701 0.2473874

Bias Exclusion 0.2250221 0.1995933 0.2526205

Combined Exclusion 0.2070326 0.1726765 0.2461079

**RMS Locations**

*Location Number of Studies Number of patients*

Abdomen, Pelvis 3 68

Biliary tract / Liver 8 117

Body wall, Trunk 7 194

Extremity 23 1984

Gastrointestinal tract 1 7 → now included in the visceral

GU - Bladder-Prostate 18 1187

GU - Non-BP 10 816

Head and neck, non-parameningeal 10 858

Head and neck, NS 6 203

Head and neck, parameningeal 11 1920

Orbit 7 552

Paratesticular 13 2092

Perineal / Perianal 12 298

Retroperitoneal 4 94

Thorax 6 252

Visceral 2 (3) 11 (18)

============================================

Bayesian Meta-analysis for Location: Head and neck, non-parameningeal

============================================

Posterior Mean Proportion of Events: 0.2585

95% Credible Interval for Proportion: 0.1675 - 0.3758

Estimated Percentage of Events: 25.85 %

95% Credible Interval for Percentage: 16.75 % - 37.58 %

============================================

Bayesian Meta-analysis for Location: Extremity

============================================

Posterior Mean Proportion of Events: 0.2808

95% Credible Interval for Proportion: 0.2096 - 0.3631

Estimated Percentage of Events: 28.08 %

95% Credible Interval for Percentage: 20.96 % - 36.31 %

============================================

Bayesian Meta-analysis for Location: GU - Bladder-Prostate

============================================

Posterior Mean Proportion of Events: 0.1439

95% Credible Interval for Proportion: 0.0968 - 0.2108

Estimated Percentage of Events: 14.39 %

95% Credible Interval for Percentage: 9.68 % - 21.08 %

============================================

Bayesian Meta-analysis for Location: Biliary tract / Liver

============================================

Posterior Mean Proportion of Events: 0.2045

95% Credible Interval for Proportion: 0.1251 - 0.3147

Estimated Percentage of Events: 20.45 %

95% Credible Interval for Percentage: 12.51 % - 31.47 %

============================================

Bayesian Meta-analysis for Location: Orbit

============================================

Posterior Mean Proportion of Events: 0.013

95% Credible Interval for Proportion: 0.0045 - 0.038

Estimated Percentage of Events: 1.3 %

95% Credible Interval for Percentage: 0.45 % - 3.8 %

============================================

Bayesian Meta-analysis for Location: Head and neck, parameningeal

============================================

Posterior Mean Proportion of Events: 0.2548

95% Credible Interval for Proportion: 0.1607 - 0.3824

Estimated Percentage of Events: 25.48 %

95% Credible Interval for Percentage: 16.07 % - 38.24 %

============================================

Bayesian Meta-analysis for Location: GU - Non-BP

============================================

Posterior Mean Proportion of Events: 0.099

95% Credible Interval for Proportion: 0.0482 - 0.1899

Estimated Percentage of Events: 9.9 %

95% Credible Interval for Percentage: 4.82 % - 18.99 %

============================================

Bayesian Meta-analysis for Location: Perineal / Perianal

============================================

Posterior Mean Proportion of Events: 0.4664

95% Credible Interval for Proportion: 0.3971 - 0.5371

Estimated Percentage of Events: 46.64 %

95% Credible Interval for Percentage: 39.71 % - 53.71 %

============================================

Bayesian Meta-analysis for Location: Body wall, Trunk

============================================

Posterior Mean Proportion of Events: 0.138

95% Credible Interval for Proportion: 0.0599 - 0.2734

Estimated Percentage of Events: 13.8 %

95% Credible Interval for Percentage: 5.99 % - 27.34 %

============================================

Bayesian Meta-analysis for Location: Paratesticular

============================================

Posterior Mean Proportion of Events: 0.1333

95% Credible Interval for Proportion: 0.1038 - 0.1705

Estimated Percentage of Events: 13.33 %

95% Credible Interval for Percentage: 10.38 % - 17.05 %

============================================

Bayesian Meta-analysis for Location: Head and neck, NS

============================================

Posterior Mean Proportion of Events: 0.1717

95% Credible Interval for Proportion: 0.0614 - 0.4064

Estimated Percentage of Events: 17.17 %

95% Credible Interval for Percentage: 6.14 % - 40.64 %

============================================

Bayesian Meta-analysis for Location: Thorax

============================================

Posterior Mean Proportion of Events: 0.1638

95% Credible Interval for Proportion: 0.0793 - 0.3055

Estimated Percentage of Events: 16.38 %

95% Credible Interval for Percentage: 7.93 % - 30.55 %

============================================

Bayesian Meta-analysis for Location: Retroperitoneal

============================================

Posterior Mean Proportion of Events: 0.2303

95% Credible Interval for Proportion: 0.085 - 0.4475

Estimated Percentage of Events: 23.03 %

95% Credible Interval for Percentage: 8.5 % - 44.75 %

============================================

Bayesian Meta-analysis for Location: Abdomen, Pelvis

============================================

Posterior Mean Proportion of Events: 0.2509

95% Credible Interval for Proportion: 0.0919 - 0.5325

Estimated Percentage of Events: 25.09 %

95% Credible Interval for Percentage: 9.19 % - 53.25 %

============================================

Bayesian Meta-analysis for Location: Visceral

============================================

Posterior Mean Proportion of Events: 0.1221

95% Credible Interval for Proportion: 0.0217 - 0.4655

Estimated Percentage of Events: 12.21 %

95% Credible Interval for Percentage: 2.17 % - 46.55 %

============================================

Bayesian Meta-analysis for Location: Head and neck, non-parameningeal

============================================

Posterior Mean of Between-Study Standard Deviation (τ): 0.7558

95% Credible Interval for τ: 0.3904 - 1.1941

============================================

Bayesian Meta-analysis for Location: Extremity

============================================

Posterior Mean of Between-Study Standard Deviation (τ): 0.7167

95% Credible Interval for τ: 0.3976 - 1.0792

============================================

Bayesian Meta-analysis for Location: GU - Bladder-Prostate

============================================

Posterior Mean of Between-Study Standard Deviation (τ): 0.7226

95% Credible Interval for τ: 0.3358 - 1.1764

============================================

Bayesian Meta-analysis for Location: Biliary tract / Liver

============================================

Posterior Mean of Between-Study Standard Deviation (τ): 0.3463

95% Credible Interval for τ: 0 - 0.8728

============================================

Bayesian Meta-analysis for Location: Orbit

============================================

Posterior Mean of Between-Study Standard Deviation (τ): 0.5468

95% Credible Interval for τ: 0 - 1.387

============================================

Bayesian Meta-analysis for Location: Head and neck, parameningeal

============================================

Posterior Mean of Between-Study Standard Deviation (τ): 0.8384

95% Credible Interval for τ: 0.4357 - 1.3163

============================================

Bayesian Meta-analysis for Location: GU - Non-BP

============================================

Posterior Mean of Between-Study Standard Deviation (τ): 0.9845

95% Credible Interval for τ: 0.4874 - 1.5678

============================================

Bayesian Meta-analysis for Location: Perineal / Perianal

============================================

Posterior Mean of Between-Study Standard Deviation (τ): 0.1794

95% Credible Interval for τ: 0 - 0.4885

============================================

Bayesian Meta-analysis for Location: Body wall, Trunk

============================================

Posterior Mean of Between-Study Standard Deviation (τ): 0.7796

95% Credible Interval for τ: 0 - 1.5014

============================================

Bayesian Meta-analysis for Location: Paratesticular

============================================

Posterior Mean of Between-Study Standard Deviation (τ): 0.4016

95% Credible Interval for τ: 0.1456 - 0.7045

============================================

Bayesian Meta-analysis for Location: Head and neck, NS

============================================

Posterior Mean of Between-Study Standard Deviation (τ): 0.9851

95% Credible Interval for τ: 0.1534 - 1.9603

============================================

Bayesian Meta-analysis for Location: Thorax

============================================

Posterior Mean of Between-Study Standard Deviation (τ): 0.7068

95% Credible Interval for τ: 0.1295 - 1.4059

============================================

Bayesian Meta-analysis for Location: Retroperitoneal

============================================

Posterior Mean of Between-Study Standard Deviation (τ): 0.5594

95% Credible Interval for τ: 0 - 1.4774

============================================

Bayesian Meta-analysis for Location: Abdomen, Pelvis

============================================

Posterior Mean of Between-Study Standard Deviation (τ): 0.5557

95% Credible Interval for τ: 0 - 1.4761

============================================

Bayesian Meta-analysis for Location: Visceral

============================================

Posterior Mean of Between-Study Standard Deviation (τ): 0.6591

95% Credible Interval for τ: 0 - 1.6595

Sensitivity Analysis for Location: Head and neck, non-parameningeal

Scenario Posterior_Mean_Proportion X95..CI..Proportion.

1 Main Analysis (All Studies) 0.2585418 [0.17, 0.38]

2 Overlap Exclusion 0.2028149 [0.12, 0.31]

3 Bias Exclusion 0.2585418 [0.17, 0.38]

4 Combined Exclusion 0.2028149 [0.12, 0.31]

Posterior_Mean_Tau X95..CI..Tau.

1 0.7557679 [0.39, 1.19]

2 0.5705440 [0.11, 1.14]

3 0.7557679 [0.39, 1.19]

4 0.5705440 [0.11, 1.14]

----------------------------------------

Sensitivity Analysis for Location: Extremity

Scenario Posterior_Mean_Proportion X95..CI..Proportion.

1 Main Analysis (All Studies) 0.2807892 [0.21, 0.36]

2 Overlap Exclusion 0.2948959 [0.21, 0.39]

3 Bias Exclusion 0.2770894 [0.2, 0.36]

4 Combined Exclusion 0.2907124 [0.2, 0.39]

Posterior_Mean_Tau X95..CI..Tau.

1 0.7166853 [0.4, 1.08]

2 0.7770741 [0.41, 1.2]

3 0.7322829 [0.4, 1.11]

4 0.7995701 [0.41, 1.24]

----------------------------------------

Sensitivity Analysis for Location: GU - Bladder-Prostate

Scenario Posterior_Mean_Proportion X95..CI..Proportion.

1 Main Analysis (All Studies) 0.1438872 [0.1, 0.21]

2 Overlap Exclusion 0.1517085 [0.08, 0.27]

3 Bias Exclusion 0.1438872 [0.1, 0.21]

4 Combined Exclusion 0.1517085 [0.08, 0.27]

Posterior_Mean_Tau X95..CI..Tau.

1 0.7225930 [0.34, 1.18]

2 0.9033982 [0.29, 1.66]

3 0.7225930 [0.34, 1.18]

4 0.9033982 [0.29, 1.66]

----------------------------------------

Sensitivity Analysis for Location: Biliary tract / Liver

Scenario Posterior_Mean_Proportion X95..CI..Proportion.

1 Main Analysis (All Studies) 0.2045105 [0.13, 0.31]

2 Overlap Exclusion 0.2239066 [0.12, 0.37]

3 Bias Exclusion 0.2045105 [0.13, 0.31]

4 Combined Exclusion 0.2239066 [0.12, 0.37]

Posterior_Mean_Tau X95..CI..Tau.

1 0.3462590 [0, 0.87]

2 0.4058197 [0, 1.04]

3 0.3462590 [0, 0.87]

4 0.4058197 [0, 1.04]

----------------------------------------

Sensitivity Analysis for Location: Orbit

Scenario Posterior_Mean_Proportion X95..CI..Proportion.

1 Main Analysis (All Studies) 0.01302016 [0, 0.04]

2 Overlap Exclusion 0.01674376 [0, 0.06]

3 Bias Exclusion 0.01302016 [0, 0.04]

4 Combined Exclusion 0.01674376 [0, 0.06]

Posterior_Mean_Tau X95..CI..Tau.

1 0.5467550 [0, 1.39]

2 0.6204677 [0, 1.54]

3 0.5467550 [0, 1.39]

4 0.6204677 [0, 1.54]

----------------------------------------

Sensitivity Analysis for Location: Head and neck, parameningeal

Scenario Posterior_Mean_Proportion X95..CI..Proportion.

1 Main Analysis (All Studies) 0.2547938 [0.16, 0.38]

2 Overlap Exclusion 0.2367912 [0.11, 0.43]

3 Bias Exclusion 0.2547938 [0.16, 0.38]

4 Combined Exclusion 0.2367912 [0.11, 0.43]

Posterior_Mean_Tau X95..CI..Tau.

1 0.8383758 [0.44, 1.32]

2 0.9126776 [0.4, 1.58]

3 0.8383758 [0.44, 1.32]

4 0.9126776 [0.4, 1.58]

----------------------------------------

Sensitivity Analysis for Location: GU - Non-BP

Scenario Posterior_Mean_Proportion X95..CI..Proportion.

1 Main Analysis (All Studies) 0.09897975 [0.05, 0.19]

2 Overlap Exclusion 0.11068699 [0.04, 0.25]

3 Bias Exclusion 0.09897975 [0.05, 0.19]

4 Combined Exclusion 0.11068699 [0.04, 0.25]

Posterior_Mean_Tau X95..CI..Tau.

1 0.9845053 [0.49, 1.57]

2 1.0641077 [0.45, 1.79]

3 0.9845053 [0.49, 1.57]

4 1.0641077 [0.45, 1.79]

----------------------------------------

Sensitivity Analysis for Location: Perineal / Perianal

Scenario Posterior_Mean_Proportion X95..CI..Proportion.

1 Main Analysis (All Studies) 0.4663884 [0.4, 0.54]

2 Overlap Exclusion 0.4348782 [0.28, 0.59]

3 Bias Exclusion 0.4657276 [0.39, 0.55]

4 Combined Exclusion 0.4348782 [0.28, 0.59]

Posterior_Mean_Tau X95..CI..Tau.

1 0.1793602 [0, 0.49]

2 0.4586593 [0, 1.16]

3 0.2176108 [0, 0.6]

4 0.4586593 [0, 1.16]

----------------------------------------

Sensitivity Analysis for Location: Body wall, Trunk

Scenario Posterior_Mean_Proportion X95..CI..Proportion.

1 Main Analysis (All Studies) 0.1380033 [0.06, 0.27]

2 Overlap Exclusion 0.1376205 [0.06, 0.28]

3 Bias Exclusion 0.1380033 [0.06, 0.27]

4 Combined Exclusion 0.1376205 [0.06, 0.28]

Posterior_Mean_Tau X95..CI..Tau.

1 0.7796314 [0, 1.5]

2 0.8191925 [0, 1.58]

3 0.7796314 [0, 1.5]

4 0.8191925 [0, 1.58]

----------------------------------------

Sensitivity Analysis for Location: Paratesticular

Scenario Posterior_Mean_Proportion X95..CI..Proportion.

1 Main Analysis (All Studies) 0.1333109 [0.1, 0.17]

2 Overlap Exclusion 0.1438097 [0.09, 0.22]

3 Bias Exclusion 0.1333109 [0.1, 0.17]

4 Combined Exclusion 0.1438097 [0.09, 0.22]

Posterior_Mean_Tau X95..CI..Tau.

1 0.4016313 [0.15, 0.7]

2 0.5555062 [0.16, 1.06]

3 0.4016313 [0.15, 0.7]

4 0.5555062 [0.16, 1.06]

----------------------------------------

Sensitivity Analysis for Location: Head and neck, NS

Scenario Posterior_Mean_Proportion X95..CI..Proportion.

1 Main Analysis (All Studies) 0.1716986 [0.06, 0.41]

2 Overlap Exclusion 0.0892466 [0.03, 0.22]

3 Bias Exclusion 0.1716986 [0.06, 0.41]

4 Combined Exclusion 0.0892466 [0.03, 0.22]

Posterior_Mean_Tau X95..CI..Tau.

1 0.9851145 [0.15, 1.96]

2 0.5712494 [0, 1.52]

3 0.9851145 [0.15, 1.96]

4 0.5712494 [0, 1.52]

----------------------------------------

Sensitivity Analysis for Location: Thorax

Scenario Posterior_Mean_Proportion X95..CI..Proportion.

1 Main Analysis (All Studies) 0.1637522 [0.08, 0.31]

2 Overlap Exclusion 0.1013018 [0.04, 0.27]

3 Bias Exclusion 0.1637522 [0.08, 0.31]

4 Combined Exclusion 0.1013018 [0.04, 0.27]

Posterior_Mean_Tau X95..CI..Tau.

1 0.7067551 [0.13, 1.41]

2 0.5731578 [0, 1.46]

3 0.7067551 [0.13, 1.41]

4 0.5731578 [0, 1.46]

----------------------------------------

Sensitivity Analysis for Location: Retroperitoneal

Scenario Posterior_Mean_Proportion X95..CI..Proportion.

1 Main Analysis (All Studies) 0.2303465 [0.08, 0.45]

2 Overlap Exclusion 0.2263767 [0.07, 0.46]

3 Bias Exclusion 0.2303465 [0.08, 0.45]

4 Combined Exclusion 0.2263767 [0.07, 0.46]

Posterior_Mean_Tau X95..CI..Tau.

1 0.5594445 [0, 1.48]

2 0.5994614 [0, 1.58]

3 0.5594445 [0, 1.48]

4 0.5994614 [0, 1.58]

----------------------------------------

Sensitivity Analysis for Location: Abdomen, Pelvis

Scenario Posterior_Mean_Proportion X95..CI..Proportion.

1 Main Analysis (All Studies) 0.2508941 [0.09, 0.53]

2 Overlap Exclusion 0.2513868 [0.08, 0.57]

3 Bias Exclusion 0.2508941 [0.09, 0.53]

4 Combined Exclusion 0.2513868 [0.08, 0.57]

Posterior_Mean_Tau X95..CI..Tau.

1 0.5556517 [0, 1.48]

2 0.5973583 [0, 1.59]

3 0.5556517 [0, 1.48]

4 0.5973583 [0, 1.59]

----------------------------------------

Sensitivity Analysis for Location: Visceral

Scenario Posterior_Mean_Proportion X95..CI..Proportion.

1 Main Analysis (All Studies) 0.1221199 [0.02, 0.47]

2 Overlap Exclusion NA <NA>

3 Bias Exclusion 0.1221199 [0.02, 0.47]

4 Combined Exclusion NA <NA>

Posterior_Mean_Tau X95..CI..Tau.

1 0.659115 [0, 1.66]

2 NA <NA>

3 0.659115 [0, 1.66]

4 NA <NA>

----------------------------------------

**RMS Histology**

*Histology Number of Studies Number of patients*

Alveolar 26 1565

Botryoid 7 50

Embryonal 34 4418

Leiomyomatous / Spnidle cell 4 32

Mixed type 3 13

Pleomorphic 2 2

Undifferentiated sarcoma 4 52

============================================

Bayesian Meta-analysis for Histology: Embryonal

============================================

Posterior Mean Proportion of Events: 0.1642

95% Credible Interval for Proportion: 0.1239 - 0.2149

Estimated Percentage of Events: 16.42 %

95% Credible Interval for Percentage: 12.39 % - 21.49 %

============================================

Bayesian Meta-analysis for Histology: Alveolar

============================================

Posterior Mean Proportion of Events: 0.3696

95% Credible Interval for Proportion: 0.276 - 0.4733

Estimated Percentage of Events: 36.96 %

95% Credible Interval for Percentage: 27.6 % - 47.33 %

============================================

Bayesian Meta-analysis for Histology: Leiomyomatous / Spindle cell

============================================

Posterior Mean Proportion of Events: 0.1276

95% Credible Interval for Proportion: 0.0338 - 0.3783

Estimated Percentage of Events: 12.76 %

95% Credible Interval for Percentage: 3.38 % - 37.83 %

============================================

Bayesian Meta-analysis for Histology: Mixed-type

============================================

Posterior Mean Proportion of Events: 0.2993

95% Credible Interval for Proportion: 0.0754 - 0.6881

Estimated Percentage of Events: 29.93 %

95% Credible Interval for Percentage: 7.54 % - 68.81 %

============================================

Bayesian Meta-analysis for Histology: Botryoid

============================================

Posterior Mean Proportion of Events: 0.1904

95% Credible Interval for Proportion: 0.0836 - 0.38

Estimated Percentage of Events: 19.04 %

95% Credible Interval for Percentage: 8.36 % - 38 %

============================================

Bayesian Meta-analysis for Histology: Undifferentiated sarcoma

============================================

Posterior Mean Proportion of Events: 0.2822

95% Credible Interval for Proportion: 0.1027 - 0.5604

Estimated Percentage of Events: 28.22 %

95% Credible Interval for Percentage: 10.27 % - 56.04 %

============================================

Bayesian Meta-analysis for Histology: Embryonal

============================================

Posterior Mean of Between-Study Standard Deviation (τ): 0.7082

95% Credible Interval for τ: 0.4329 - 1.016

============================================

Bayesian Meta-analysis for Histology: Alveolar

============================================

Posterior Mean of Between-Study Standard Deviation (τ): 0.8424

95% Credible Interval for τ: 0.4901 - 1.2338

============================================

Bayesian Meta-analysis for Histology: Leiomyomatous / Spindle cell

============================================

Posterior Mean of Between-Study Standard Deviation (τ): 0.5996

95% Credible Interval for τ: 0 - 1.5235

============================================

Bayesian Meta-analysis for Histology: Mixed-type

============================================

Posterior Mean of Between-Study Standard Deviation (τ): 0.6697

95% Credible Interval for τ: 0 - 1.6698

============================================

Bayesian Meta-analysis for Histology: Botryoid

============================================

Posterior Mean of Between-Study Standard Deviation (τ): 0.4602

95% Credible Interval for τ: 0 - 1.1925

============================================

Bayesian Meta-analysis for Histology: Undifferentiated sarcoma

============================================

Posterior Mean of Between-Study Standard Deviation (τ): 0.5665

95% Credible Interval for τ: 0 - 1.4637

Sensitivity Analysis for Histology: Embryonal

Scenario Posterior_Mean_Proportion X95..CI..Proportion.

1 Main Analysis (All Studies) 0.1642363 [0.12, 0.21]

2 Overlap Exclusion 0.1392684 [0.09, 0.21]

3 Bias Exclusion 0.1639050 [0.12, 0.21]

4 Combined Exclusion 0.1392684 [0.09, 0.21]

Posterior_Mean_Tau X95..CI..Tau.

1 0.7082339 [0.43, 1.02]

2 0.8239629 [0.42, 1.29]

3 0.7111387 [0.43, 1.02]

4 0.8239629 [0.42, 1.29]

----------------------------------------

Sensitivity Analysis for Histology: Alveolar

Scenario Posterior_Mean_Proportion X95..CI..Proportion.

1 Main Analysis (All Studies) 0.3696206 [0.28, 0.47]

2 Overlap Exclusion 0.3383043 [0.22, 0.49]

3 Bias Exclusion 0.3643065 [0.27, 0.47]

4 Combined Exclusion 0.3383043 [0.22, 0.49]

Posterior_Mean_Tau X95..CI..Tau.

1 0.8423691 [0.49, 1.23]

2 0.9139670 [0.4, 1.5]

3 0.8437129 [0.49, 1.24]

4 0.9139670 [0.4, 1.5]

----------------------------------------

Sensitivity Analysis for Histology: Leiomyomatous / Spindle cell

Scenario Posterior_Mean_Proportion X95..CI..Proportion.

1 Main Analysis (All Studies) 0.1276071 [0.03, 0.38]

2 Overlap Exclusion NA <NA>

3 Bias Exclusion 0.1276071 [0.03, 0.38]

4 Combined Exclusion NA <NA>

Posterior_Mean_Tau X95..CI..Tau.

1 0.5995721 [0, 1.52]

2 NA <NA>

3 0.5995721 [0, 1.52]

4 NA <NA>

----------------------------------------

Sensitivity Analysis for Histology: Mixed-type

Scenario Posterior_Mean_Proportion X95..CI..Proportion.

1 Main Analysis (All Studies) 0.2992639 [0.08, 0.69]

2 Overlap Exclusion NA <NA>

3 Bias Exclusion 0.2992639 [0.08, 0.69]

4 Combined Exclusion NA <NA>

Posterior_Mean_Tau X95..CI..Tau.

1 0.6697461 [0, 1.67]

2 NA <NA>

3 0.6697461 [0, 1.67]

4 NA <NA>

----------------------------------------

Sensitivity Analysis for Histology: Botryoid

Scenario Posterior_Mean_Proportion X95..CI..Proportion.

1 Main Analysis (All Studies) 0.1904269 [0.08, 0.38]

2 Overlap Exclusion 0.1949241 [0.08, 0.42]

3 Bias Exclusion 0.1904269 [0.08, 0.38]

4 Combined Exclusion 0.1949241 [0.08, 0.42]

Posterior_Mean_Tau X95..CI..Tau.

1 0.4601930 [0, 1.19]

2 0.5137016 [0, 1.33]

3 0.4601930 [0, 1.19]

4 0.5137016 [0, 1.33]

----------------------------------------

Sensitivity Analysis for Histology: Undifferentiated sarcoma

Scenario Posterior_Mean_Proportion X95..CI..Proportion.

1 Main Analysis (All Studies) 0.2822059 [0.1, 0.56]

2 Overlap Exclusion 0.3093158 [0.1, 0.64]

3 Bias Exclusion 0.2822059 [0.1, 0.56]

4 Combined Exclusion 0.3093158 [0.1, 0.64]

Posterior_Mean_Tau X95..CI..Tau.

1 0.5664833 [0, 1.46]

2 0.6024748 [0, 1.55]

3 0.5664833 [0, 1.46]

4 0.6024748 [0, 1.55]

----------------------------------------

**NRSTS**

- all patients: 11546
- **LN all patients**
  - Posterior Mean Proportion of Events: 0.1117
    - 95% Credible Interval for Proportion: 0.0927 - 0.1339
  - Estimated Percentage of Events: 11.17 %
    - 95% Credible Interval for Percentage: 9.27 % - 13.39 %
  - Posterior Mean of Between-Study Standard Deviation (τ): 0.9368
    - 95% Credible Interval for τ: 0.7678 - 1.1139
  - Sensitivity Analysis

*Mean* *CI_Lower* *CI_Upper*

Main Analysis (All Studies) 0.1116715 0.09274296 0.1338526

Overlap Exclusion 0.1092029 0.07717161 0.1522800

Bias Exclusion 0.1127306 0.09324100 0.1356419

Combined Exclusion 0.1092029 0.07717161 0.1522800

**NRSTS Location**

*Location Number of Studies Number of patients*

Abdomen, Pelvis 18 158

Biliary tract / Liver 7 136

Body wall, Trunk 16 138

Extremity 30 977

Gastrointestinal tract 1 4

GU - Non-BP 2 3

Head and neck, non-parameningeal 14 101

Head and neck, NS 6 70

Head and neck, parameningeal 3 16

Orbit 3 5

Paratesticular 1 1

Perineal / Perianal 1 1

Retroperitoneal 10 18

Thorax 12 95

Visceral 19 679

============================================

Bayesian Meta-analysis for Location: Extremity

============================================

Posterior Mean Proportion of Events: 0.0747

95% Credible Interval for Proportion: 0.0478 - 0.1168

Estimated Percentage of Events: 7.47 %

95% Credible Interval for Percentage: 4.78 % - 11.68 %

============================================

Bayesian Meta-analysis for Location: Body wall, Trunk

============================================

Posterior Mean Proportion of Events: 0.0954

95% Credible Interval for Proportion: 0.0458 - 0.1903

Estimated Percentage of Events: 9.54 %

95% Credible Interval for Percentage: 4.58 % - 19.03 %

============================================

Bayesian Meta-analysis for Location: Visceral

============================================

Posterior Mean Proportion of Events: 0.205

95% Credible Interval for Proportion: 0.156 - 0.2657

Estimated Percentage of Events: 20.5 %

95% Credible Interval for Percentage: 15.6 % - 26.57 %

============================================

Bayesian Meta-analysis for Location: Head and neck, NS

============================================

Posterior Mean Proportion of Events: 0.095

95% Credible Interval for Proportion: 0.0257 - 0.3024

Estimated Percentage of Events: 9.5 %

95% Credible Interval for Percentage: 2.57 % - 30.24 %

============================================

Bayesian Meta-analysis for Location: Abdomen, Pelvis

============================================

Posterior Mean Proportion of Events: 0.3392

95% Credible Interval for Proportion: 0.2299 - 0.4662

Estimated Percentage of Events: 33.92 %

95% Credible Interval for Percentage: 22.99 % - 46.62 %

============================================

Bayesian Meta-analysis for Location: Thorax

============================================

Posterior Mean Proportion of Events: 0.1431

95% Credible Interval for Proportion: 0.074 - 0.2613

Estimated Percentage of Events: 14.31 %

95% Credible Interval for Percentage: 7.4 % - 26.13 %

============================================

Bayesian Meta-analysis for Location: Head and neck, non-parameningeal

============================================

Posterior Mean Proportion of Events: 0.1455

95% Credible Interval for Proportion: 0.07 - 0.2825

Estimated Percentage of Events: 14.55 %

95% Credible Interval for Percentage: 7 % - 28.25 %

============================================

Bayesian Meta-analysis for Location: Retroperitoneal

============================================

Posterior Mean Proportion of Events: 0.3251

95% Credible Interval for Proportion: 0.1368 - 0.594

Estimated Percentage of Events: 32.51 %

95% Credible Interval for Percentage: 13.68 % - 59.4 %

============================================

Bayesian Meta-analysis for Location: Orbit

============================================

Posterior Mean Proportion of Events: 0.2891

95% Credible Interval for Proportion: 0.0322 - 0.833

Estimated Percentage of Events: 28.91 %

95% Credible Interval for Percentage: 3.22 % - 83.3 %

============================================

Bayesian Meta-analysis for Location: Biliary tract / Liver

============================================

Posterior Mean Proportion of Events: 0.1147

95% Credible Interval for Proportion: 0.0339 - 0.3453

Estimated Percentage of Events: 11.47 %

95% Credible Interval for Percentage: 3.39 % - 34.53 %

============================================

Bayesian Meta-analysis for Location: Head and neck, parameningeal

============================================

Posterior Mean Proportion of Events: 0.0887

95% Credible Interval for Proportion: 0.0101 - 0.4832

Estimated Percentage of Events: 8.87 %

95% Credible Interval for Percentage: 1.01 % - 48.32 %

============================================

Bayesian Meta-analysis for Location: Extremity

============================================

Posterior Mean of Between-Study Standard Deviation (τ): 0.649

95% Credible Interval for τ: 0.0801 - 1.1844

============================================

Bayesian Meta-analysis for Location: Body wall, Trunk

============================================

Posterior Mean of Between-Study Standard Deviation (τ): 0.4456

95% Credible Interval for τ: 0 - 1.0479

============================================

Bayesian Meta-analysis for Location: Visceral

============================================

Posterior Mean of Between-Study Standard Deviation (τ): 0.4094

95% Credible Interval for τ: 0.0331 - 0.7565

============================================

Bayesian Meta-analysis for Location: Head and neck, NS

============================================

Posterior Mean of Between-Study Standard Deviation (τ): 0.7401

95% Credible Interval for τ: 0 - 1.6804

============================================

Bayesian Meta-analysis for Location: Abdomen, Pelvis

============================================

Posterior Mean of Between-Study Standard Deviation (τ): 0.486

95% Credible Interval for τ: 0 - 1.0887

============================================

Bayesian Meta-analysis for Location: Thorax

============================================

Posterior Mean of Between-Study Standard Deviation (τ): 0.3559

95% Credible Interval for τ: 0 - 0.9169

============================================

Bayesian Meta-analysis for Location: Head and neck, non-parameningeal

============================================

Posterior Mean of Between-Study Standard Deviation (τ): 0.4649

95% Credible Interval for τ: 0 - 1.1315

============================================

Bayesian Meta-analysis for Location: Retroperitoneal

============================================

Posterior Mean of Between-Study Standard Deviation (τ): 0.4507

95% Credible Interval for τ: 0 - 1.1428

============================================

Bayesian Meta-analysis for Location: Orbit

============================================

Posterior Mean of Between-Study Standard Deviation (τ): 0.7215

95% Credible Interval for τ: 0 - 1.7844

============================================

Bayesian Meta-analysis for Location: Biliary tract / Liver

============================================

Posterior Mean of Between-Study Standard Deviation (τ): 0.9344

95% Credible Interval for τ: 0 - 1.8755

============================================

Bayesian Meta-analysis for Location: Head and neck, parameningeal

============================================

Posterior Mean of Between-Study Standard Deviation (τ): 0.6989

95% Credible Interval for τ: 0 - 1.7357

Sensitivity Analysis for Location: Extremity

Scenario Posterior_Mean_Proportion

1 Main Analysis (All Studies) 0.07465865

2 Overlap Exclusion 0.05743493

3 Bias Exclusion 0.07418742

4 Combined Exclusion 0.05743493

X95..CI..Proportion. Posterior_Mean_Tau X95..CI..Tau.

1 [0.05, 0.12] 0.6490027 [0.08, 1.18]

2 [0.03, 0.12] 0.5092733 [0, 1.18]

3 [0.05, 0.12] 0.6597590 [0.08, 1.21]

4 [0.03, 0.12] 0.5092733 [0, 1.18]

----------------------------------------

Sensitivity Analysis for Location: Body wall, Trunk

Scenario Posterior_Mean_Proportion

1 Main Analysis (All Studies) 0.0953524

2 Overlap Exclusion 0.1700285

3 Bias Exclusion 0.0953524

4 Combined Exclusion 0.1700285

X95..CI..Proportion. Posterior_Mean_Tau X95..CI..Tau.

1 [0.05, 0.19] 0.4456473 [0, 1.05]

2 [0.06, 0.39] 0.4504187 [0, 1.14]

3 [0.05, 0.19] 0.4456473 [0, 1.05]

4 [0.06, 0.39] 0.4504187 [0, 1.14]

----------------------------------------

Sensitivity Analysis for Location: Visceral

Scenario Posterior_Mean_Proportion

1 Main Analysis (All Studies) 0.2050424

2 Overlap Exclusion 0.2602713

3 Bias Exclusion 0.2050424

4 Combined Exclusion 0.2602713

X95..CI..Proportion. Posterior_Mean_Tau X95..CI..Tau.

1 [0.16, 0.27] 0.4094488 [0.03, 0.76]

2 [0.17, 0.38] 0.3121833 [0, 0.84]

3 [0.16, 0.27] 0.4094488 [0.03, 0.76]

4 [0.17, 0.38] 0.3121833 [0, 0.84]

----------------------------------------

Sensitivity Analysis for Location: Head and neck, NS

Scenario Posterior_Mean_Proportion

1 Main Analysis (All Studies) 0.09500293

2 Overlap Exclusion 0.09579481

3 Bias Exclusion 0.09500293

4 Combined Exclusion 0.09579481

X95..CI..Proportion. Posterior_Mean_Tau X95..CI..Tau.

1 [0.03, 0.3] 0.7400523 [0, 1.68]

2 [0.01, 0.47] 0.6652076 [0, 1.67]

3 [0.03, 0.3] 0.7400523 [0, 1.68]

4 [0.01, 0.47] 0.6652076 [0, 1.67]

----------------------------------------

Sensitivity Analysis for Location: Abdomen, Pelvis

Scenario Posterior_Mean_Proportion

1 Main Analysis (All Studies) 0.3392027

2 Overlap Exclusion 0.3318519

3 Bias Exclusion 0.3392027

4 Combined Exclusion 0.3318519

X95..CI..Proportion. Posterior_Mean_Tau X95..CI..Tau.

1 [0.23, 0.47] 0.4859751 [0, 1.09]

2 [0.18, 0.53] 0.5134479 [0, 1.28]

3 [0.23, 0.47] 0.4859751 [0, 1.09]

4 [0.18, 0.53] 0.5134479 [0, 1.28]

----------------------------------------

Sensitivity Analysis for Location: Thorax

Scenario Posterior_Mean_Proportion

1 Main Analysis (All Studies) 0.1431349

2 Overlap Exclusion 0.2124616

3 Bias Exclusion 0.1431349

4 Combined Exclusion 0.2124616

X95..CI..Proportion. Posterior_Mean_Tau X95..CI..Tau.

1 [0.07, 0.26] 0.3559384 [0, 0.92]

2 [0.02, 0.79] 0.6969900 [0, 1.73]

3 [0.07, 0.26] 0.3559384 [0, 0.92]

4 [0.02, 0.79] 0.6969900 [0, 1.73]

----------------------------------------

Sensitivity Analysis for Location: Head and neck, non-parameningeal

Scenario Posterior_Mean_Proportion

1 Main Analysis (All Studies) 0.1455037

2 Overlap Exclusion 0.1498374

3 Bias Exclusion 0.1455037

4 Combined Exclusion 0.1498374

X95..CI..Proportion. Posterior_Mean_Tau X95..CI..Tau.

1 [0.07, 0.28] 0.4649450 [0, 1.13]

2 [0.03, 0.47] 0.5581589 [0, 1.41]

3 [0.07, 0.28] 0.4649450 [0, 1.13]

4 [0.03, 0.47] 0.5581589 [0, 1.41]

----------------------------------------

Sensitivity Analysis for Location: Retroperitoneal

Scenario Posterior_Mean_Proportion

1 Main Analysis (All Studies) 0.3251334

2 Overlap Exclusion 0.3114508

3 Bias Exclusion 0.3251334

4 Combined Exclusion 0.3114508

X95..CI..Proportion. Posterior_Mean_Tau X95..CI..Tau.

1 [0.14, 0.59] 0.4506877 [0, 1.14]

2 [0.09, 0.68] 0.5716605 [0, 1.45]

3 [0.14, 0.59] 0.4506877 [0, 1.14]

4 [0.09, 0.68] 0.5716605 [0, 1.45]

----------------------------------------

Sensitivity Analysis for Location: Orbit

Scenario Posterior_Mean_Proportion

1 Main Analysis (All Studies) 0.2890583

2 Overlap Exclusion NA

3 Bias Exclusion 0.2890583

4 Combined Exclusion NA

X95..CI..Proportion. Posterior_Mean_Tau X95..CI..Tau.

1 [0.03, 0.83] 0.7215372 [0, 1.78]

2 <NA> NA <NA>

3 [0.03, 0.83] 0.7215372 [0, 1.78]

4 <NA> NA <NA>

----------------------------------------

Sensitivity Analysis for Location: Biliary tract / Liver

Scenario Posterior_Mean_Proportion

1 Main Analysis (All Studies) 0.1147112

2 Overlap Exclusion 0.4241246

3 Bias Exclusion 0.1147112

4 Combined Exclusion 0.4241246

X95..CI..Proportion. Posterior_Mean_Tau X95..CI..Tau.

1 [0.03, 0.35] 0.9343958 [0, 1.88]

2 [0.08, 0.87] 0.6951498 [0, 1.73]

3 [0.03, 0.35] 0.9343958 [0, 1.88]

4 [0.08, 0.87] 0.6951498 [0, 1.73]

----------------------------------------

Sensitivity Analysis for Location: Head and neck, parameningeal

Scenario Posterior_Mean_Proportion

1 Main Analysis (All Studies) 0.08869568

2 Overlap Exclusion NA

3 Bias Exclusion 0.08869568

4 Combined Exclusion NA

X95..CI..Proportion. Posterior_Mean_Tau X95..CI..Tau.

1 [0.01, 0.48] 0.6989178 [0, 1.74]

2 <NA> NA <NA>

3 [0.01, 0.48] 0.6989178 [0, 1.74]

4 <NA> NA <NA>

----------------------------------------

**NRSTS Histology**

*Histology Number of Studies Number of patients*

Adult-type fibrosarcoma 5 33

Alveolar soft part sarcoma 15 546

Angiomatoid fibrous histiocytoma 3 44

Angiosarcoma 5 45

Chondrosarcoma 2 2

Clear cell sarcoma 16 638

Dermatofibrosarcoma protuberans 3 92

Desmoplastic small round cell tumor 9 285

Embryonal sarcoma of the liver 1 39

Epithelioid hemangioendothelioma 2 27

Epithelioid sarcoma 15 454

Ewing sarcoma 13 502

Fibromyxoid sarcoma 1 1

Fibrosarcoma 2 27

Hemangiopericytoma 3 18

High-grade sarcoma 1 2

Infantile fibrosarcoma 4 138

Inflammatory myofibroblastic tumor 3 139

Leiomyosarcoma 6 104

Liposarcoma 7 250

Malignant fibrous histiocytoma 4 57

Malignant glomus tumor 1 1

Malignant rhabdoid tumor 8 253

MPNST 10 815

Primitive peripheral neuroectodermic tumor 5 220

Synovial sarcoma 18 2444

Undifferentiated sarcoma 7 214

============================================

Bayesian Meta-analysis for Histology: Epithelioid sarcoma

============================================

Posterior Mean Proportion of Events: 0.1685

95% Credible Interval for Proportion: 0.1226 - 0.228

Estimated Percentage of Events: 16.85 %

95% Credible Interval for Percentage: 12.26 % - 22.8 %

============================================

Bayesian Meta-analysis for Histology: Synovial sarcoma

============================================

Posterior Mean Proportion of Events: 0.0572

95% Credible Interval for Proportion: 0.0375 - 0.0871

Estimated Percentage of Events: 5.72 %

95% Credible Interval for Percentage: 3.75 % - 8.71 %

============================================

Bayesian Meta-analysis for Histology: Undifferentiated sarcoma

============================================

Posterior Mean Proportion of Events: 0.0833

95% Credible Interval for Proportion: 0.0329 - 0.2081

Estimated Percentage of Events: 8.33 %

95% Credible Interval for Percentage: 3.29 % - 20.81 %

============================================

Bayesian Meta-analysis for Histology: Clear cell sarcoma

============================================

Posterior Mean Proportion of Events: 0.2121

95% Credible Interval for Proportion: 0.163 - 0.2749

Estimated Percentage of Events: 21.21 %

95% Credible Interval for Percentage: 16.3 % - 27.49 %

============================================

Bayesian Meta-analysis for Histology: MPNST

============================================

Posterior Mean Proportion of Events: 0.0539

95% Credible Interval for Proportion: 0.0296 - 0.0924

Estimated Percentage of Events: 5.39 %

95% Credible Interval for Percentage: 2.96 % - 9.24 %

============================================

Bayesian Meta-analysis for Histology: Angiosarcoma

============================================

Posterior Mean Proportion of Events: 0.1547

95% Credible Interval for Proportion: 0.063 - 0.3323

Estimated Percentage of Events: 15.47 %

95% Credible Interval for Percentage: 6.3 % - 33.23 %

============================================

Bayesian Meta-analysis for Histology: Alveolar soft part sarcoma

============================================

Posterior Mean Proportion of Events: 0.0481

95% Credible Interval for Proportion: 0.0306 - 0.0747

Estimated Percentage of Events: 4.81 %

95% Credible Interval for Percentage: 3.06 % - 7.47 %

============================================

Bayesian Meta-analysis for Histology: Liposarcoma

============================================

Posterior Mean Proportion of Events: 0.0543

95% Credible Interval for Proportion: 0.0211 - 0.1312

Estimated Percentage of Events: 5.43 %

95% Credible Interval for Percentage: 2.11 % - 13.12 %

============================================

Bayesian Meta-analysis for Histology: PPNT

============================================

Posterior Mean Proportion of Events: 0.1966

95% Credible Interval for Proportion: 0.0843 - 0.3977

Estimated Percentage of Events: 19.66 %

95% Credible Interval for Percentage: 8.43 % - 39.77 %

============================================

Bayesian Meta-analysis for Histology: DSRCT

============================================

Posterior Mean Proportion of Events: 0.4395

95% Credible Interval for Proportion: 0.3347 - 0.5516

Estimated Percentage of Events: 43.95 %

95% Credible Interval for Percentage: 33.47 % - 55.16 %

============================================

Bayesian Meta-analysis for Histology: Malignant rhabdoid tumor

============================================

Posterior Mean Proportion of Events: 0.1993

95% Credible Interval for Proportion: 0.1411 - 0.273

Estimated Percentage of Events: 19.93 %

95% Credible Interval for Percentage: 14.11 % - 27.3 %

============================================

Bayesian Meta-analysis for Histology: Dermatofibrosarcoma protuberans

============================================

Posterior Mean Proportion of Events: 0.0313

95% Credible Interval for Proportion: 0.0061 - 0.1445

Estimated Percentage of Events: 3.13 %

95% Credible Interval for Percentage: 0.61 % - 14.45 %

============================================

Bayesian Meta-analysis for Histology: Angiomatoid fibrous histiocytoma

============================================

Posterior Mean Proportion of Events: 0.0952

95% Credible Interval for Proportion: 0.0195 - 0.3651

Estimated Percentage of Events: 9.52 %

95% Credible Interval for Percentage: 1.95 % - 36.51 %

============================================

Bayesian Meta-analysis for Histology: Ewing sarcoma

============================================

Posterior Mean Proportion of Events: 0.1297

95% Credible Interval for Proportion: 0.0818 - 0.2005

Estimated Percentage of Events: 12.97 %

95% Credible Interval for Percentage: 8.18 % - 20.05 %

============================================

Bayesian Meta-analysis for Histology: Infantile Fibrosarcoma

============================================

Posterior Mean Proportion of Events: 0.037

95% Credible Interval for Proportion: 0.0099 - 0.1281

Estimated Percentage of Events: 3.7 %

95% Credible Interval for Percentage: 0.99 % - 12.81 %

============================================

Bayesian Meta-analysis for Histology: Hemangiopericytoma

============================================

Posterior Mean Proportion of Events: 0.1681

95% Credible Interval for Proportion: 0.0349 - 0.527

Estimated Percentage of Events: 16.81 %

95% Credible Interval for Percentage: 3.49 % - 52.7 %

============================================

Bayesian Meta-analysis for Histology: Leiomyosarcoma

============================================

Posterior Mean Proportion of Events: 0.1318

95% Credible Interval for Proportion: 0.0428 - 0.3353

Estimated Percentage of Events: 13.18 %

95% Credible Interval for Percentage: 4.28 % - 33.53 %

============================================

Bayesian Meta-analysis for Histology: Adult-Type Fibrosarcoma

============================================

Posterior Mean Proportion of Events: 0.1201

95% Credible Interval for Proportion: 0.0343 - 0.3457

Estimated Percentage of Events: 12.01 %

95% Credible Interval for Percentage: 3.43 % - 34.57 %

============================================

Bayesian Meta-analysis for Histology: Malignant fibrous histiocytoma

============================================

Posterior Mean Proportion of Events: 0.1626

95% Credible Interval for Proportion: 0.0577 - 0.3798

Estimated Percentage of Events: 16.26 %

95% Credible Interval for Percentage: 5.77 % - 37.98 %

============================================

Bayesian Meta-analysis for Histology: Inflammatory myofibroblastic tumor

============================================

Posterior Mean Proportion of Events: 0.0476

95% Credible Interval for Proportion: 0.0102 - 0.1904

Estimated Percentage of Events: 4.76 %

95% Credible Interval for Percentage: 1.02 % - 19.04 %

============================================

Bayesian Meta-analysis for Histology: Fibrosarcoma

============================================

Posterior Mean Proportion of Events: 0.075

95% Credible Interval for Proportion: 0.0098 - 0.4058

Estimated Percentage of Events: 7.5 %

95% Credible Interval for Percentage: 0.98 % - 40.58 %

============================================

Bayesian Meta-analysis for Histology: Epithelioid hemangioendothelioma

============================================

Posterior Mean Proportion of Events: 0.1434

95% Credible Interval for Proportion: 0.0228 - 0.5129

Estimated Percentage of Events: 14.34 %

95% Credible Interval for Percentage: 2.28 % - 51.29 %

============================================

Bayesian Meta-analysis for Histology: Epithelioid sarcoma

============================================

Posterior Mean of Between-Study Standard Deviation (τ): 0.2511

95% Credible Interval for τ: 0 - 0.654

============================================

Bayesian Meta-analysis for Histology: Synovial sarcoma

============================================

Posterior Mean of Between-Study Standard Deviation (τ): 0.6517

95% Credible Interval for τ: 0.2937 - 1.0611

============================================

Bayesian Meta-analysis for Histology: Undifferentiated sarcoma

============================================

Posterior Mean of Between-Study Standard Deviation (τ): 0.692

95% Credible Interval for τ: 0 - 1.6028

============================================

Bayesian Meta-analysis for Histology: Clear cell sarcoma

============================================

Posterior Mean of Between-Study Standard Deviation (τ): 0.3406

95% Credible Interval for τ: 0 - 0.7035

============================================

Bayesian Meta-analysis for Histology: MPNST

============================================

Posterior Mean of Between-Study Standard Deviation (τ): 0.5137

95% Credible Interval for τ: 0 - 1.0518

============================================

Bayesian Meta-analysis for Histology: Angiosarcoma

============================================

Posterior Mean of Between-Study Standard Deviation (τ): 0.4585

95% Credible Interval for τ: 0 - 1.1956

============================================

Bayesian Meta-analysis for Histology: Alveolar soft part sarcoma

============================================

Posterior Mean of Between-Study Standard Deviation (τ): 0.2617

95% Credible Interval for τ: 0 - 0.6616

============================================

Bayesian Meta-analysis for Histology: Liposarcoma

============================================

Posterior Mean of Between-Study Standard Deviation (τ): 0.539

95% Credible Interval for τ: 0 - 1.3552

============================================

Bayesian Meta-analysis for Histology: PPNT

============================================

Posterior Mean of Between-Study Standard Deviation (τ): 0.7891

95% Credible Interval for τ: 0.1184 - 1.5989

============================================

Bayesian Meta-analysis for Histology: DSRCT

============================================

Posterior Mean of Between-Study Standard Deviation (τ): 0.3943

95% Credible Interval for τ: 0 - 0.9004

============================================

Bayesian Meta-analysis for Histology: Malignant rhabdoid tumor

============================================

Posterior Mean of Between-Study Standard Deviation (τ): 0.2523

95% Credible Interval for τ: 0 - 0.6611

============================================

Bayesian Meta-analysis for Histology: Dermatofibrosarcoma protuberans

============================================

Posterior Mean of Between-Study Standard Deviation (τ): 0.6504

95% Credible Interval for τ: 0 - 1.64

============================================

Bayesian Meta-analysis for Histology: Angiomatoid fibrous histiocytoma

============================================

Posterior Mean of Between-Study Standard Deviation (τ): 0.7297

95% Credible Interval for τ: 0 - 1.7547

============================================

Bayesian Meta-analysis for Histology: Ewing sarcoma

============================================

Posterior Mean of Between-Study Standard Deviation (τ): 0.5495

95% Credible Interval for τ: 0.1085 - 1.0439

============================================

Bayesian Meta-analysis for Histology: Infantile Fibrosarcoma

============================================

Posterior Mean of Between-Study Standard Deviation (τ): 0.643

95% Credible Interval for τ: 0 - 1.5753

============================================

Bayesian Meta-analysis for Histology: Hemangiopericytoma

============================================

Posterior Mean of Between-Study Standard Deviation (τ): 0.6551

95% Credible Interval for τ: 0 - 1.6508

============================================

Bayesian Meta-analysis for Histology: Leiomyosarcoma

============================================

Posterior Mean of Between-Study Standard Deviation (τ): 0.7646

95% Credible Interval for τ: 0 - 1.7452

============================================

Bayesian Meta-analysis for Histology: Adult-Type Fibrosarcoma

============================================

Posterior Mean of Between-Study Standard Deviation (τ): 0.5507

95% Credible Interval for τ: 0 - 1.4053

============================================

Bayesian Meta-analysis for Histology: Malignant fibrous histiocytoma

============================================

Posterior Mean of Between-Study Standard Deviation (τ): 0.5276

95% Credible Interval for τ: 0 - 1.3845

============================================

Bayesian Meta-analysis for Histology: Inflammatory myofibroblastic tumor

============================================

Posterior Mean of Between-Study Standard Deviation (τ): 0.7858

95% Credible Interval for τ: 0 - 1.8439

============================================

Bayesian Meta-analysis for Histology: Fibrosarcoma

============================================

Posterior Mean of Between-Study Standard Deviation (τ): 0.7247

95% Credible Interval for τ: 0 - 1.802

============================================

Bayesian Meta-analysis for Histology: Epithelioid hemangioendothelioma

============================================

Posterior Mean of Between-Study Standard Deviation (τ): 0.7555

95% Credible Interval for τ: 0 - 1.8441

Sensitivity Analysis for Histology: Epithelioid sarcoma

Scenario Posterior_Mean_Proportion

1 Main Analysis (All Studies) 0.1684819

2 Overlap Exclusion 0.1256466

3 Bias Exclusion 0.1685497

4 Combined Exclusion 0.1256466

X95..CI..Proportion. Posterior_Mean_Tau X95..CI..Tau.

1 [0.12, 0.23] 0.2510928 [0, 0.65]

2 [0.06, 0.25] 0.4424386 [0, 1.19]

3 [0.12, 0.23] 0.2562918 [0, 0.67]

4 [0.06, 0.25] 0.4424386 [0, 1.19]

----------------------------------------

Sensitivity Analysis for Histology: Synovial sarcoma

Scenario Posterior_Mean_Proportion

1 Main Analysis (All Studies) 0.05715150

2 Overlap Exclusion 0.02700075

3 Bias Exclusion 0.05638260

4 Combined Exclusion 0.02700075

X95..CI..Proportion. Posterior_Mean_Tau X95..CI..Tau.

1 [0.04, 0.09] 0.6517221 [0.29, 1.06]

2 [0.01, 0.08] 0.5898403 [0, 1.52]

3 [0.04, 0.09] 0.6531669 [0.29, 1.07]

4 [0.01, 0.08] 0.5898403 [0, 1.52]

----------------------------------------

Sensitivity Analysis for Histology: Undifferentiated sarcoma

Scenario Posterior_Mean_Proportion

1 Main Analysis (All Studies) 0.08325352

2 Overlap Exclusion NA

3 Bias Exclusion 0.08325352

4 Combined Exclusion NA

X95..CI..Proportion. Posterior_Mean_Tau X95..CI..Tau.

1 [0.03, 0.21] 0.6919769 [0, 1.6]

2 <NA> NA <NA>

3 [0.03, 0.21] 0.6919769 [0, 1.6]

4 <NA> NA <NA>

----------------------------------------

Sensitivity Analysis for Histology: Clear cell sarcoma

Scenario Posterior_Mean_Proportion

1 Main Analysis (All Studies) 0.2120653

2 Overlap Exclusion 0.2500452

3 Bias Exclusion 0.2120653

4 Combined Exclusion 0.2500452

X95..CI..Proportion. Posterior_Mean_Tau X95..CI..Tau.

1 [0.16, 0.27] 0.3406088 [0, 0.7]

2 [0.14, 0.42] 0.4046298 [0, 1.12]

3 [0.16, 0.27] 0.3406088 [0, 0.7]

4 [0.14, 0.42] 0.4046298 [0, 1.12]

----------------------------------------

Sensitivity Analysis for Histology: MPNST

Scenario Posterior_Mean_Proportion

1 Main Analysis (All Studies) 0.05392354

2 Overlap Exclusion 0.04464062

3 Bias Exclusion 0.05392354

4 Combined Exclusion 0.04464062

X95..CI..Proportion. Posterior_Mean_Tau X95..CI..Tau.

1 [0.03, 0.09] 0.5136547 [0, 1.05]

2 [0.02, 0.08] 0.4375443 [0, 1.05]

3 [0.03, 0.09] 0.5136547 [0, 1.05]

4 [0.02, 0.08] 0.4375443 [0, 1.05]

----------------------------------------

Sensitivity Analysis for Histology: Angiosarcoma

Scenario Posterior_Mean_Proportion

1 Main Analysis (All Studies) 0.1546904

2 Overlap Exclusion NA

3 Bias Exclusion 0.1546904

4 Combined Exclusion NA

X95..CI..Proportion. Posterior_Mean_Tau X95..CI..Tau.

1 [0.06, 0.33] 0.4585016 [0, 1.2]

2 <NA> NA <NA>

3 [0.06, 0.33] 0.4585016 [0, 1.2]

4 <NA> NA <NA>

----------------------------------------

Sensitivity Analysis for Histology: Alveolar soft part sarcoma

Scenario Posterior_Mean_Proportion

1 Main Analysis (All Studies) 0.04807301

2 Overlap Exclusion 0.03574937

3 Bias Exclusion 0.04807301

4 Combined Exclusion 0.03574937

X95..CI..Proportion. Posterior_Mean_Tau X95..CI..Tau.

1 [0.03, 0.07] 0.2616819 [0, 0.66]

2 [0.01, 0.16] 0.6534460 [0, 1.63]

3 [0.03, 0.07] 0.2616819 [0, 0.66]

4 [0.01, 0.16] 0.6534460 [0, 1.63]

----------------------------------------

Sensitivity Analysis for Histology: Liposarcoma

Scenario Posterior_Mean_Proportion

1 Main Analysis (All Studies) 0.05425413

2 Overlap Exclusion 0.07987744

3 Bias Exclusion 0.05425413

4 Combined Exclusion 0.07987744

X95..CI..Proportion. Posterior_Mean_Tau X95..CI..Tau.

1 [0.02, 0.13] 0.5389501 [0, 1.36]

2 [0.02, 0.33] 0.6458726 [0, 1.63]

3 [0.02, 0.13] 0.5389501 [0, 1.36]

4 [0.02, 0.33] 0.6458726 [0, 1.63]

----------------------------------------

Sensitivity Analysis for Histology: PPNT

Scenario Posterior_Mean_Proportion

1 Main Analysis (All Studies) 0.1966115

2 Overlap Exclusion NA

3 Bias Exclusion 0.1966115

4 Combined Exclusion NA

X95..CI..Proportion. Posterior_Mean_Tau X95..CI..Tau.

1 [0.08, 0.4] 0.7890573 [0.12, 1.6]

2 <NA> NA <NA>

3 [0.08, 0.4] 0.7890573 [0.12, 1.6]

4 <NA> NA <NA>

----------------------------------------

Sensitivity Analysis for Histology: DSRCT

Scenario Posterior_Mean_Proportion

1 Main Analysis (All Studies) 0.4395199

2 Overlap Exclusion 0.3782355

3 Bias Exclusion 0.4395199

4 Combined Exclusion 0.3782355

X95..CI..Proportion. Posterior_Mean_Tau X95..CI..Tau.

1 [0.33, 0.55] 0.3942763 [0, 0.9]

2 [0.16, 0.68] 0.6995123 [0, 1.63]

3 [0.33, 0.55] 0.3942763 [0, 0.9]

4 [0.16, 0.68] 0.6995123 [0, 1.63]

----------------------------------------

Sensitivity Analysis for Histology: Malignant rhabdoid tumor

Scenario Posterior_Mean_Proportion

1 Main Analysis (All Studies) 0.1992541

2 Overlap Exclusion 0.1967495

3 Bias Exclusion 0.1992541

4 Combined Exclusion 0.1967495

X95..CI..Proportion. Posterior_Mean_Tau X95..CI..Tau.

1 [0.14, 0.27] 0.252350 [0, 0.66]

2 [0.09, 0.36] 0.425634 [0, 1.2]

3 [0.14, 0.27] 0.252350 [0, 0.66]

4 [0.09, 0.36] 0.425634 [0, 1.2]

----------------------------------------

Sensitivity Analysis for Histology: Dermatofibrosarcoma protuberans

Scenario Posterior_Mean_Proportion

1 Main Analysis (All Studies) 0.03129736

2 Overlap Exclusion 0.02595868

3 Bias Exclusion 0.03129736

4 Combined Exclusion 0.02595868

X95..CI..Proportion. Posterior_Mean_Tau X95..CI..Tau.

1 [0.01, 0.14] 0.6503903 [0, 1.64]

2 [0, 0.24] 0.7556937 [0, 1.86]

3 [0.01, 0.14] 0.6503903 [0, 1.64]

4 [0, 0.24] 0.7556937 [0, 1.86]

----------------------------------------

Sensitivity Analysis for Histology: Angiomatoid fibrous histiocytoma

Scenario Posterior_Mean_Proportion

1 Main Analysis (All Studies) 0.09523784

2 Overlap Exclusion NA

3 Bias Exclusion 0.09523784

4 Combined Exclusion NA

X95..CI..Proportion. Posterior_Mean_Tau X95..CI..Tau.

1 [0.02, 0.37] 0.729723 [0, 1.75]

2 <NA> NA <NA>

3 [0.02, 0.37] 0.729723 [0, 1.75]

4 <NA> NA <NA>

----------------------------------------

Sensitivity Analysis for Histology: Ewing sarcoma

Scenario Posterior_Mean_Proportion

1 Main Analysis (All Studies) 0.1296515

2 Overlap Exclusion 0.1737869

3 Bias Exclusion 0.1296515

4 Combined Exclusion 0.1737869

X95..CI..Proportion. Posterior_Mean_Tau X95..CI..Tau.

1 [0.08, 0.2] 0.5494560 [0.11, 1.04]

2 [0.06, 0.38] 0.7741165 [0, 1.61]

3 [0.08, 0.2] 0.5494560 [0.11, 1.04]

4 [0.06, 0.38] 0.7741165 [0, 1.61]

----------------------------------------

Sensitivity Analysis for Histology: Infantile Fibrosarcoma

Scenario Posterior_Mean_Proportion

1 Main Analysis (All Studies) 0.03699826

2 Overlap Exclusion 0.02354640

3 Bias Exclusion 0.03699826

4 Combined Exclusion 0.02354640

X95..CI..Proportion. Posterior_Mean_Tau X95..CI..Tau.

1 [0.01, 0.13] 0.6429637 [0, 1.58]

2 [0, 0.22] 0.7529638 [0, 1.85]

3 [0.01, 0.13] 0.6429637 [0, 1.58]

4 [0, 0.22] 0.7529638 [0, 1.85]

----------------------------------------

Sensitivity Analysis for Histology: Hemangiopericytoma

Scenario Posterior_Mean_Proportion

1 Main Analysis (All Studies) 0.1680797

2 Overlap Exclusion 0.1257900

3 Bias Exclusion 0.1680797

4 Combined Exclusion 0.1257900

X95..CI..Proportion. Posterior_Mean_Tau X95..CI..Tau.

1 [0.03, 0.53] 0.6550963 [0, 1.65]

2 [0.01, 0.73] 0.7450361 [0, 1.84]

3 [0.03, 0.53] 0.6550963 [0, 1.65]

4 [0.01, 0.73] 0.7450361 [0, 1.84]

----------------------------------------

Sensitivity Analysis for Histology: Leiomyosarcoma

Scenario Posterior_Mean_Proportion

1 Main Analysis (All Studies) 0.1317668

2 Overlap Exclusion 0.2317183

3 Bias Exclusion 0.1317668

4 Combined Exclusion 0.2317183

X95..CI..Proportion. Posterior_Mean_Tau X95..CI..Tau.

1 [0.04, 0.34] 0.7646023 [0, 1.75]

2 [0.06, 0.62] 0.7628465 [0, 1.81]

3 [0.04, 0.34] 0.7646023 [0, 1.75]

4 [0.06, 0.62] 0.7628465 [0, 1.81]

----------------------------------------

Sensitivity Analysis for Histology: Adult-Type Fibrosarcoma

Scenario Posterior_Mean_Proportion

1 Main Analysis (All Studies) 0.1200986

2 Overlap Exclusion 0.1114106

3 Bias Exclusion 0.1200986

4 Combined Exclusion 0.1114106

X95..CI..Proportion. Posterior_Mean_Tau X95..CI..Tau.

1 [0.03, 0.35] 0.5507456 [0, 1.41]

2 [0.01, 0.65] 0.7227694 [0, 1.8]

3 [0.03, 0.35] 0.5507456 [0, 1.41]

4 [0.01, 0.65] 0.7227694 [0, 1.8]

----------------------------------------

Sensitivity Analysis for Histology: Malignant fibrous histiocytoma

Scenario Posterior_Mean_Proportion

1 Main Analysis (All Studies) 0.1626172

2 Overlap Exclusion 0.1626172

3 Bias Exclusion 0.1626172

4 Combined Exclusion 0.1626172

X95..CI..Proportion. Posterior_Mean_Tau X95..CI..Tau.

1 [0.06, 0.38] 0.5275639 [0, 1.38]

2 [0.06, 0.38] 0.5275639 [0, 1.38]

3 [0.06, 0.38] 0.5275639 [0, 1.38]

4 [0.06, 0.38] 0.5275639 [0, 1.38]

----------------------------------------

Sensitivity Analysis for Histology: Inflammatory myofibroblastic tumor

Scenario Posterior_Mean_Proportion

1 Main Analysis (All Studies) 0.04763694

2 Overlap Exclusion 0.07255505

3 Bias Exclusion 0.04763694

4 Combined Exclusion 0.07255505

X95..CI..Proportion. Posterior_Mean_Tau X95..CI..Tau.

1 [0.01, 0.19] 0.7858049 [0, 1.84]

2 [0.01, 0.32] 0.7628557 [0, 1.83]

3 [0.01, 0.19] 0.7858049 [0, 1.84]

4 [0.01, 0.32] 0.7628557 [0, 1.83]

----------------------------------------

Sensitivity Analysis for Histology: Fibrosarcoma

Scenario Posterior_Mean_Proportion

1 Main Analysis (All Studies) 0.07501531

2 Overlap Exclusion NA

3 Bias Exclusion 0.07501531

4 Combined Exclusion NA

X95..CI..Proportion. Posterior_Mean_Tau X95..CI..Tau.

1 [0.01, 0.41] 0.7247217 [0, 1.8]

2 <NA> NA <NA>

3 [0.01, 0.41] 0.7247217 [0, 1.8]

4 <NA> NA <NA>

----------------------------------------

Sensitivity Analysis for Histology: Epithelioid hemangioendothelioma

Scenario Posterior_Mean_Proportion

1 Main Analysis (All Studies) 0.1433653

2 Overlap Exclusion NA

3 Bias Exclusion 0.1433653

4 Combined Exclusion NA

X95..CI..Proportion. Posterior_Mean_Tau X95..CI..Tau.

1 [0.02, 0.51] 0.7554876 [0, 1.84]

2 <NA> NA <NA>

3 [0.02, 0.51] 0.7554876 [0, 1.84]

4 <NA> NA <NA>

----------------------------------------
