# Supplementary figures and images for: Prevalence of locoregional and distant lymph node metastases in children and adolescents/young adults with soft tissue sarcomas: a Bayesian meta-analysis of proportions
Source: eClinicalMedicine. 2025 Aug 7;87:103390. doi: 10.1016/j.eclinm.2025.103390 (PMC12355419; doi:10.1016/j.eclinm.2025.103390)

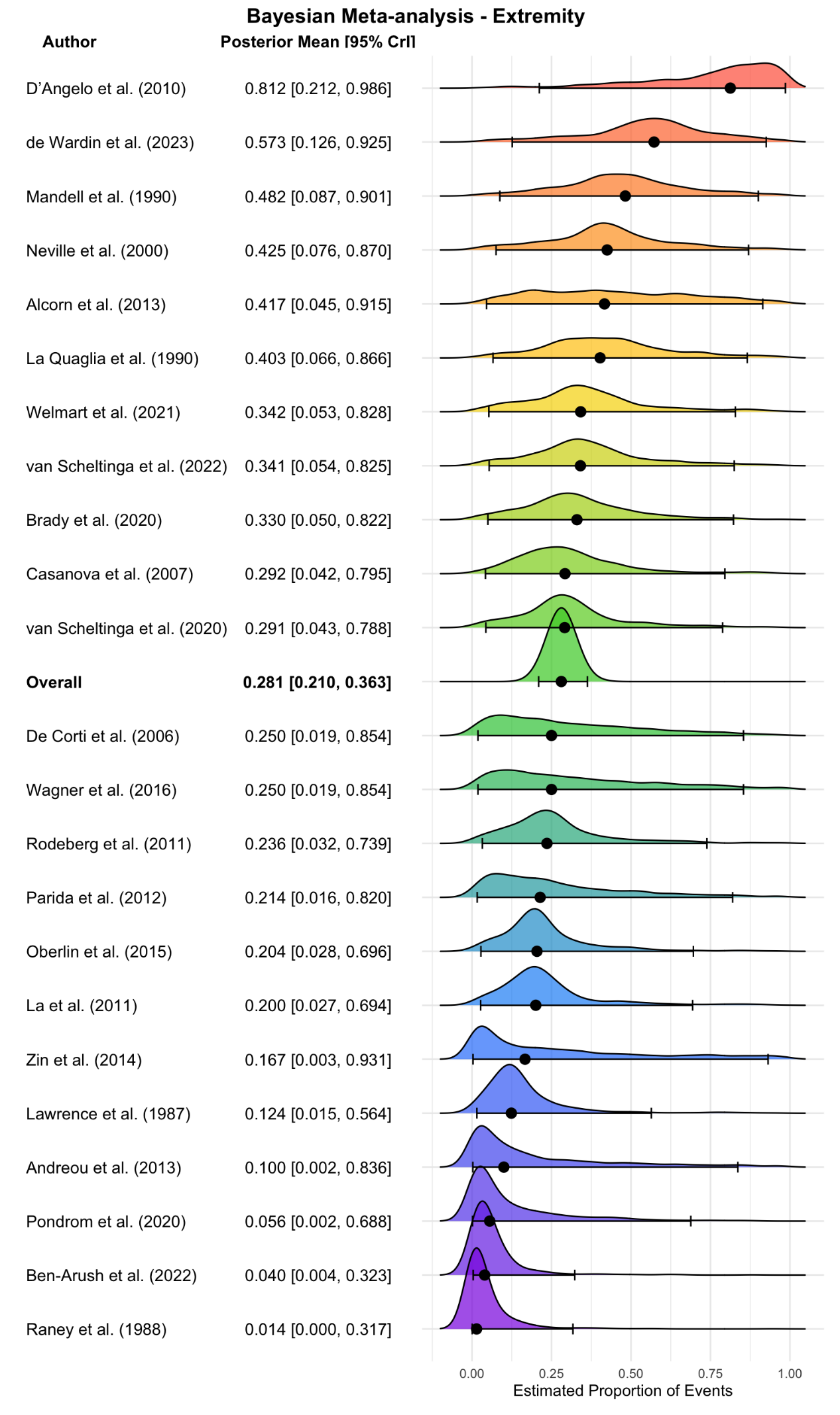


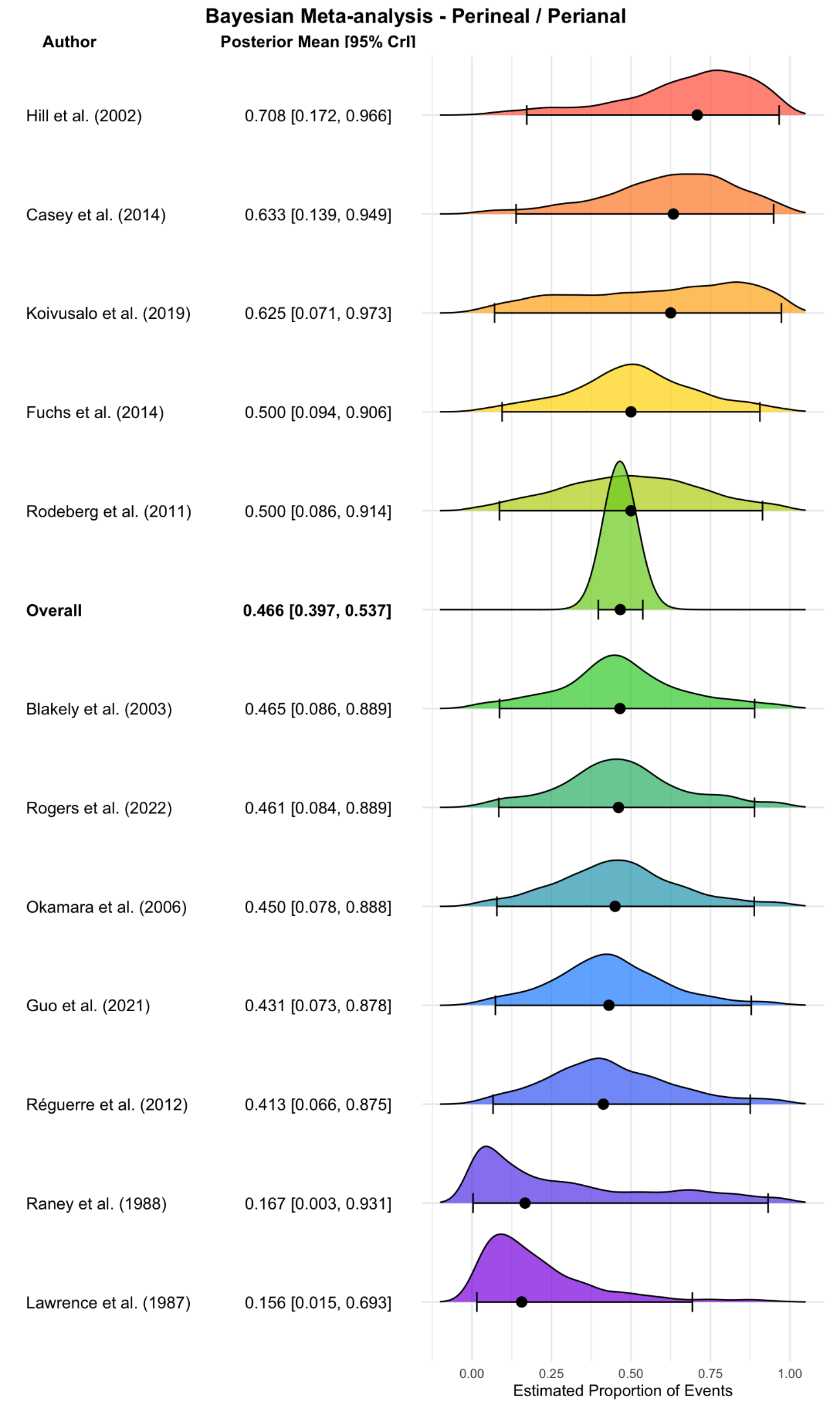


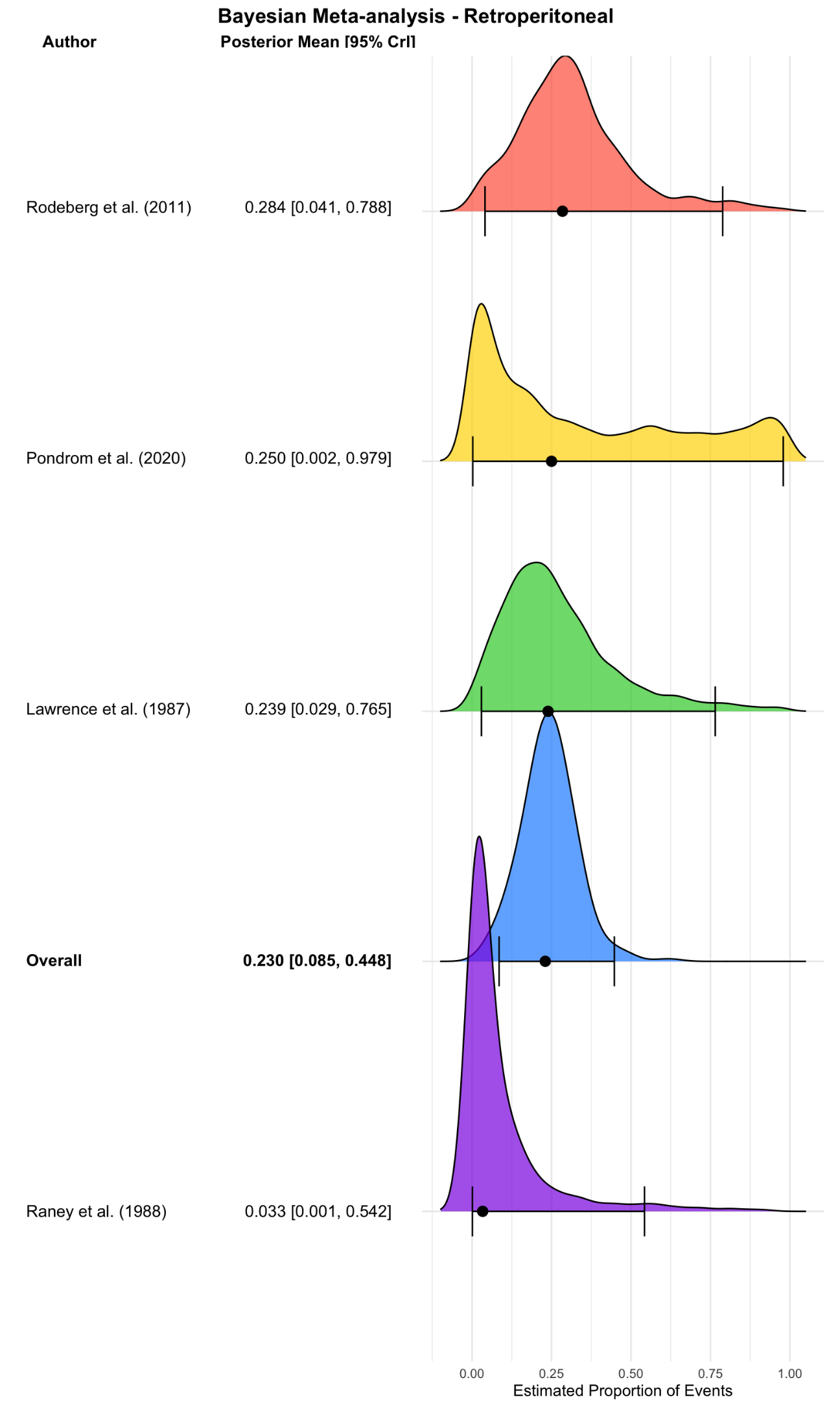


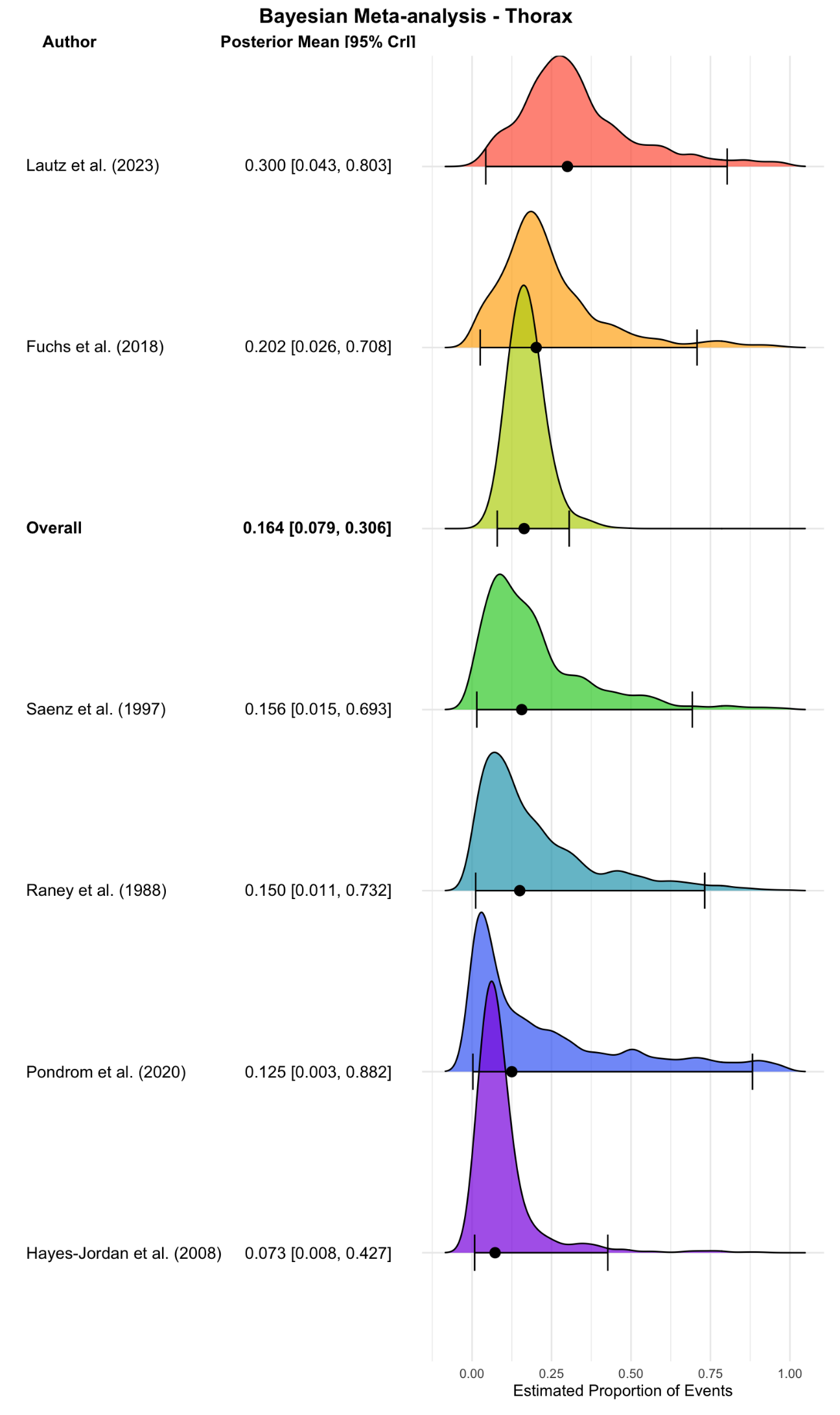


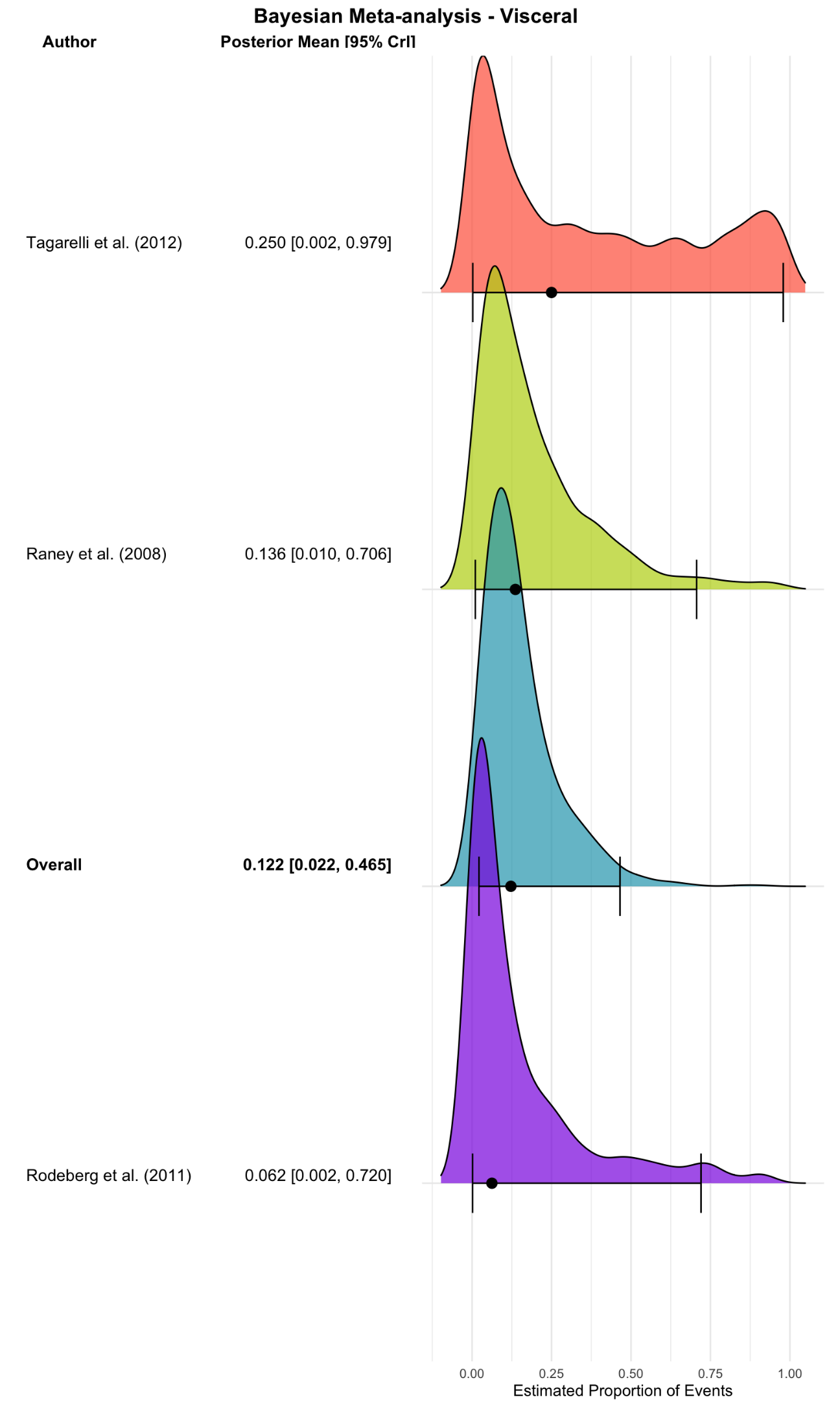


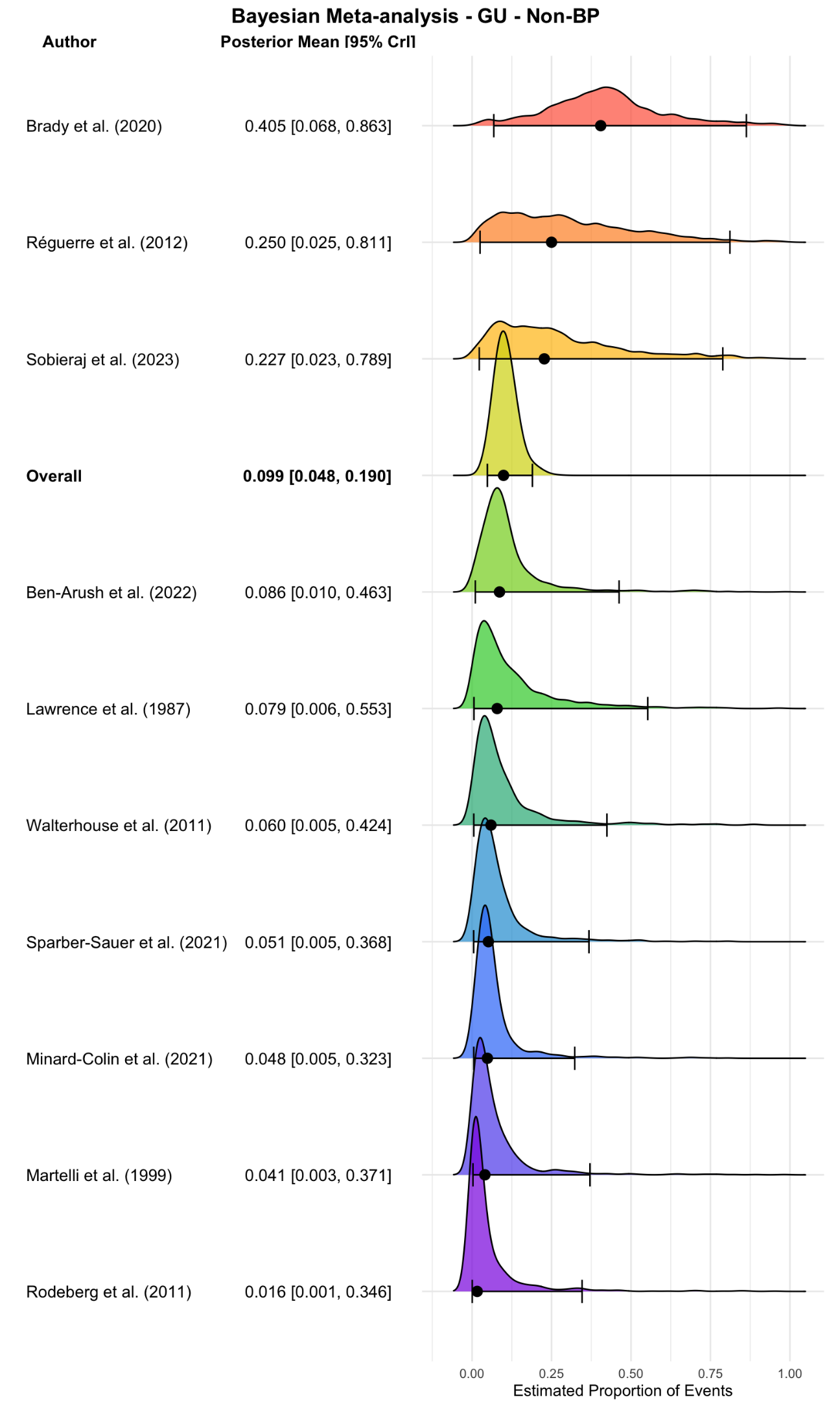


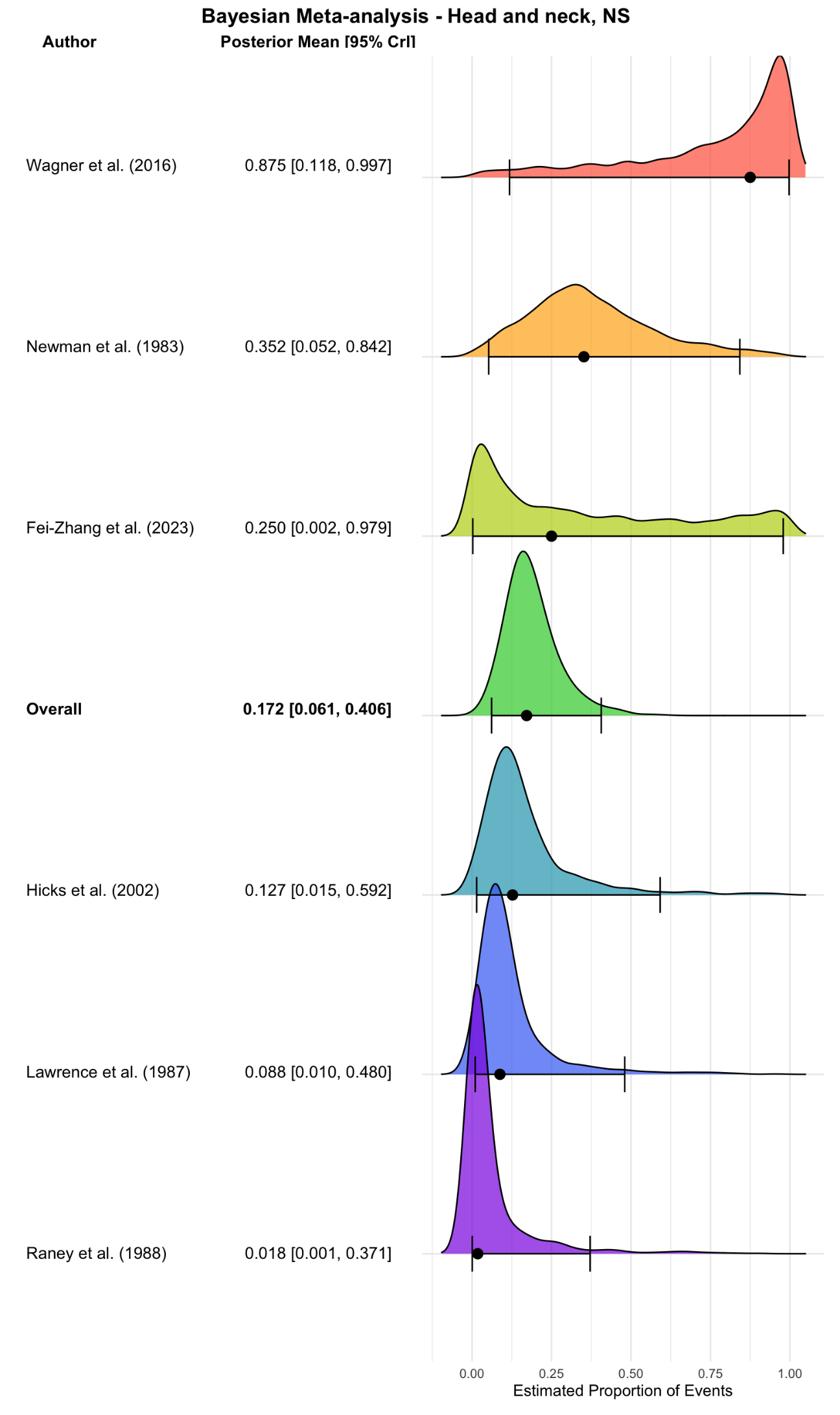


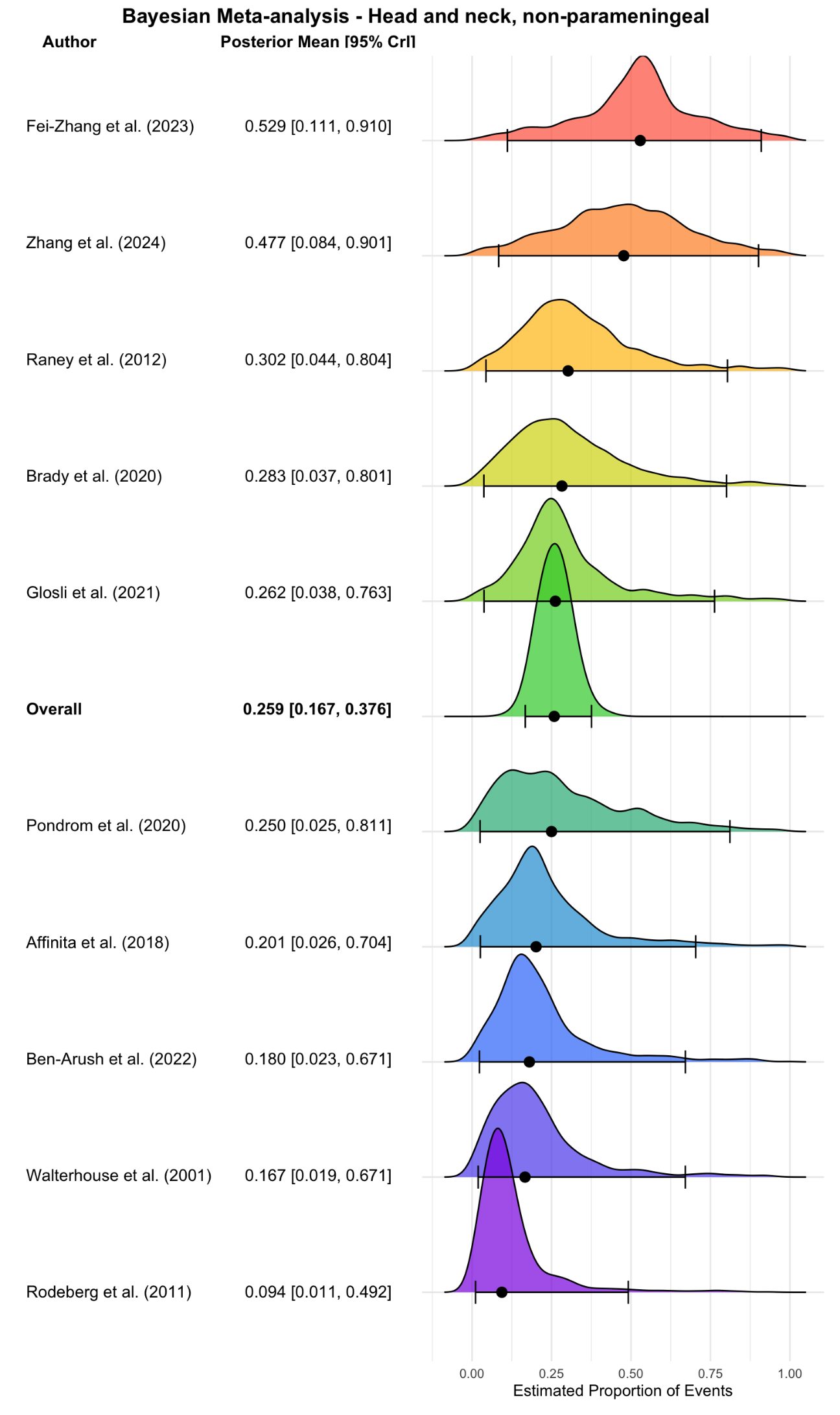


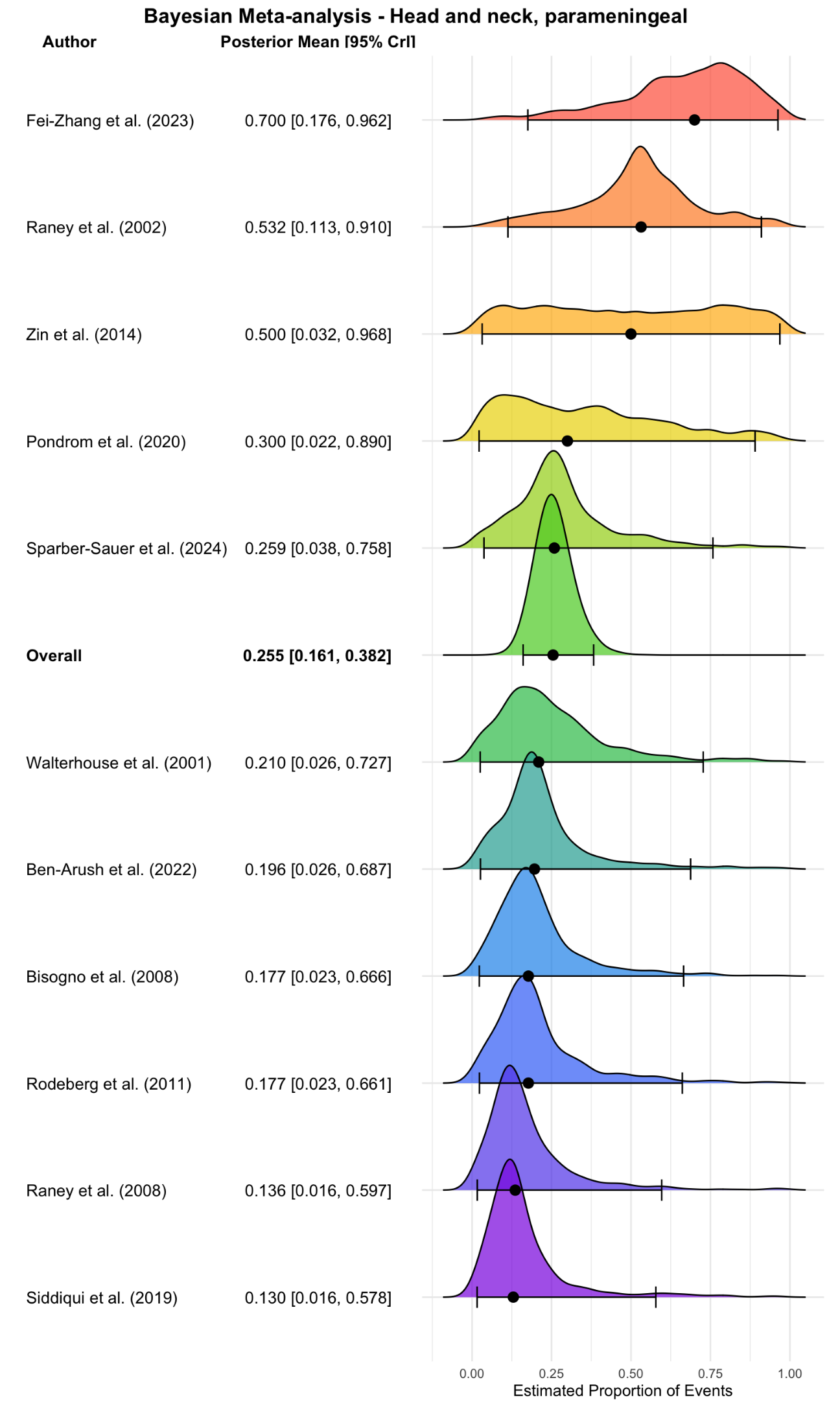


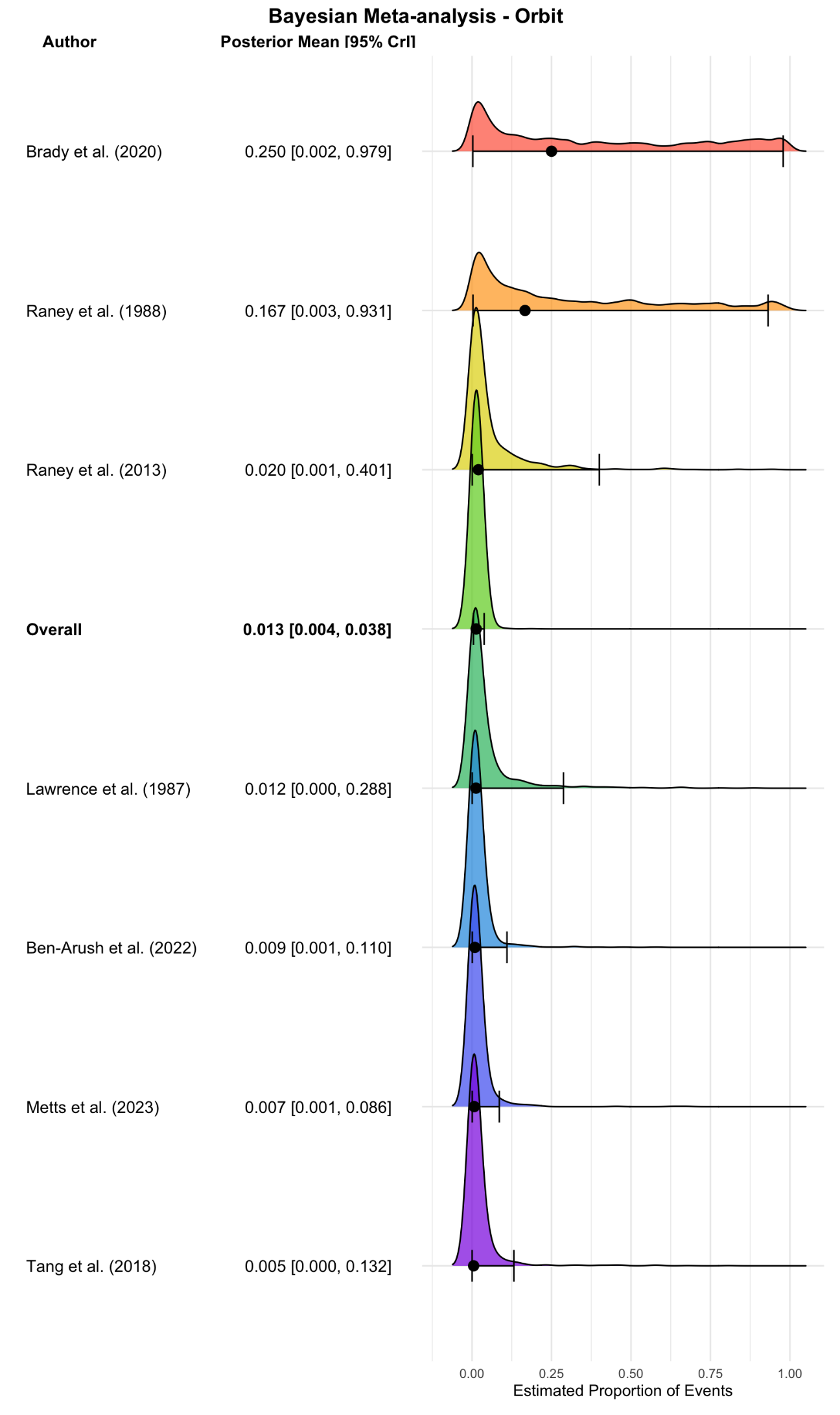


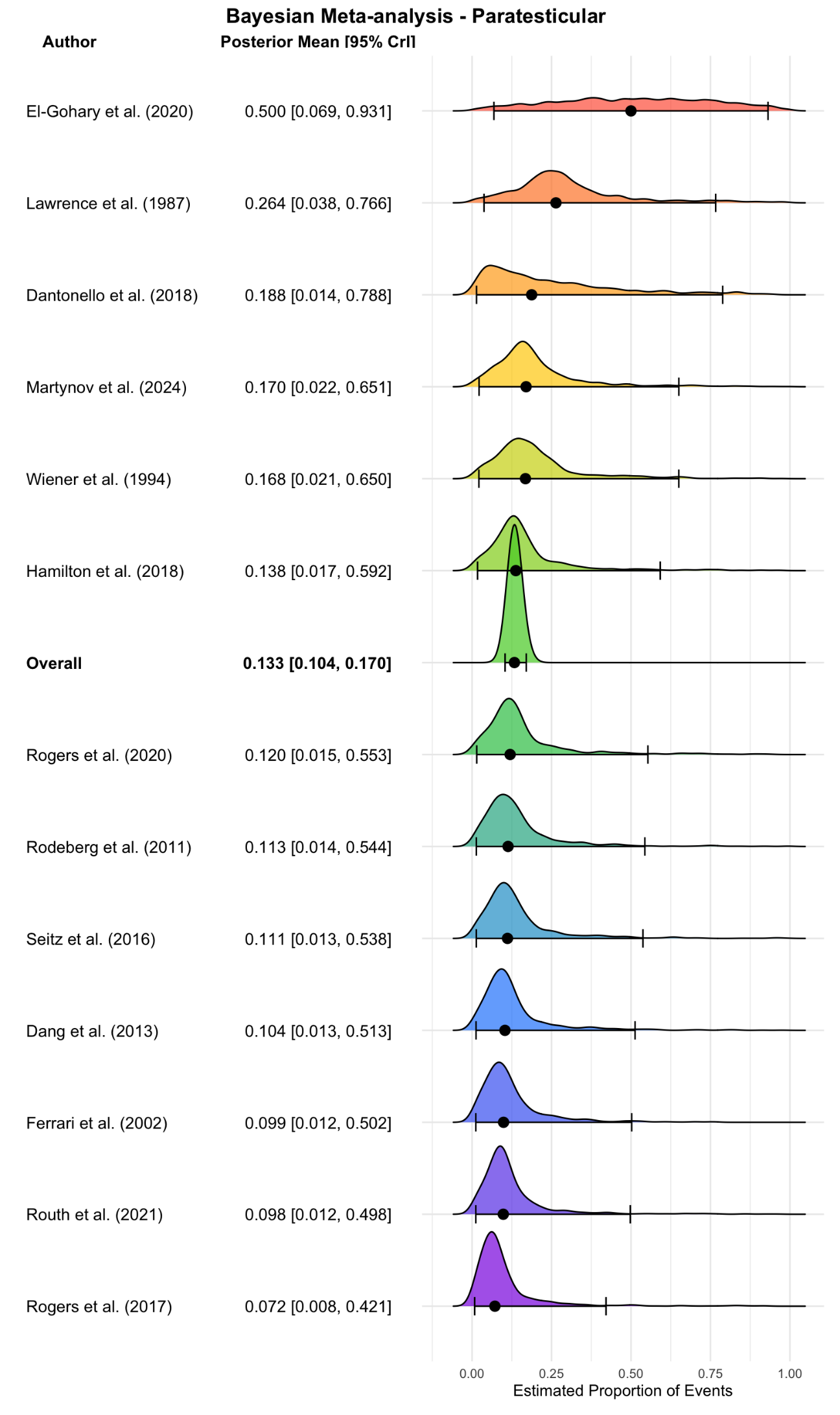


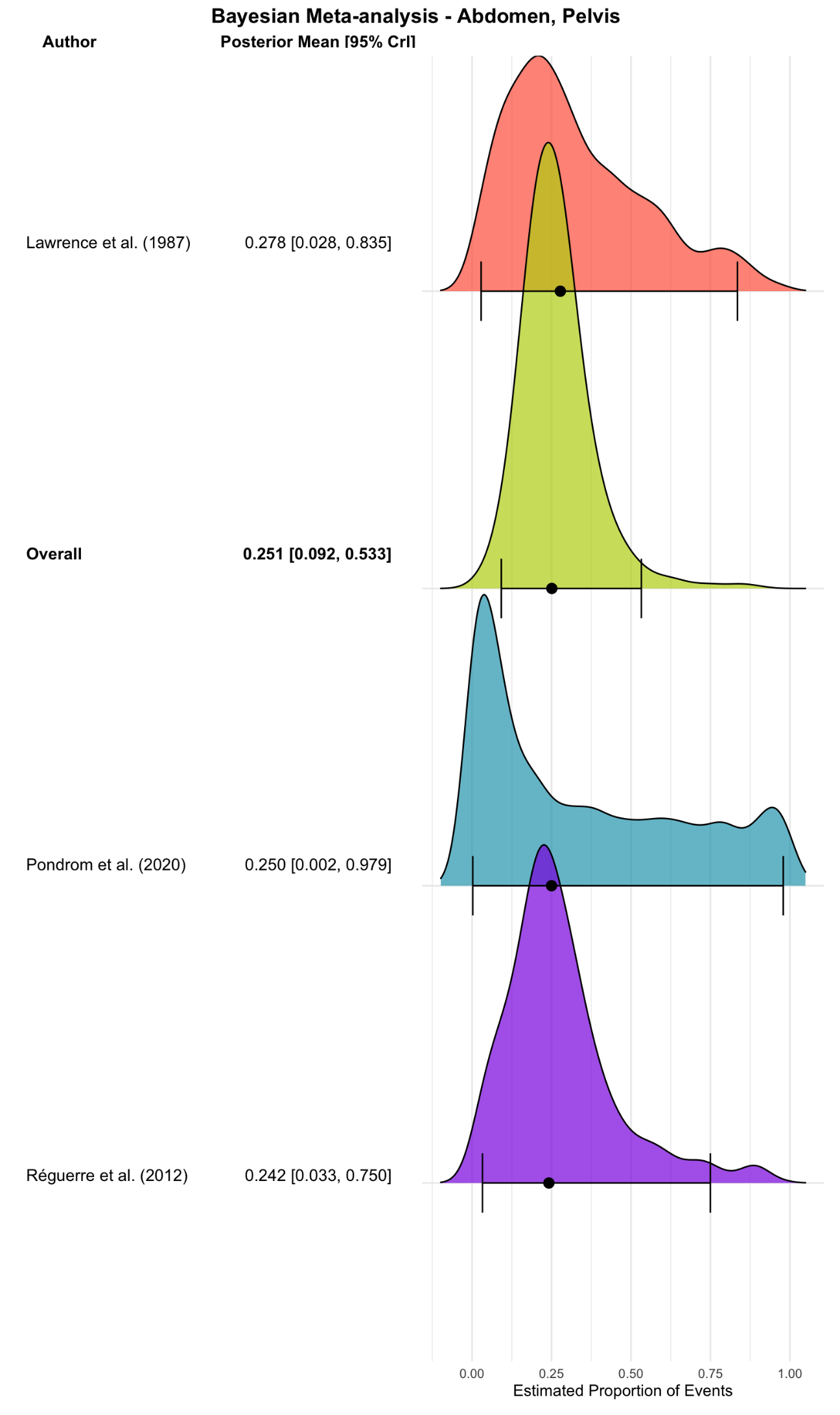


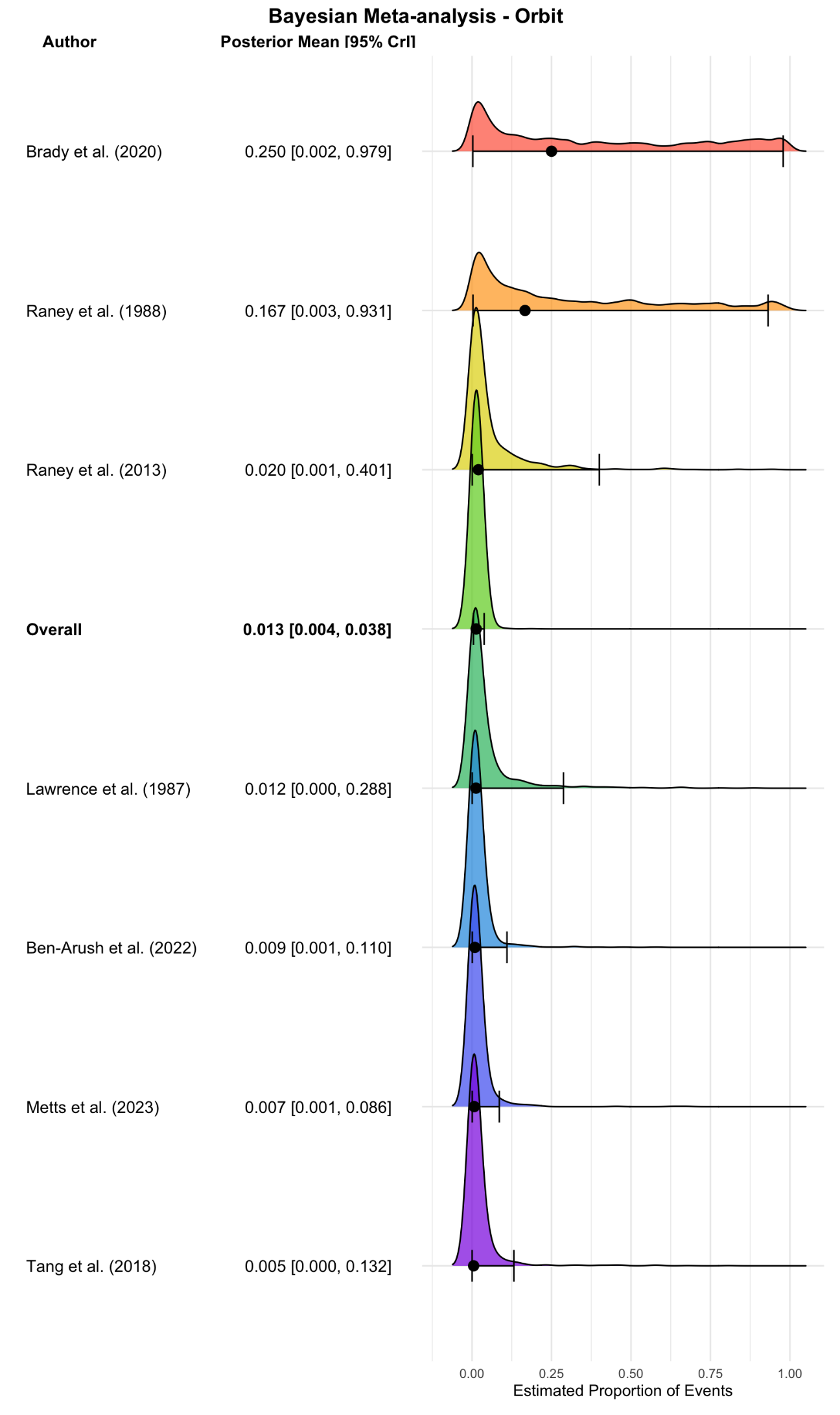


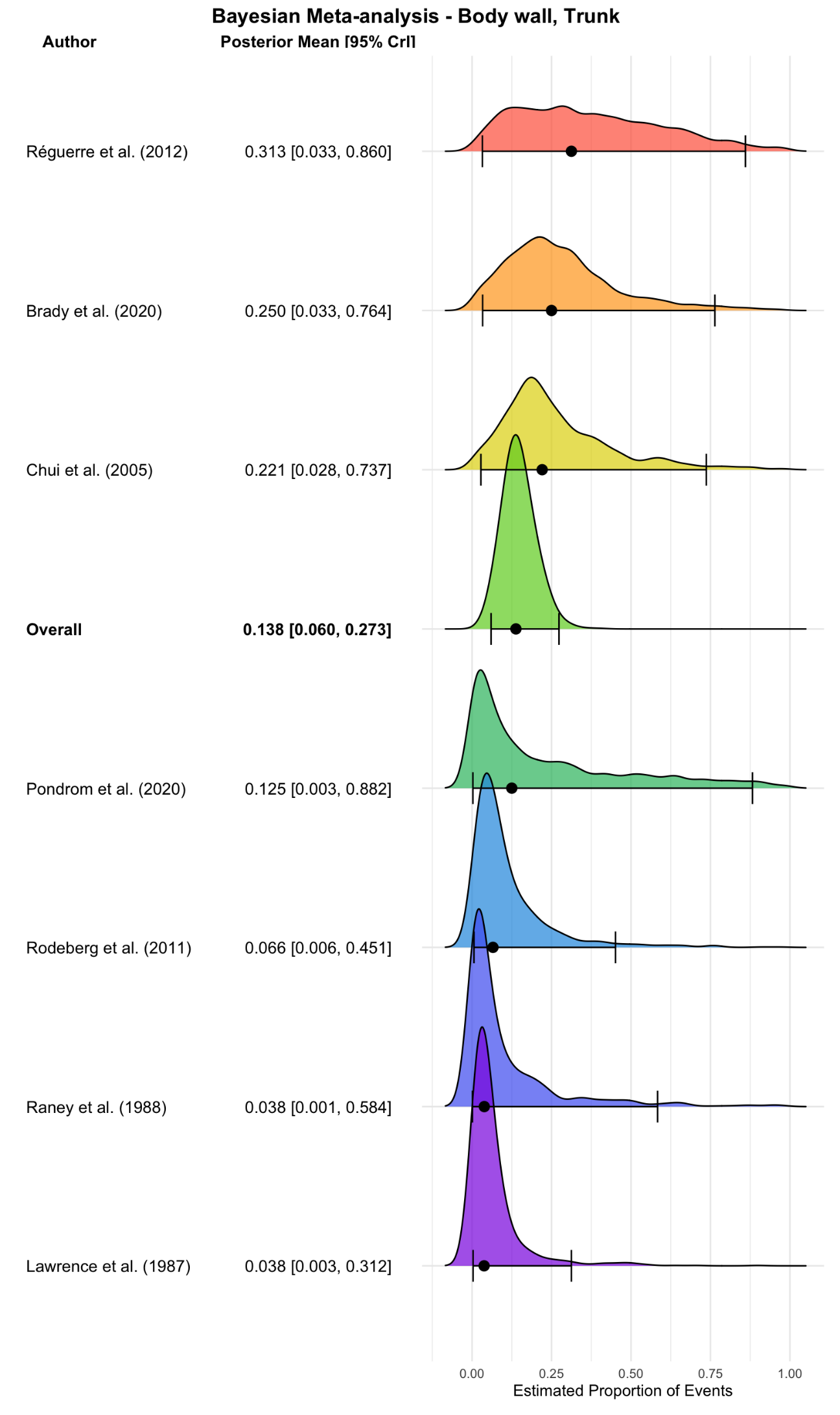


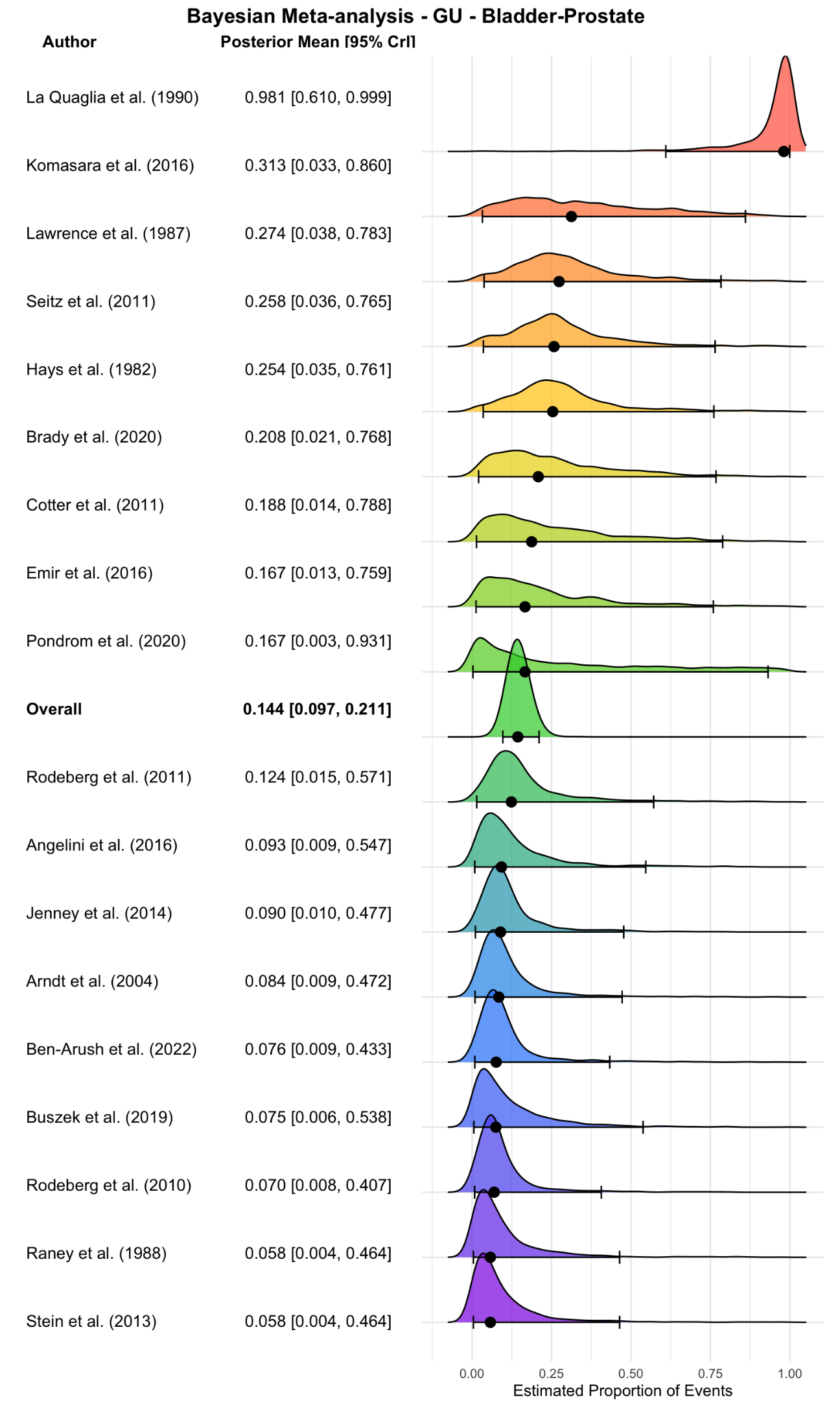

Supplement: Supplementary Fig. S4 [file mmc20.docx]

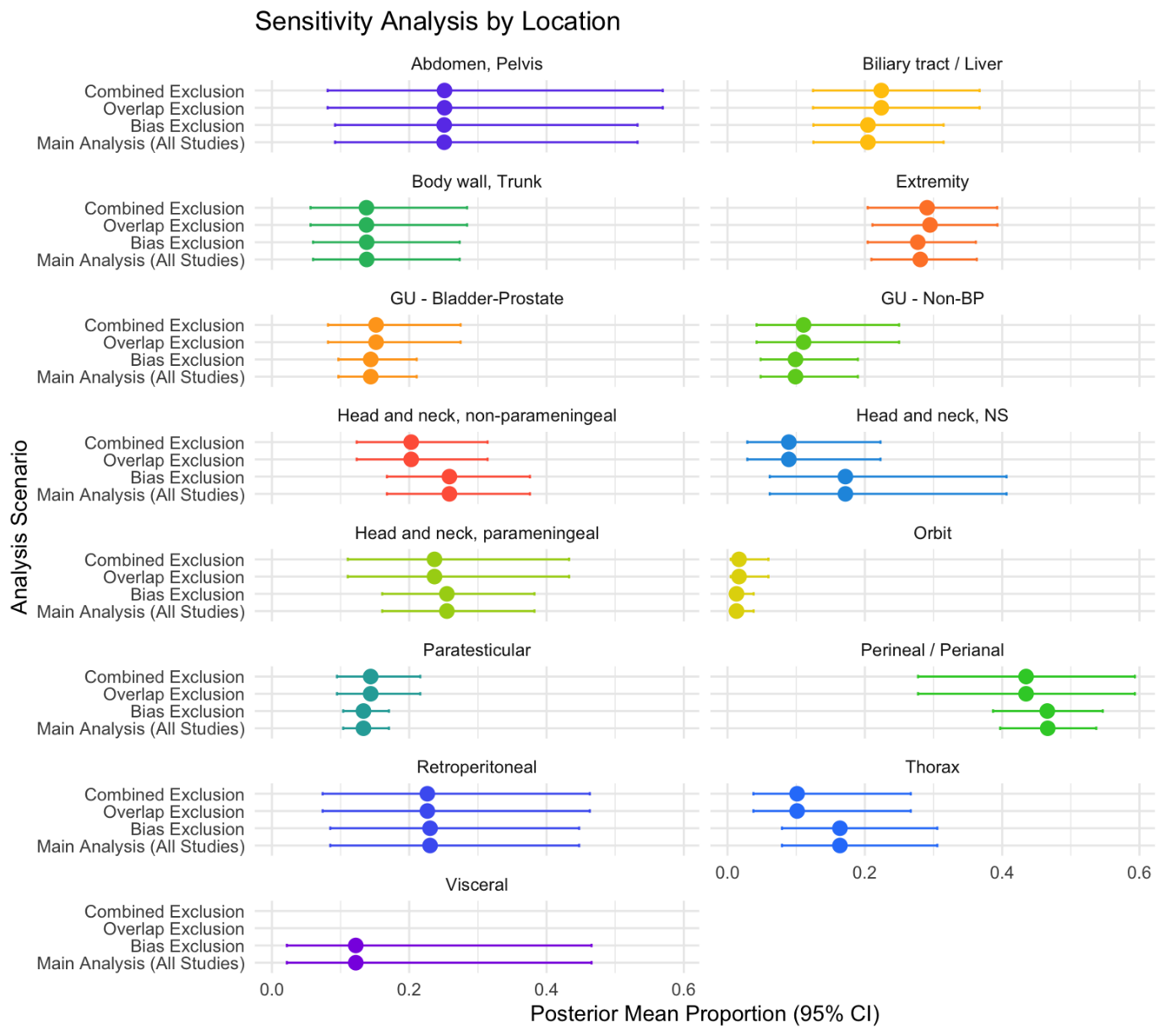

Supplement: Supplementary Fig. S5 [file mmc21.docx]

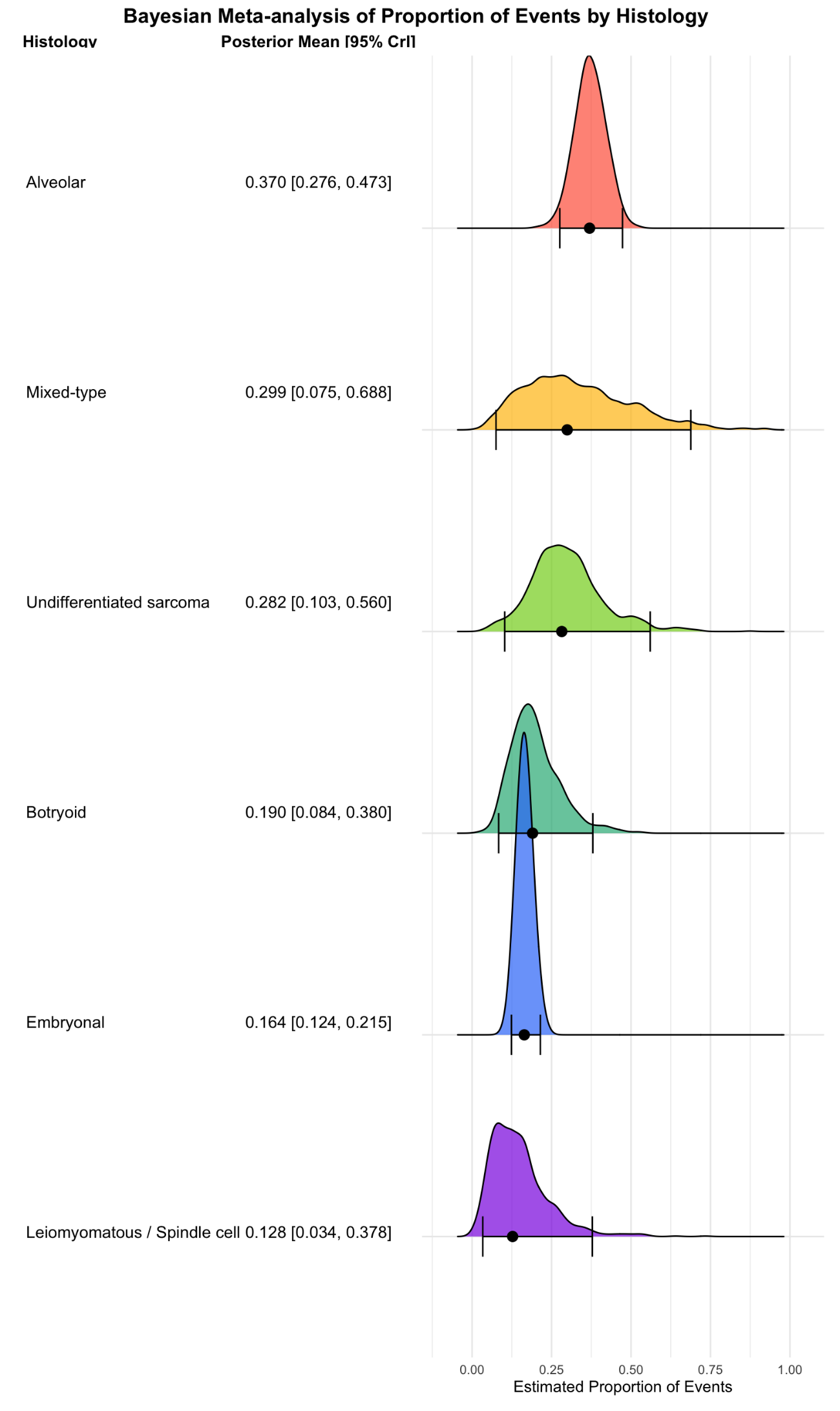

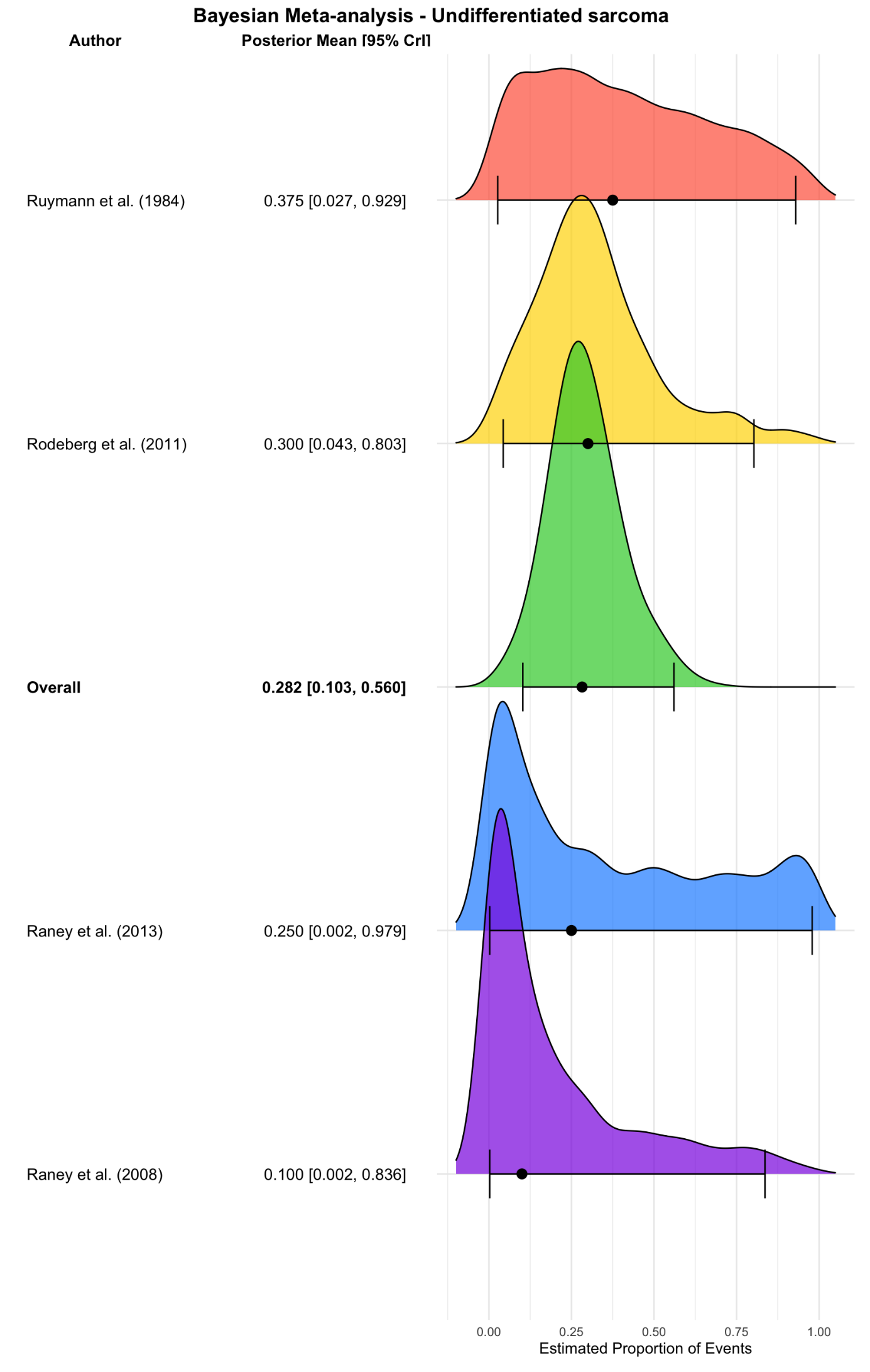

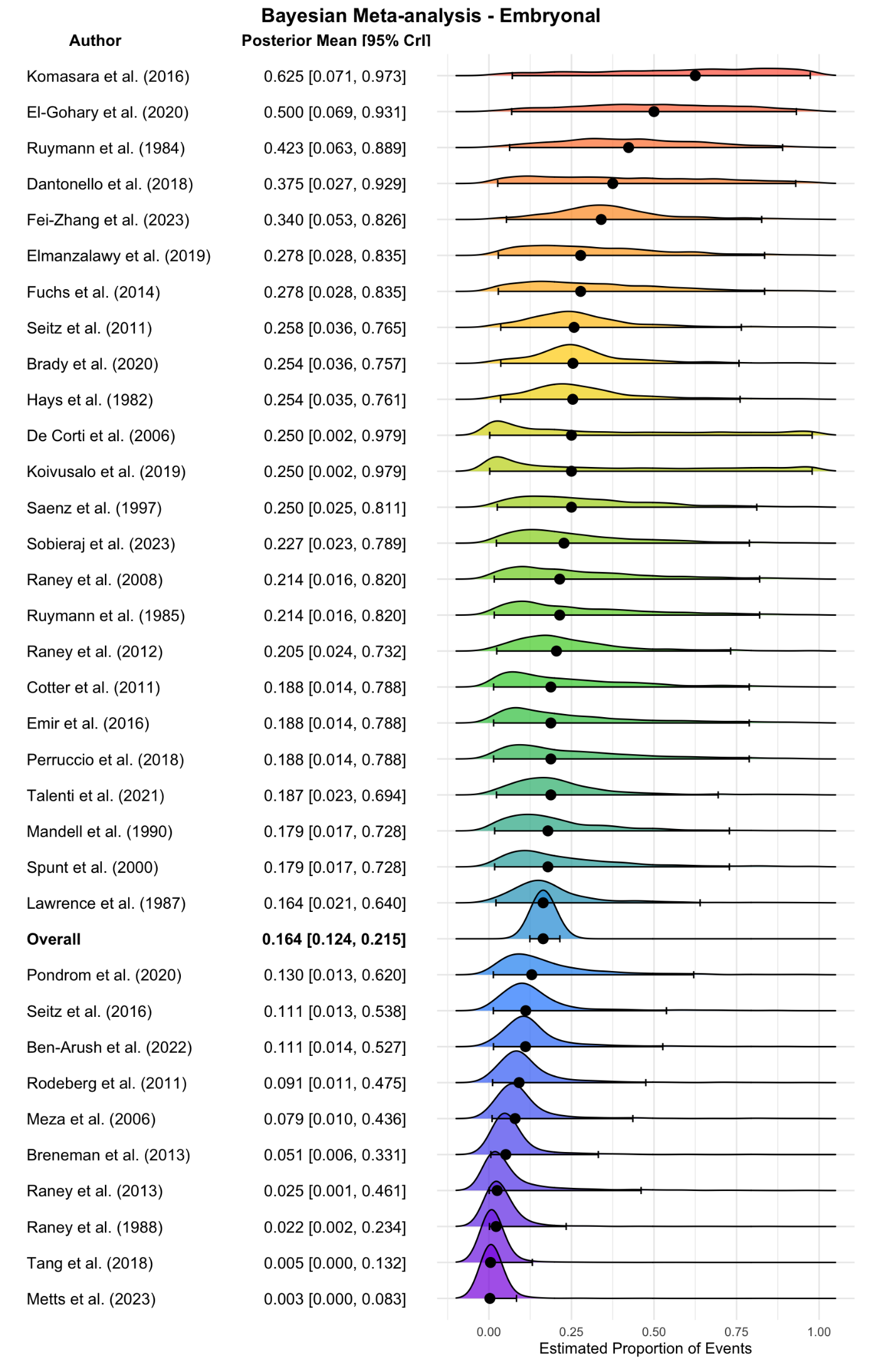


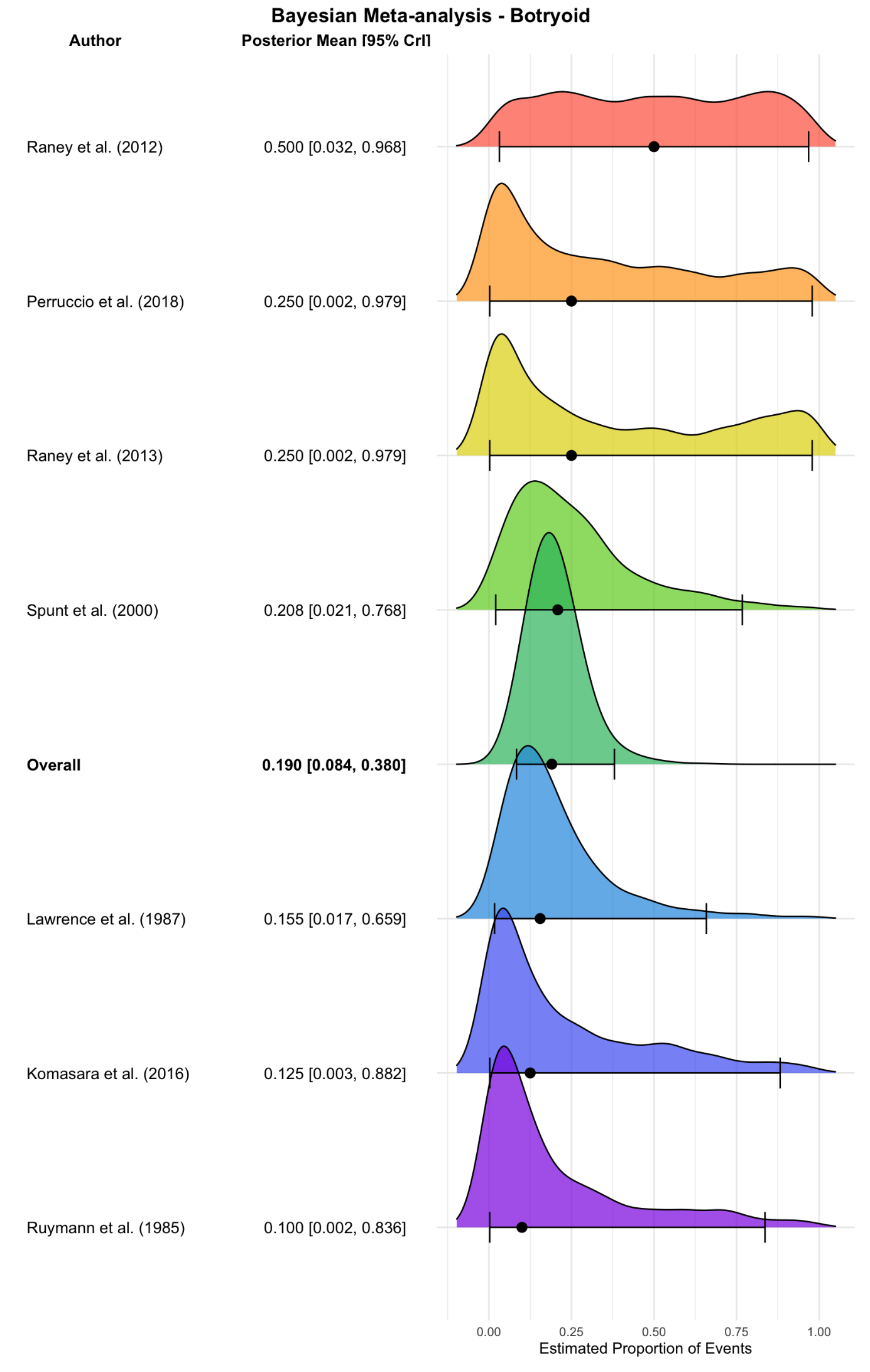


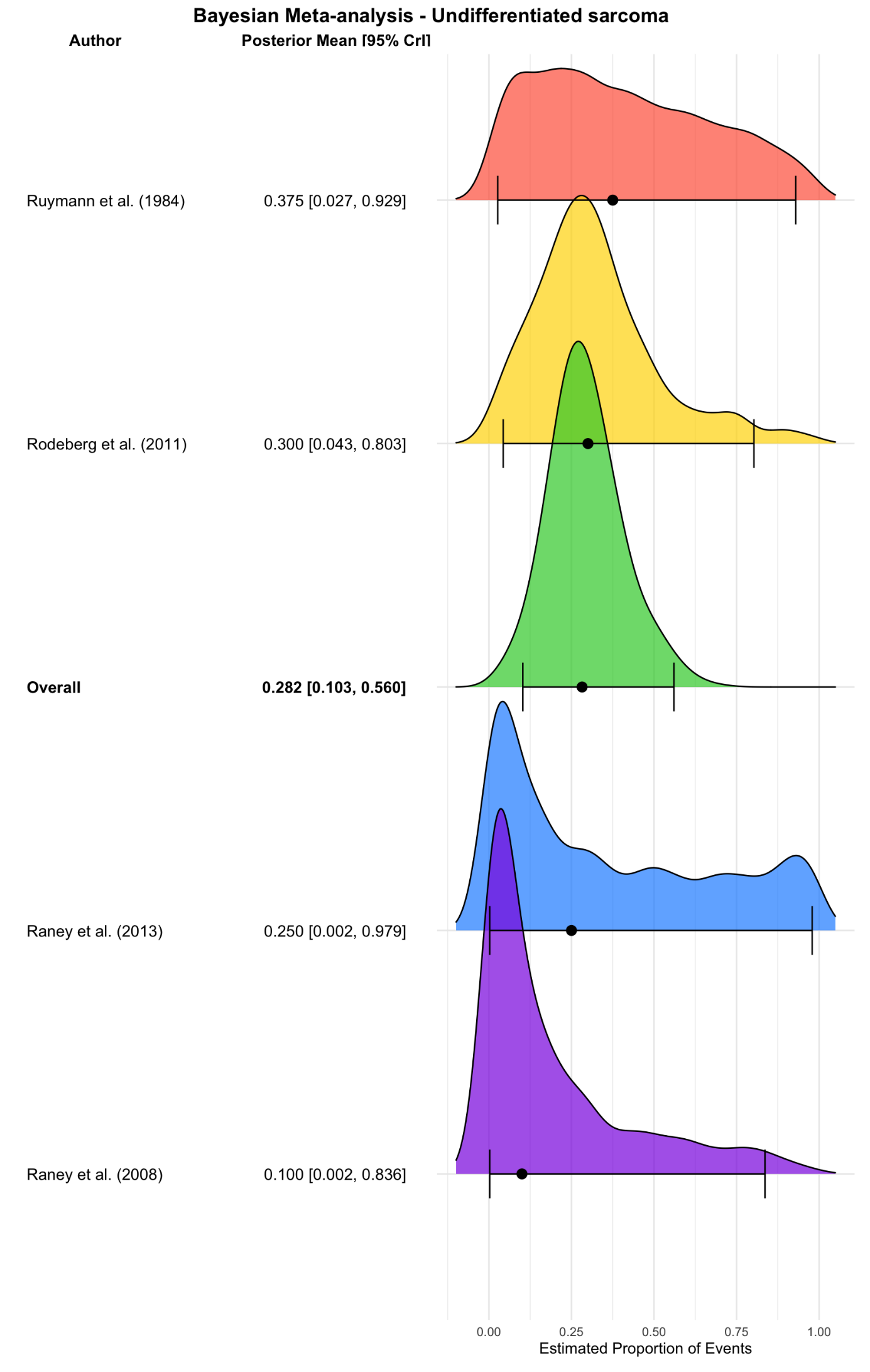


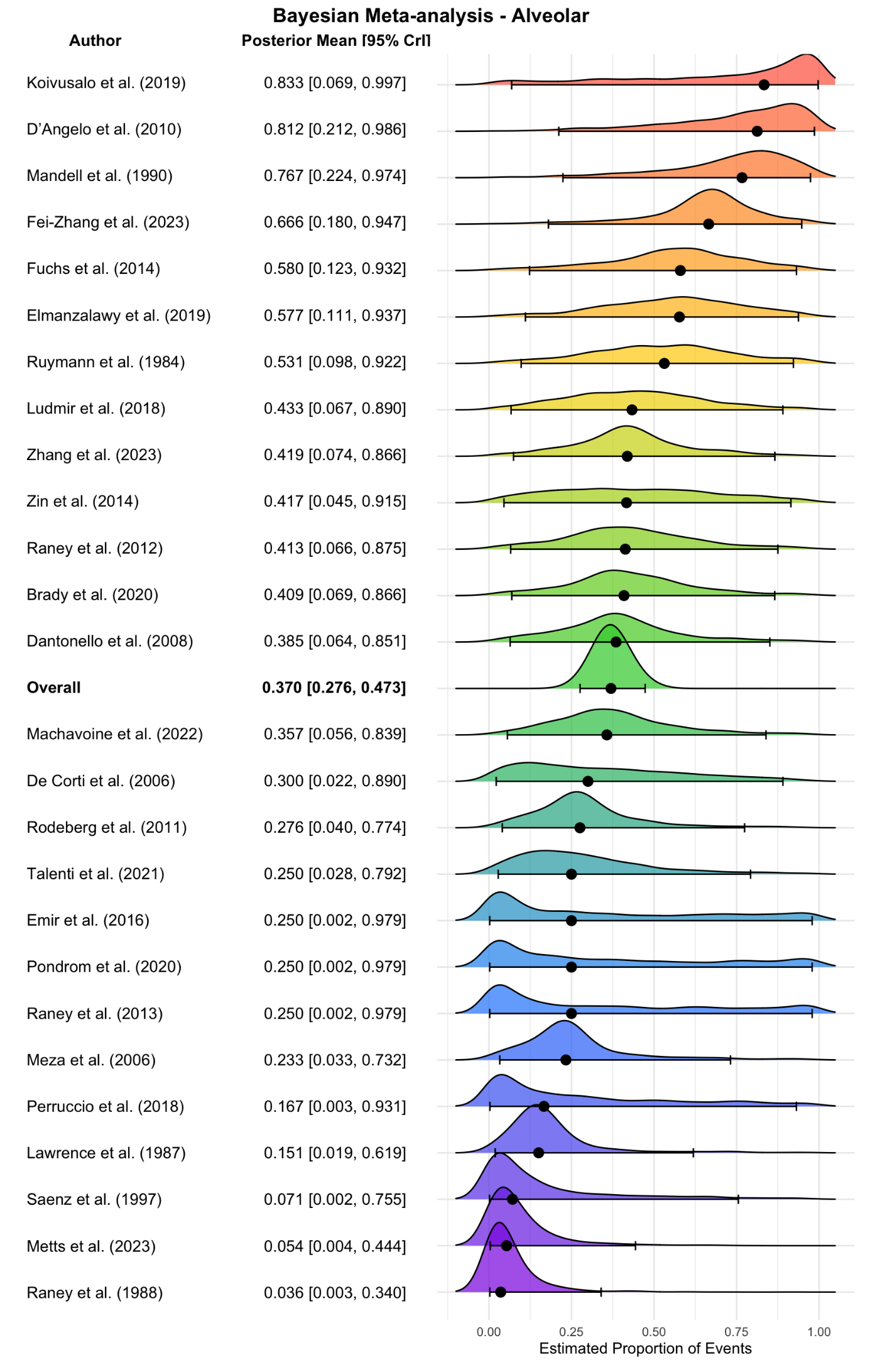


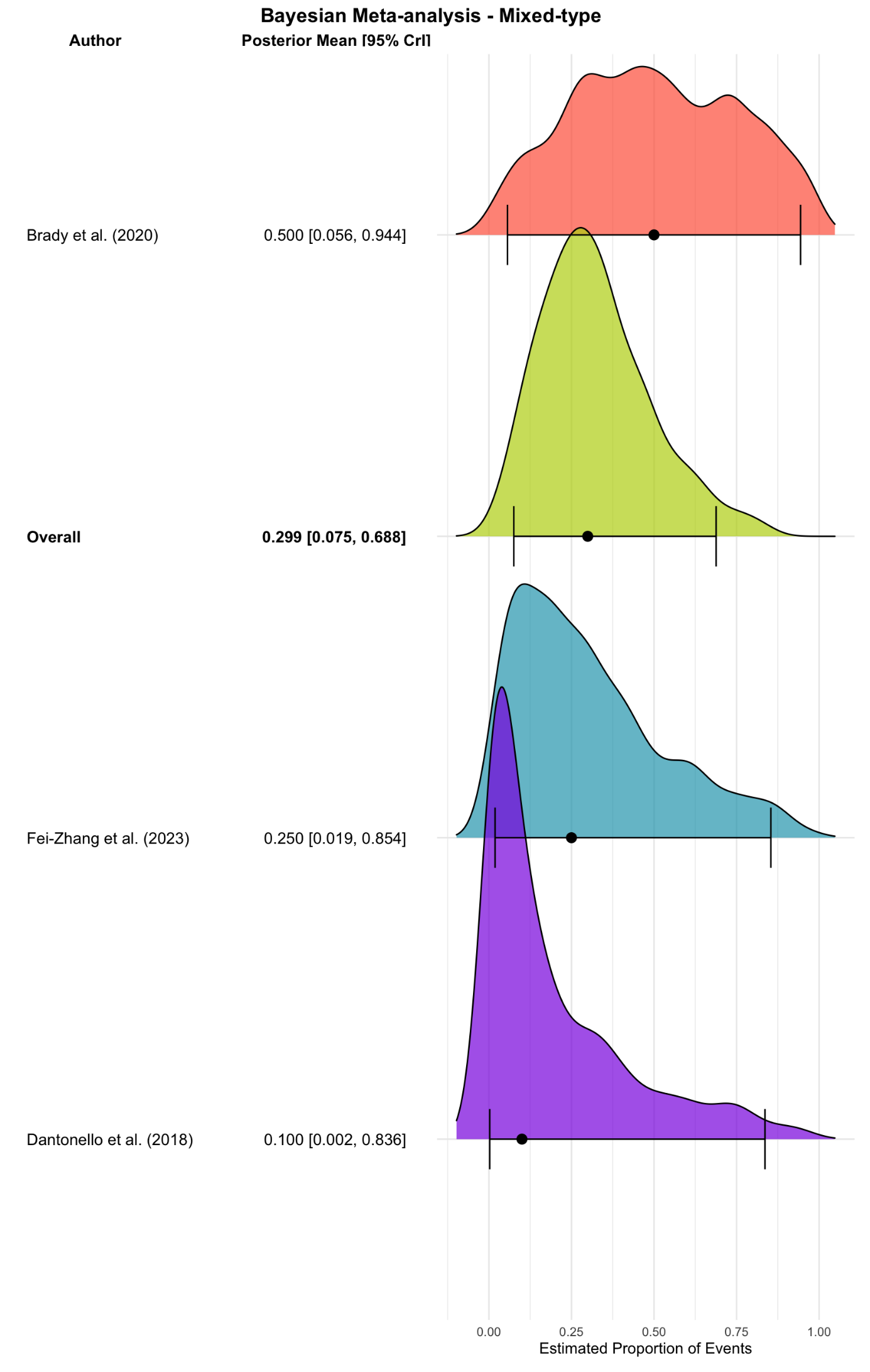

Supplement: Supplementary Fig. S6 [file mmc22.docx]

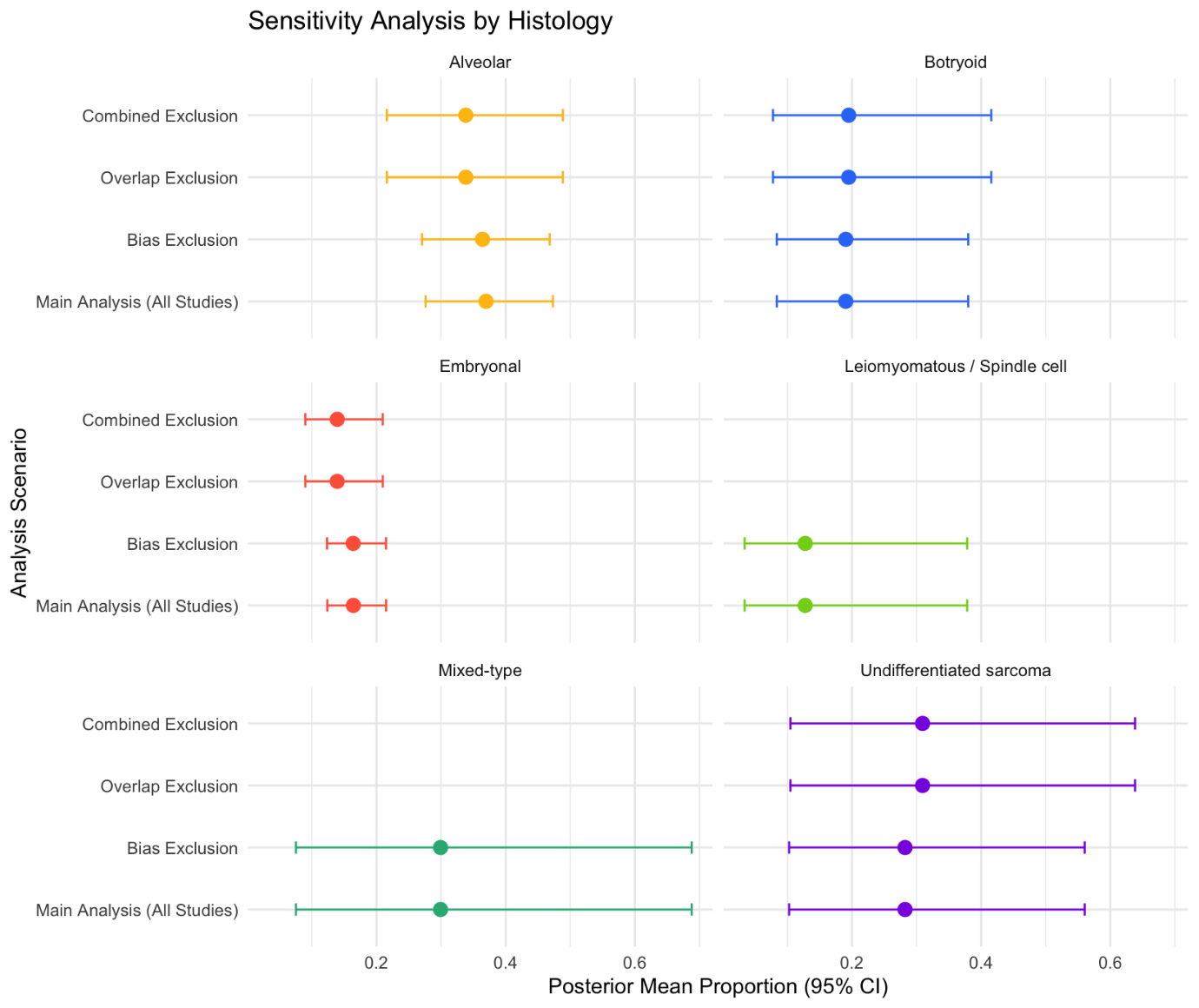

Supplement: Supplementary Fig. S8 [file mmc23.docx]

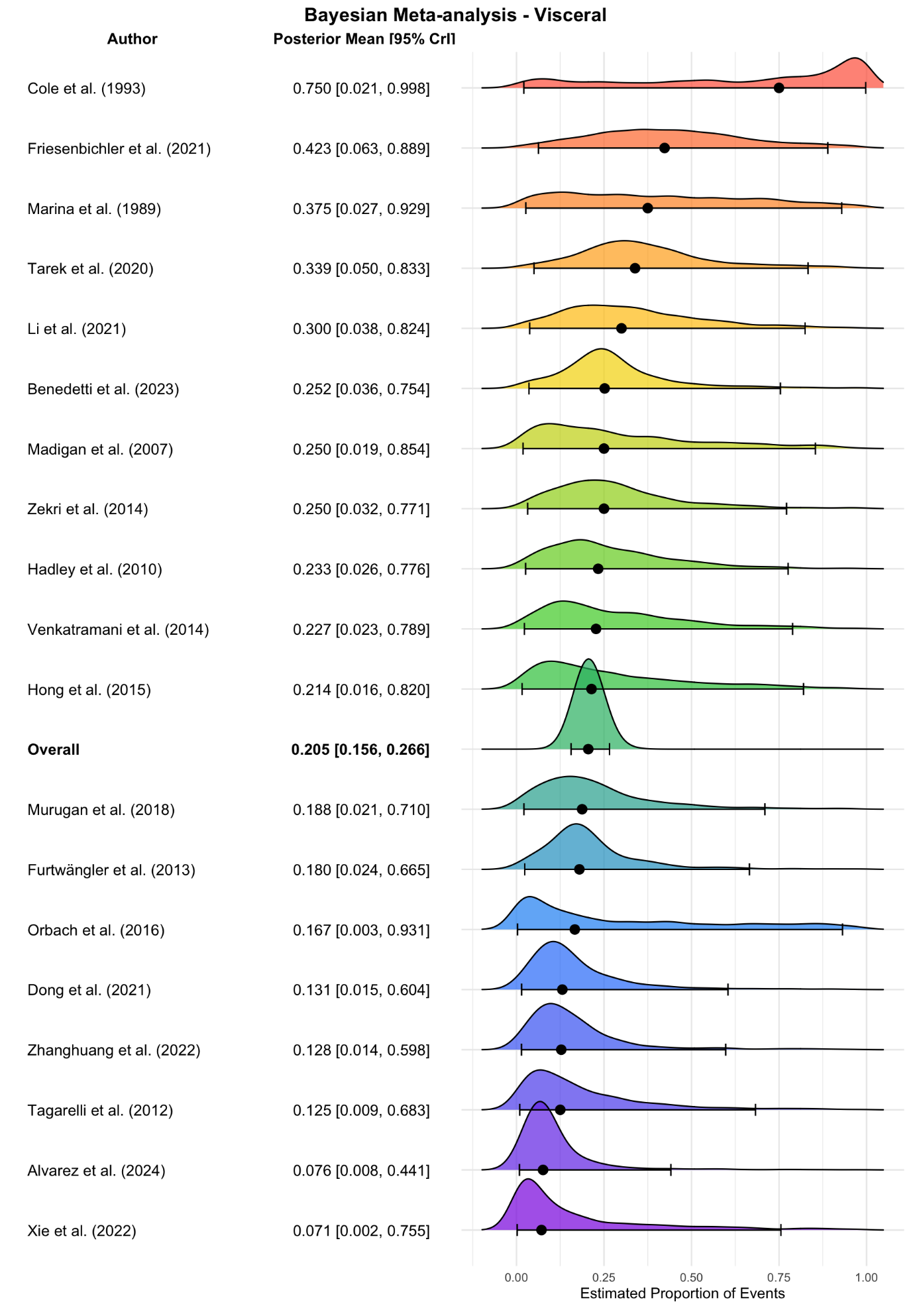

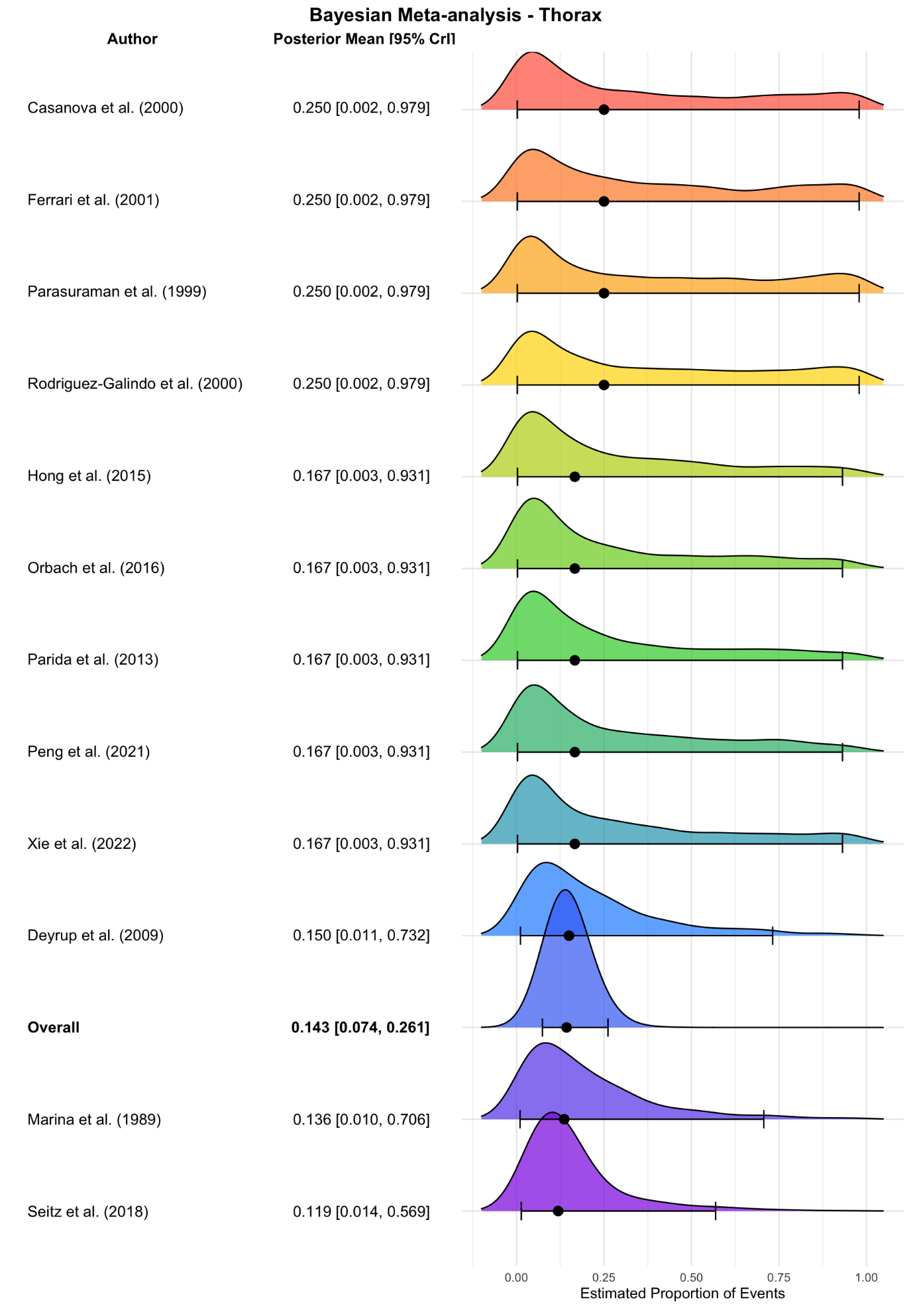

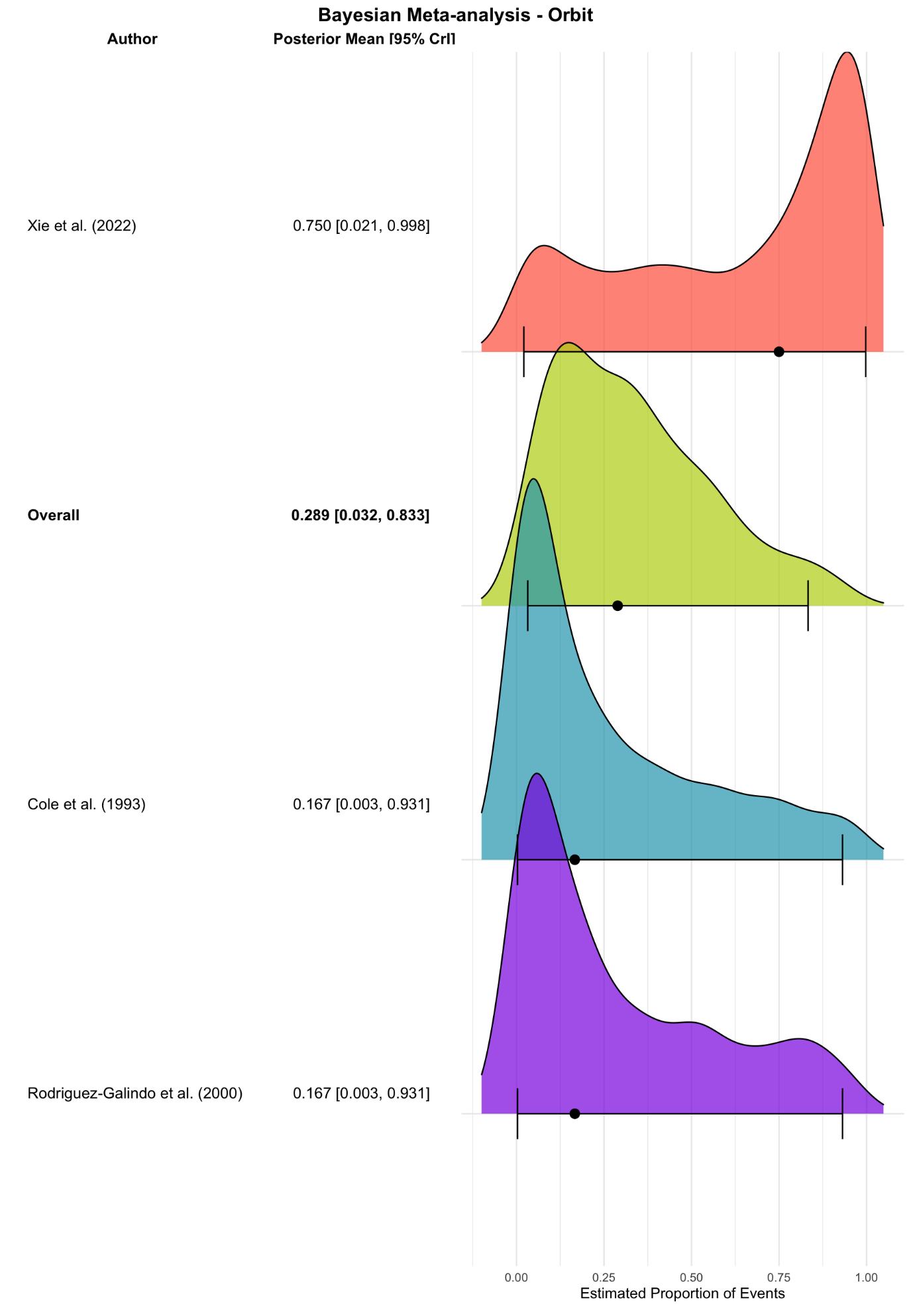

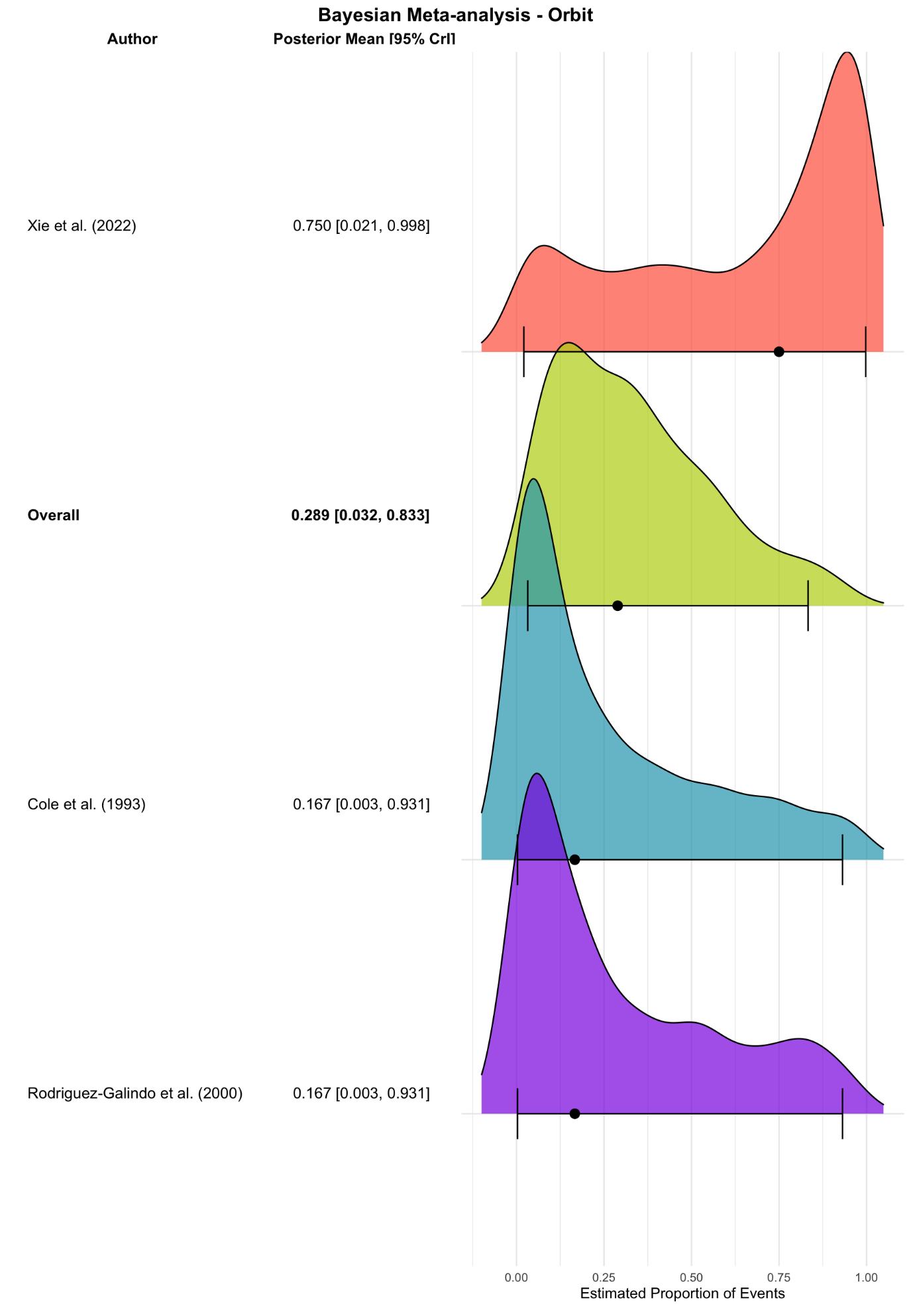

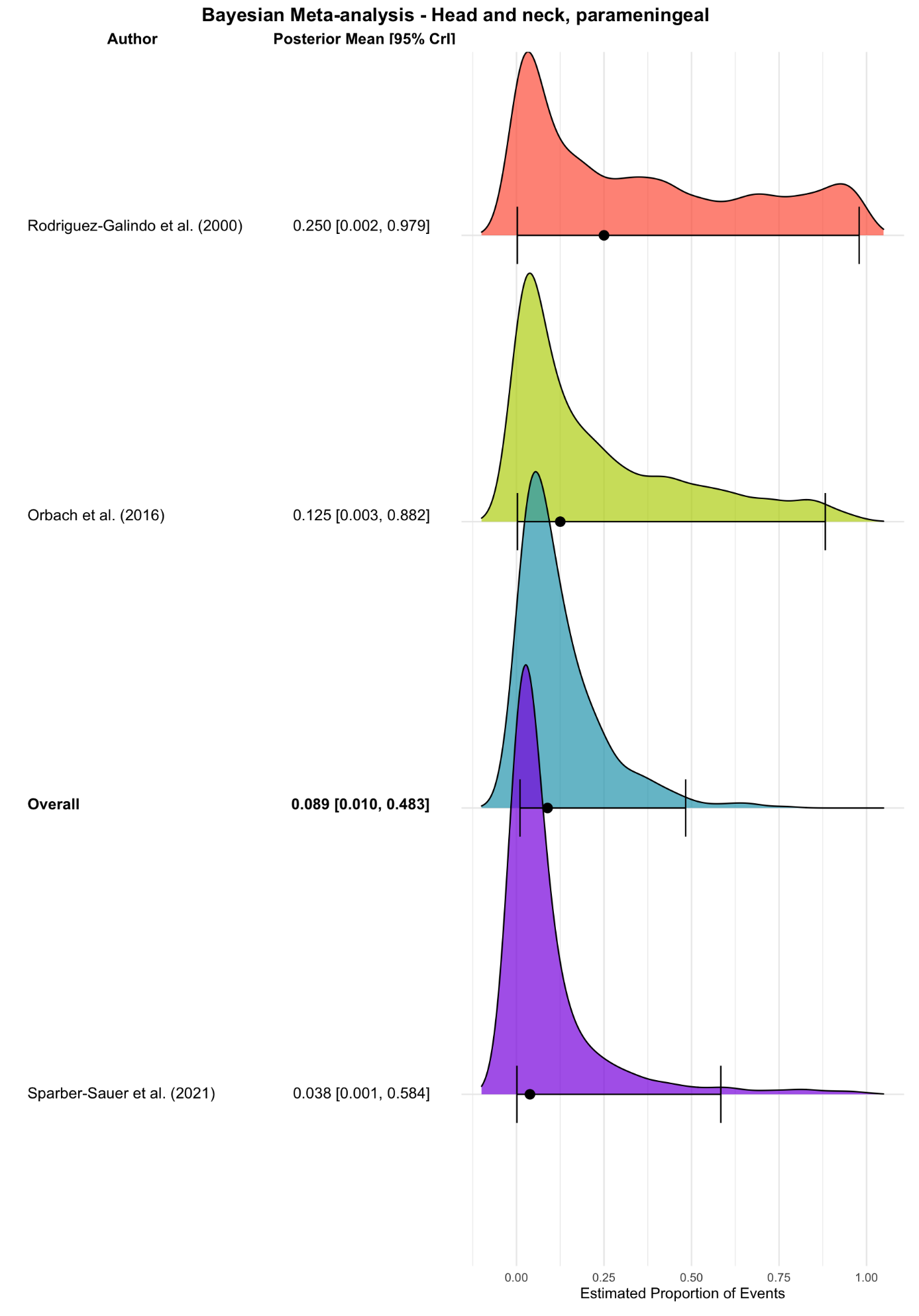

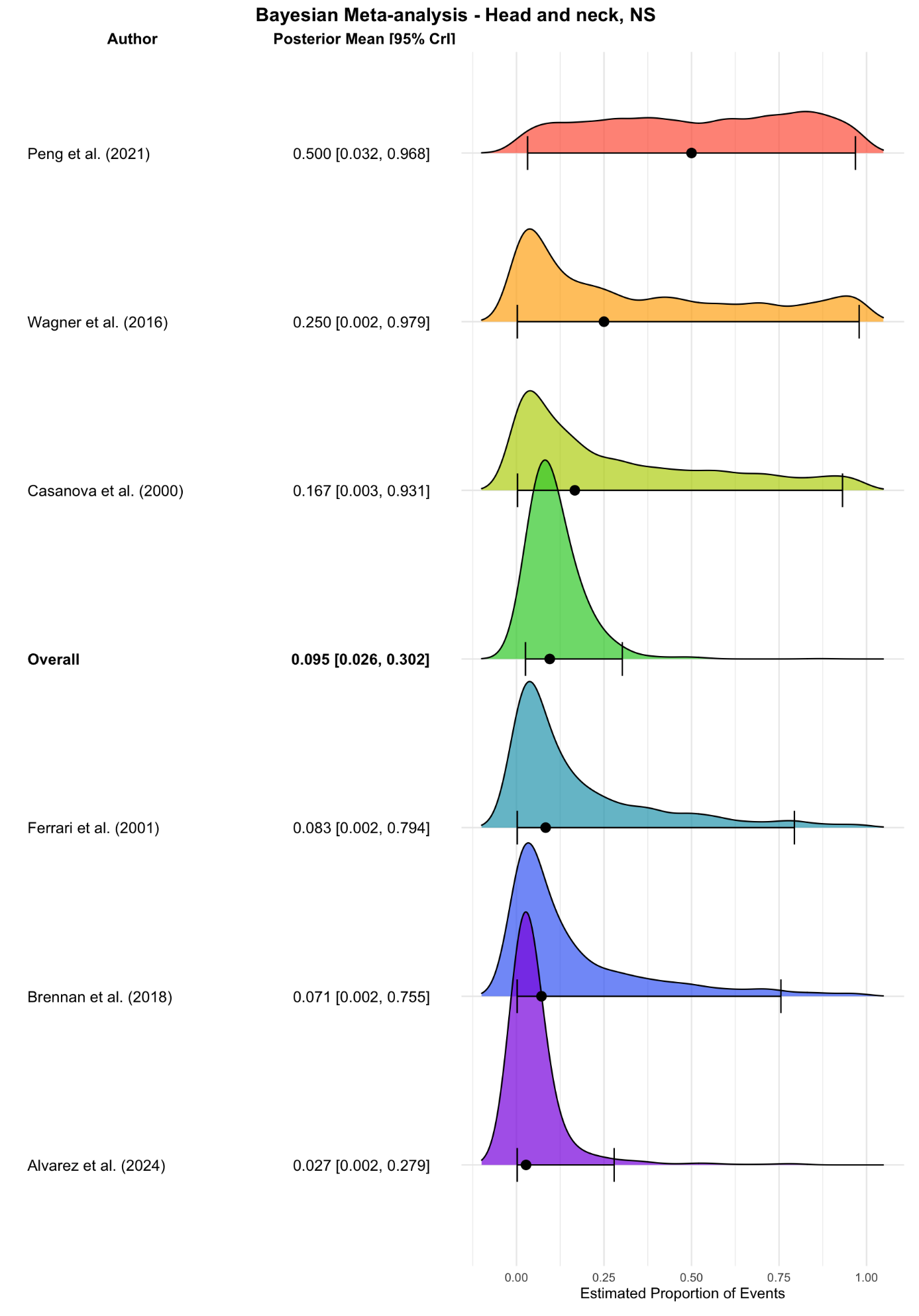

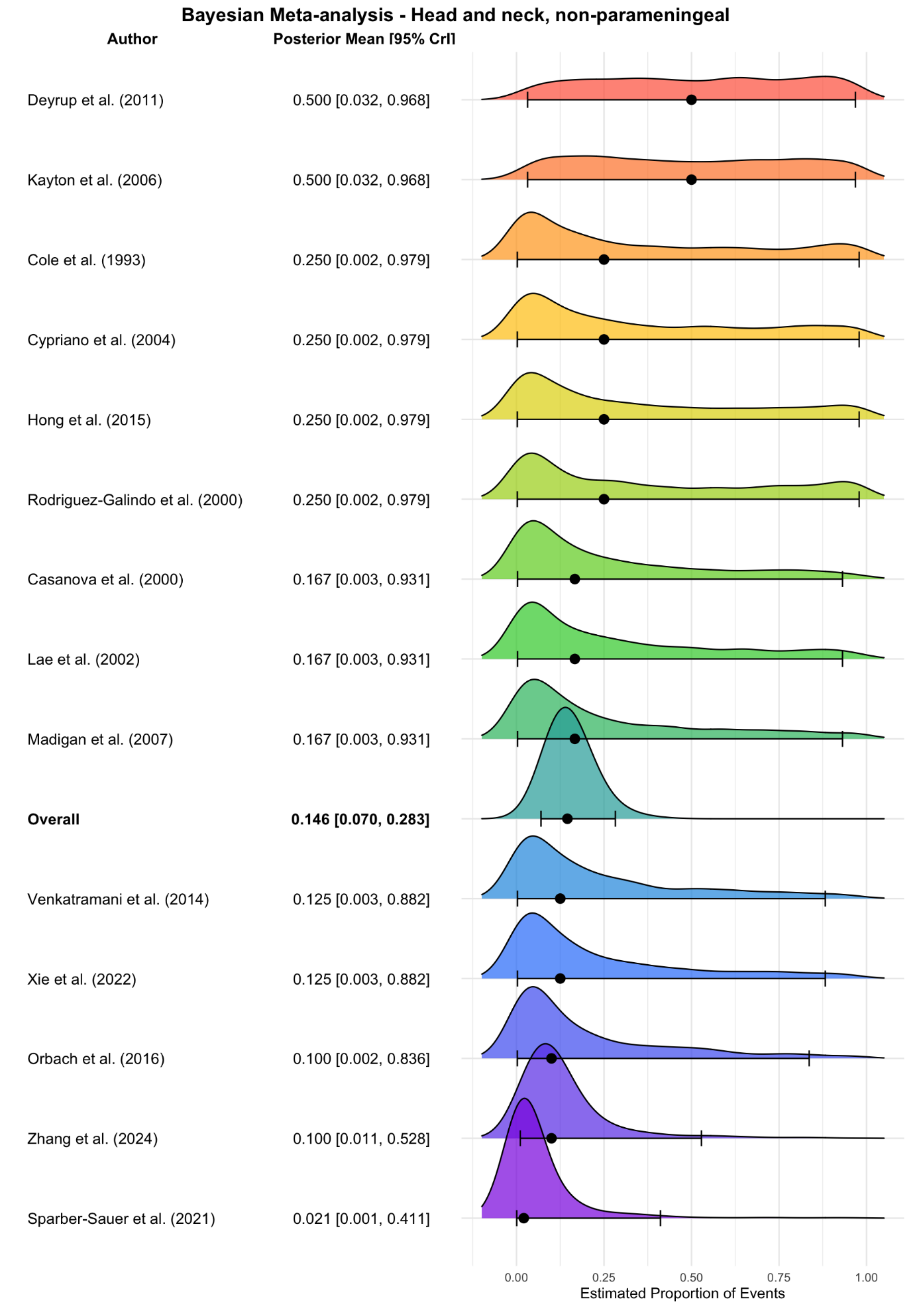

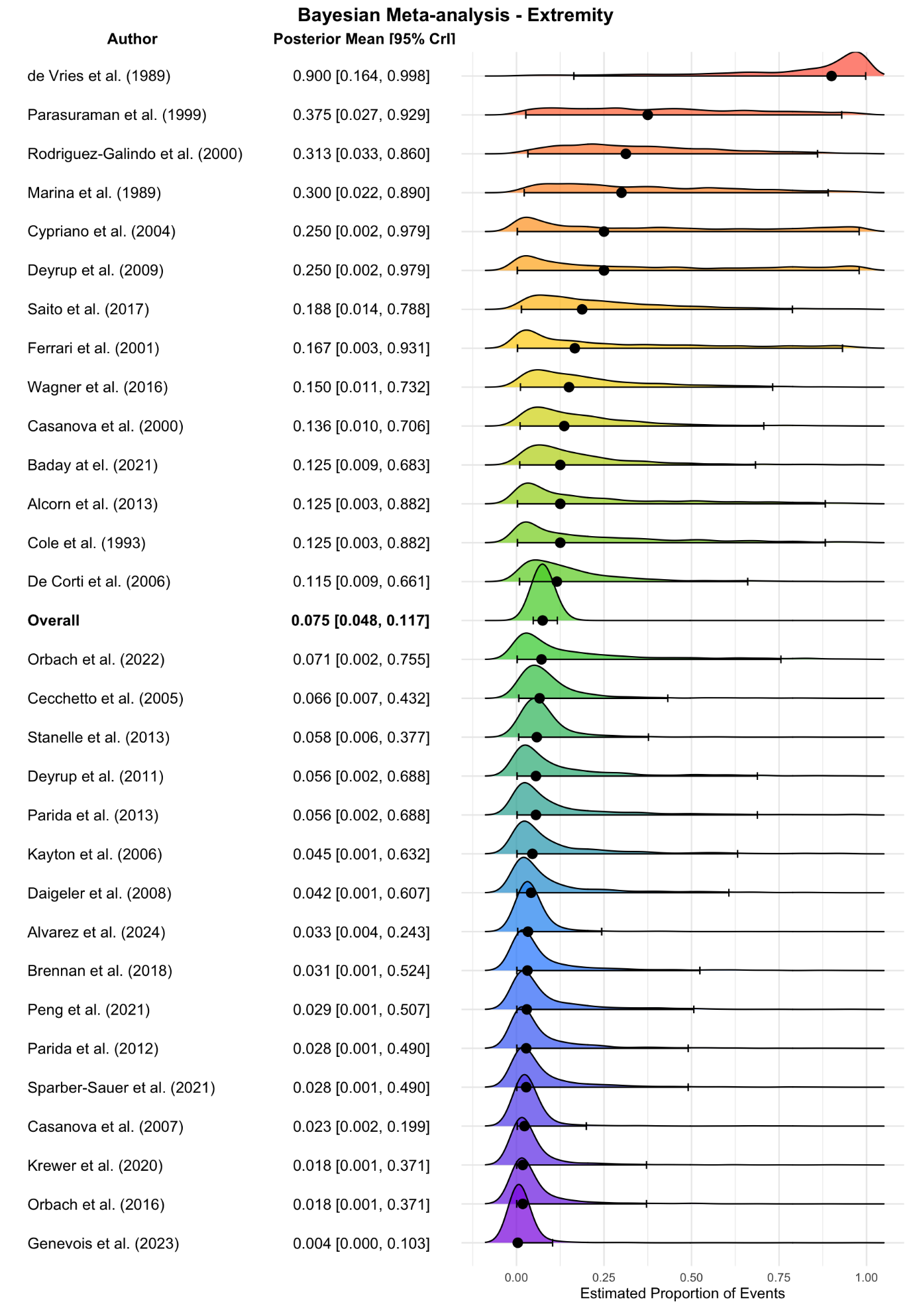

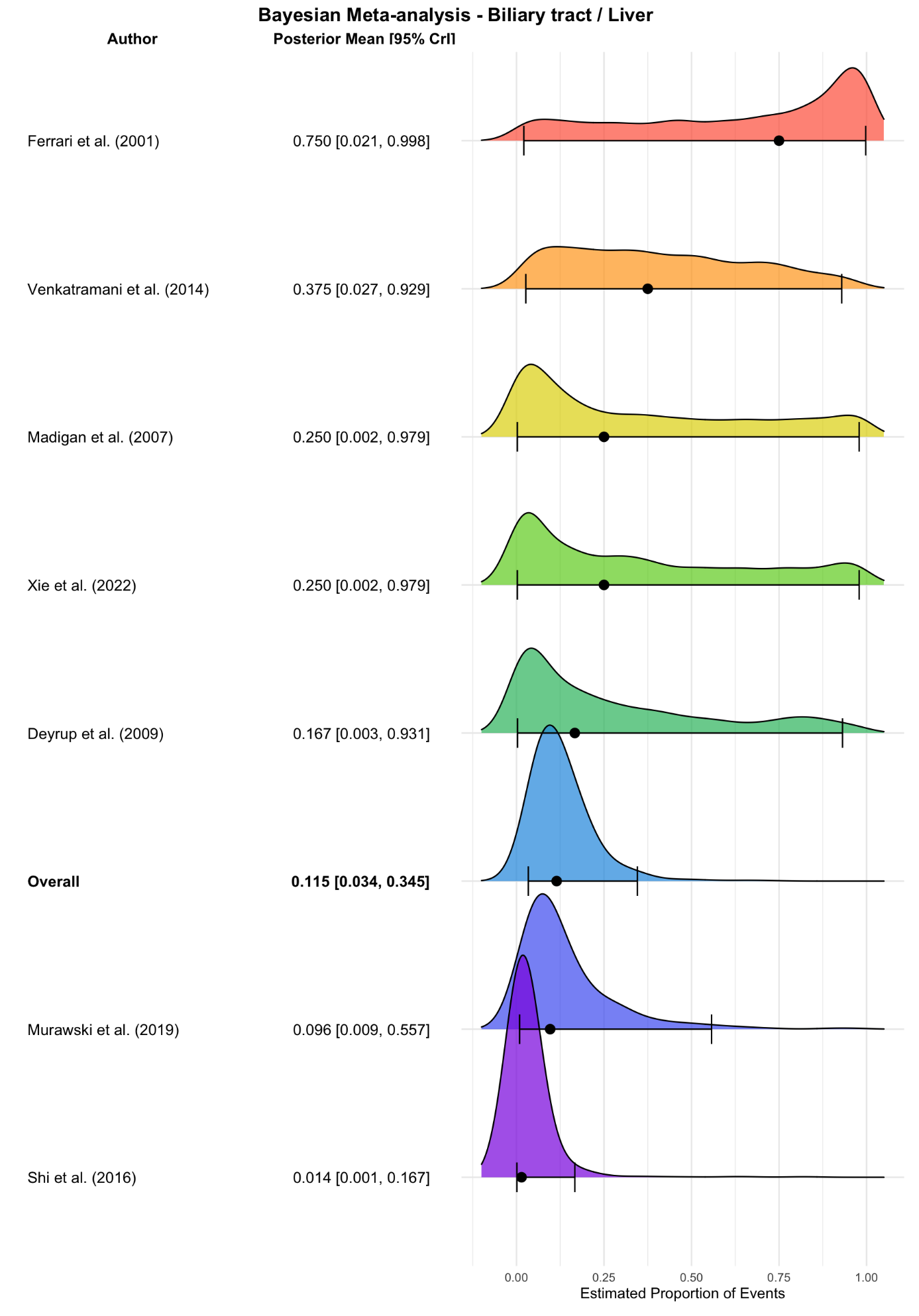

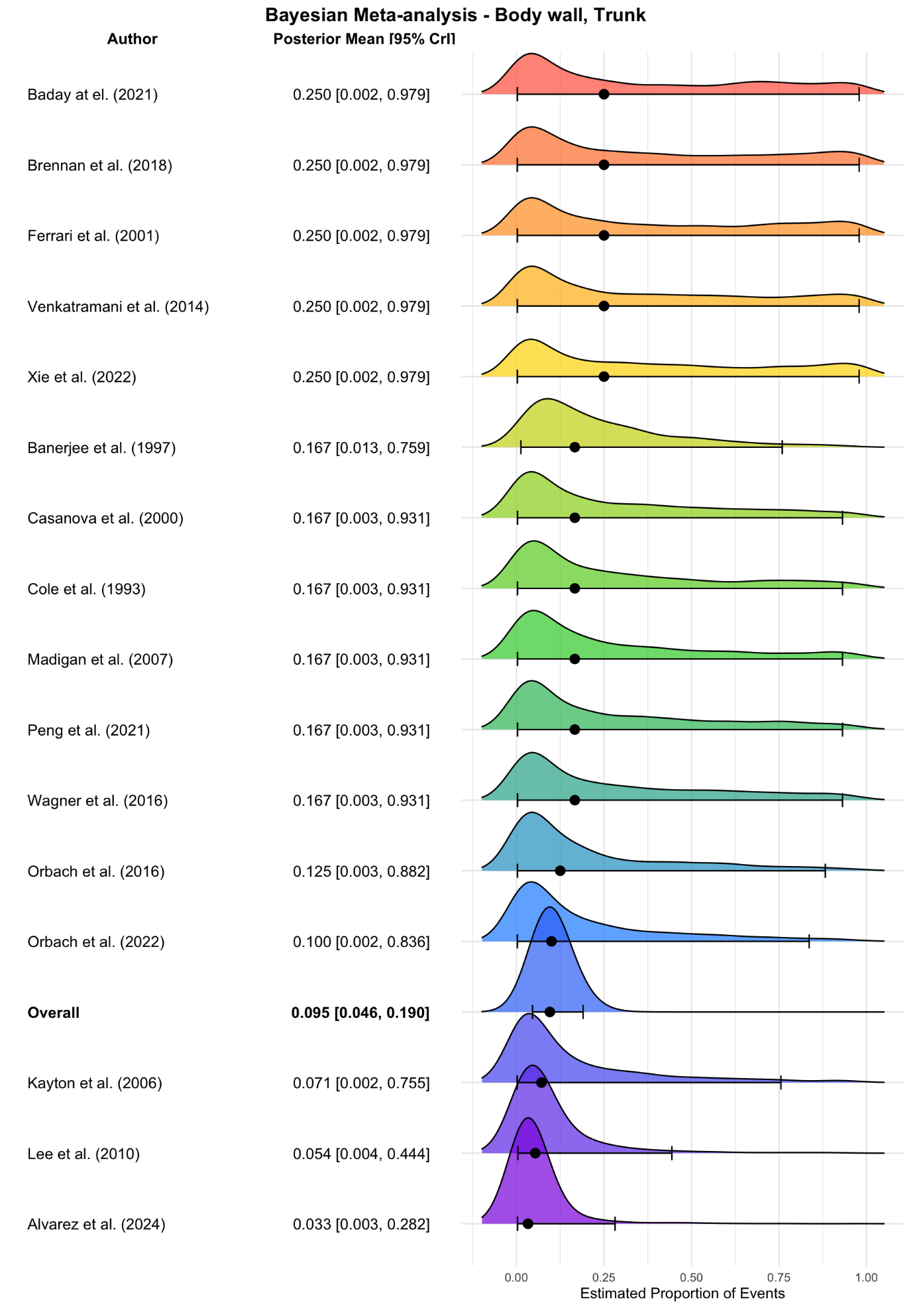

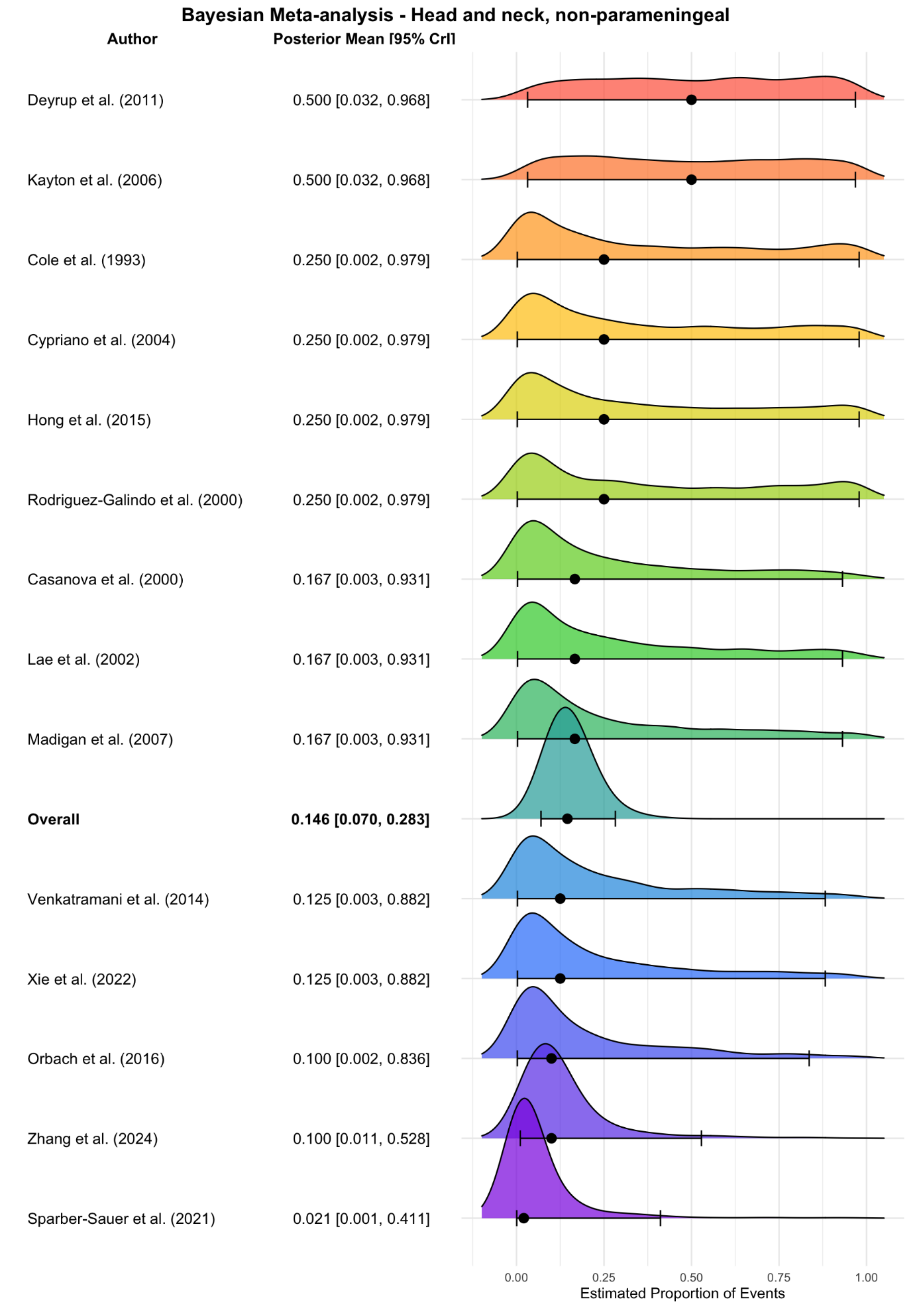

Supplement: Supplementary Fig. S9 [file mmc24.docx]

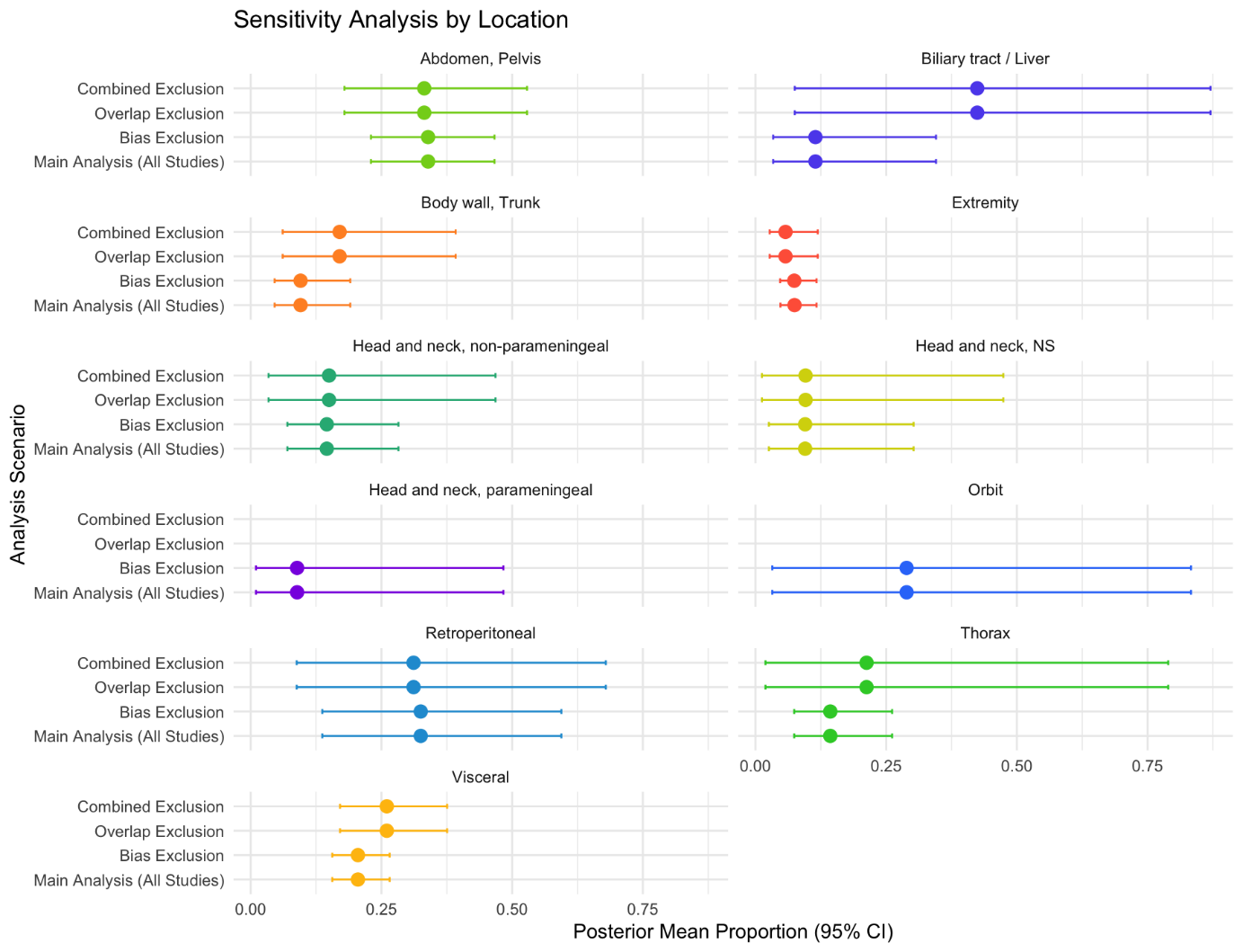

Supplement: Supplementary Fig. S10 [file mmc25.docx]

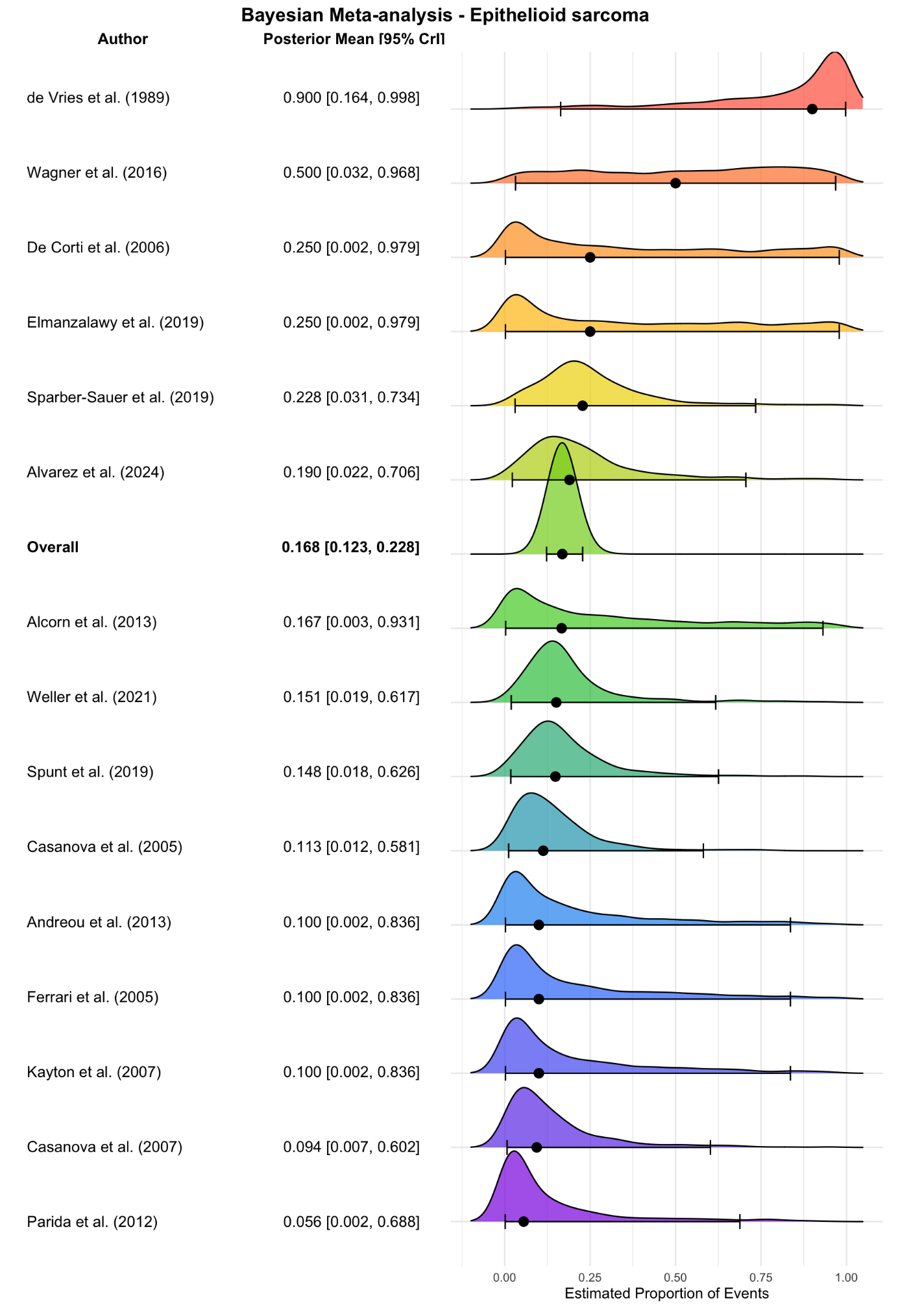

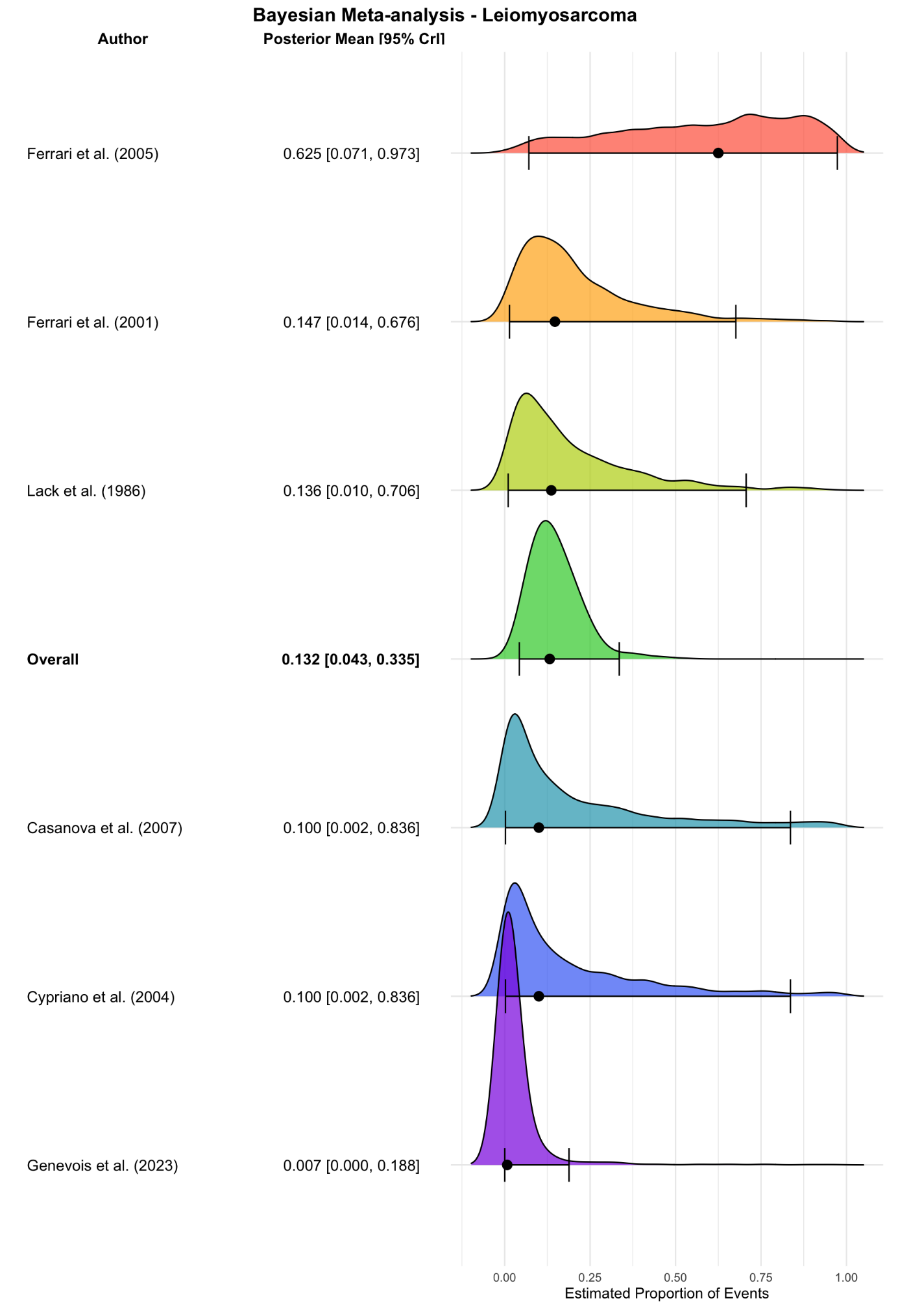

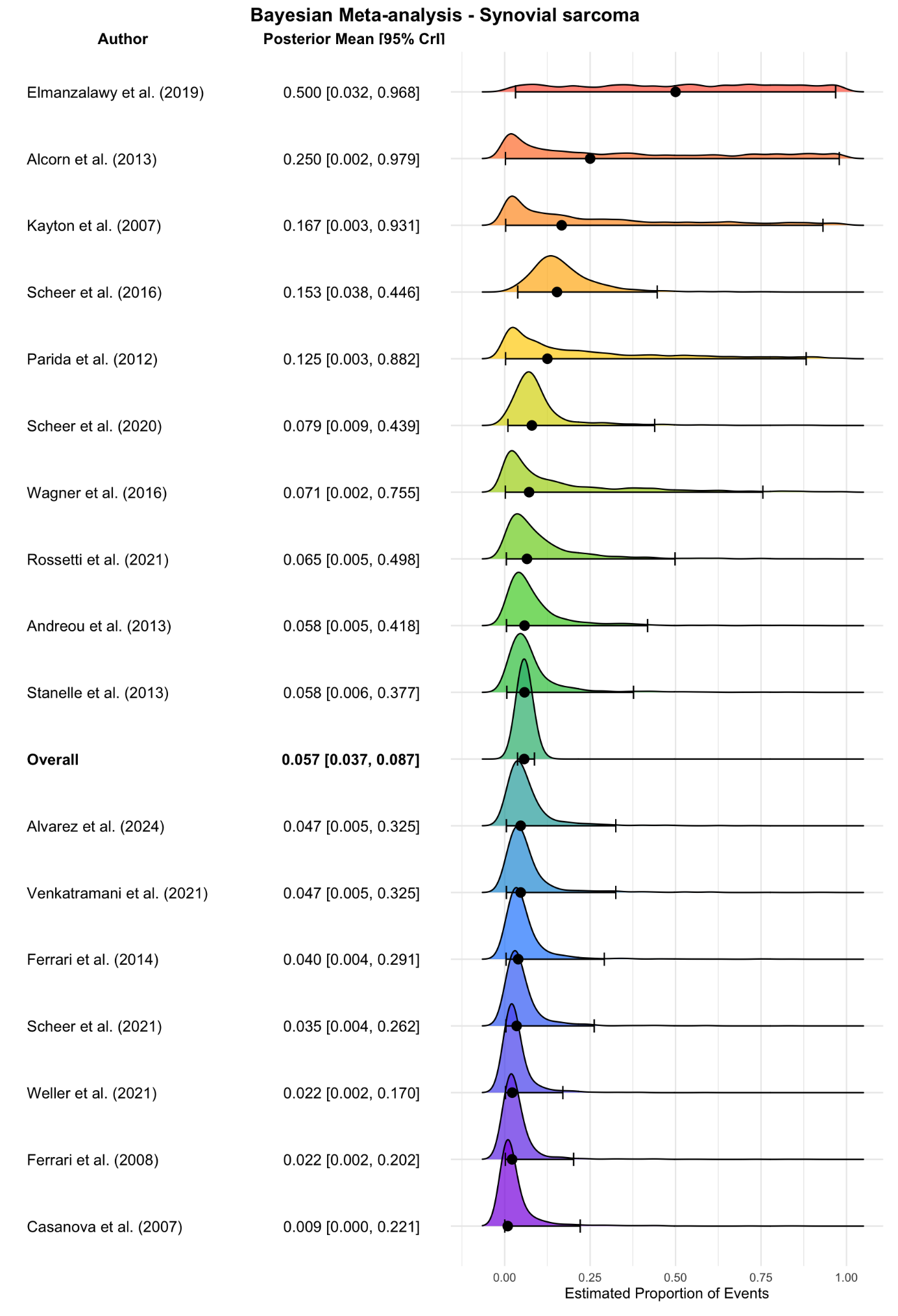

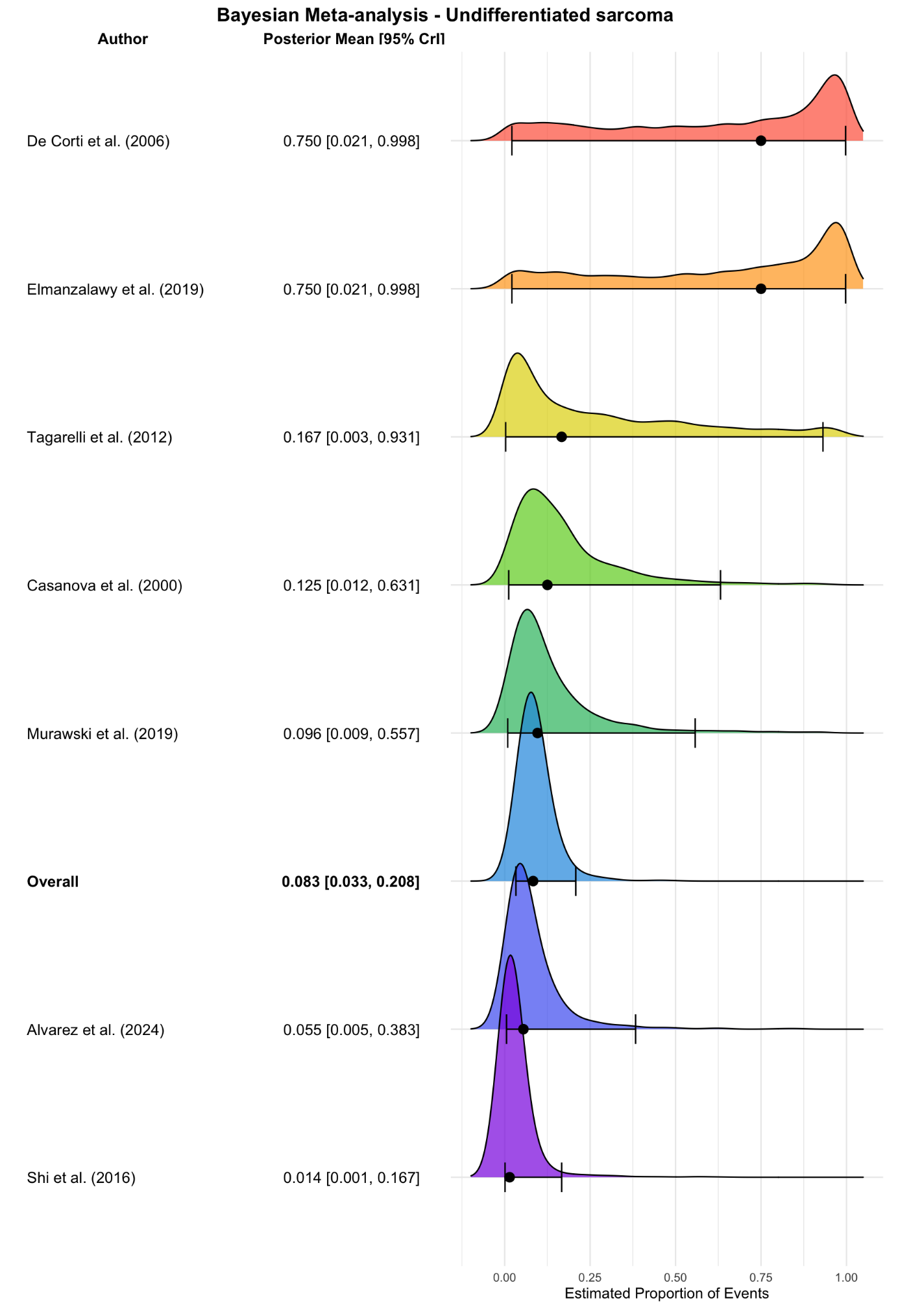

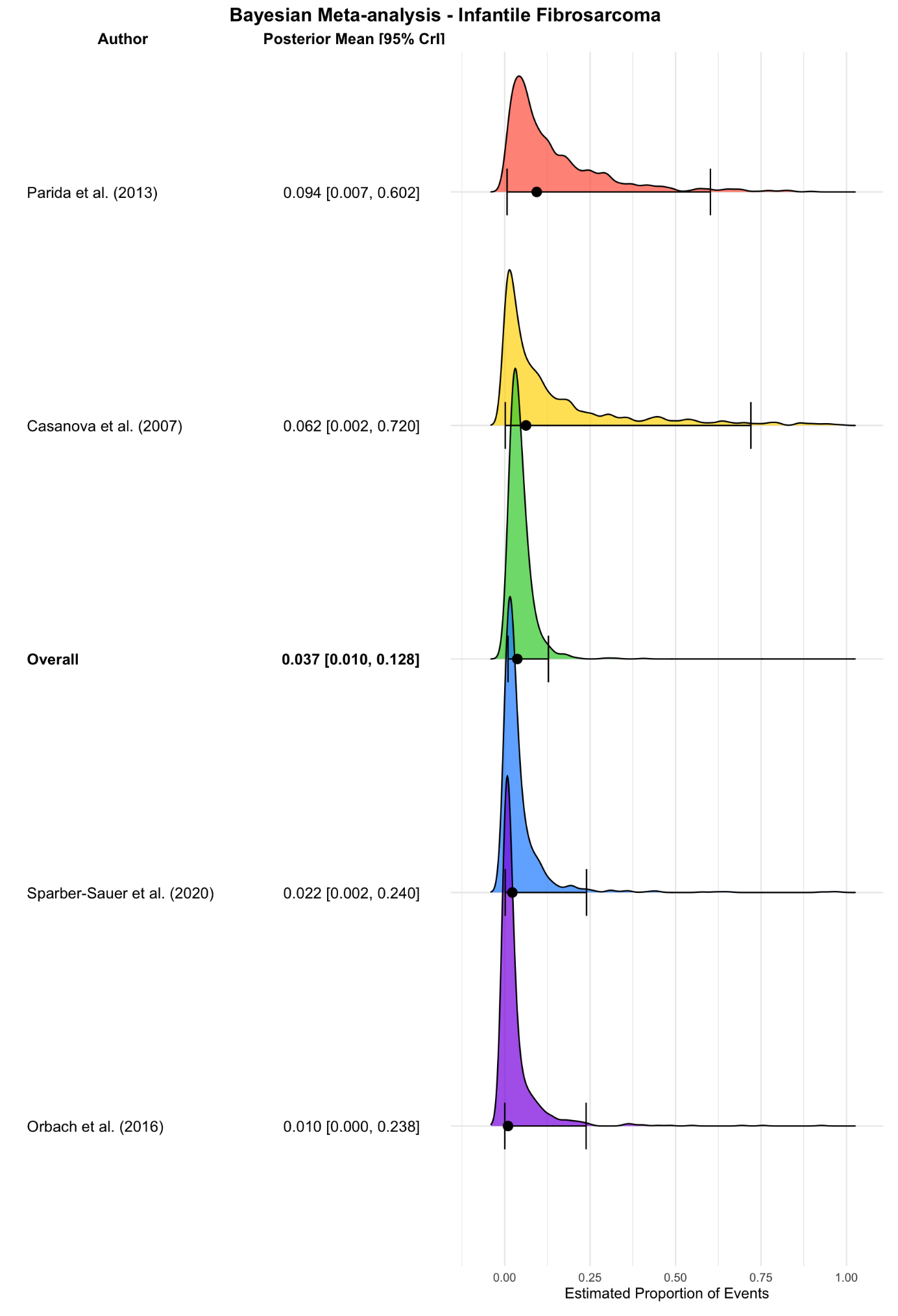

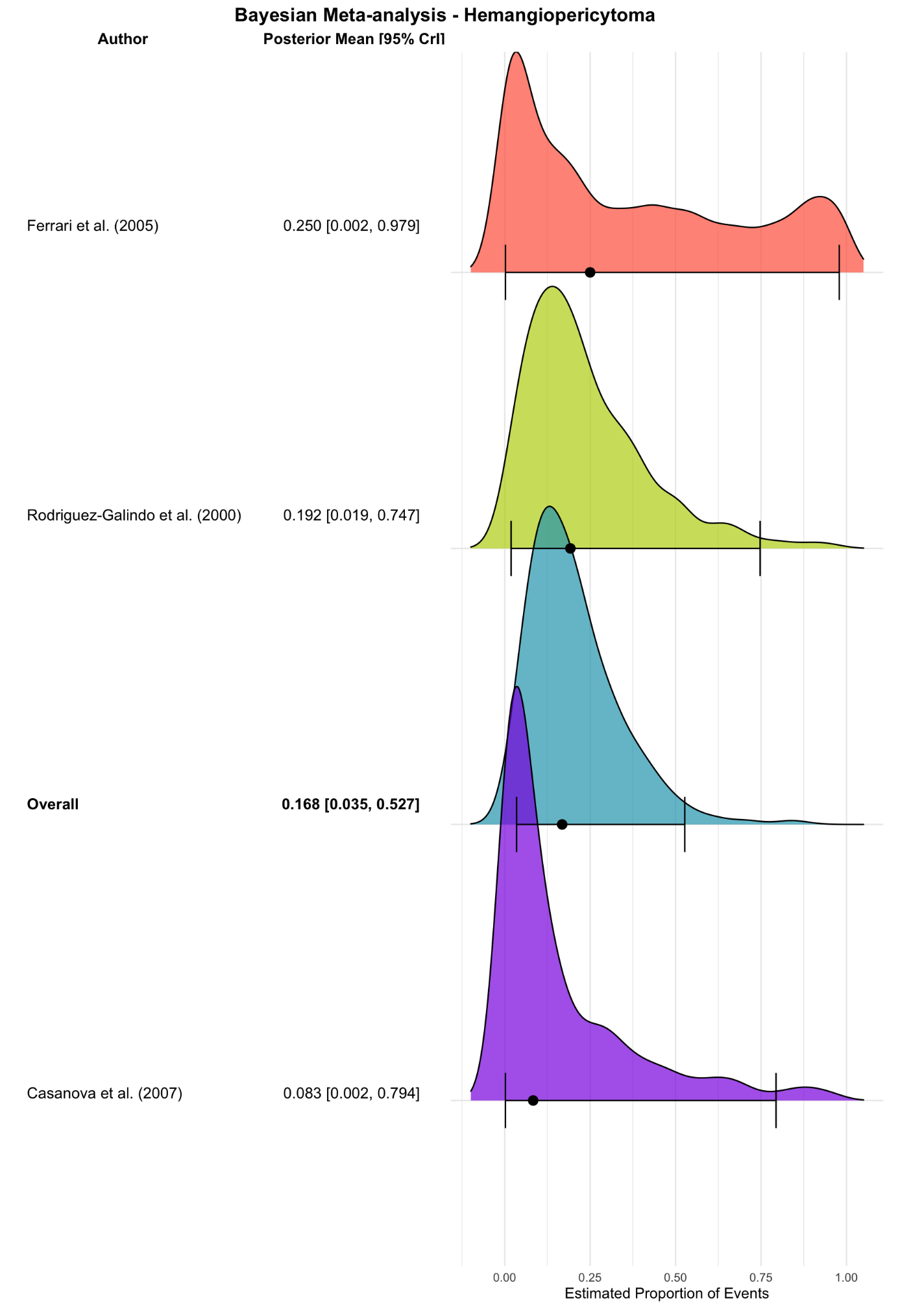

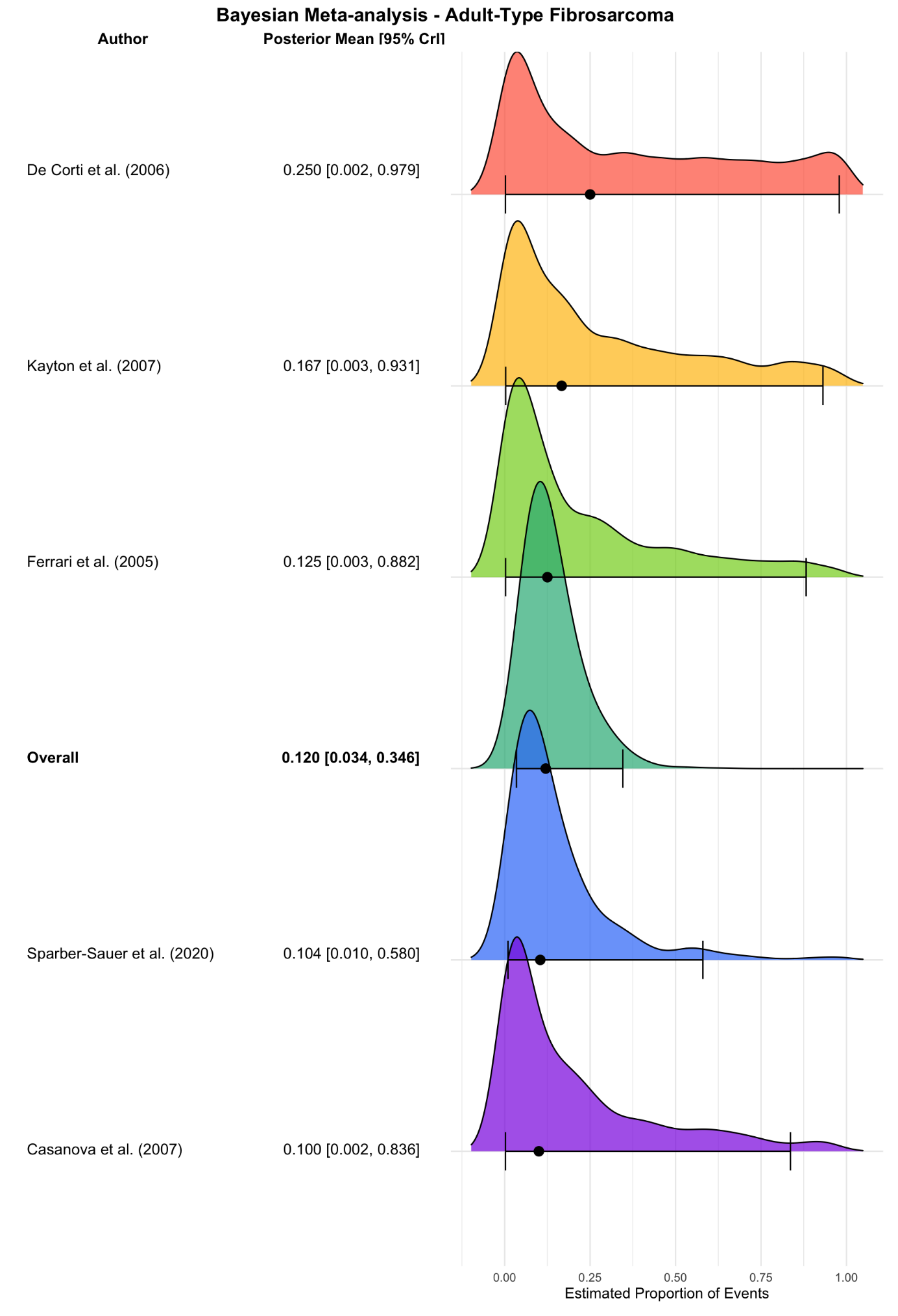

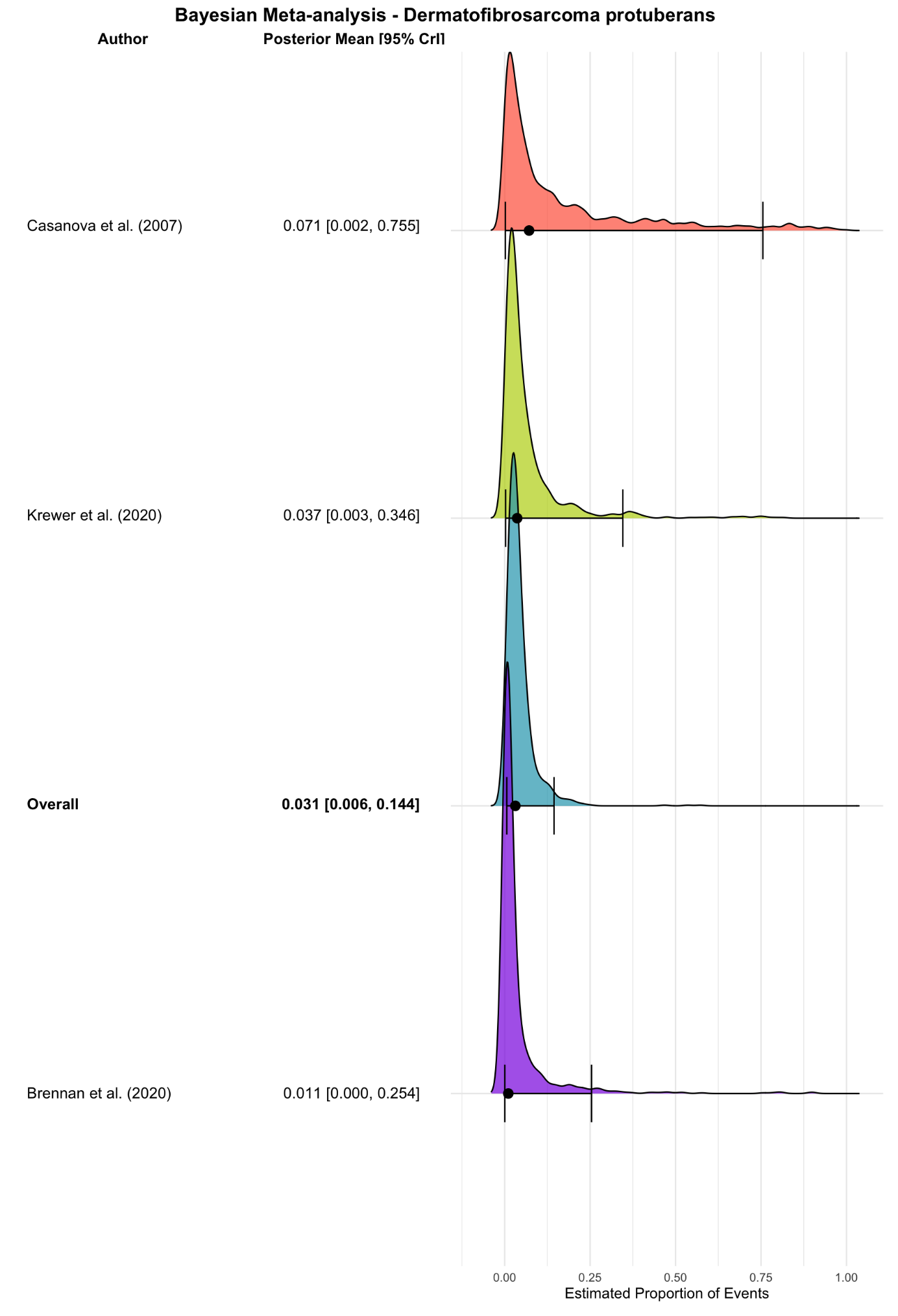

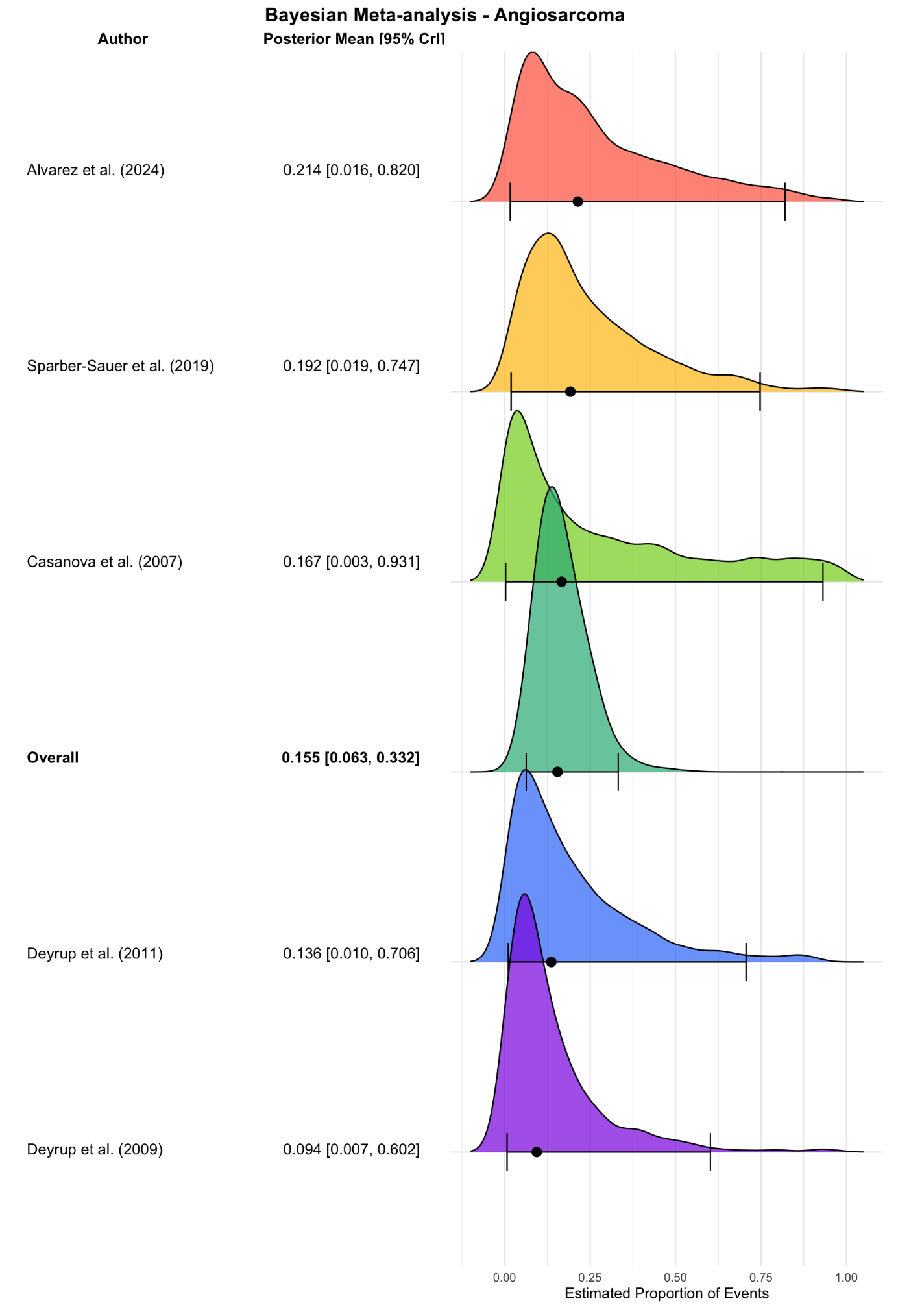

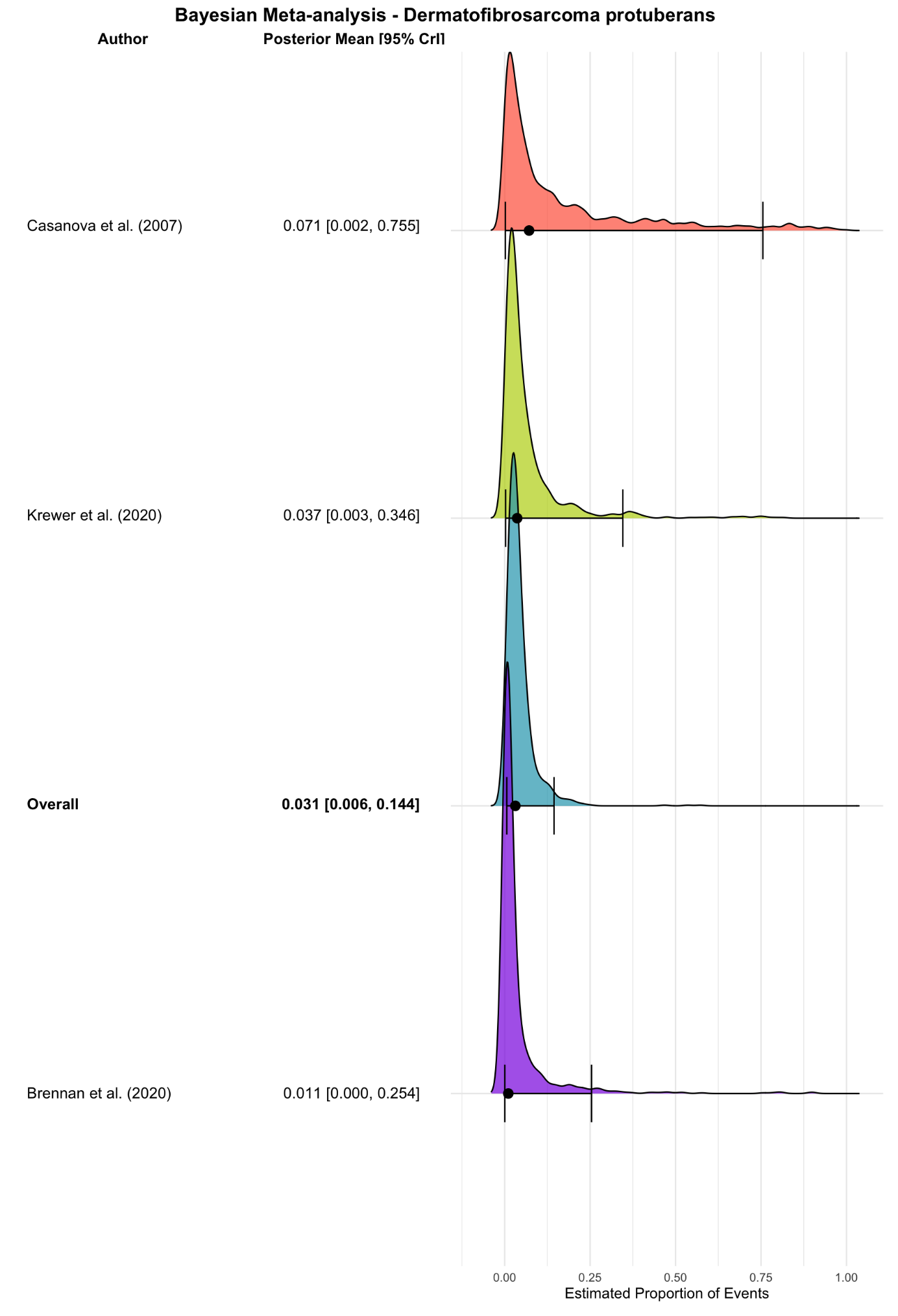

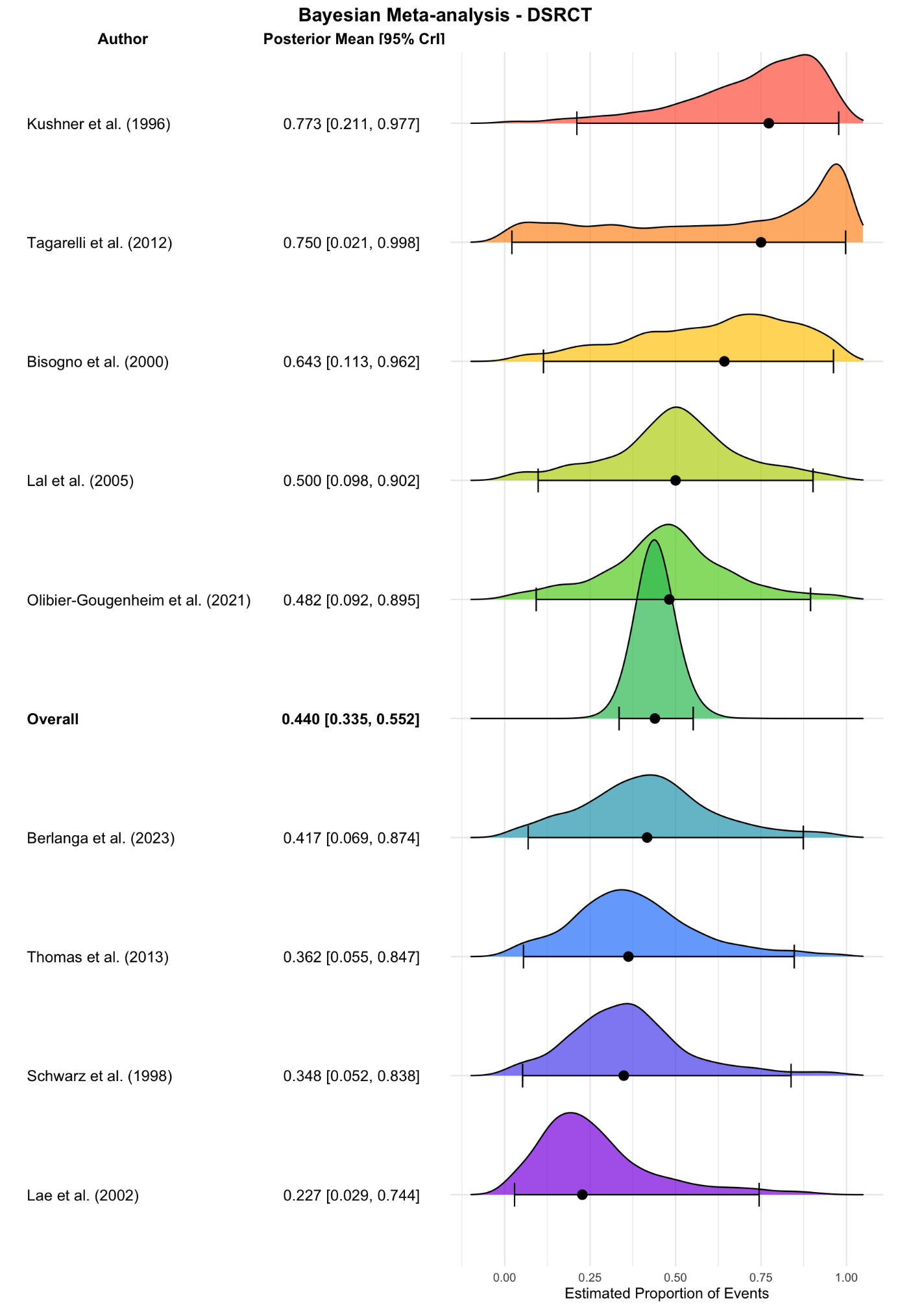

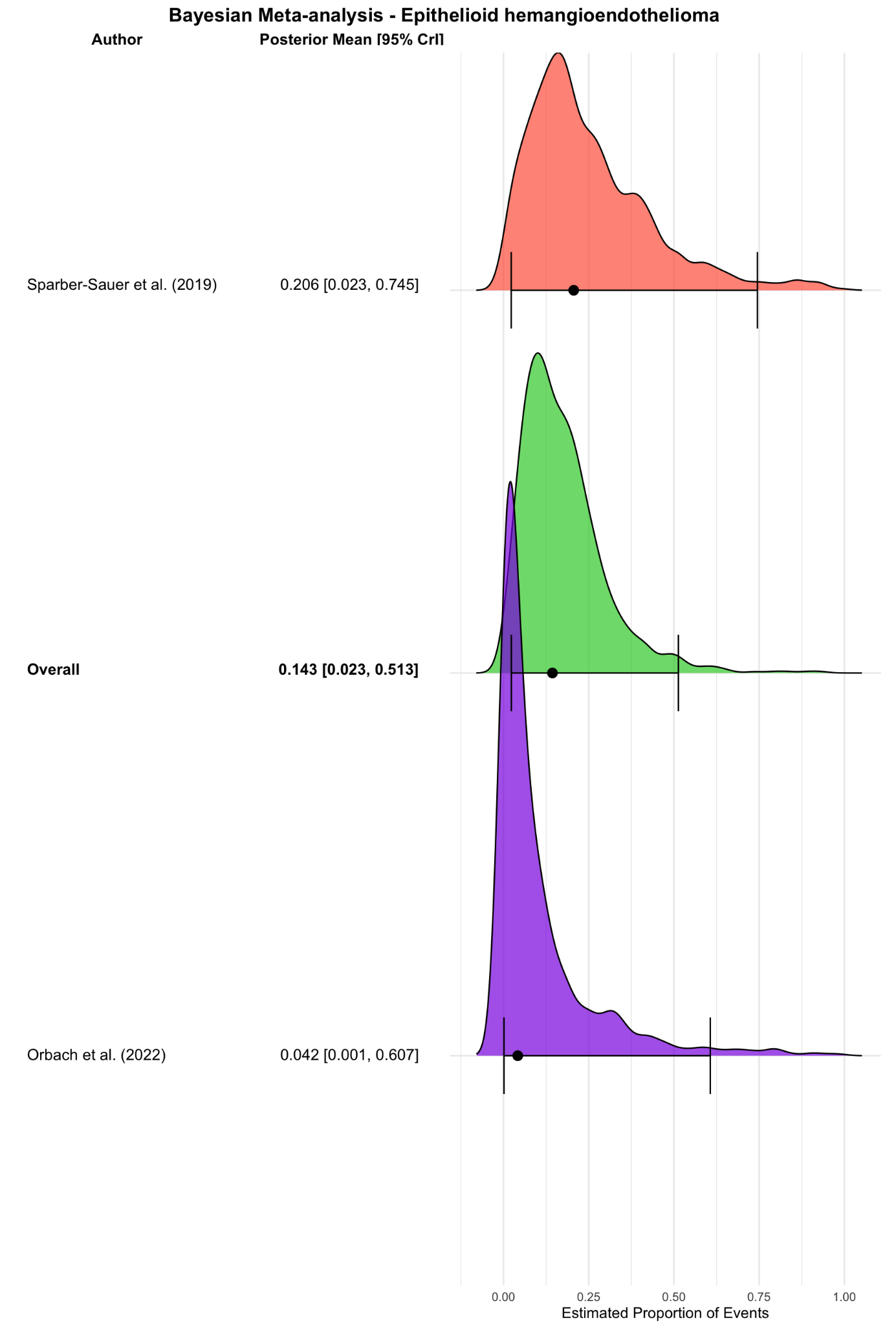

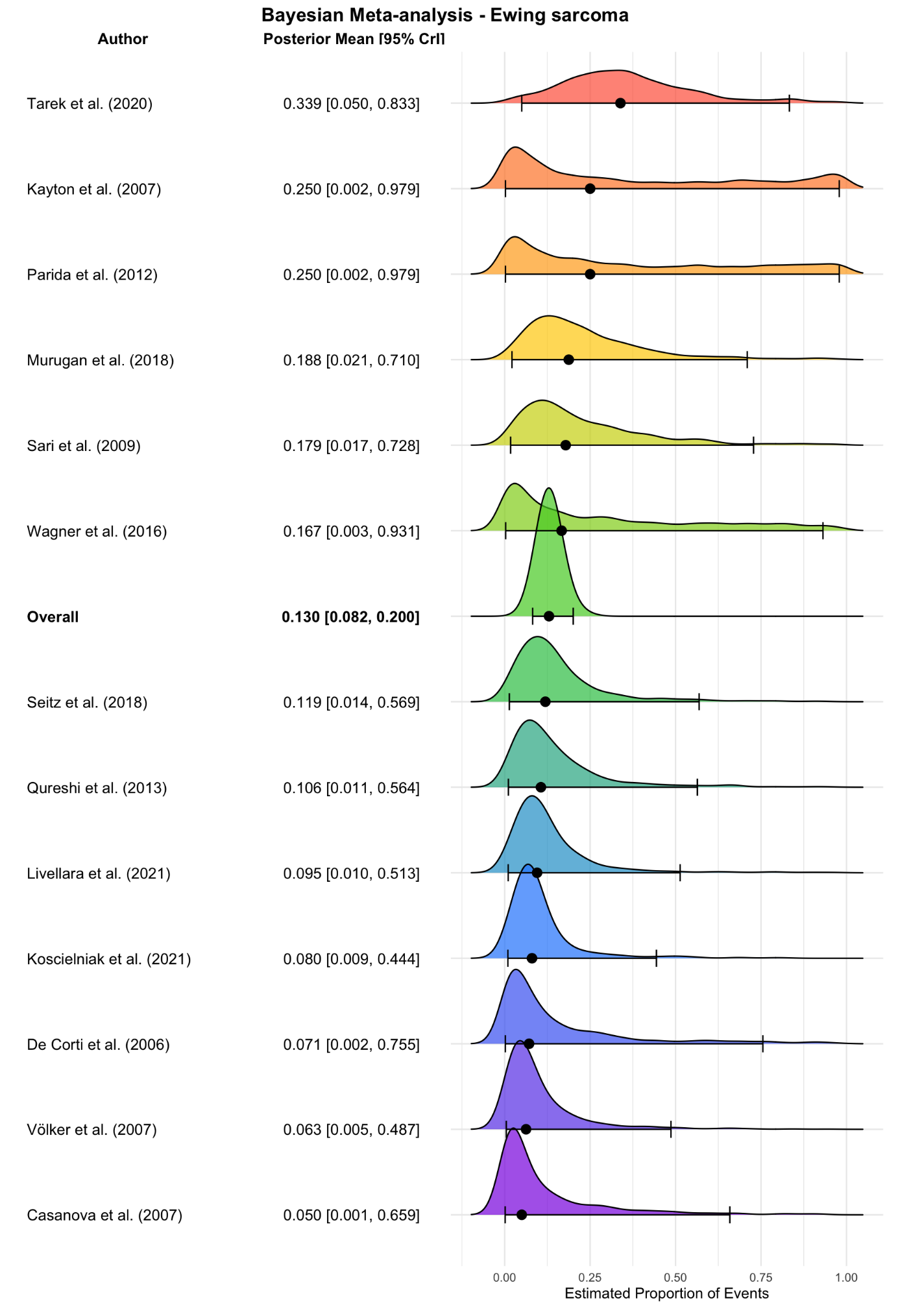

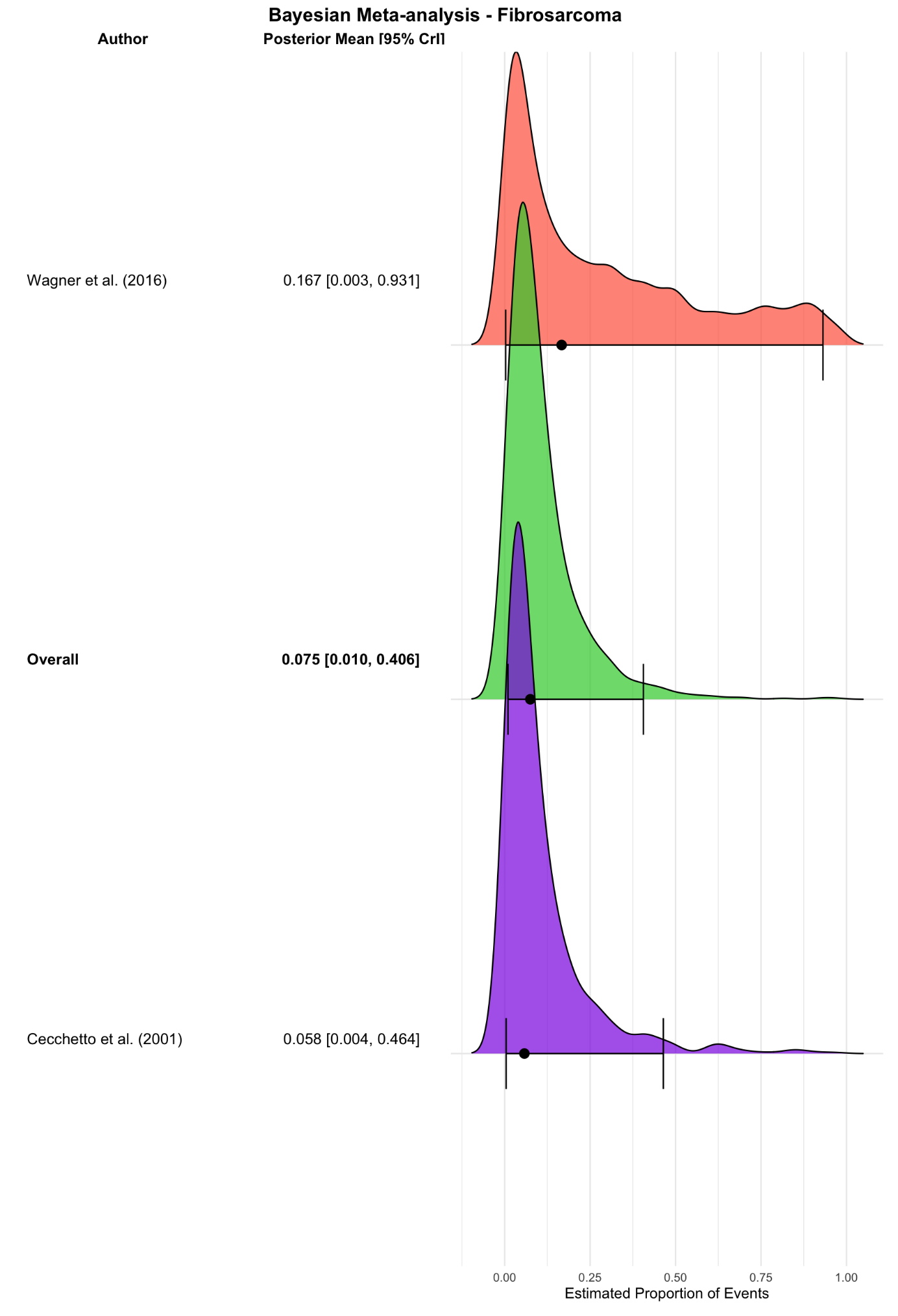

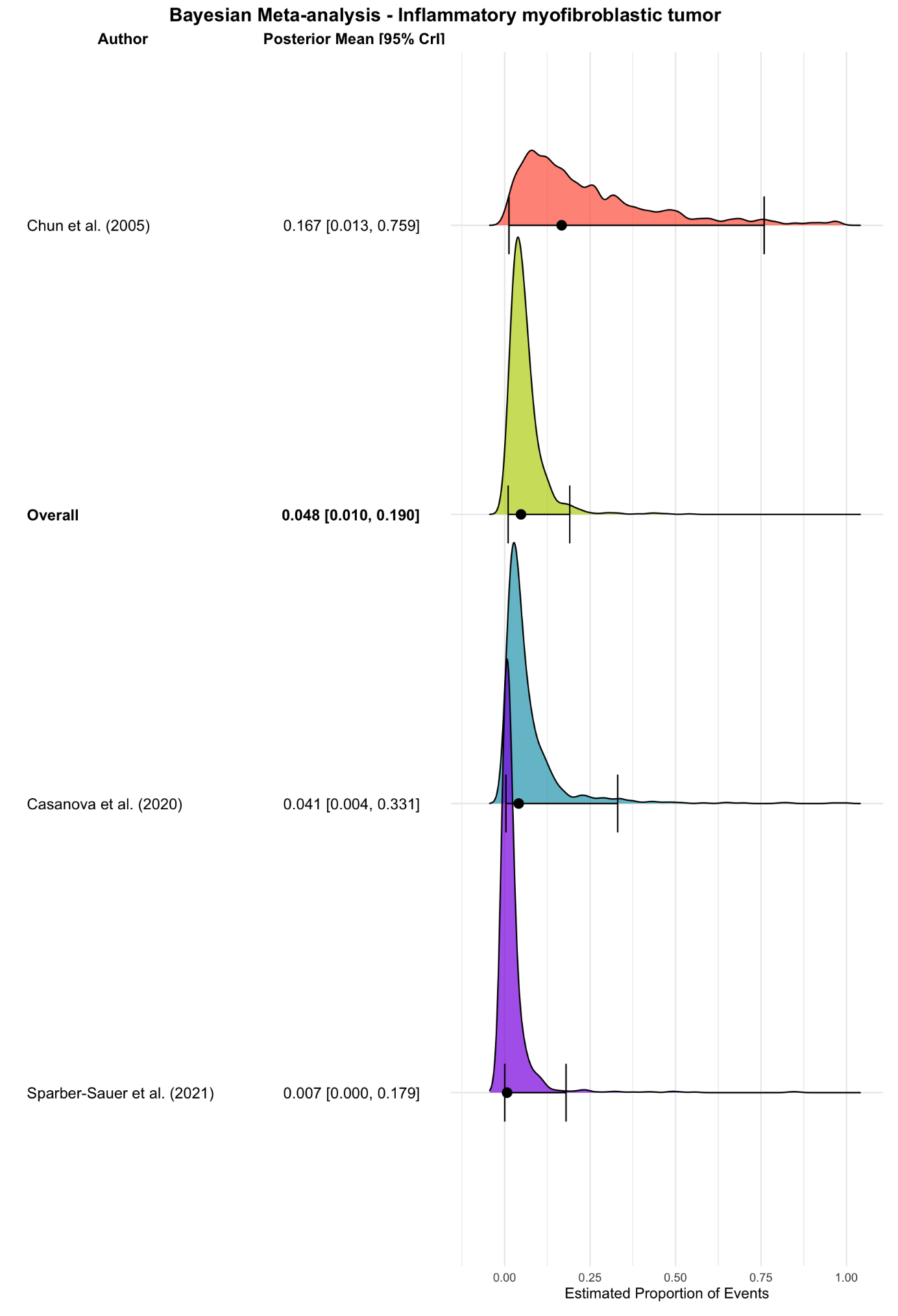

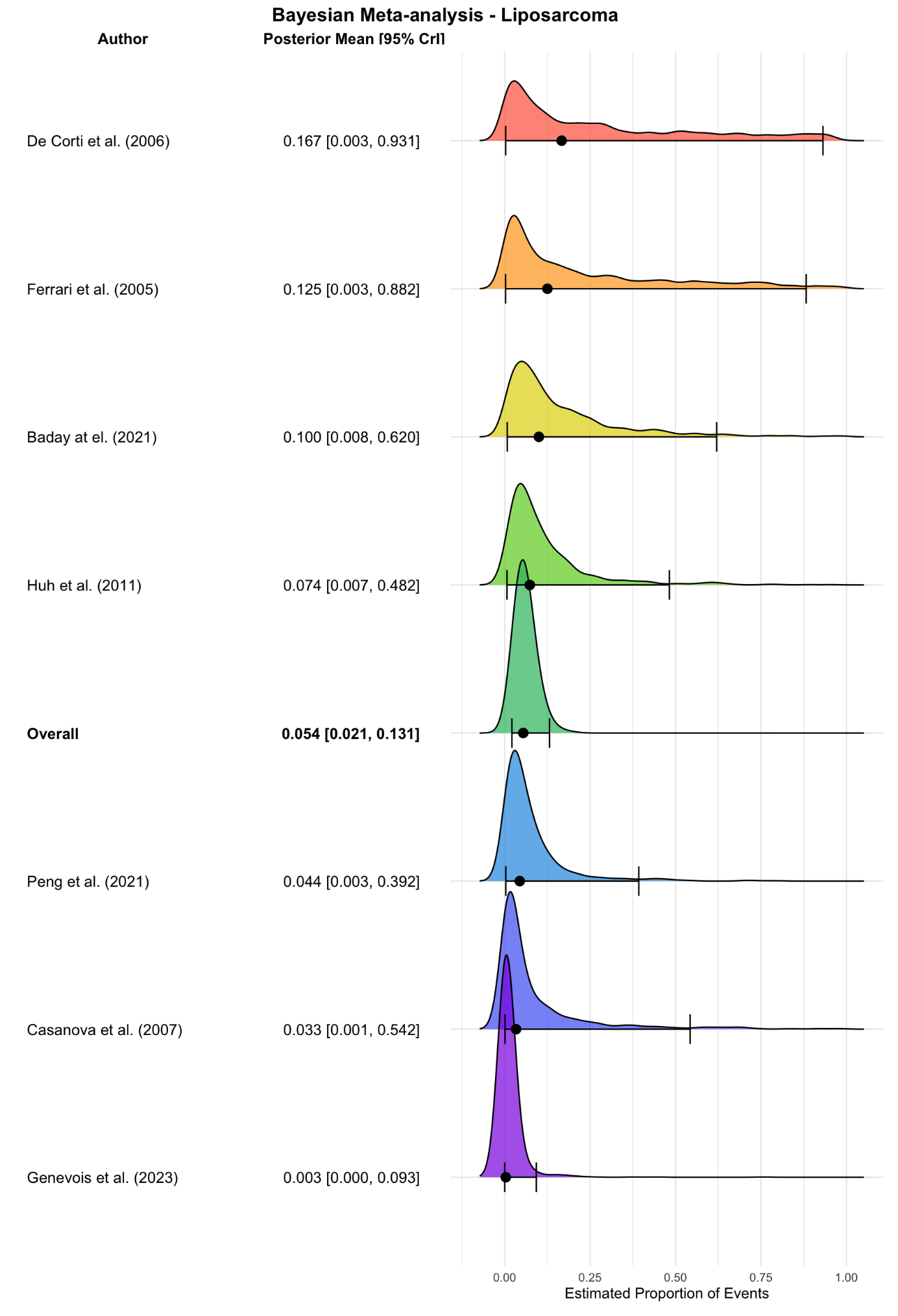

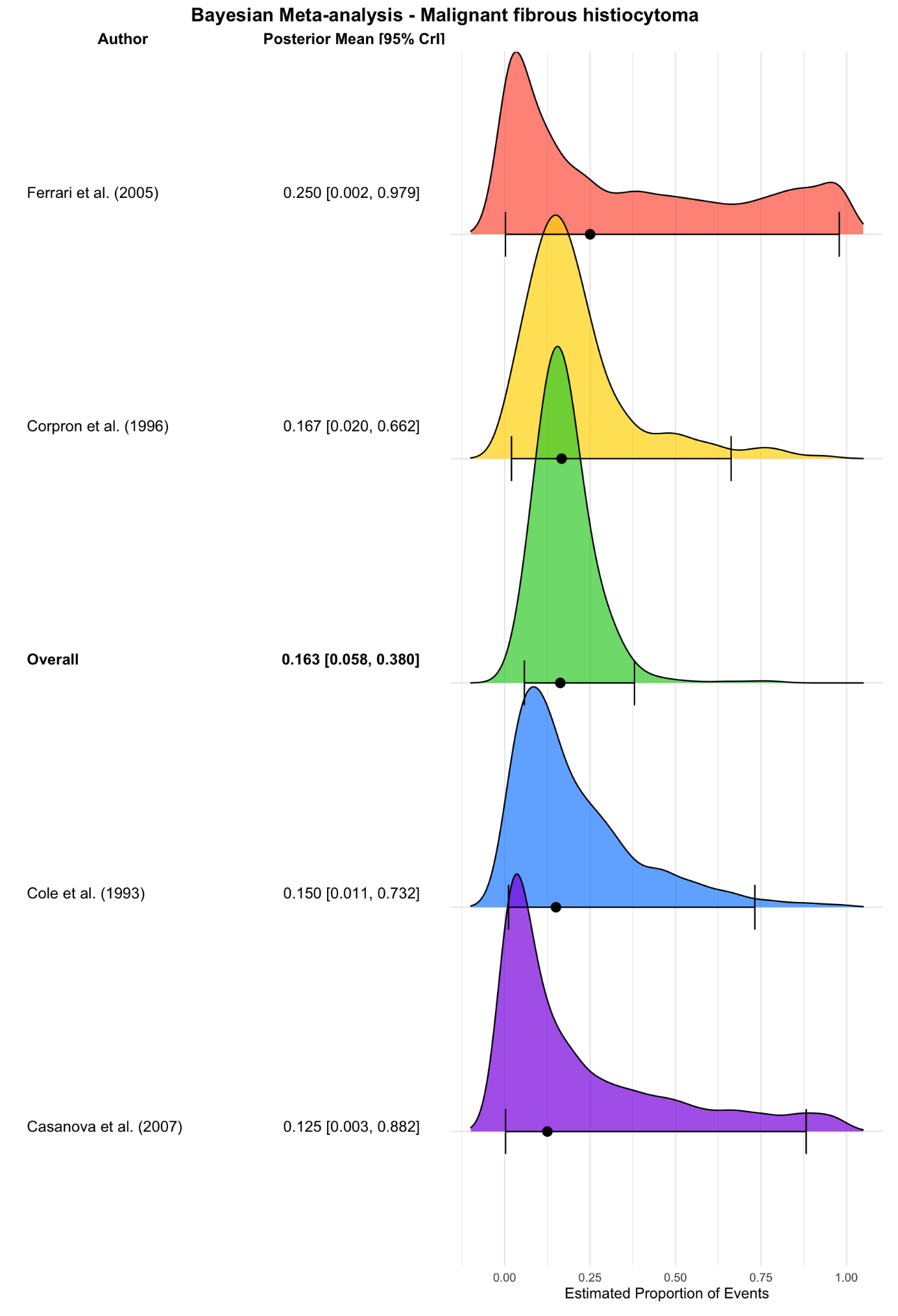

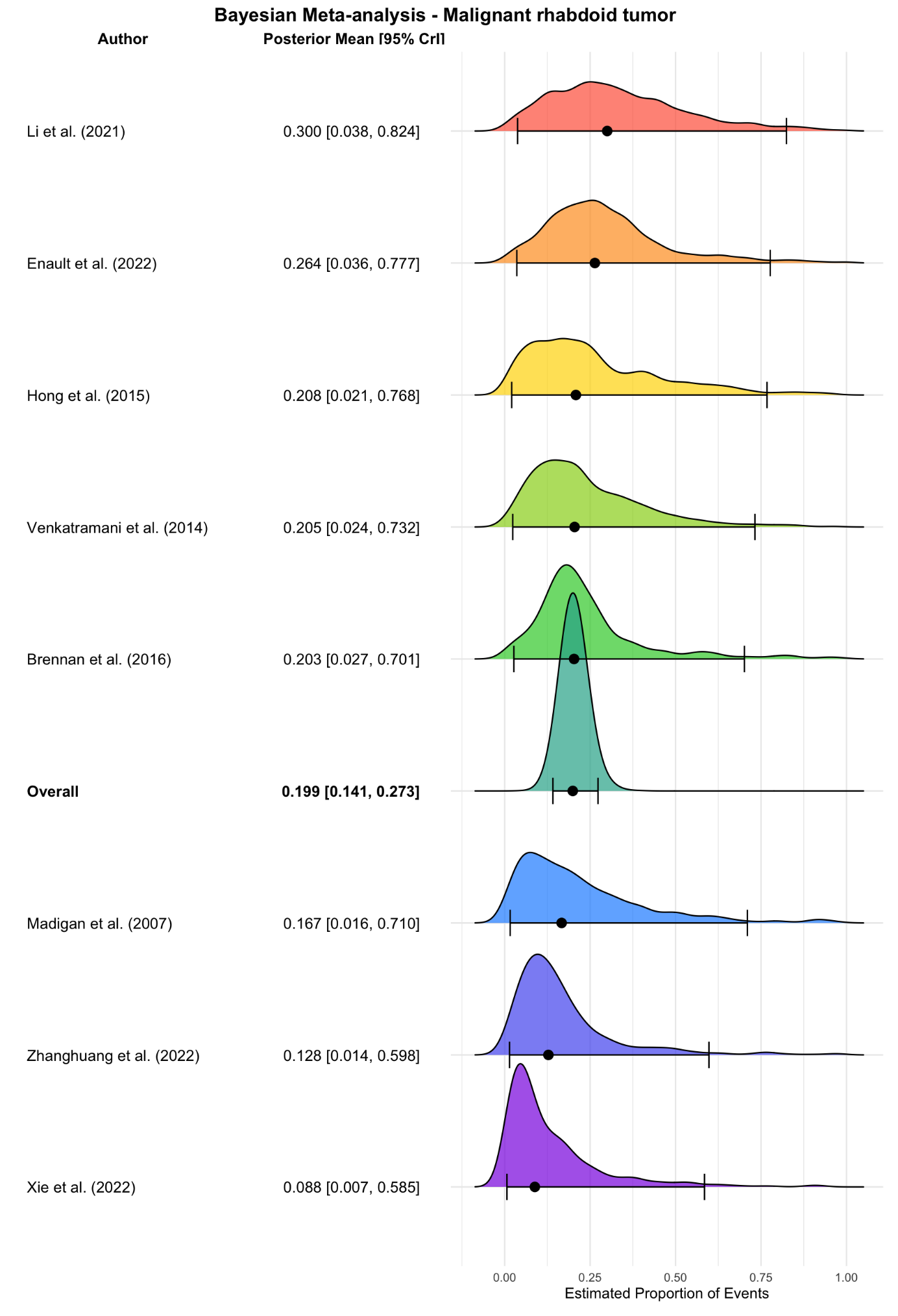

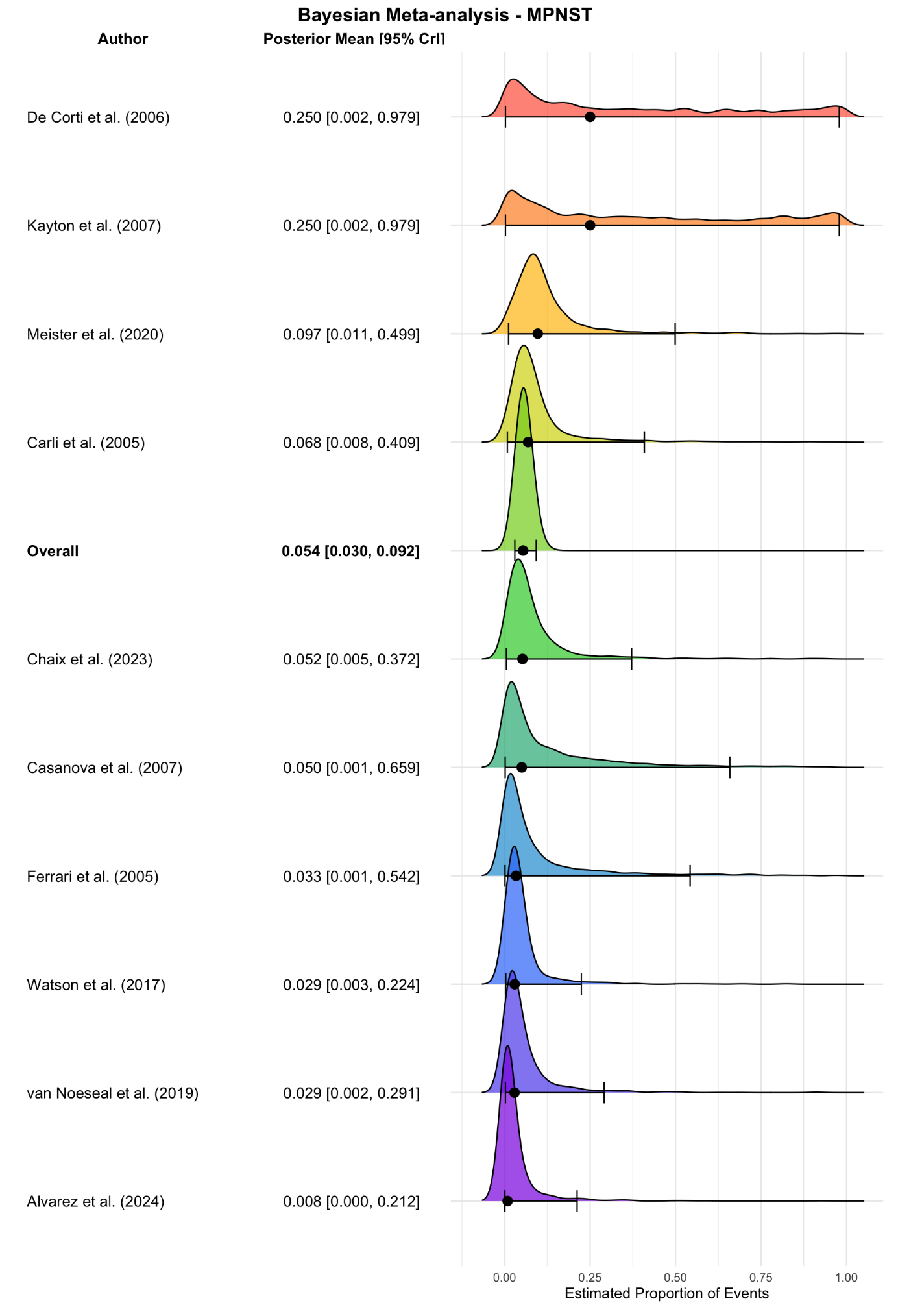

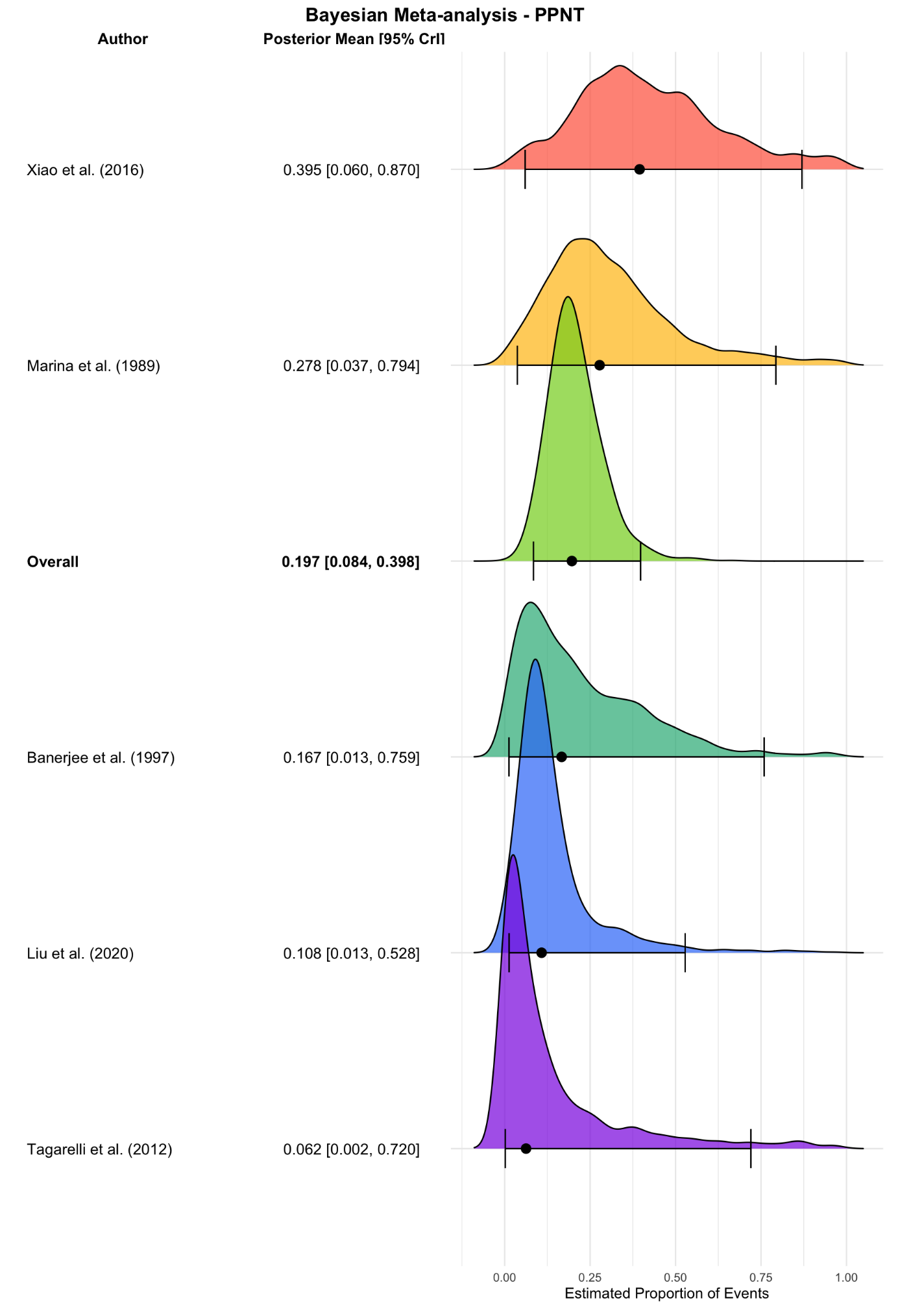

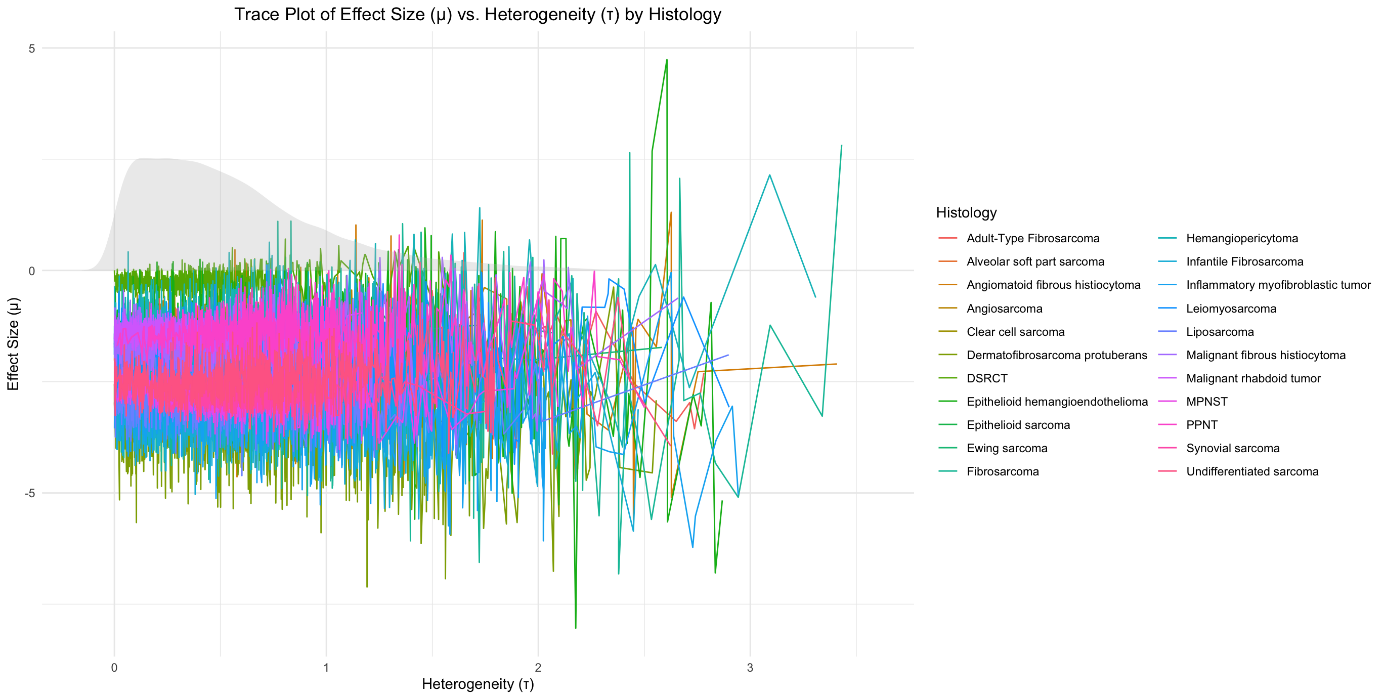

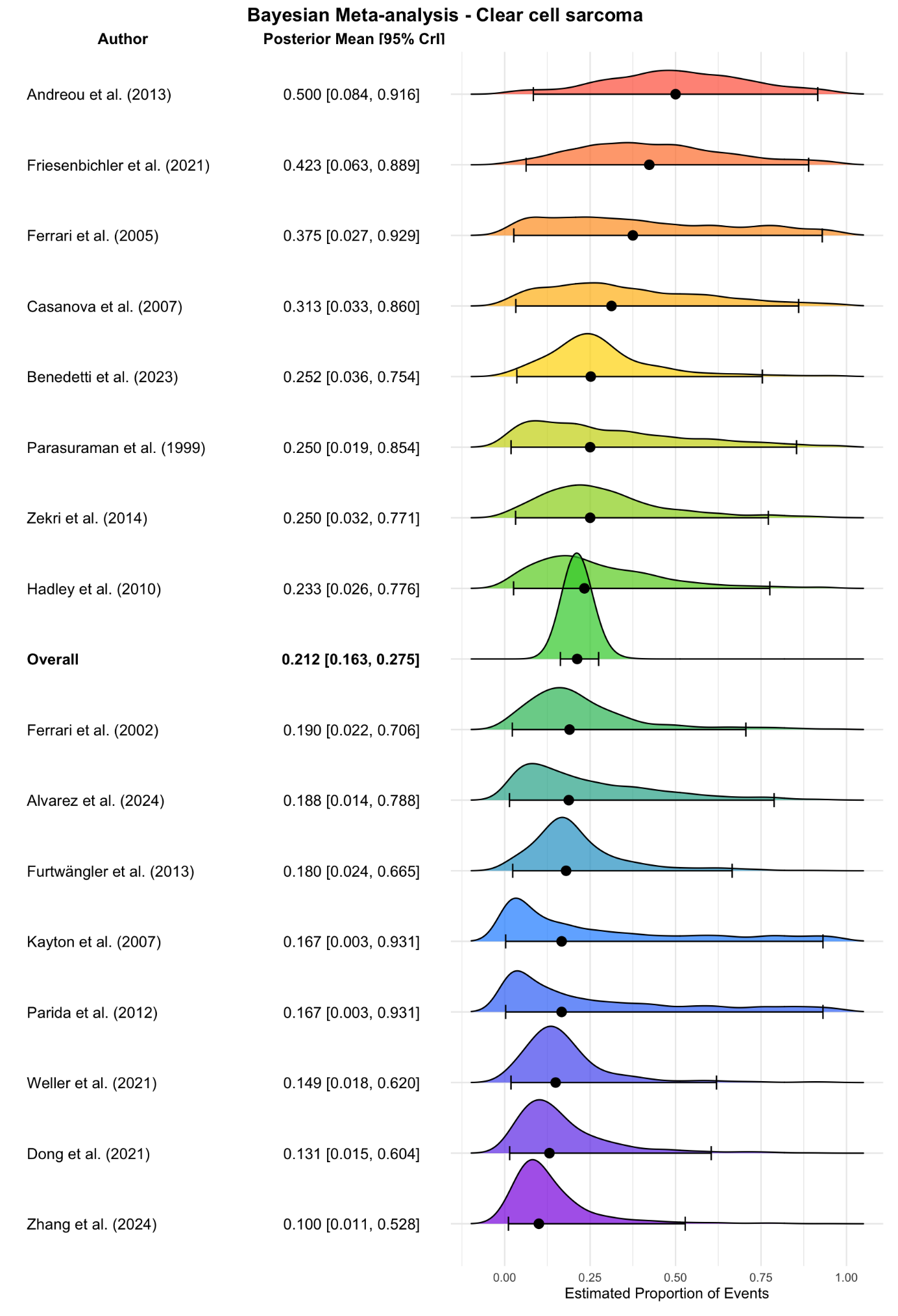

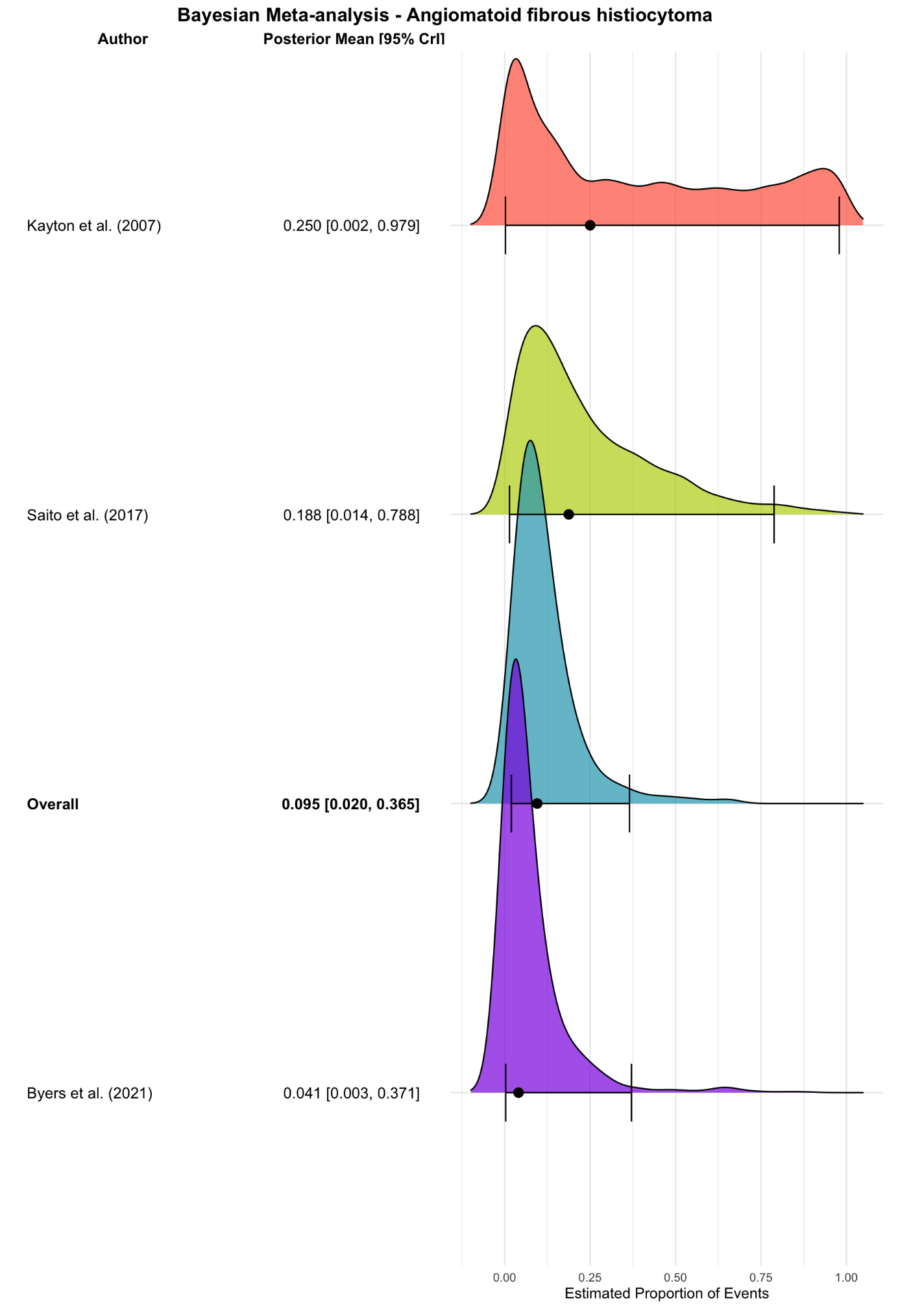

Supplement: Supplementary Fig. S11 [file mmc26.docx]

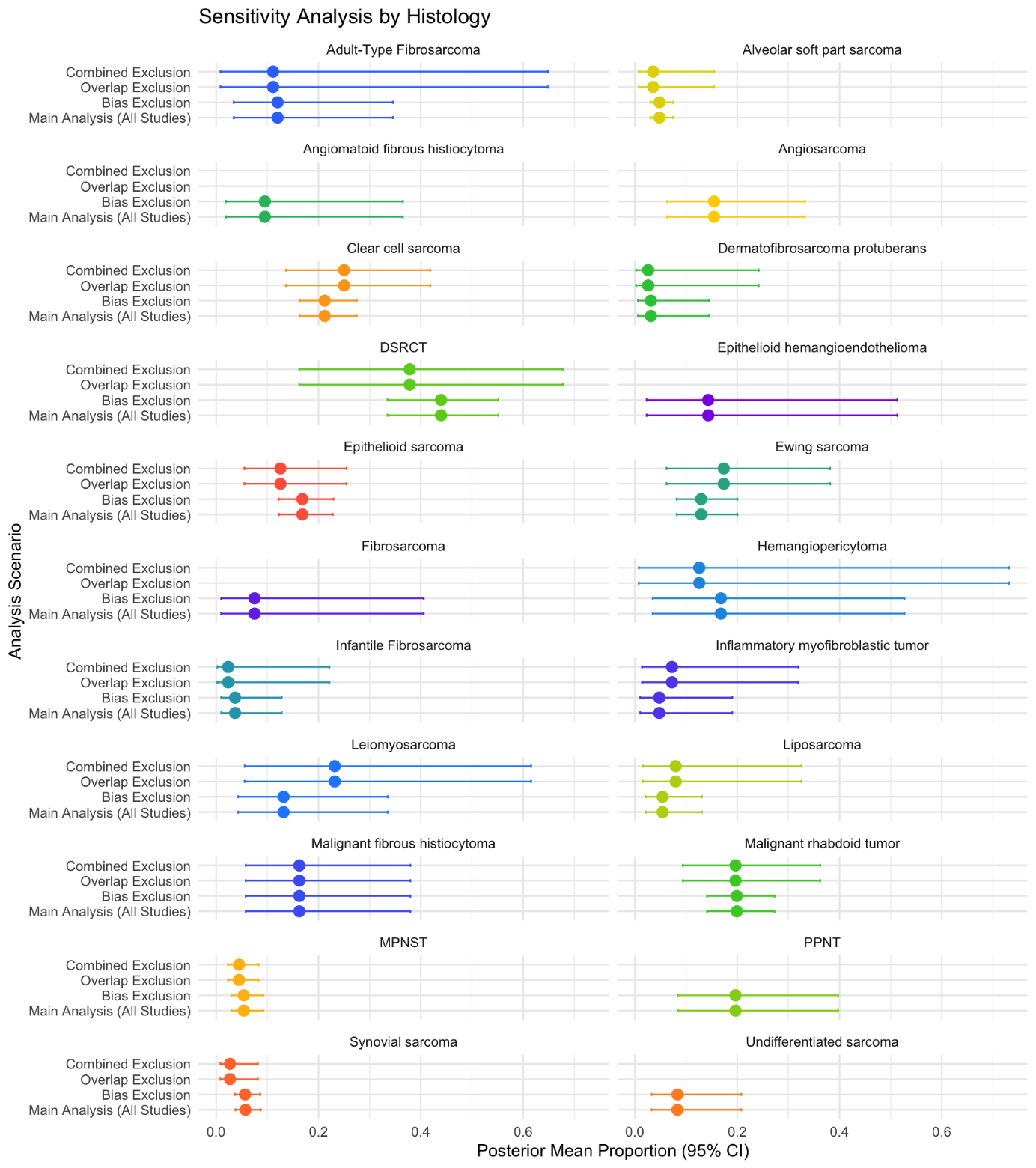

Supplement: Supplementary Fig. S12 [file mmc27.docx]
