# Supplementary material for: Prevalence of locoregional and distant lymph node metastases in children and adolescents/young adults with soft tissue sarcomas: a Bayesian meta-analysis of proportions
Source: eClinicalMedicine. 2025 Aug 7;87:103390. doi: 10.1016/j.eclinm.2025.103390 (PMC12355419; doi:10.1016/j.eclinm.2025.103390)

RMS Density Plot (overall)


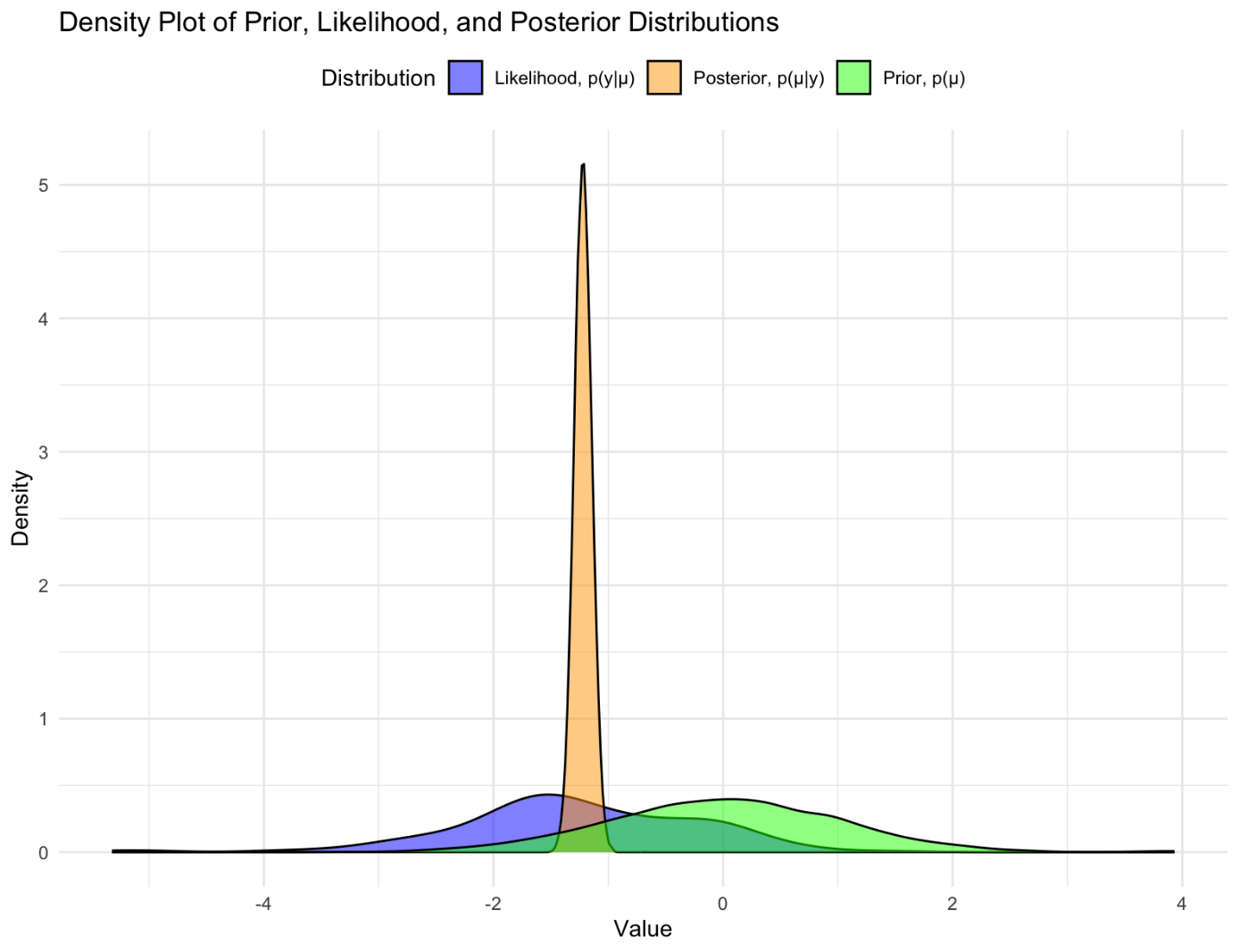


RMS Density Plot by Histology


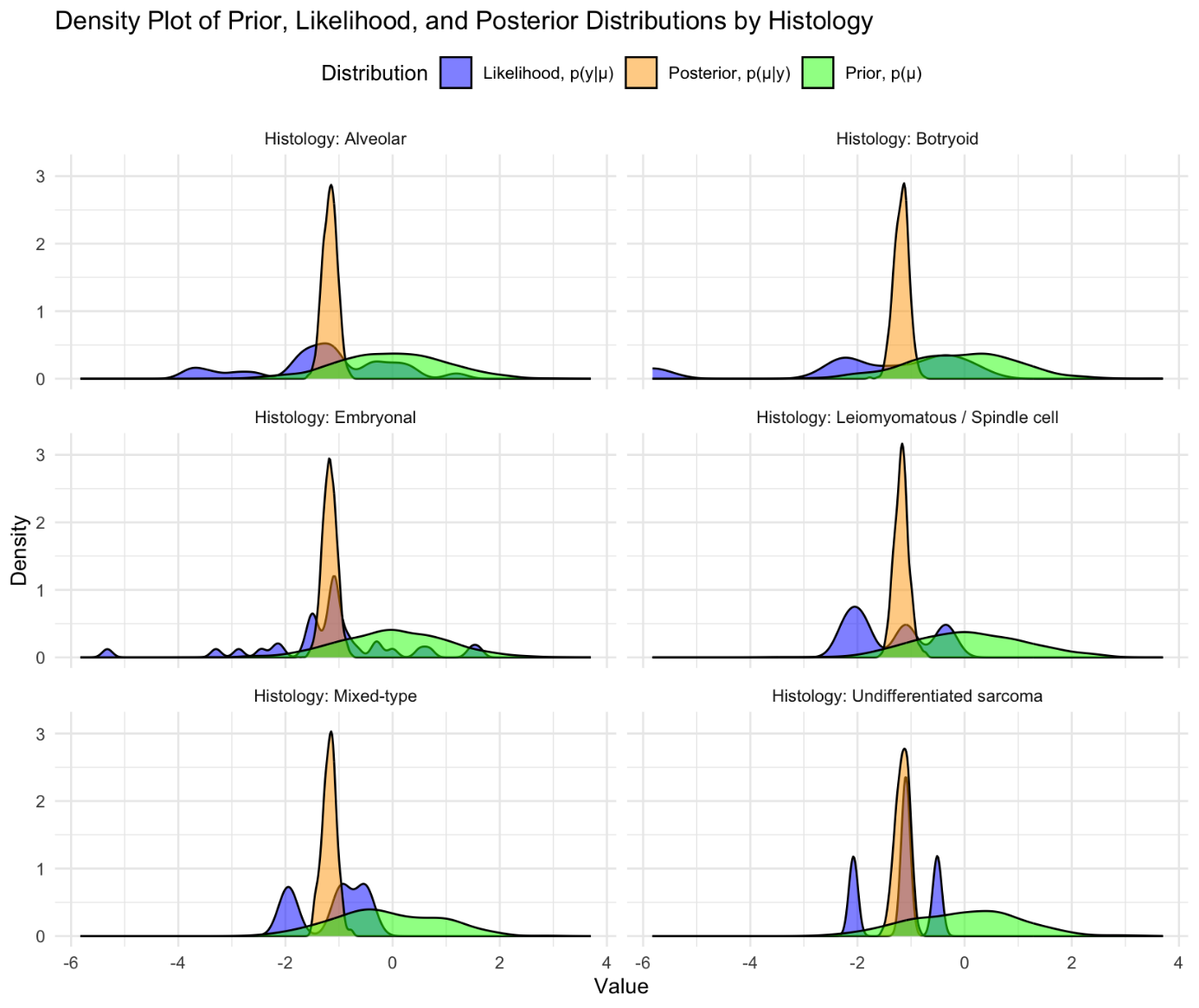


RMS Trace Plot (overall)


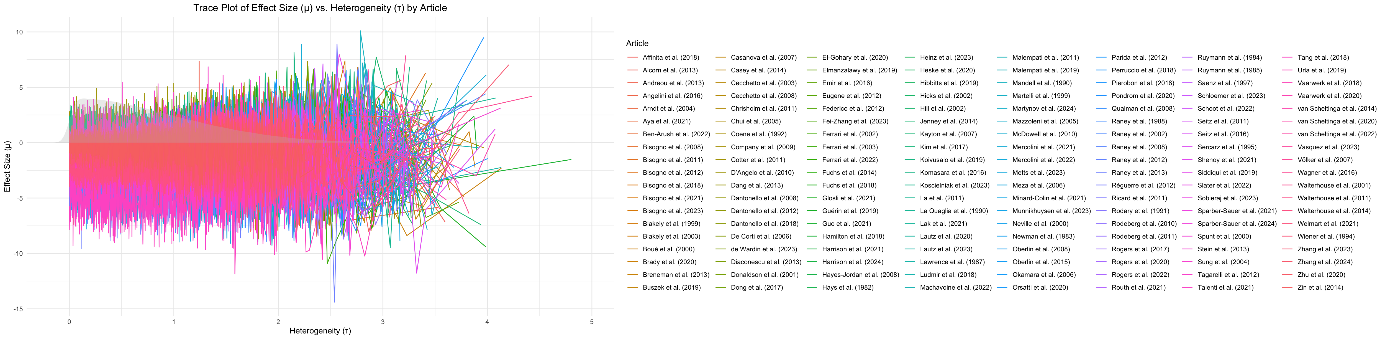


RMS Trace Plot by Histology


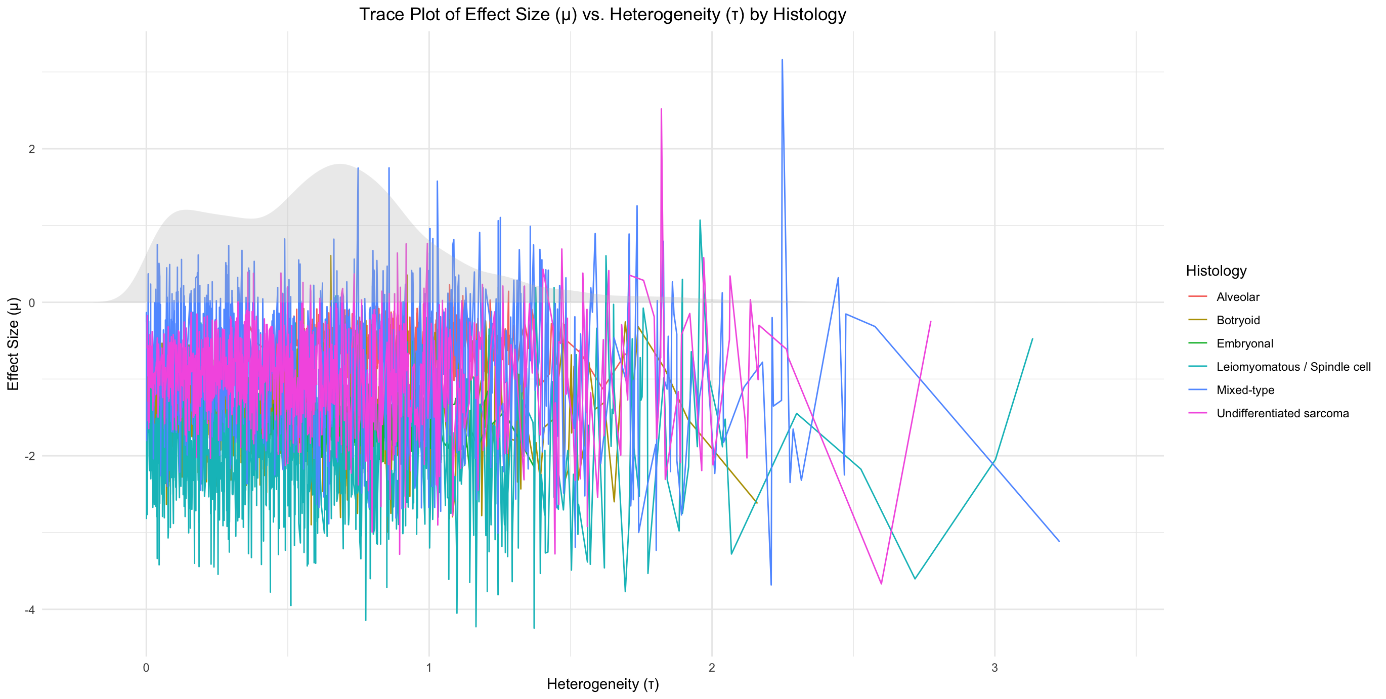


RMS Density Plot by tumor location


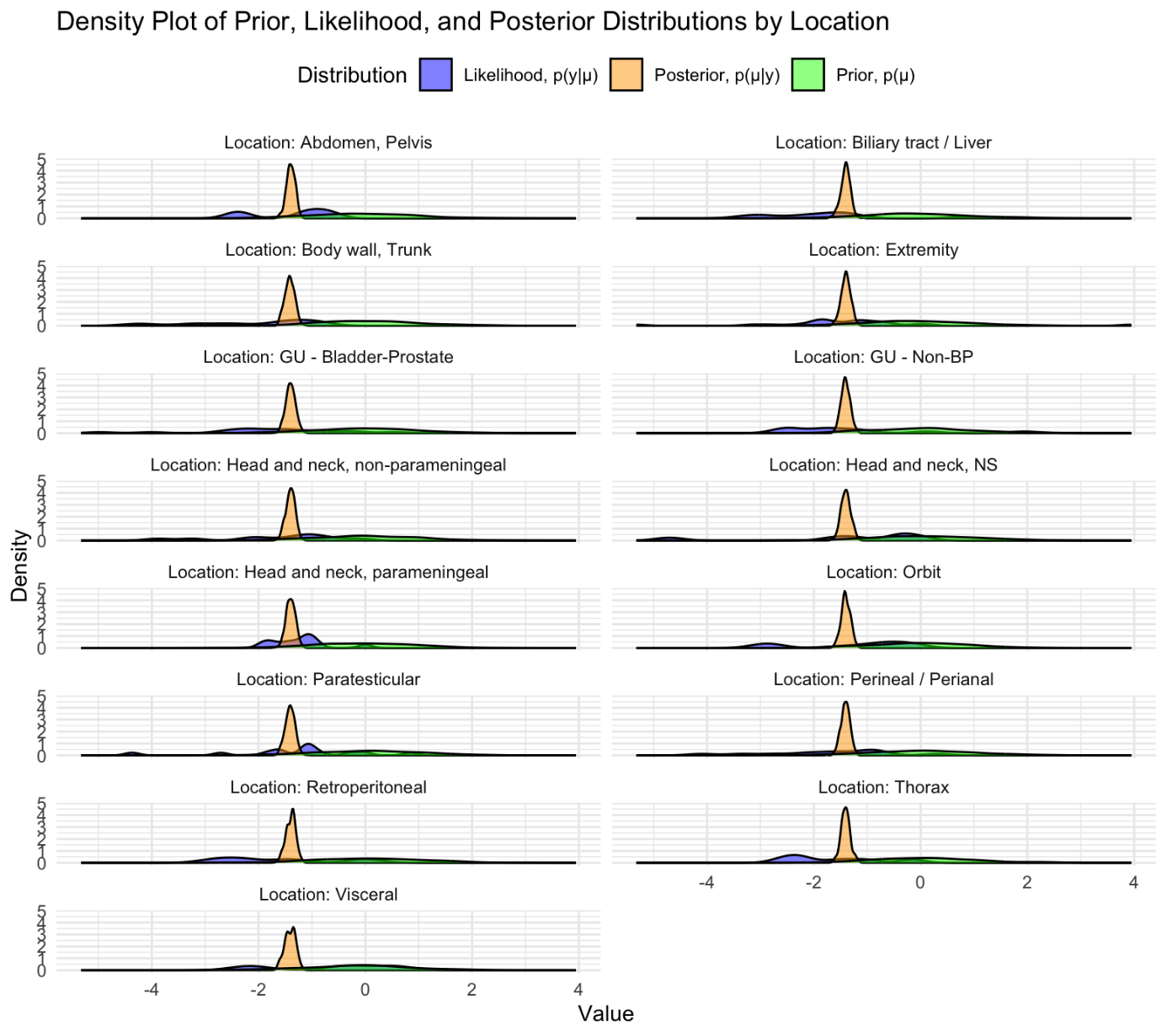


RMS Trace Plot by tumor location


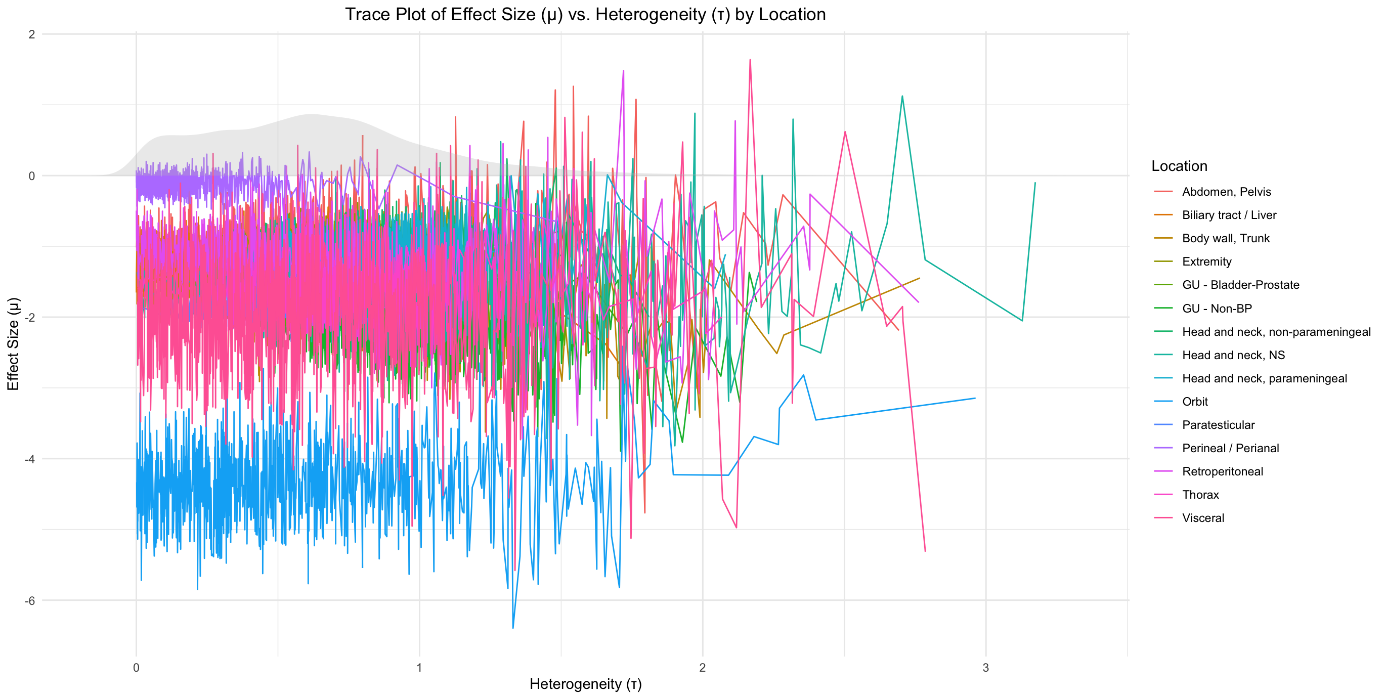


DNRSTS Density Plot overall


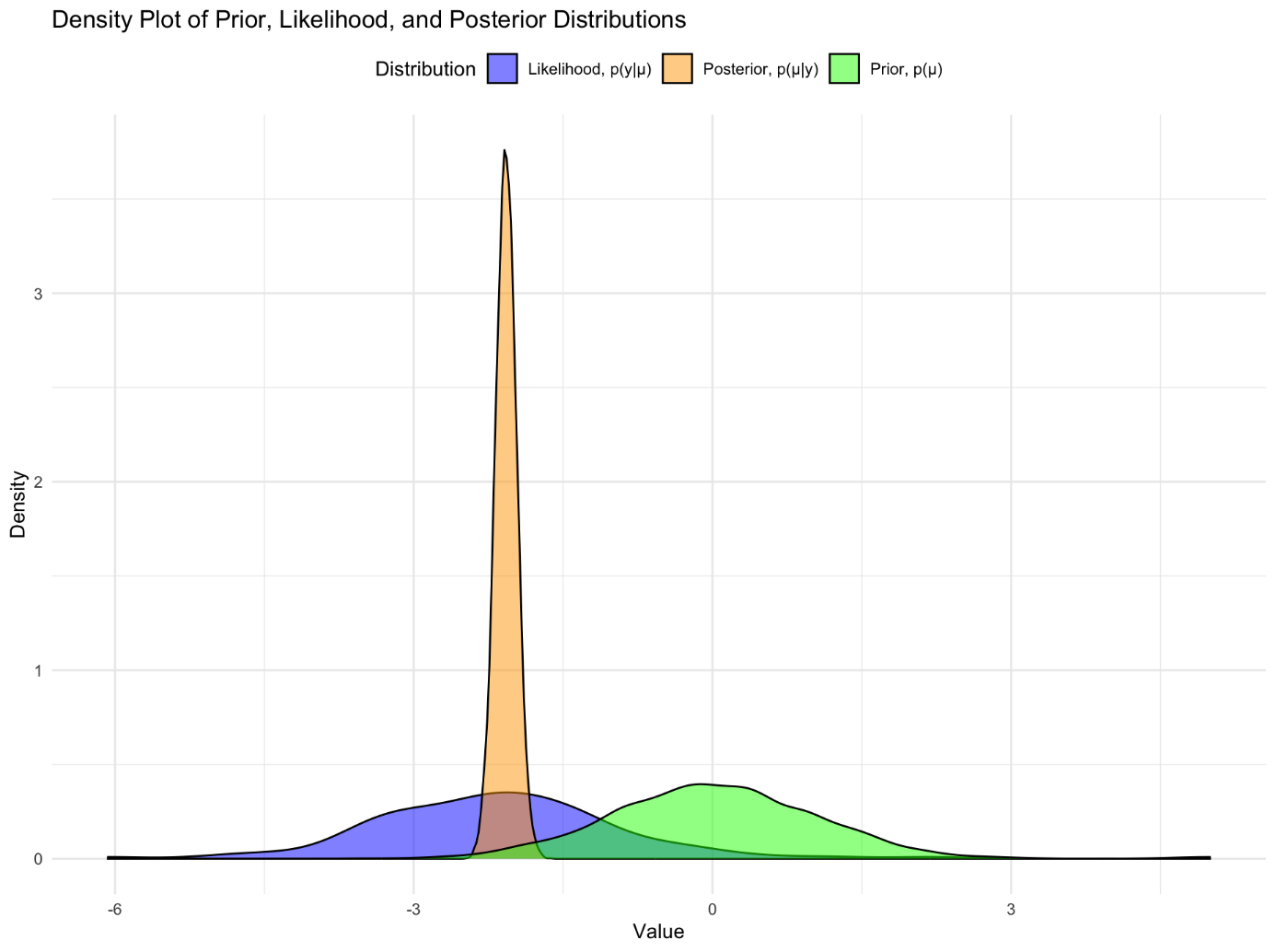


NRSTS Density Plot by Histology


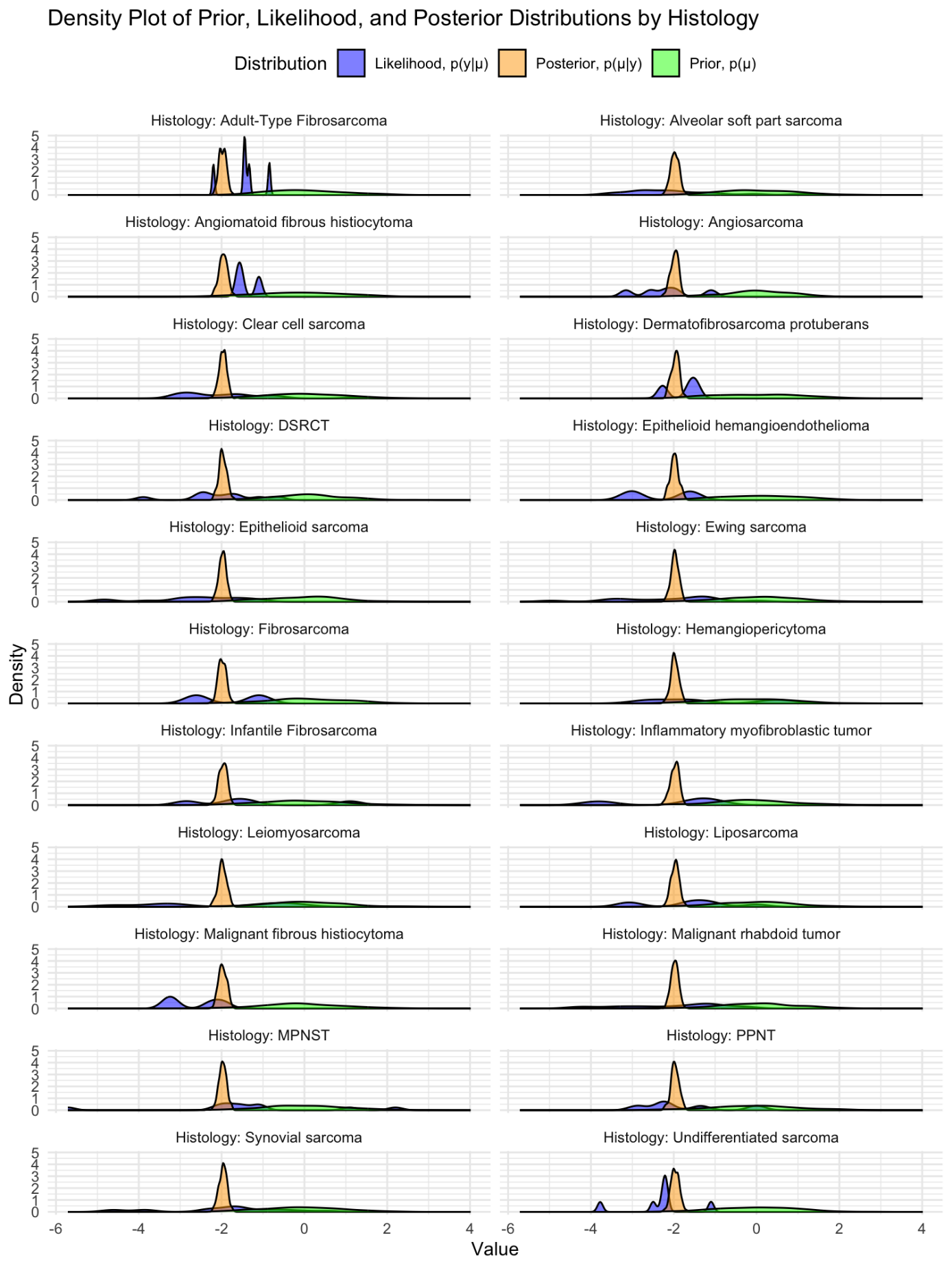


NRSTS Density Plot by Location


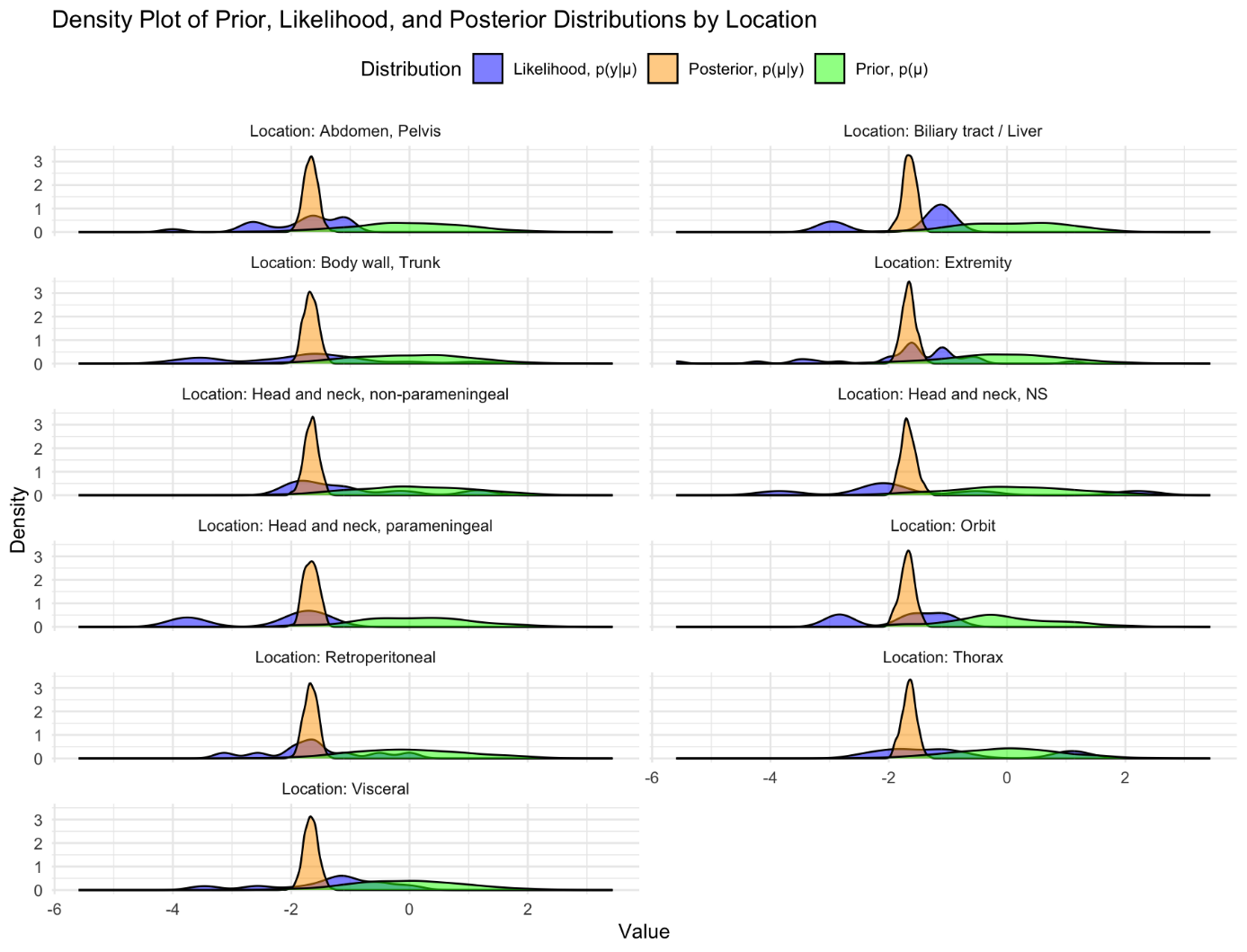


NRSTS Trace Plot (overall)


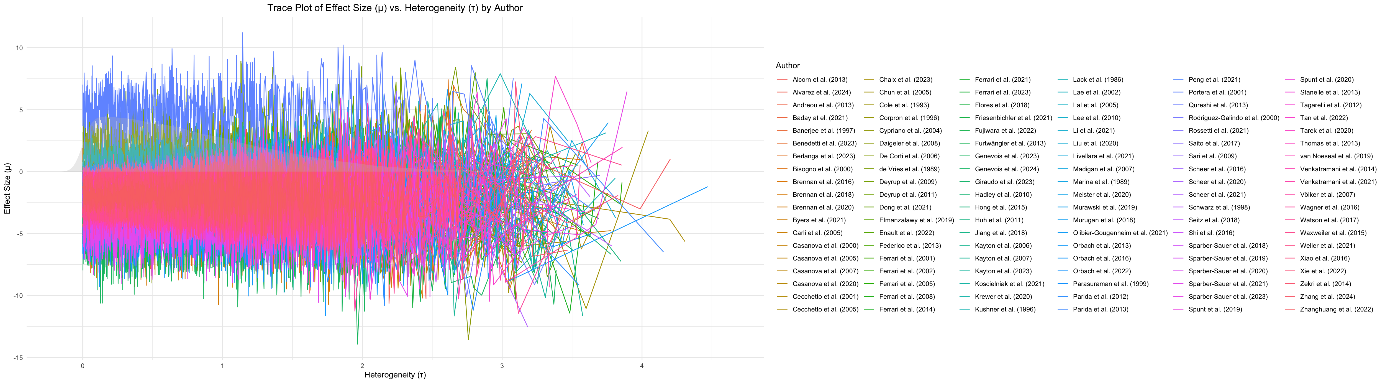


NRSTS Trace Plot by Location


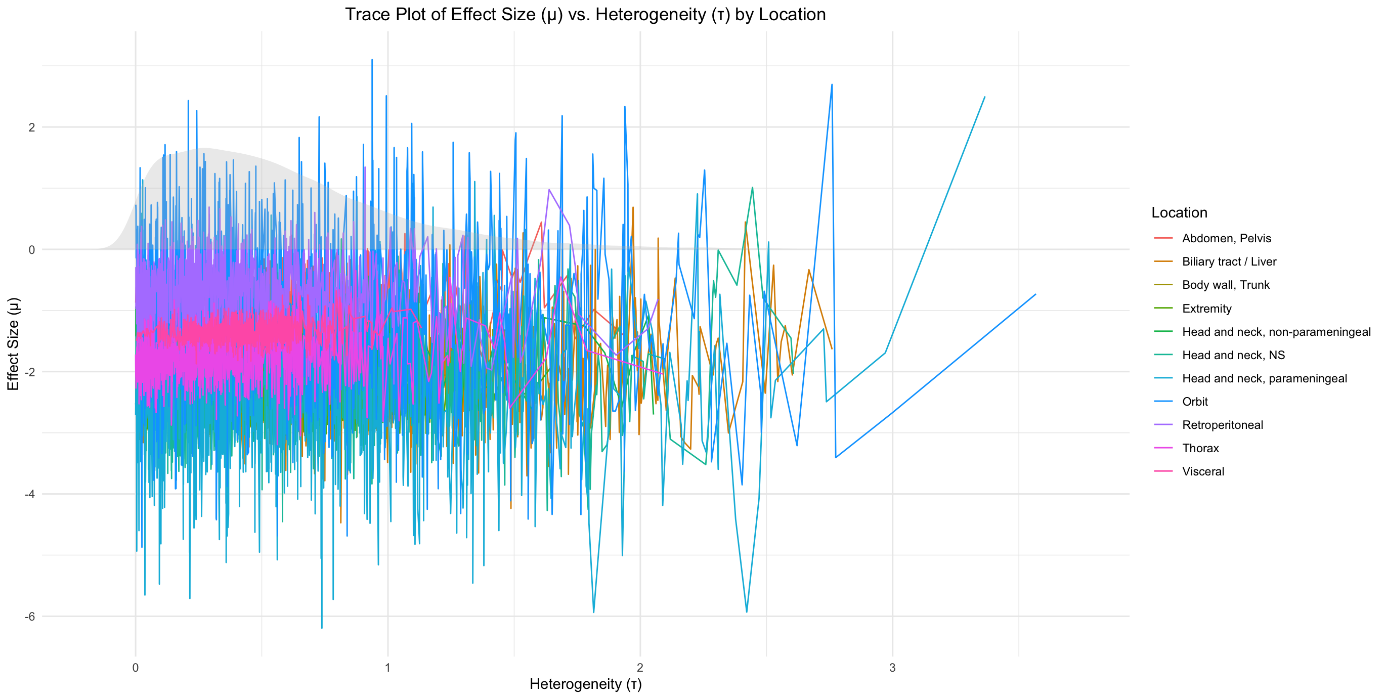

Supplement: Supplementary Fig. S3 [file mmc28.docx]
